# IPNA clinical practice recommendations for the diagnosis and management of children with steroid-resistant nephrotic syndrome

**DOI:** 10.1007/s00467-020-04519-1

**Published:** 2020-05-07

**Authors:** Agnes Trautmann, Marina Vivarelli, Susan Samuel, Debbie Gipson, Aditi Sinha, Franz Schaefer, Ng Kar Hui, Olivia Boyer, Moin A Saleem, Luciana Feltran, Janina Müller-Deile, Jan Ulrich Becker, Francisco Cano, Hong Xu, Yam Ngo Lim, William Smoyer, Ifeoma Anochie, Koichi Nakanishi, Elisabeth Hodson, Dieter Haffner, on behalf of the International Pediatric Nephrology Association

**Affiliations:** 1 Division of Pediatric Nephrology, Center for Pediatrics and Adolescent Medicine, Heidelberg, Germany; 2 Department of Pediatric Subspecialties, Division of Nephrology and Dialysis, Bambino Gesù Pediatric Hospital and Research Center, Rome, Italy; 3 Department of Pediatrics, Section of Pediatric Nephrology, Alberta Children’s Hospital, University of Calgary, Calgary, Canada; 4 Division of Nephrology, University of Michigan, Ann Arbor, MI USA; 5 Department of Pediatrics, Division of Nephrology, All India Institute of Medical Sciences, New Delhi, India; 6 Department of Paediatrics, Yong Loo Lin School of Medicine, National University of Singapore, Singapore, Singapore; 7 Laboratory of Hereditary Kidney Diseases, Imagine Institute, INSERM U1163, Paris Descartes University, Paris, France; 8 Department of Pediatric Nephrology, Reference Center for Idiopathic Nephrotic Syndrome in Children and Adults, Necker Hospital, APHP, 75015 Paris, France; 9 Department of Pediatric Nephrology, Bristol Royal Hospital for Children, University of Bristol, Bristol, UK; 10 Hospital Samaritano and HRim/UNIFESP, Federal University of São Paulo, São Paulo, Brazil; 11 Department of Nephrology, University Hospital Erlangen, Erlangen, Germany; 12 Institute of Pathology, University Hospital of Cologne, Cologne, Germany; 13 Department of Nephrology, Luis Calvo Mackenna Children’s Hospital, University of Chile, Santiago, Chile; 14 Department of Nephrology, Children’s Hospital of Fudan University, Shanghai, China; 15 Department of Pediatrics, Prince Court Medical Centre, Kuala Lumpur, Malaysia; 16 The Research Institute at Nationwide Children’s Hospital, The Ohio State University, Columbus, OH USA; 17 Department of Paediatrics, University of Port Harcourt Teaching Hospital, Port Harcourt, Rivers State Nigeria; 18 Department of Child Health and Welfare (Pediatrics), Graduate School of Medicine, University of the Ryukyus, Okinawa, Japan; 19 Cochrane Kidney and Transplant, Centre for Kidney Research, The Children’s Hospital at Westmead and the Sydney School of Public Health, University of Sydney, Sydney, Australia; 20 Department of Paediatric Kidney, Liver and Metabolic Diseases, Hannover Medical School Children’s Hospital, Hannover, Germany; 21 Department of Paediatric Kidney, Liver and Metabolic Diseases, Paediatric Research Center, Hannover Medical School, Carl-Neuberg-Str. 1, 30625 Hannover, Germany; 22 Center for Rare Diseases, Hannover Medical School Children’s Hospital, Hannover, Germany

**Keywords:** Steroid-resistant nephrotic syndrome, Children, Chronic kidney disease, Genetics, Outcome, Pediatrics, Immunosuppressive treatment

## Abstract

Idiopathic nephrotic syndrome newly affects 1–3 per 100,000 children per year. Approximately 85% of cases show complete remission of proteinuria following glucocorticoid treatment. Patients who do not achieve complete remission within 4–6 weeks of glucocorticoid treatment have steroid-resistant nephrotic syndrome (SRNS). In 10–30% of steroid-resistant patients, mutations in podocyte-associated genes can be detected, whereas an undefined circulating factor of immune origin is assumed in the remaining ones. Diagnosis and management of SRNS is a great challenge due to its heterogeneous etiology, frequent lack of remission by further immunosuppressive treatment, and severe complications including the development of end-stage kidney disease and recurrence after renal transplantation. A team of experts including pediatric nephrologists and renal geneticists from the International Pediatric Nephrology Association (IPNA), a renal pathologist, and an adult nephrologist have now developed comprehensive clinical practice recommendations on the diagnosis and management of SRNS in children. The team performed a systematic literature review on 9 clinically relevant PICO (Patient or Population covered, Intervention, Comparator, Outcome) questions, formulated recommendations and formally graded them at a consensus meeting, with input from patient representatives and a dietician acting as external advisors and a voting panel of pediatric nephrologists. Research recommendations are also given.

**Electronic supplementary material:**

The online version of this article (10.1007/s00467-020-04519-1) contains supplementary material, which is available to authorized users.

## Introduction

Idiopathic nephrotic syndrome (NS), characterized by severe proteinuria, hypoalbuminemia, and/or presence of edema [1, 2], newly affects about 1–3 per 100,000 children aged below 16 years [3–5]. Approximately 85% of cases experience complete remission of proteinuria following daily oral prednisolone/prednisone (PDN) treatment at standard doses [6]. Those who do not achieve remission after 4–6 weeks of treatment are presumed to have steroid resistant NS (SRNS) [7]. The duration of PDN required before a patient is considered steroid-resistant is a matter of discussion and longer treatment periods (6–8 weeks), as well as additional intravenous methylprednisolone (MPDN) pulses, have been reported [6].

In 10–30% of patients with non-familial SRNS, mutations in podocyte-associated genes can be detected, whereas an undefined circulating factor(s) is assumed in the remaining cases [8–10]. The principal histopathological entities encountered in SRNS are focal and segmental glomerulosclerosis (FSGS), minimal change disease (MCD), and diffuse mesangial sclerosis. Treatment usually includes inhibitors of the renin-angiotensin-aldosterone system (RAASi) and calcineurin inhibitors (CNI) in patients with non-genetic forms of SRNS. With this approach, complete or partial remission can be achieved in 50–70% of cases [6, 7].

Management of SRNS is a great challenge due to its heterogeneous etiology, frequent lack of remission induced by immunosuppressive treatment, and complications including drug toxicity, infections, thrombosis, the development of end-stage kidney disease (ESKD), and recurrence after renal transplantation [11]. There are currently no evidence-based, systematically developed recommendations on the diagnosis and management of children with SRNS available, with the exception of a focused document from KDIGO (Kidney Disease: Improving Global Outcomes) Glomerulonephritis guideline [6]. Therefore, the International Pediatric Nephrology Association (IPNA) convened a clinical practice recommendation (CPR) workgroup in December 2018 to develop CPRs for the diagnosis and management of children with SRNS. Future research recommendations regarding key outcome measures in patients with SRNS are also presented.

## Methods

### Overview of the guideline project

We have followed the RIGHT (Reporting Items for practice Guidelines in HealThcare) Statement for Practice Guidelines [12]. Three groups were assembled: a core leadership group, an external expert group, and a voting panel. The core group comprised 18 members of IPNA, including pediatric nephrologists, renal geneticists, epidemiologists, an adult nephrologist, and a renal pathologist. The individual expertise and responsibilities of the core group members are given in Supplementary Table S1. The external expert group included 3 patient representatives and one dietician. The patient representatives discussed the manuscript provided by the core group members within their local parents’ association, and their suggestions were then incorporated into the manuscript. The voting panel included 23 pediatric nephrologists including 3–5 representatives of each IPNA Regional Society with expertise in the management of SRNS in children. Voting group members were asked by electronic questionnaire to provide a level of agreement on a 5-point scale (strongly disagree, disagree, neither agree/disagree, agree, strongly agree) (Delphi method). For topics that failed to achieve a 70% level of consensus, the recommendations were re-evaluated and modified by the core group and then reviewed again by the voting panel until a consensus level of > 70% was achieved.

### Developing the PICO questions

We developed PICO (Patient or Population covered, Intervention, Comparator, Outcome) questions as follows [13]: *Population:* Children (> 3 months and < 18 years) with SRNS; *Intervention and Comparators:* treatment compared with no treatment, other treatment or placebo; *Outcomes Addressed:* We addressed recommendations for the diagnosis, treatment, and follow-up of children with SRNS (including efficacy to induce remission and side effects of medications).

### Literature search

The PubMed database was searched for studies published by 15 September 2019; all systematic reviews of randomized controlled trials (RCTs) on treatment of SRNS in children, RCTs, prospective uncontrolled trials, observational studies, and registry studies on diagnosis and treatment of children with SRNS, restricted to human studies in English. Where possible, meta-analyses of RCTs using risk ratios were cited from the updated Cochrane systematic review regarding interventions for childhood steroid resistant NS (SRNS) [14]. Further details and a summary of the publications used for this CPR are given in the Supplementary material (Supplementary Tables S2–S5**)**.

### Grading system

**Fig. 1:**
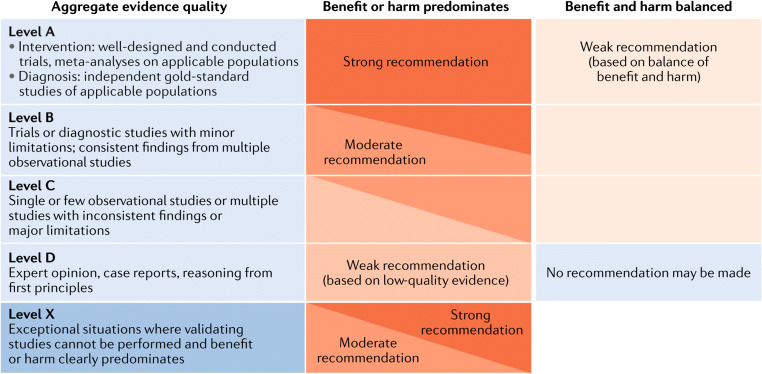
Matrix for grading of evidence and assigning strength of recommendations as currently used by the American Academy of Pediatrics. Reproduced with permission from [15]

We followed the grading system of the American Academy of Pediatrics (Fig. 1**;** [16]). The quality of evidence was graded as High (A), Moderate (B), Low (C), Very low (D), or Not applicable (X). The latter refers to exceptional situations where validating studies cannot be performed because benefit or harm clearly predominates. This letter was used to grade contra-indications of therapeutic measures and safety parameters. The strength of a recommendation was graded as strong, moderate, weak, or discretionary (when no recommendation can be made).

### Limitations of the guideline process

SRNS is a rare disease. Consequently, the sizes and numbers of some RCTs were small and of poor methodological quality so most recommendations are weak to moderate. Due to the limited budget of this IPNA initiative, patient representatives and dieticians were only included as external experts.

## Clinical practice recommendations

### Definitions and diagnostic work-up

#### Definitions

- We recommend quantification of proteinuria by protein/creatinine ratio (UPCR) in either a first morning (AM) urine or 24-h urine sample at least once before defining a patient as SRNS and/or starting alternative immunosuppression. We suggest using this baseline value for assessment of subsequent response (grade A, strong recommendation).
- We suggest using the definitions listed in Table 1 for the diagnosis and management of SRNS (grade B, moderate recommendation).
- We suggest using the “confirmation period,” which is the time period between 4 and 6 weeks from start of oral PDN at standard doses, to assess the response to further treatment with glucocorticoids and initiate RAASi (grade C, weak recommendation). We also recommend performing genetic testing and/or a renal biopsy at this time (grade B, moderate recommendation).
- We suggest the submission of histological, clinical, and genetic data from all SRNS patients into patient registries and genetic databases to help improve our understanding of the disease and its treatment (ungraded).

**Table 1:** Definitions relating to nephrotic syndrome in children

Term	Definition
Nephrotic-range proteinuria	UPCR ≥ 200 mg/mmol (2 mg/mg) in first morning void or 24 h urine sample ≥ 1000 mg/m^2^/day corresponding to 3+ or 4+ by urine dipstick
Nephrotic syndrome	Nephrotic-range proteinuria and either hypoalbuminemia (serum albumin < 30 g/l) or edema when serum albumin level is not available
SSNS	Complete remission within 4 weeks of prednisone or prednisolone (PDN) at standard dose (60 mg/m^2^/day or 2 mg/kg/day, maximum 60 mg/day).
SRNS	Lack of complete remission within 4 weeks of treatment with PDN at standard dose
Confirmation period	Time period between 4 and 6 weeks from PDN initiation during which response to further oral PDN and/or pulses of iv MPDN and RAASi are ascertained in patients achieving only partial remission at 4 weeks. A patient achieving complete remission at 6 weeks is defined as a late responder. A patient not achieving complete remission at 6 weeks although he had achieved partial remission at 4 weeks is defined as SRNS.
Complete remission	UPCR (based on first morning void or 24 h urine sample) ≤ 20 mg/mmol (0.2 mg/mg) or negative or trace dipstick on three or more consecutive occasions.
Partial remission	UPCR (based on first morning void or 24 h urine sample) > 20 but < 200 mg/mmol and, if available, serum albumin ≥ 30 g/l.
Relapse	Recurrence of nephrotic-range proteinuria. In children, relapse is commonly assessed by urine dipstick and is thus defined as dipstick ≥ 3+ on 3 consecutive days, or UPCR ≥ 200 mg/mmol (2 mg/mg) on a first morning urine sample, with or without reappearance of edema in a child who had previously achieved partial or complete remission.
CNI-resistant SRNS	Absence of at least partial remission after 6 months of treatment with a CNI at adequate doses and/or levels.
Multi-drug-resistant SRNS	Absence of complete remission after 12 months of treatment with 2 mechanistically distinct steroid-sparing agents at standard doses (see text).
Secondary steroid resistance	Children with initial steroid-sensitivity who in subsequent relapses develop SRNS
Recurrent nephrotic syndrome post-renal transplantation	A child with SRNS presenting post-renal transplantation with a relapse of nephrotic-range proteinuria in the absence of other apparent causes and/or podocyte foot process effacement on kidney biopsy. This diagnosis should also be considered in case of persistent proteinuria (UPCR ≥ 100 mg/mmol (1 mg/mg) in a previously anuric patient, or an increase of UPCR ≥ 100 mg/mmol (1 mg/mg) in a patient with prevalent proteinuria at the time of transplant in the absence of other apparent causes.

*UPCR* urine protein/creatinine ratio, *SSNS* steroid sensitive nephrotic syndrome, *SRNS* steroid-resistant nephrotic syndrome, *PDN* prednisolone or prednisone, *MPDN* methylprednisolone, *RAASi* renin-angiotensin-aldosterone system, *CNI* calcineurin inhibitor

## Evidence and rationale

### Assessment of proteinuria

The conventional definition of NS in children is proteinuria > 40 mg/h/m^2^ or ≥ 1000 mg/m^2^/day or urinary protein creatinine ratio (UPCR) ≥ 200 mg/mmol (2 mg/mg) or 3+ on urine dipstick plus either hypoalbuminemia (< 30 g/l) or edema [17]. Urinary dipstick analysis is useful for screening and at home monitoring of proteinuria, but therapeutic decisions should be based on at least one precise quantification of proteinuria, i.e., UPCR on a first-morning urine sample, or 24-h urine collection after treatment for > 4 weeks with full-dose PDN. First-morning urine samples are preferred over random spot samples to reduce the influence of orthostatic proteinuria [18, 19]. Given the linear relationship between UPCR in spot and 24-h urine protein, determination of UPCR is recommended. If either UPCR measurement is ≥ 200 mg/mmol (2 mg/mg), then treatment for SRNS should begin. Semiquantitative expression of dipstick results is given in Supplementary Table S6.

### Definition of SRNS

**Fig. 2:**
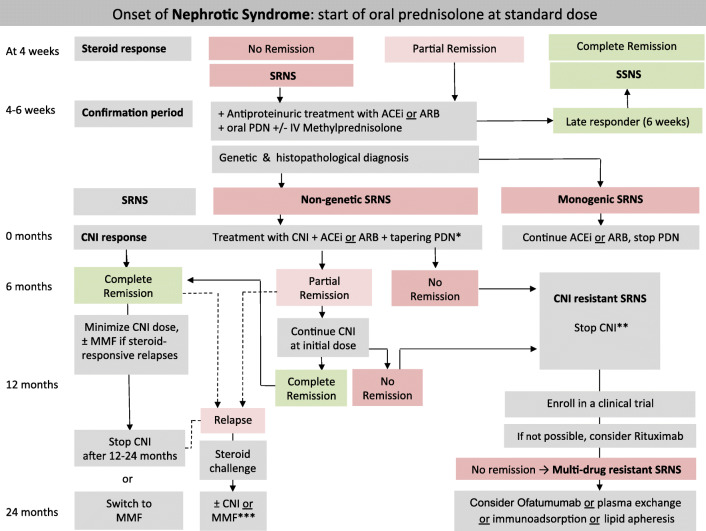
Algorithm for the management of children with nephrotic syndrome. Patients are characterized according to response to a 4-week treatment with oral prednisolone (PDN). Patients showing no complete remission enter the confirmation period in which responses to further oral prednisolone (PDN) with or without methylprednisolone (MPDN) pulses in conjunction with either angiotensin-converting enzyme inhibitors (ACEi) or angiotensin-receptor blockers (ARBs) are ascertained and genetic and histopathological evaluation is initiated. Patients with non-genetic SRNS should be candidates for further immunosuppression, whereas those with monogenetic forms are not (further details are given in the text). In the setting of low resource countries where genetic and/or histopathology assessment is not available, immediate immunosuppressive treatment with CNI may be started. If CNI are not available intravenous or oral cyclophosphamide may be started. * = We suggest tapering PDN after CNI initiation as follows: 40 mg/m^2^ QOD for 4 weeks, 30 mg/m^2^ QOD for 4 weeks, 20 mg/m^2^ QOD for 4 weeks, 10 mg/m^2^ QOD for 8 weeks, and discontinuing thereafter; ** = CNI may be continued in case of partial remission; *** = in cases of no complete response within 4 weeks, frequent relapses or side effects of medications, we recommend following the refractory SRNS protocol; SRNS, steroid-resistant nephrotic syndrome; ACEi, angiotensin-converting enzyme inhibitor; ARB, angiotensin-receptor blocker; PDN, prednisolone; IV, intravenous; CNI, calcineurin inhibitor; MMF, mycophenolate mofetil

The *initial treatment of children with idiopathic NS* usually comprises oral PDN 60 mg/m^2^/day or 2 mg/kg/day (maximum 60 mg/day) for 4–6 weeks, followed by 40 mg/m^2^ or 1.5 mg/kg per dose on alternate days (QOD) for another 4–6 weeks. After the initial 4 weeks of full-dose oral PDN, a child can achieve complete remission (UPCR ≤ 20 mg/mmol (0.2 mg/mg) or negative or trace dipstick on three or more consecutive occasions), which confirms SSNS. If partial remission is observed, given the fact that a small percentage of children achieve complete remission if given 2 additional weeks of time, the “confirmation period” begins. During this time, responses to further daily oral PDN with or without 3 pulses of MPDN (500 mg/m^2^ or 15 mg/kg), and RAASi are ascertained (Fig. 2). If complete remission is achieved by 6 weeks, the child is defined as “late responder” SSNS and treated as SSNS. If no remission is achieved by 6 weeks, the diagnosis of SRNS is confirmed (Fig. 2). We recommend performing a renal biopsy as well as obtaining genetic testing results (where available) as soon as possible, ideally within the 2-week confirmation period. If genetic results are not available at the end of the confirmation period, we suggest to start treatment with CNI and to reassess treatment after receiving genetic results. In the setting of low-resource countries where genetic and/or histopathology assessment is not available, immediate immunosuppressive treatment with CNI may be started. If CNI are not available intravenous or oral cyclophosphamide (CPH) may be started (vide infra). Details on evidence and rationale for these definitions are given in the Supplementary Material.

### Definition of CNI-resistant nephrotic syndrome

Among those children defined as SRNS without a genetic cause, a substantial proportion will respond to CNIs in a variable amount of time (weeks to months). Children with initial SRNS who are CNI responders subsequently either remain in stable remission with no or infrequent relapses or may develop secondary SSNS. Resistance to CNIs is defined when a child fails to attain at least partial remission after at least 6 months of CNI treatment administered at adequate doses and blood levels.

### Definition of multi-drug resistant nephrotic syndrome

Children resistant to CNIs may be treated with other steroid-sparing agents (see “Developing the PICO questions”; Fig. 2 and Supplementary Table S2). Patients with SRNS are defined as “multi-drug resistant” in the absence of complete remission after 12 months of treatment with 2 mechanistically distinct steroid-sparing agents (including CNIs) administered at standard doses.

#### Initial diagnostic workup of a child with SRNS

- We recommend obtaining a careful family history for renal and extra-renal manifestations including asking about consanguinity. Where renal diseases are present in family members, the age at onset, clinical course including response to medications, renal function, and renal biopsy and genetic testing results should be obtained wherever possible (grade A, strong recommendation).
- We recommend careful physical examination of the patient including a meticulous search for extra-renal manifestations such as skeletal, neurological, eye, ear and urogenital abnormalities, and for secondary causes (mainly infectious) of NS (Table 2**)** (grade A, strong recommendation).
- We suggest that the blood, serum, and urine tests listed in Table 2 be performed to search for immunological or infectious causes of SRNS and to evaluate the degree of proteinuria, estimated GFR, and renal histology (grade B, moderate recommendation).
- We suggest offering urinalysis to siblings of SRNS patients even before genetic testing is done (grade C, moderate recommendation).

**Table 2:** Initial workup and follow-up for a child with steroid-resistant nephrotic syndrome

Investigations	Initial workup	Follow-up monitoring
Clinical evaluation		
Patient history - Including results of dipstick assessments at home, physical activity, fever episodes, pain, abdominal discomfort, swelling, fatigue, school attendance, adherence to medication, menstrual cycle in female adolescents	✓	Every 3 months
- Search for risk factors for secondary causes (sickle cell disease, HIV, SLE, HepB, malaria, parvovirus B19)	✓	As appropriate
- Check for tuberculosis in endemic areas before starting immunosuppressant drugs	✓	As appropriate
Physical examination - Assessing fluid status including signs of edema (e.g., ascites, pericardial & pleural effusions), tetany, lymphadenopathy	✓	Every 3 months
- Drug toxicity (e.g., eyes, skin)		Every 3 months
- Skeletal status	✓	Every 3 months
- Extrarenal features, e.g., dysmorphic features or ambiguous genitalia	✓ ✓	As appropriate
Full neurological examination & standardized assessment of cognitive status	✓	Every 12 months or as appropriate
Pubertal status: Tanner stage, testicular volume in boys (in patients aged > 10 years)	✓	Every 12 months
Vital parameters: blood pressure	✓	Every 3 months; yearly 24 h ambulatory BP monitoring in patients with hypertension, if feasible
Anthropometry^a^: - Growth chart: height/length, weight, - Head circumference < 2 years - Calculation of BMI and annual height velocity	✓	Every 3 months (monthly in infants)
Vaccination status - Check and complete, especially for encapsulated bacteria—pneumococcal, meningococcal, hemophilus influenza, and varicella-zoster if available	✓	Every 12 month or as appropriate
Family history - Renal and extrarenal manifestations - Consanguinity	✓	Every 12 month or as appropriate
Biochemistry		
Urine Spot urine (first morning void) or 24 h urine: protein/creatinine	✓ Essential	Every 3 months (more frequently until remission)
Urinalysis including hematuria	✓	Every 6–12 months
Spot urine: calcium/creatinine ratio, low molecular weight proteinuria (e.g., α_1_-microglobulin/creatinine ratio	Conditional	
Blood Complete blood count (CBC) Creatinine, BUN, or urea Electrolytes (including ionized calcium, potassium*, and albumin corrected albumin if available) Serum albumin, total protein Blood gas analysis (HCO_3_)	✓ Essential	Every 3 months (more frequently until remission and in CKD stage 4–5) Every day or every other day when using high dose diuretics
C-reactive protein	✓	As required (clinical decision)
Estimated GFR^b^	✓	Every 3 months (more frequently in CKD stage 4)
ALP, PTH, 25(OH) vitamin D	✓	Every 12 months (more frequently in patients with CKD stages 3–5)
Lipid profile (LDL- and HDL-cholesterol, triglycerides)	✓	Every 12 months or as appropriate
Baseline coagulation tests (prothrombine time (INR), aPTT, fibrinogen, ATIII), detailed thrombophilic screening in patients with reported previous thrombotic events, central venous lines, persistent nephrotic range proteinuria, and/or increased familial history for thrombotic events	✓	At diagnosis and then as appropriate, e.g., in case of relapses
Thyroid function (T3, FT4, TSH)	✓	Every 12 months or as appropriate especially in patients with prolonged proteinuria
Immunoglobulin G	✓	In case of recurrent infections
Glucose/fasting glucose	✓	Every 6 months or as appropriate
HbA1c	✓	Every 12 months or as appropriate
C3, antinuclear antibodies ds-DNA, ENA, ANCA	✓ Conditional	As appropriate As appropriate
HBs-Ag, anti-HCV-IgG, syphilis, and HIV tests	✓	Before prednisolone and as appropriate
Vaccination status including blood titer tests	✓	Yearly or as appropriate
Genetics		
Next-generation sequencing (NGS)/Whole Exome Sequencing (WES)	✓	Extended genetic screening for patients with SRNS depending on new findings (Table 3); whole exome sequencing if indicated Before transplantation, if not previously performed
Drug-specific monitoring		
CsA and Tacrolimus: Drug trough levels	–	Weekly during titration period (for 4 weeks), thereafter every 3 months or as appropriate
MMF: mycophenolic acid kinetic (2 h)^c^	–	AUC after 4 weeks of treatment, thereafter every 6–12 months or as appropriate
Rituximab	–	CD19 B cell count: baseline, 1 month after the first dose (nadir), every 1–3 months until B cell recovery
Statins: creatinine kinase (CK)	–	If on statins, every 6 months
Prolonged glucocorticoid therapy	- Conditional	Ophthalmological examination for cataract and intraocular pressure Bone mineral density by lumbar DEXA
Imaging		
Renal ultrasound: renal echogenicity and size of kidneys	✓	At presentation (mandatory prerenal biopsy)
Ultrasound of abdomen & pleural space (ascites, effusions, thrombosis)	✓	as appropriate
Cardiac ultrasound (left ventricular mass, effusions)	✓	Every 12 months in hypertensive patients or in case of severe edema
Chest X-ray	✓ Optional	If indicated
X-ray of the left wrist (bone age assessment in children aged > 5 years, mineralization)	✓	Every 12 months or as appropriate
Histopathology		
Renal biopsy	✓	See text: at diagnosis, and subsequently if indicated: in case of unexplained drop in eGFR, unexplained increase in proteinuria, to rule out and/or to monitor CNI nephrotoxicity during prolonged (< 2 years) treatment
Dietary assessment		
Dietician review and advice by a dietician regarding salt, potassium, caloric and protein intake	✓	Every 3 months (more frequently in infants, malnourished patients, and patients with CKD stage 4–5)
Assessment for extrarenal involvement		
Depending on underlying disease and clinically evident extrarenal features: - *Brain MRI* (e.g., microcephaly, psychomotor delay, mental retardation, myoclonic epilepsy, tremor, ataxia, hypotonia) - *Interdisciplinary evaluation* by *Ophthalmology* (e.g., microcoria, cataract, glaucoma, optic atrophy, keratoconus, macular spots, lenticonus, nystagmus), - *Cardiology* (e.g., congenital heart defects), - *Endocrinology* (ambiguous genitalia, delayed puberty, primary amenorrhea, pesudohermaphroditism, diabetes mellitus), - *Dermatology* (e.g., epidermolysis bullosa), - *Orthopedics* (absent or hypoplastic patella, spondyloepiphyseal dysplasia), - *Immunology* (T cell immunodeficiency), - *Hematology* (thrombocytopenia with large platelets, Döhle bodies), - Audiology (sensorineural hearing loss)	✓ If indicated	If indicated

*ALP* alkaline phosphatase, *PTH* parathyroid hormone, *CNI* calcineurin inhibitor, *CsA* cyclosporine A, *BP* blood pressure, *MMF* mycophenolate mofetil

^a^Anthropometric data should be compared with updated national or international (WHO charts [20]) standards

^b^eGFR (ml/min/1.73 m^2^) = *k* height (cm)/plasma creatinine (mg/dl); where *k* is a constant = 0.413. In malnourished or obese patients cystatin-based equations should be used [21]

^c^According to Gellerman et al. [22]

## Evidence and rationale

Early identification of genetic forms of SRNS (listed in Table 3) is important, as these patients are unlikely to benefit from prolonged and potentially harmful immunosuppression. Delineation of family history to recognize familial forms and a careful physical examination to identify extra-renal features (given in Supplementary Table S7) of genetic conditions are essential. Occasionally, SRNS can be secondary to infectious causes, mainly cytomegalovirus (CMV), human immunodeficiency virus (HIV), hepatitis B, malaria, parvovirus B19, and syphilis. Other causes of SRNS can be sickle-cell disease, lymphoma, membranous nephropathy, membranoproliferative glomerulonephritis, C3 glomerulopathy, IgA nephropathy, systemic lupus erythematosus, Alport syndrome/collagen IV glomerulopathy, amyloidosis, and thrombotic microangiopathy (TMA). Workup for these conditions should be considered especially in patients presenting with a reduced estimated GFR (eGFR) and may include kidney biopsy, genetic testing, and/or assessment of complement C3, C4, antinuclear antibodies, anti-streptococcal antibodies, and ANCA. Later in the disease course, a low eGFR may signal progression of disease, acute kidney injury (AKI), or drug toxicity. Renal ultrasound including Doppler evaluation assists with evaluation of congenital abnormalities of the kidney and urinary tract and vascular thrombosis, which can also be a cause of proteinuria. Given the 25% risk of disease in siblings if a patient has autosomal recessive SRNS, urinalysis is advisable for siblings.

**Table 3:** Genes to be included in Next Generation Sequencing (from [8]) in a child with SRNS

Gene	Inheritance	Accession no.	Disease
*ACTN4*∗	AD	NM_004924	Familial and sporadic SRNS (usually adult)
*ADCK4*^∗^	AR	NM_024876	SRNS
*ALG1*	AR	NM_019109	Congenital disorder of glycosylation
*ANKFY1*	AR	NM_001330063.2	Pediatric SRNS
*ANLN*	AD	NM_018685	FSGS (mainly adult)
*ARHGAP24*	AD	NM_001025616	FSGS
*ARHGDIA*	AR	NM_001185078	CNS
*AVIL*	AR	NM_006576.3	SRNS
*CD151*	AR	NM_004357	NS, pretibial bullous skin lesions, neurosensory deafness, bilateral lacrimal duct stenosis, nail dystrophy, and thalassemia minor
*CD2AP*	AD/AR	NM_012120	FSGS/SRNS
*CFH*	AR	NM_000186	MPGN type II + NS
*CLCN5*	XR	NM_001127898.4	Dent’s disease ± FSGS ± hypercalcuria and nepthrolithiasis
*COL4A3*^∗^	AR	NM_000091	Alport’s disease/FSGS
*COL4A4*	AR	NM_000092	Alport’s disease/FSGS
*COL4A5*∗	XR	NM_000495	Alport’s disease/FSGS
*COQ2*	AR	NM_015697	Mitochondrial disease/isolated nephropathy
*COQ6*	AR	NM_182476	NS ± sensorineural deafness; DMS
*CRB2*^∗^	AR	NM_173689	SRNS
*CUBN*	AR	NM_001081	Intermittent nephrotic range proteinuria ± with epilepsy
*DGKE*^∗^	AR	NM_003647	Hemolytic-uremic syndrome, SRNS
*DLC1*	AR	NM_182643.3	Childhood and adult SSNS and SRNS
*E2F3*	AD	NM_001949	FSGS + mental retardation (whole gene deletion)
*EMP2*	AR	NM_001424	Childhood-onset SRNS and SSNS
*FAT1*	AR	NM_005245.4	Combination of SRNS, tubular ectasia, hematuria, and facultative
*FN1*	AD?	NM_212482.3	Fibronectin glomerulopathy
*GAPVD1*	AR	NM_001282680.3	Early-onset NS
*INF2*	AD	NM_022489	Familial and sporadic SRNS, FSGS-associated Charcot-Marie-Tooth neuropathy
*ITGA3*	AR	NM_002204	Congenital interstitial lung disease, nephrotic syndrome, and mild epidermolysis bullosa
*ITGB4*	AR	NM_000213	Epidermolysis bullosa and pyloric atresia + FSGS
*ITSN1*	AR	NM_003024.3	CNS/SRNS/SSNS (with MCD/FSGS on biopsy)
*ITSN2*	AR	NM_019595.4	SSNS/SDNS (with MCD/MPGN on biopsy)
*KANK1*	AR	NM_015158	SSNS
*KANK2*	AR	NM_015493	SSNS/SDNS ± hematuria
*KANK4*	AR	NM_181712	SRNS + hematuria
*KIRREL1*	AR	NM_018240.7	SRNS
*LAGE3*	AR	NM_006014.4	NS with primary microcephaly
*LAMA5*	AR	NM_005560.6	Childhood NS
*LAMB2*^∗^	AR	NM_002292	Pierson syndrome
*LCAT*	AR	NM_000229.2	Norum disease
*LMNA*	AD	NM_170707	Familial partial lipodystrophy + FSGS
*LMX1B*^∗^	AD	NM_002316	Nail patella syndrome; also FSGS without extrarenal involvement
*MAFB*	AD	NM_005461.5	FSGS with Duane retraction syndrome
*MAGI2*	AR	NM_012301.4	NS ± neurological impairment
*MMACHC*	AR	NM_015506.3	Cobalamin C deficiency, TMA, and nephrotic syndrome
*MYO1E*^∗^	AR	NM_004998	Familial SRNS
*NEU1*	AR	NM_000434.4	Nephrosialidosis (sialidosis type II + childhood NS)
*NPHP4*	AR	NM_015102.5	Nephronophthisis with FSGS and nephrotic range proteinuria
*NPHS1*^∗^	AR	NM_004646	CNS/SRNS
*NPHS2*^∗^	AR	NM_014625	CNS, SRNS
*NUP85*	AR	NM_024844.5	SRNS
*NUP93*^∗^	AR	NM_014669	Childhood SRNS
*NUP107*^∗^	AR	NM_020401	Childhood SRNS
*NUP160*	AR	NM_015231.2	SRNS
*NUP205*	AR	NM_015135	Childhood SRNS
*NXF5*	XR	NM_032946	FSGS with co-segregating heart block disorder
*OCRL*^∗^	XR	NM_000276	Dent’s disease-2, Lowe syndrome, ± FSGS, ± nephrotic range proteinuria
*OSGEP*	AR	NM_017807.4	NS with primary microcephaly
*PAX2*	AD	NM_003987	Adult-onset FSGS without extrarenal manifestations
*PDSS2*	AR	NM_020381	Leigh syndrome
*PLCe1*	AR	NM_016341	CNS/SRNS
*PMM2*	AR	NM_000303	Congenital disorder of glycosylation
*PODXL*^∗^	AD	NM_005397	FSGS
*PTPRO*	AR	NM_030667	NS
*SCARB2*	AR	NM_005506	Action myoclonus renal failure syndrome ± hearing loss
*SGPL1*	AR	NM_003901.4	Primary adrenal insufficiency and SRNS
*SMARCAL1*	AR	NM_014140	Schimke immuno-osseous dysplasia
*SYNPO*	AD	NM_007286	Sporadic FSGS (promoter mutations)
*TBC1D8B*	XR	NM_017752.3	Early-onset SRNS with FSGS
*TNS2*	AR	NM_170754.3	SSNS/SDNS (with MCD/FSGS/DMS on biopsy)
*TP53RK*	AR	NM_033550.4	NS with primary microcephaly
*TPRKB*	AR	NM_001330389.1	NS with primary microcephaly
*TRPC6*^∗^	AD	NM_004621	Familial and sporadic SRNS (mainly adult)
*TTC21B*	AR	NM_024753	FSGS with tubulointerstitial involvement
*WDR73*	AR	NM_032856	Galloway-Mowat syndrome (microcephaly and SRNS)
*WT1*^∗^	AD	NM_024426	Sporadic SRNS (children: may be associated with abnormal genitalia); Denys-Drash and Frasier syndrome
*XPO5*	AR	NM_020750	Childhood SRNS
*ZMPSTE24*	AR	NM_005857	Mandibuloacral dysplasia with FSGS
*MYH9*	AD/assoc.	NM_002473	MYH9-related disease; Epstein and Fechtner syndromes
*APOL1*^∗^	G1, G2 risk alleles	NM_003661	Increased susceptibility to FSGS and ESRD in African Americans, Hispanic Americans and in individuals of African descent

*AD* autosomal dominant, *AR* autosomal recessive, *CNS* congenital nephrotic syndrome, *DMS* diffuse mesangial sclerosis, *ESRD* end-stage renal disease, *FSGS* focal segmental glomerulosclerosis, *MPGN* membranoproliferative glomerulonephritis, *NS* nephrotic syndrome, *SDNS* steroid-dependent nephrotic syndrome, *SRNS* steroid resistant nephrotic syndrome, *SSNS* steroid sensitive nephrotic syndrome*Genes with a likely or known mutation, or a risk allele, in this cohort

### Indications for genetic testing and renal biopsy

- We recommend, if available, that genetic testing be performed in all children diagnosed with primary SRNS (grade B, moderate recommendation).
- We suggest giving priority to genetic testing in familial cases (family history of proteinuria/hematuria or CKD of unknown origin), cases with extra-renal features, and those undergoing preparation for renal transplantation (grade C, weak recommendation).
- We recommend a kidney biopsy in all children diagnosed with SRNS, except in known infection or malignancy-associated secondary disease or potentially in patients with familial and/or syndromic cases or genetic causes of SRNS (grade A, strong recommendation).
- We suggest genetic testing before a kidney biopsy in children with SRNS, especially in priority cases (see above), provided the results will be readily available (within few weeks) (grade D, weak recommendation).
- We do not recommend performing genetic testing in patients with initial steroid sensitivity who subsequently develop steroid resistance later in their disease course (i.e., secondary steroid resistance) (grade C, moderate recommendation).

## Evidence and rationale

### Genetic testing

Genetic testing in SRNS patients (i) may provide patients and families with an unequivocal diagnosis, (ii) may uncover a form of SRNS that is amenable to treatment (e.g., coenzyme Q10), (iii) may avoid the necessity of a renal biopsy and allow early weaning of immunosuppressive therapy, (iv) may allow accurate, well-informed genetic counseling including risk of recurrence post-transplantation [23, 24], and (v) may allow appropriate diagnosis and management of extrarenal manifestations [25, 26]. With whole exome sequencing (WES) technology, 10–30% of children are now diagnosed with a monogenic disease [8]. Mutations in *NPHS2*, *WT1*, and *NPHS1* are the most common genetic SRNS causes in European patients, accounting for 42, 16, and 13% of genetic cases, respectively [26]. Mutations in the *NPHS2* gene caused SRNS in ~ 20–30% of sporadic Caucasian cases [23]. The likelihood of identifying a causative mutation is inversely related to age at disease onset and is increased with either a positive family history or the presence of extrarenal manifestations [27], but genes commonly implicated in one population may not be common in another population [28–30]. In patients with monogenic forms of SRNS, immunosuppressive treatment should be withdrawn since there is evidence supporting the ineffectiveness of this treatment [31].

### Renal biopsy

Renal biopsy allows the exclusion of the other differential diagnoses listed above (e.g., membranous nephropathy) and the confirmation of a primary podocytopathy (MCD, FSGS, or DMS). Moreover, it allows the detection and grading of tubular atrophy, interstitial fibrosis, and glomerulosclerosis as prognostic markers [32, 33]. Therefore, once a child is defined as having SRNS, a renal biopsy should be performed according to current standards as described in Supplementary Material to determine the underlying pathology before initiating treatment with CNI, unless a clear monogenic form of SRNS known to be unresponsive to immunosuppression is identified. This is particularly relevant in settings where access to genetic testing is limited.

#### Genetic testing and counseling

- We recommend comprehensive gene panel analysis (i.e., a next generation sequencing panel to include all currently known SRNS genes, which is currently the most cost-effective approach to genetic testing) (genes are listed in Table 3) unless the clinical phenotype is suggestive of a specific condition, in which case we suggest performing a single gene analysis instead (grade B, moderate recommendation).
- We suggest determining the pathogenicity of identified genetic variants according to the guidelines of the American College of Medical Genetics [34]. Family segregation analysis may be performed in selected cases (grade B, moderate recommendation).
- We recommend genetic counseling for patients and their families to help them interpret both anticipated and unanticipated genetic findings (grade B, moderate recommendation).

## Evidence and rationale

We recommend performing genetic testing according to current standards [24, 35]. This includes confirmation of pathogenic or likely pathogenic variants by Sanger sequencing. In cases where no causative mutations are found in known gene panels, whole exome sequencing or whole genome sequencing may be considered, especially if the suspicion of a genetic etiology is high. Caution and expertise are required in interpreting variants of unknown significance [36]. Without genetic counseling patients and their families may not understand the significance of genetic findings [37].

### Screening for infections

- We recommend evaluation for subclinical tuberculosis according to country-specific guidelines (i.e., chest radiography, tuberculin test, quantiferon assay), if clinically suspected, or in case of residence in or travel from endemic areas (grade C, moderate recommendation).
- We suggest testing for hepatitis B, C, syphilis, and HIV: (i) to rule out secondary causes of NS and (ii) before immunosuppression, especially rituximab, given the endemicity of these infections in various countries (grade C, weak recommendation).

## Evidence and rationale

Accounting for country-specific disease prevalence and individual risk assessment, evaluations for infections causing secondary forms of SRNS should be completed.

### Treatment

#### First-line non-immunosuppressive treatment in children with SRNS

- We recommend starting RAASi with either angiotensin converting enzyme inhibitors (ACEi) *or* angiotensin receptor blockers (ARBs) once the diagnosis of SRNS is made (Fig. 2) (grade B, moderate recommendation).
- We suggest quantifying the change in first-morning proteinuria after starting RAASi therapy (grade D, weak recommendation).
- We suggest aiming for the maximum approved dosages given in Table S8 as tolerated (grade C, weak recommendation).
- ACEi or ARBs should be used with caution in patients with CKD stage 4, and they should not be started or should be stopped in case of intravascular volume depletion, acute kidney injury (AKI), hyperkalemia, or frequent vomiting/diarrhea (grade X, strong recommendation).
- We suggest using RAASi with non-renal metabolism (i.e., ramipril and ARBs) since they do not accumulate in renal failure (grade D, weak recommendation).
- In female adolescents, contraception should be ensured in order to avoid the teratogenic effects of RAASi (grade X, strong recommendation).

## Evidence and rationale

In CKD patients RAAS blockade by ACEi or ARBs decreases intraglomerular pressure, decelerates progression of CKD, and reduces proteinuria [38–42]. We recommend aiming for the maximum approved dosages as tolerated since dose-dependent antiproteinuric effects of ACEi with reductions of ~ 30% are expected [39]. Complete remissions have been reported in children with SRNS after therapy with ACEi or ARBs without additional medications other than PDN [43]. Therefore, in children with confirmed or suspected SRNS, this treatment may be commenced as early as 4 weeks from PDN initiation, during the so-called confirmation period. However, ACEi/ARBs may increase the risk for AKI, especially in patients with advanced CKD or intravascular volume depletion [44, 45]. Combined treatment with ACEi and ARBs is discouraged due to the increased risk for adverse events including AKI and death [46]. Agents with non-renal metabolism should be preferred since they do not accumulate in CKD (Table S8) [44]. Contraception is essential in female adolescents to avoid RAAS blocker fetopathy [47].

### First-line immunosuppressive treatment in children with SRNS

- We recommend that CNI (cyclosporine or tacrolimus) should be the first-line immunosuppressive therapy in children with SRNS and started once the diagnosis is confirmed (Fig. 2) (grade B, moderate recommendation).
- We suggest tapering PDN treatment once diagnosis of SRNS is established and discontinuing PDN therapy after 6 months (grade D, weak recommendation).
- We recommend withholding or delaying CNI treatment in patients with an eGFR < 30 ml/min/1.73 m^2^, AKI, and/or uncontrolled hypertension (grade X, strong recommendation).
- We recommend withholding CNI and stopping PDN treatment in patients with evidence for a monogenic form of SRNS (grade B, moderate recommendation).
- When CNIs are not available or unaffordable, we suggest using cyclophosphamide (CPH) [intravenous or po] with or without high-dose steroids (grade D, weak recommendation).
- We recommend making patients and families aware of potential side effects of immunosuppressive medication as given in Table 4 (grade X, strong recommendation).

**Table 4:** Common medication-related complications and side effects to be assessed for patient monitoring

Type of drug	Common medication-related side effect	Prevention
All	Recurrent infections (bacterial, viral, fungal)	Adequate but minimal dosing of immunosuppressive medication Vaccination (if feasible)
Glucocorticoids	Cushing syndrome Hypertension Glucose intolerance Growth retardation Reduced bone mineral density Cataracts, glaucoma Behavioral problems	Careful use of glucocorticoids No prolonged treatment Use of steroid-sparing agents
CNI	Hypertension Nephrotoxicity Neurotoxicity (tremor) Leg cramps Hypomagnesemia Interaction with other drugs	Adequate but minimal dosing of immunosuppressive medication, adapted by drug monitoring. Dose reduction in case of significant side effects
Tacrolimus-specific:	Glucose intolerance and diabetes mellitus	
Cyclosporine A-specific:	Hypertrichosis Gingival hyperplasia	
MMF	Hematology: - Leukopenia/neutropenia - Pancytopenia Gastrointestinal intolerance (nausea, vomiting, abdominal pain, diarrhea) Weight loss	Adequate but minimal dosing of immunosuppressive medication, adapted by drug monitoring
	Dermatological problems: - Verrucae - Neoplasm of the skin Neurological: - Headaches - Paraesthesia- Leg cramps	Additional sun/UV protection
RITUXIMAB	- Hep. B and fulminant hepatitis Specific Infections	- *Pneumocystis jirovecii* pneumonia
	Prophylaxis with cotrimoxazole Hypogammaglobulinemia Leukopenia/neutropeniaPancytopenia	Hepatitis B vaccination
	Acute infusion reactions - Angioedema - Bronchospasm, - Urticaria Progressive multifocal leukoencephalopathy (PML), induced by JC-Virus	Premedication

## Evidence and rationale

### Calcineurin inhibitors

The use of CNI as first-line therapy in children with SRNS was assessed in 8 RCTs comparing the efficacy of cyclosporine (CsA) with either placebo [48], no treatment [49, 50], intravenous MPDN [51], MMF with dexamethasone [52], or tacrolimus (TAC) [53, 54], and CsA or TAC with intravenous CPH [55, 56], on the outcome of “number with complete or partial remission” (Supplementary Table S2). CsA compared with placebo, no treatment, or intravenous MPDN showed superior outcome (~ 75% vs. 22%) irrespective of histopathology (risk ratio 3.50 (95% CI 1.04–9.57) [14]. There was no difference in outcome when TAC was compared with CsA (risk ratio 1.05 [95% CI 0.87–1.25]) [14, 53, 54]. CsA or TAC was more effective than intravenous CPH (78% vs. 40%; risk ratio 1.98 [95% CI 1.25–3.13]) [55, 56]. CsA compared with MMF in combination with dexamethasone was similarly effective (46% vs. 33%; risk ratio 1.38 [95% CI 0.9–2.10] [52]. TAC was more effective when compared with MMF in order to maintain remission (90% vs. 45%; risk ratio 2.01 [95% CI 1.32–3.07) [57]. When CsA was compared with placebo, no treatment, or MPDN, no differences were detected in the number of patients developing ESKD but event numbers were very small [48, 51, 58]. When CNIs were compared with intravenous CPH, there was an increase in serious adverse effects with CPH, but there were no differences in persistent nephrotoxicity or death [55]. No differences were detected in comparisons of CsA, MMF + dexamethasone, or TAC in terms of outcomes of ESKD, or 50% decline in eGFR [52, 53, 55, 57].

Treatment with CNIs is discouraged in patients with reduced eGFR, AKI, and/or uncontrolled hypertension due to their nephrotoxic effects. However, in patients with chronic CKD and no other option for disease control, CNIs may improve proteinuria and long-term kidney survival [59].

SRNS patients who do not show at least partial remission to CNI by 6 months are deemed CNI resistant, and those who do not respond to CNI plus another agent that is mechanistically distinct by 12 cumulative months of therapy as multi-drug resistant (vide supra). If a monogenic form of SRNS known not to respond to immunosuppression is identified in a patient and no response to immunosuppression has previously been observed in the patient, then immunosuppression should be discontinued. We suggest that patients in these categories remain off immunosuppression but continue on RAASi therapy until they reach advanced stages of CKD and can no longer tolerate RAASi **(**Fig. 2**)**.

### Alkylating agents and low resource settings

When compared with PDN/placebo, CPH showed no difference in the outcome of complete remission (risk ratio 1.06 95% CI 0.61–1.87) [60, 61]. Overall, 36% children on CPH compared with 35% on PDN achieved complete remission [60]*.* Similar remission rates were noted in patients receiving intravenous or oral CPH (each ~ 50%) [14, 62–64]. The response to CPH reported in some observational studies may indicate a certain overlap of SSNS and SRNS [65, 66]. Older studies may have included children with monogenic causes of NS, given that genetic testing was not commonly available for patients before 2000–2010 resulting in low response rates to CPH. CPH may be trialed to induce remission in resource-limited settings, but should be stopped in case of achieving no-response. Since chlorambucil was not evaluated in any RCTs, we make no suggestions for its use.

#### CNI schedule, monitoring, and co-interventions

- We suggest a starting CsA dose of 3–5 mg/kg/day (max starting 250 mg/day) given orally twice daily (grade B, weak recommendation).
- We suggest titrating the CsA dosage in at least daily intervals aiming for CsA whole blood trough levels between 80 and 120 ng/ml based on assays validated against tandem mass spectrometry (grade B, weak recommendation).
- We suggest a TAC starting dose of 0.1–0.2 mg/kg/day (max starting 5 mg/day) given orally twice daily (grade B, weak recommendation).
- We suggest titrating the TAC dose aiming for trough levels between 4 and 8 ng/ml. We also suggest titration intervals of at least 3 days (grade B, weak recommendation).
- We suggest monitoring CsA/TAC trough levels at least weekly until target levels are reached, and then every 1–3 months together with serum creatinine as a safety parameter (grade D, weak recommendation) (Table 2).
- We recommend reducing CNI dosage or its withdrawal if eGFR decreases below 30 ml/min/1.73 m^2^ (grade X, strong).

## Evidence and rationale

Although monitoring of CsA at 2 h post dose (C2) is the most accurate single time point for assessment for therapeutic level [67], C2 target levels in SRNS patients are not widely established or practical for routine use. Instead, whole blood trough measurements by tandem mass spectrometry are recommended. These assays give lower readings than immunoassays, which were previously used. The ranges of CsA levels reported in RCTs vary widely [48, 49, 52, 53, 56, 58]. More recent studies use lower levels of CsA (troughs of 80–150 ng/ml), with an initial starting dose of 5–6 mg/kg/day [53–55, 57]. Since, even low CsA trough levels can be associated with long-term nephrotoxicity in children with NS, we suggest targeting CsA trough levels of 80–120 ng/ml, although higher levels may be more effective but should be analyzed together with serum creatinine as a safety parameter. High dosages of CsA (C2 levels > 600 ng/ml) showed increased risk for CsA nephrotoxicity especially when given in combination with ACEis/ARBs in children with SDNS [68]. Levels should be monitored weekly until steady state and then every 1–3 months.

### Duration of CNI treatment

- We suggest a minimum treatment period of 6 months to determine the response to CNIs (grade B, weak recommendation).
- We recommend that CNIs should be stopped if partial remission is not achieved at 6 months (grade B, moderate recommendation).
- If complete remission is achieved, CNI dosages should be reduced to the lowest dosage required to maintain remission. We also suggest considering discontinuation of CNIs after 12–24 months in these patients to reduce the risk of nephrotoxicity (grade C, weak recommendation). In these patients, switching to MMF can be considered to minimize nephrotoxicity and maintain remission (vide infra).
- If relapses occur after CNI discontinuation, we suggest restarting patients on CNIs for a trial together with 4 weeks of high-dose oral PDN. Alternately MMF or may be considered (grade C, weak recommendation).
- If partial remission is achieved, we suggest continuing CNI at the same dosage for a minimum of 12 months (grade C, weak recommendation).

## Evidence and rationale

Due to the risk of nephrotoxicity and side effects related to long-term immunosuppression (see Table 4), CNIs should be discontinued after 6 months if at least a partial remission is not achieved. If complete remission is achieved, we suggest considering discontinuation of CNIs after 12–24 months. See “Treatment of relapse” for relapse therapy.

### Mycophenolate mofetil

- If immunosuppression is considered in a child with SRNS and an eGFR < 30 ml/min/1.73 m^2^, we suggest that MMF rather than CNIs be used due to the risk for nephrotoxicity with CNI (grade C, weak recommendation).
- We suggest considering the use of MMF to maintain remission in children with SRNS in remission following CNI if they develop a steroid sensitive relapse (grade C, weak recommendation).
- In patients with SRNS who have attained full remission on CNI therapy for at least 12 months, we suggest considering conversion to MMF as an alternative immunosuppressive agent rather than continuing CNIs (grade C, weak recommendation).

## Evidence and rationale

If immunosuppression is considered in a child with SRNS and an eGFR < 30 ml/min/1.73 m^2^, then MMF may be used to avoid CNI nephrotoxicity. CsA was not superior in achieving remission when compared with MMF in combination with dexamethasone (45% vs. 33%) [52]. When a child with SRNS achieves remission following CNI therapy but subsequently has a steroid sensitive relapse, then based on RCTs evaluating MMF in relapsing SSNS [22, 69, 70], MMF may be used to maintain remission. The rationale to switch to a CNI-free immunosuppressive protocol is to avoid long-term CNI toxicity. A CNI-to-MMF conversion protocol was applied successfully in children with SRNS after a mean of 1.7 years of CNI therapy with regular drug monitoring [71]. However, in an RCT conversion from TAC to MMF was shown to be inferior to maintain remission in patients achieving remission by TAC [57]. We suggest a MMF starting dose of 1200 mg/m^2^ per day, and performing therapeutic drug monitoring in SRNS patients aiming for a mycophenolic acid exposure (AUC) > 50 μg × h/ml based on the results in SSNS patients [22].

### Repeat kidney biopsy

- If there is an unexplained drop in eGFR or increase in proteinuria during follow-up, we suggest considering a repeat kidney biopsy for assessment of CNI nephrotoxicity (grade C, weak recommendation).
- We suggest considering a renal biopsy in those patients who have prolonged CNI exposure (> 2 years) or when being restarted on CNI-treatment for a second course (grade C, weak recommendation).

## Evidence and rationale

An unexplained eGFR decrease or an increase in proteinuria may be due to disease progression or drug toxicity, especially in patients on long-term CNI treatment. The latter is supported in the presence of arteriolar hyalinization and smooth muscle vacuolization, ischemic glomerular collapse, juxtaglomerular apparatus hyperplasia, (striped) interstitial fibrosis and tubular atrophy on light microscopy, and mitochondrial damage on transmission electron microscopy [72].

### Co-intervention with glucocorticoids

- We do not recommend prolonged (> 6 months) routine PDN treatment in conjunction with CNI and RAASi (grade C, moderate recommendation)
- We suggest tapering PDN after CNI initiation as follows: 40 mg/m^2^ QOD for 4 weeks, 30 mg/m^2^ QOD for 4 weeks, 20 mg/m^2^ QOD for 4 weeks, 10 mg/m^2^ QOD for 8 weeks, and discontinuing thereafter (grade D, weak recommendation).

## Evidence and rationale

Prednisone was used as a co-intervention in several RCTs [52, 53, 55, 56]. The dose and duration of PDN ranged from 1 mg/kg/day for 6 months QOD to 0.3 mg/kg/day for 6 months. There is no evidence that prolonged treatment with oral PDN is beneficial in SRNS patients but may cause steroid toxicity; therefore, we suggest a gradual reduction of PDN using the regimen suggested above, [73, 74]. PDN may be weaned off more quickly especially in patients presenting with glucocorticoid toxicity. However, this does not apply to a proportion of SRNS patients who achieve complete remission with CNI and subsequently behave as SDNS patients. These patients may be treated accordingly with additional low-dose alternate day oral PDN.

### Second-line approaches

- Patients with SRNS who fail to achieve at least partial remission with CNIs (and who do not have genetic or syndromic disease) should be approached for participation in a clinical trial evaluating novel potential therapies for SRNS (ungraded).
- If a clinical trial is not available, the use of rituximab may be considered (grade C, weak recommendation).
- We suggest administering two rituximab infusions at a dose of 375 mg/m^2^ per infusion in order to reduce the CD19 cell count below 5 per microliter or 1% (usually 1–2 infusions within 2 weeks) (grade C, weak recommendation).
- Rituximab should not be given in the presence of tuberculosis, hepatitis B, or JC virus infections. In case of clinical suspicion and endemic background, patients should undergo screening by a chest X-ray, tuberculosis skin or blood test, HBs-Ag serology in case of elevated liver enzymes, and spinal fluid examination in case of neurological symptoms suggesting JC virus infection before commencing rituximab (grade X, strong recommendation).
- In rituximab-resistant or rituximab-intolerant patients, the use of ofatumumab and extracorporeal blood purification therapies such as plasma exchange, immunoadsorption, or lipid apheresis may be considered (grade C, weak recommendation)

## Evidence and rationale

Observational studies showed complete remissions in ~ 30% of patients treated with rituximab as a rescue therapy for multidrug-resistant SRNS [75–85]. However, rituximab was not superior compared with treatment protocols including plasma exchange and immunoadsorption [85]. In most studies, patients with multidrug-resistant SRNS received rituximab at a dose of 375 mg/m^2^ per infusion, and 1–2 infusions over 2 weeks were usually sufficient to reduce the CD19 cell count below 5 per microliter or 1% of lymphocyte count. In patients achieving partial or complete remission, first-AM proteinuria and B cell counts should be monitored and a second course of rituximab be administered when proteinuria increases substantially after B cell reconstitution (CD19 cell count > 5 per microliter or 1% of lymphocyte count). Contraindications for rituximab include hepatitis B, tuberculosis, or JC virus infections. Cotrimoxazole prophylaxis and completion of age appropriate vaccination schedule is recommended (see sections Antibiotic prophylaxis and Vaccination). Serum levels of IgG should be monitored after rituximab treatment as they were found to be low in ~ 30% of patients [86, 87].

In several small pediatric studies, rituximab-resistant or rituximab-intolerant cases as well as patients without rituximab pretreatment reportedly underwent complete remission with the alternative CD20 cell-depleting agent ofatumumab [88–90]. Ofatumumab was administered in two studies at an initial dose of 300 mg/1.73 m^2^ (max 300 mg) followed by 5 weekly doses of 2000 mg/1.73 m^2^ (max 2000 mg) [89, 90] and in a single case report 750 mg/1.73 m^2^ [88].

Various pharmacological and extracorporeal therapies have been applied experimentally in patients with multidrug resistant SRNS. Partial or complete remission has been observed in individual case reports or in a few cases within small series of patients receiving plasmapheresis, plasma exchange, immunoadsorption, lipid apheresis [91–93], the B7-1 inhibitor abatacept [94–96], and oral galactose [97–99]. Inclusion of patients in clinical trials testing these and other novel therapies is strongly encouraged (ongoing studies are listed here: https://kidneyhealthgateway.com/trials-research/).

### Withdrawing immunosuppression in non-responsive patients

- We recommend that screening for all known podocytopathy genes be offered to enable decisions on further immunosuppression (grade X, strong recommendation).
- We recommend counseling patients and parents regarding the high risk of progression to ESKD in patients with hereditary forms and/or multidrug-resistant SRNS (grade X, strong recommendation).
- We recommend discontinuing ineffective immunosuppressive therapies, and continuing non-immunosuppressive management, including RAASi and other supportive measures (grade X, strong recommendation).
- In patients with non-genetic disease, we suggest exploring available options for novel therapies being assessed in clinical trials (grade X, strong recommendation).
- In patients with inherited defects who have achieved *partial* or *complete* remission with immunosuppression, we suggest the following:
  - The genetic variant(s) should be reviewed to confirm whether it is indeed pathogenic or likely pathogenic (grade A, strong recommendation).
  - A decision to continue or discontinue immunosuppression should follow parental counseling regarding the anticipated benefits of remission (symptomatic relief; potentially lower risk of disease progression) versus the potential risks (therapy related toxicity; infections) and cost of therapy (grade A, strong recommendation).

## Evidence and rationale

Non-response is associated with rapid progression to ESKD [11, 100, 101]. In patients with genetic forms of SRNS, low rates of complete (2.7–3.0%) or partial response (10.8–16%) to immunosuppression were reported [9, 11, 101, 102]. Patients with genetic forms of SRNS progress to ESKD more often than those without inherited defects (71–74% vs. 4–29%) and show shorter median renal survival (45–48 months vs. 58–205 months) [11, 100–102]. Given the likelihood of harm versus benefit, we suggest withdrawing immunosuppression in non-responsive monogenic SRNS patients. In those with defects in the COQ pathway, COQ10 supplementation may be considered [103–105]. While the probability of response to experimental therapies is low in patients with multi-drug-resistant disease, therapy could be contemplated after direct counseling of patients and parents about the low likelihood of benefit, and the possibility of toxicity with such therapies [89, 91, 106–108].

### Additional measures to reduce symptoms and control edema

#### Salt

- We suggest that excessive salt intake should be avoided in children with SRNS (Table S11) (grade C, weak recommendation).
- When available, a dietician should provide advice to patients and families on suitable low-salt foods and on the high-salt foods to avoid (grade D, weak recommendation).

#### Fluid

- We do not recommend routine fluid restriction in SRNS patients (grade C, weak recommendation).
- We suggest a balanced fluid intake taking into account the urine output, volume status, and serum sodium (grade C, weak recommendation).

#### Diuretics

- We suggest considering treatment with loop diuretics (e.g., furosemide) in patients with severe edema. In patients with refractory edema, the addition of metolazone, thiazides, or potassium sparing diuretics may also be considered (grade C, moderate recommendation).
- Diuretics should not be given to patients with signs of intravascular volume depletion including prolonged capillary refill time, tachycardia, hypotension, and oliguria due to the risk of thrombosis and AKI (grade X, strong recommendation).

#### Albumin infusions

- We suggest treating patients with refractory edema (pericardial/pleural effusions, anasarca, genital edema) and/or symptomatic hypovolemia or with prerenal crisis (oliguria due to intravascular volume depletion) with human albumin infusions (grade C, moderate recommendation).
- We suggest a starting dose of 20–25% albumin of 0.5–1 g/kg body weight given intravenously over a period of 4–8 h, and adding furosemide (1–2 mg/kg given i.v.) in the middle and/or at the end of the infusion (grade C, weak recommendation).
- Children receiving albumin infusions should initially be monitored with blood pressure and heart rate measurements every 30 min, and the infusion slowed or ceased if they develop any symptoms suggestive of vascular overload (grade X, strong recommendation).

#### Protein

- There is insufficient evidence to recommend an increased protein intake in SRNS patients (ungraded).

## Evidence and rationale

Severe edema in NS may be associated with either intravascular volume contraction (“underfilled patient”) or volume expansion (“overfilled patient”) [109]. Therefore, all measures should be tailored according to the degree of edema and intravascular volume status. Clinical indicators for intravascular volume depletion are peripheral vasoconstriction (prolonged capillary refill time), tachycardia, hypotension, and oliguria, in the setting of urinary sodium retention (fractional sodium excretion (FeNa) < 0.2%). In contrast, hypertension and a FeNa > 0.2% would suggest an overfilled patient [110–112].

### Salt

According to the “underfilled” and “overfilled” hypotheses edema formation in idiopathic NS is thought to be associated with salt retention and/or diminished excretion of salt [109]. Consequently, a strict dietary restriction of sodium intake < 2 mEq/kg/day (< 35 mg/kg/day) was proposed for children with NS [110, 113, 114]. However, such a strong sodium restriction seems not to be feasible in children and may not be required in many patients. Therefore, instead of an upper limit, we recommend avoiding excessive salt intake depending on the degree of edema (Supplementary Table S11). This usually requires dietary advice—from a dietician.

### Fluid

General restriction of fluids to two-thirds of maintenance have been suggested in children with NS [7111]. However, this may put patients, who already have intravascular volume depletion (“underfilled patient”) despite the presence of concomitant edema, at risk for symptomatic hypovolemia. Therefore, we do not recommend routine fluid restriction in SRNS patients. Instead, we suggest a balanced fluid intake taking into account urine output, volume status, and serum sodium (low serum sodium suggests fluid overload). Patients should avoid salty foods, as they increase thirst (Supplementary Table S11).

### Diuretics

Treatment of severe edema in children with NS with diuretics alone is safe and effective in the presence of volume expansion (“overfilled patient”) [113], whereas aggressive treatment with diuretics carries the risk of intravascular hypovolemia, AKI, and thrombosis in “underfilled patients” [115]. Therefore, we suggest considering treatment with diuretics (preferably loop diuretics) in patients with severe edema only when intravascular volume depletion has been excluded based on the abovementioned clinical indicators. Combination therapy with metolazone, thiazides, or potassium sparing diuretics including the epithelial sodium channel blocker amiloride and the aldosterone antagonist spironolactone can enhance diuresis as compared with a loop diuretic alone and should be considered in patients with refractory edema [116]. However, patients need to be carefully monitored to avoid severe hypokalemia or hyperkalemia, volume depletion and alkalosis [117–120]. Since furosemide has a short duration of action (*t*_1/2_ 6 h) and great variation in oral bioavailability (10–100%), it should be administered at least twice daily as oral doses or intravenously if the diuretic response is poor [121, 122].

### Albumin infusions

Albumin infusions in combination with loop diuretics increase diuresis via improved oncotic pressure and renal hemodynamics in patients with severe refractory edema, especially when used in “underfilled patients” [123–125]. However, they work only transiently [126], and are associated with allergic reactions [127], respiratory failure, and congestive heart failure, especially when given too rapidly, used in “overfilled patients,” and patients with oliguria [126]. Therefore, careful assessment of the patient’s intravascular volume status and urine output is mandatory [110]. Dosages up to 1 g/kg given as 20–25% albumin over a period of at least 4 h are thought to be safe [128]. We suggest restricting albumin infusions to patients with severe edema (pericardial/pleural effusions, anasarca, genital edema), symptomatic hypovolemia, or with prerenal crisis. Adding furosemide in the middle and/or at the end of the infusion enhances the diuretic response.

### Protein intake

Hypoalbuminemia is associated with several complications in SRNS including thrombosis and risk of AKI [115], but there is no evidence that increased oral protein intake improves serum albumin levels or patient outcome [129].

### Recommendations for lifestyle

- We recommend supporting physical activity and a healthy nutrition in children with SRNS and adapting to the patient’s ability and stage of CKD. We recommend advising against smoking (grade C, moderate recommendation)

## Evidence and rationale

Patients with SRNS have an increased risk for cardiovascular disease [130] and impaired bone health [131, 132]. Therefore, regular physical activity; refraining from smoking, vaping, or substance use; and a healthy nutrition as in the general population are recommended. Nutrition should be guided by a dietician allowing adequate energy intake and avoiding high salt (vide supra) or phosphorus intake and adapted to the child’s age or height age in short children, and stage of CKD [133, 134]. Eating home-prepared meals using fresh ingredients instead of canned, frozen, or packaged meals are preferred (Table S11), since the latter has a much higher content of salt and inorganic phosphorus which is up to 100% absorbed by the intestine [134].

### Monitoring and management of complications of NS and side effects of medications

#### Monitoring of complications

- We recommend monitoring for complications of the persistent NS and medication side effects (see Table 4**)** (grade B, moderate recommendation).

## Evidence and rationale

Disease-related complications include infections, hypogammaglobulinemia, hyperlipidemia, hypertension, hypothyroidism, venous thromboembolism, vitamin D deficiency, growth failure, obesity, malnutrition, AKI, and CKD. Potential side effects of medications are shown in Table 4, and primary outcome parameters for use in registries/studies are shown in Supplementary Table S9.

### Interventions—prevention and treatment

#### Hypogammaglobulinemia—immunoglobulin substitution

- We suggest that immunoglobulin substitution be considered in cases of low serum IgG levels AND recurrent and/or severe infections (grade D, weak recommendation).

## Evidence and rationale

Arguments against routine IgG substitution in patients with low IgG include (a) the rapid urinary loss following infusion, (b) commercial immunoglobulin preparations contain low IgG titers against bacteria mainly responsible for the septic episodes (staphylococci, streptococci, gram-negative bacteria) [135], and (c) the high costs. We thus suggest considering prophylactic IgG substitution as in other cases of secondary hypogammaglobulinemia in patients presenting with recurrent and/or severe infections [136].

### Antibiotic prophylaxis

- We do not recommend routine antibiotic prophylaxis in children with SRNS (grade C, weak recommendation).
- We suggest antibiotic prophylaxis with cotrimoxazole in patients treated with rituximab for a period of 3 up-to 6 months depending on B cell recovery and immunosuppressive co-medication (grade C, weak recommendation).

## Evidence and rationale

Although 60% of NS-associated deaths are attributable to infection [137], there is no evidence to recommend antibiotic prophylaxis in children with SRNS [138–142]. Thirty to 50% of infections were due to pneumococcal infection, with the rest are due to gram-negative bacilli principally *E. coli* [2, 114, 137, 143–146]. It was estimated that 110 children would need to be treated for 1 year to prevent 1 pneumococcal peritonitis [147]. Given the high mortality of *Pneumocystis jirovecii* pneumonia, we suggest to administer cotrimoxazole in patients on rituximab therapy for a period of 3 up to 6 months depending on B cell recovery and use of additional immunosuppressive co-medications [75]. Prophylactic cotrimoxazole dosing is recommend with 5–10 mg TMP/kg/day or 150 mg TMP/m^2^/day in infants (at least 4 weeks of age) and children, given as single daily dose or in two divided doses every 12 h 3 times weekly (on consecutive or alternate days) with a maximum TMP dose of 320 mg/day [148]. The oral dosing in adolescents is 80 to 160 mg TMP daily or 160 mg TMP 3 times per week [149]. Whereas a 50% dose reduction of cotrimoxazole is required when eGFR < 30 ml/m^2^/min, use of cotrimoxazole is not recommend with eGFR < 15 ml/m^2^/min. In those cases, an alternative option may be prophylactic aerosolized pentamidin, but there is insufficient evidence in the efficacy.

### Vaccination

- We recommend reviewing the child’s vaccination status at disease onset and completing all vaccinations without delay, especially for encapsulated bacteria (pneumococcal, meningococcal, *Haemophilus influenzae*) and, if possible, varicella-zoster virus (grade A, strong recommendation).
- We suggest administering inactivated influenza vaccine annually (grade A, strong recommendation).
- We recommend following national vaccination guidelines for the administration of inactive and live attenuated vaccines in immunocompromised patients (grade A, strong recommendation)
- Live vaccines should not be given in SRNS patients on daily immunosuppressive medication including CNIs, MMF, and PDN (grade X, strong recommendation)**.**

### Prevention of varicella infection

- We recommend treating susceptible patients (i.e., those not or inadequately immunized to varicella and exposed to chickenpox) with varicella-zoster immunoglobulin (VZIG) (grade A, strong recommendation).
- If VZIG is not available, we suggest treatment with oral acyclovir (10 mg/kg QID for 7 days) within 7–10 days of exposure (grade C, moderate recommendation).
- We recommend varicella vaccine should be administered to unimmunized patients in remission and not on immunosuppressive medications (grade A, strong recommendation).

## Evidence and rationale

Varicella infection can be life threatening in children with SRNS. The Food and Drug Administration (FDA) approved VZIG for reducing chickenpox symptoms in susceptible patients, i.e., those not immunized and having no history of chickenpox [150]. VZIG should be given as soon as possible up to 10 days post-exposure [151–154]. Unfortunately, VZIG is not readily available in most countries. Two small studies in 52 immuno-competent children and one in 8 children with renal disease on corticosteroids suggest that administration of acyclovir reduces the risk of chickenpox when given within 7–10 days after exposure and continued for 7 days [155–157]. Once in remission and not on immunosuppressive medications, varicella vaccine should be administered in unimmunized patients and family members.

### Prevention of thrombosis

- We recommend mobilizing patients as much as possible and not placing central venous lines, except for a specific and transient need (grade X, strong recommendation).
- There is insufficient evidence to recommend routine prophylactic anticoagulation for children with SRNS and with no prior history or risk of thrombosis (ungraded).
- We suggest preventive anticoagulation with low molecular weight heparin or oral anticoagulants in those patients with a previous history of venous thromboembolic events, and consideration of treatment for those with additional risk factors (indwelling central venous lines, known hereditary thrombophilic predisposition, acute illnesses with hospitalization, infection or risk of dehydration) (grade C, weak recommendation).
- We suggest thrombophilic screening in SRNS patients with additional risk factors including central venous lines, persistent nephrotic range proteinuria, and positive family history for thrombophilic predisposition (Table 2) (grade C, weak recommendation).

## Evidence and rationale

A 3% incidence of thromboembolic events has been reported in children with NS (summarized in [158–160]. Risk factors include disease-related hypercoagulability, underlying thrombophilic predisposition, infections [161], and treatment, e.g., central venous lines. In all SRNS children, baseline coagulation tests (stated in Table 2) should be performed during the initial workup. We suggest extending the thrombophilic screening in patients with high-risk (previous thrombotic events or known hereditary thrombotic predisposition) by screening for hereditary deficiencies of anticoagulant proteins (e.g., protein C, protein S, and antithrombin) and single-nucleotide polymorphisms in the prothrombin (factor II G20210A) and factor V genes (factor V G1691A). We also suggest considering preventive anticoagulation with low-molecular weight heparin in SRNS patients at high thrombotic risk for the short term, with vitamin K antagonists for the long term [158].

### Treatment of hyper- or dyslipidemia

- We suggest considering age-dependent lipid-lowering treatment in children with persistent multidrug-resistant NS and persistently high fasting LDL-cholesterol (> 130 mg/dl; > 3.4 mmol/l) (grade C, weak recommendation).

## Evidence and rationale

Prolonged hyper-/dyslipidemia complicates persistent NS and is a risk factor for cardiovascular morbidity, but data to guide antihyperlipidemic treatment in children are scarce [162–166]. Uncontrolled studies in children with NS showed a reduction in LDL and total cholesterol levels by 30–40% using a combination of statins and lifestyle changes, but a RCT in children with SRNS showed no significant reduction in lipid levels [167–169]. Given the high cardiovascular morbidity associated with dyslipidemia, we suggest considering lipid-lowering treatment in children with SRNS and persistent LDL-cholesterol levels > 130 mg/dl (3.4 mmol/l), starting with lifestyle changes, including dietary modifications, enhanced physical activity and weight control [166]. There is no evidence to recommend the use of lipid-lowering statins in NS. Some experts suggest considering statins when fasting LDL-cholesterol is persistently > 160 mg/dl (4.1 mmol/l) [140, 170] or earlier (> 130 mg/dl (3.4 mmol/l)), in case of additional cardiovascular risk factors [166].

### Calcium, magnesium, and vitamin D supplementations

- We suggest administering oral calcium if hypocalcemia exists based on ionized and/or albumin-corrected calcium levels (grade C, weak recommendation).
- We suggest supplementing with cholecalciferol or ergocalciferol if 25-OH-vitamin D levels are low (< 30 ng/mL) (grade C, moderate recommendation).
- We suggest administering oral magnesium in case of symptomatic hypomagnesemia (grade D, weak recommendation).

## Evidence and rationale

Children with SRNS have urinary losses of vitamin-D binding protein and 25-dihydroxyvitamin D and may develop vitamin D deficiency leading to hypocalcemia, hyperparathyroidism, and impaired bone mineralization [171]. Vitamin D supplementation in these patients is effective [172–174], and recommended as in other CKD patients [175]. CNI treatment may cause hypomagnesemia causing leg cramps. Administering oral magnesium will avoid symptomatic hypomagnesemic episodes.

### Thyroid hormone replacement

- We recommend substituting levothyroxine (T4) in case of hypothyroidism (grade A, strong recommendation).

## Evidence and rationale

Hypothyroidism in children with SRNS is a result of urinary loss of thyroxine-binding proteins [176, 177]. Therefore, TSH and free T4 levels should be regularly monitored in patients with persistently high-grade proteinuria (Table 2) [178, 179]. For those children with TSH levels > 10 mU/l and low free T4, we recommend treating with levothyroxine (T4) [180]. In asymptomatic children with TSH elevations of 4.5–10 mU/l and normal free T4, thyroid function can be monitored periodically and the indication for treatment re-evaluated [177, 180, 181].

### Treatment of hypertension and CKD-associated complications

- We recommend treatment of hypertension and CKD-associated complications such as anemia, metabolic acidosis, and hyperparathyroidism, according to current guidelines (grade A, strong recommendation).

## Evidence and rationale

Children with SRNS have a significantly increased risk for cardiovascular disease [130, 132]. As in any child with CKD, high blood pressure (> 95th age-sex and height specific percentile) should be treated aiming for blood pressure values < 75th percentile in children without proteinuria, and < 50th percentile in children with proteinuria [182, 183]. Other CKD-associated complications should be treated according to current guidelines [133, 175, 184].

### Diagnosis, prevention, and treatment of relapsing SRNS in native kidneys

#### Prevention of relapse

- No clinical or histological parameters at initial clinical presentation are available to predict relapsing SRNS (ungraded).

## Evidence and rationale

It is unknown to what degree medications should be tapered or discontinued once remission is achieved [53, 71]. Relapse occurred in up to 70% of those responding to CNI therapy after discontinuation at 6 or 12 months. We recommend continuing immunosuppressive therapy with CNI or MMF after remission over a period of at least 1 year [6, 57]. Gradual reduction of CNI/MMF instead of abrupt stopping may prevent an early relapse [50].

### Treatment of relapse

#### Relapse on CNI treatment

- We recommend adherence to CNIs be monitored using serum trough levels according to the monitoring schedule shown in Table 2 (grade C, moderate recommendation)
- We suggest administering oral PDN 60 mg/m^2^ daily until remission is achieved or for a maximum period of 4 weeks, with subsequent taper when remission is achieved (grade C, weak recommendation).
- In case of no response, frequent relapses, or side effects of medications, we recommend following the refractory SRNS protocol (see “Second-line approaches”) (ungraded).

#### Relapse post withdrawal of immunosuppressive treatment

- We suggest giving oral PDN (60 mg/m^2^ daily) until remission is achieved *or* for a maximum period of 4 weeks, with subsequent taper when remission is achieved. Alternatively, we suggest restarting the immunosuppressive agent, which was able to prevent, relapses (grade D, weak recommendation).
- In cases of no complete response within 4 weeks, frequent relapses or side effects of medications, we recommend following the refractory SRNS protocol (see “Second-line approaches”) (ungraded).

## Evidence and rationale

### Relapsing SRNS and role of steroids

Several studies have shown the effectiveness of PDN in relapsing SRNS at 2 mg/kg/day to induce remission [52, 53] with a change to QOD PDN, followed by tapering until the end of month 6 [185, 186]. Intravenous MPDN was also effective in inducing remission in relapsing patients [71, 74, 187]. Re-starting non-glucocorticoid medications which were effective in the particular patient is also reasonable.

### Management of children with ESKD

#### Dialyzed patients

- We recommend that urine protein excretion should be measured prior to transplantation in patients with residual native kidney function to facilitate accurate post-transplant surveillance for recurrence (grade A, strong recommendation).
- We recommend that the anticipated recurrence risk after renal transplantation should be discussed with the family in renal replacement therapy planning (grade A, strong recommendation).
- If transplant will occur before resolution of NS in the setting of ESKD, we suggest considering medical or surgical nephrectomies prior to transplantation (grade D, weak recommendation).

## Evidence and rationale

Preparation for transplantation ideally requires the resolution of NS to minimize the risk for venous thromboembolism and improve the accuracy of monitoring for post-transplant recurrence. If adequate resolution of proteinuria does not occur after the initiation of dialysis based on 24-h urine protein, we suggest considering medical or surgical nephrectomies. However, the benefits of residual kidney function and urine output in facilitating dialysis should also be considered.

### Selection of transplant recipients

- We recommend that genetic testing be performed before transplantation to inform SRNS recurrence risk (grade B, moderate recommendation).
- We recommend kidney transplant be offered to children with ESKD secondary to SRNS regardless of genetic or non-genetic cause of SRNS (grade B, moderate recommendation).
- We suggest that the risks and benefits of a repeat transplant in a patient with a history of SRNS recurrence should be discussed within the transplant team and with the patient and family in planning for a repeat-transplant (grade A, strong recommendation).

## Evidence and rationale

Factors associated with post-transplant recurrence of SRNS are non-genetic vs. monogenic forms of SRNS (recurrence 24% vs. 0% in Brazilian cohort [188] and 50% vs. 7% in European cohort [101]; initial steroid resistance vs. sensitivity (OR 30, 95% CI 6.6–135.9) [189]; time to ESKD < 48 vs. > 48 months (OR 11.7, 95% CI 1.53–89.1) and glomerulosclerosis percentage < 55% at renal biopsy (OR 16, 95% CI 1.45–1.76) [190]. Children with a history of SRNS recurrence in a prior transplant have a > 80% likelihood of recurrence in a subsequent transplant [188]. Complete and partial remission has been reported in 63% and 8% of patients with recurrent NS post-transplant with a 10 years allograft survival of 50% [191, 192].

### Selection of transplant donors

- We recommend candidate living-related allograft donors undergo genetic testing as part of evaluation in the setting of genetic SRNS if available (grade X, strong recommendation).
- We recommend a donor candidate with a pathogenic or likely pathogenic variant in a dominant gene, with or without symptoms, be excluded as a potential donor (grade X, strong recommendation).
- A heterozygous carrier of a recessive SRNS genetic variant may be considered a potential donor, after genetic counseling (except for carriers of pathological variations in *COL4A5*, *COL4A3*, and *COL4A4*) (grade C, weak recommendation).
- An asymptomatic carrier of a variant of unknown significance may be considered as a transplant donor following extensive evaluation and counseling where other organ donation options are not available (grade C, weak recommendation).
- We recommend that the expected risk of recurrence and premature allograft failure be included in the consideration of donor candidacy (grade A, strong recommendation).

## Evidence and rationale

Living-related kidney donation in the context of genetic kidney diseases should follow detailed donor evaluation, careful review of pattern of disease inheritance, and genetic counseling and testing [193, 194]. While a family history of a genetic kidney disease with an autosomal recessive (AR) mode of inheritance is not considered a contraindication for living kidney donation, long-term follow-up data are lacking [193]. In cases where SRNS follows an autosomal dominant (AD) mode of inheritance, donation from living related donors from the side of family with affected members is discouraged. If it remains uncertain whether the donor candidate has a genetic kidney disease and whether the disease can cause CKD, donation should proceed only after informing the donor candidate of the risks of donation if the disease manifests later in life [193, 194].

Hemizygous carriers (mothers and sisters) of *COL4A5* defects should be dissuaded from kidney donation, since they are known to develop ESKD [195]. Similar advice should be given to donors with pathogenic heterozygous defects in other COL4A (*COL4A3* and *COL4A4*). Further, the risk to donors carrying heterozygous *NPHS2* mutations may be modified by variants such as R229Q, which are considered to have a dominant-negative variant that might theoretically pose risk to the donor [196, 197]. Tests including evaluation of proteinuria and hematuria done as part of the donor assessment should be interpreted with special consideration in the setting of familial SRNS. If genetic evaluation of the potential donor is normal but the family history is positive, donation should proceed only after a full informed consent.

### Accepting living donor for kidney transplantation in view of risk of recurrence

- Either living related or deceased donors are encouraged for patients with non-genetic SRNS receiving their first allograft (grade B, moderate recommendation).

## Evidence and rationale

Similar proportions of patients with recurrence were observed among living versus deceased allografts (10–50% vs. 3–45%), but allograft survival was superior in living donor allografts with recurrent FSGS compared with deceased donor allografts [198–200].

### Withholding transplantation from patients who have previously recurred

- We recommend, discouraging living related donation for recipients who have had disease recurrence in the first transplant (grade B, moderate recommendation).
- Deceased donor transplant may be offered to potential recipients with a history of prior allograft loss to recurrence of NS, particularly if dialysis is difficult to sustain, or associated with life-threatening events, serious infections, poor growth, and/or low quality of life (grade C, weak recommendation).

## Evidence and rationale

Transplantation should not be delayed in SRNS patients, since this does not reduce the recurrence risk [200–204]. Recurrence in first allograft indicates a 60–80% risk of recurrence in subsequent allografts [199, 203, 204]. Strategies used to manage recurrent disease (high-dose CNI, intravenous MPDN, rituximab; and extracorporeal therapies) induced remission in ~ 60% of cases [200, 205, 206]. While a few reports suggest that early diagnosis and aggressive therapy of recurrent disease may result in outcomes comparable to those in allografts without recurrence [207, 208], outcomes after recurrence are usually poor for patients who do not respond to interventions [209–214]. Therefore, repeat transplants from living donors are discouraged in the setting of prior SRNS disease recurrence and deceased donor transplantation, rather than dialysis, is considered ethically appropriate.

### Prevention of recurrence after renal transplantation

- There is insufficient evidence to recommend intervention strategies for the prevention of recurrence in children undergoing a first kidney transplant (ungraded).
- We suggest prophylactic plasmapheresis or immunoadsorption or lipid apheresis and perioperative rituximab for use in children with a history of allograft loss due to NS recurrence in a prior transplant (grade C, weak recommendation).

## Evidence and rationale

There are no proven preventative strategies to reduce the likelihood of recurrence in SRNS patients undergoing the first renal transplantation. Preventative strategies for SRNS recurrence in primary, non-genetic SRNS with a history of SRNS recurrence within 1 year of transplant were shown to be effective in case reports and small series including 8 patients. They include prophylactic plasmapheresis three times weekly for 2 weeks, beginning 1 week prior to living donor transplant or within 1 day of deceased donor transplant with 1.5 plasma volume exchanges and rituximab peri-operatively or immediately after transplant with/or without a second dose post-transplant day 7 [93, 215–217].

### Transplant recurrence (as defined in Table 1)

- We recommend surveillance for recurrence beginning on the day of kidney transplantation by monitoring UPCR, continued daily throughout the initial transplant hospitalization, and then continued periodically (e.g., weekly for 4 weeks, monthly for 1 year, then quarterly thereafter) (grade C, moderate recommendation).
  - We suggest in a previously anuric patient, post-transplant UPCR ≥ 100 mg/mmol (1 mg/mg) may be indicative of early recurrence, infection, or other diagnoses and requires evaluation (grade C, weak recommendation).
  - We suggest in a patient with prevalent proteinuria at the time of transplant, an increase of UPCR ≥ 100 mg/mmol (1 mg/mg) may be indicative of early recurrence, infection or other diagnoses and requires evaluation (grade C, weak recommendation).
  - We suggest that early onset acute tubular necrosis (ATN) or graft non-function/dysfunction should be regarded as first signs of recurrence (grade C, weak recommendation).
  - We suggest that an allograft biopsy is not required to diagnose rapid recurrence of NS as defined in Table 1, but allograft biopsy is recommended for the exclusion of differential diagnosis in the setting of subnephrotic proteinuria, recurrence after 48 h, or in the setting of delayed graft function (grade B, moderate recommendation).
  - We suggest that a diagnostic evaluation precede adjustments of immunosuppression therapy in the setting of late NS recurrence (> 3 months post-transplant) including assessment of infection, donor-specific antibodies serologies, and histopathology including electron microscopy (grade B, moderate recommendation).

## Evidence and rationale

Nephrotic syndrome may recur as early as within 24 h after transplantation and is indicated by a UPCR ratio ≥ 100 mg/mmol (1 mg/mg) in a previously anuric patient (Table 1). Early onset acute tubular necrosis (ATN) or graft non-function/dysfunction should be considered as a first sign of transplant recurrence [218, 219]. A diagnosis of FSGS recurrence can be inferred on renal biopsy with diffuse foot process effacement in the absence of other histopathological findings, even if the glomerular scar defining FSGS is not present. Late-onset or insidious proteinuria requires a renal biopsy for the exclusion of the differential diagnoses including de novo TMA and antibody-mediated rejection with transplant glomerulopathy, since both can show secondary FSGS [191, 220–222].

### Treatment of recurrence

- We recommend implementing NS recurrence-specific therapy as soon as possible after diagnosis is established (grade X, strong recommendation).
- We suggest applying increasing doses of CNI, intravenous MPDN pulses, and/or plasmapheresis (or immunoadsorption) with or without rituximab (grade C, weak recommendation).
- We suggest initiating RAASi when no complete remission is achieved following recurrence targeted therapy (grade C, weak recommendation).

## Evidence and rationale

Strategies used to manage recurrent disease including high-dose CNI, intravenous MPDN, rituximab, and extracorporeal blood purification induced remission in ~ 60% of transplant recurrence [198, 223]. We suggest in patients, treated with rituximab, to administer a second dose of rituximab (375 mg/m^2^) in the setting of incomplete B cell depletion and/or recurrence of proteinuria.

## Electronic supplementary material

ESM 1 (DOCX 728 kb)
