# Supplementary material for: IPNA clinical practice recommendations for the diagnosis and management of children with steroid-resistant nephrotic syndrome
Source: Pediatr Nephrol. 2020 May 7;35(8):1529–61. doi: 10.1007/s00467-020-04519-1 (PMC7316686; doi:10.1007/s00467-020-04519-1)
Supplement: Supplementary file 1 — (DOCX 728 kb) [file 467_2020_4519_MOESM1_ESM.docx]

**IPNA Clinical Practice Recommendations for the Diagnosis and Management of Children with Steroid-resistant Nephrotic Syndrome**

Agnes Trautmann^1^, Marina Vivarelli^2^, Susan Samuel^3^, Debbie Gipson^4^, Aditi Sinha^5^, Franz Schaefer^1^, Ng Kar Hui^6^, Olivia Boyer^7,8^, Moin A Saleem^9^, Luciana Feltran^10^, Janina Müller-Deile^11^, Jan Ulrich Becker^12^, Francisco Cano^13^, Hong Xu^14^, Yam Ngo Lim^15^, William Smoyer^16^, Ifeoma Anochie^17^, Koichi Nakanishi^18^, Elisabeth Hodson^19^, Dieter Haffner^20,21^ on behalf of the International Pediatric Nephrology Association

^1^ Division of Pediatric Nephrology, Center for Pediatrics and Adolescent Medicine, Heidelberg, Germany.

^2^ Division of Nephrology and Dialysis, Department of Pediatric Subspecialties, Bambino Gesù Pediatric Hospital and Research Center, Rome, Italy

^3^ Section of Pediatric Nephrology, Department of Pediatrics, Alberta Children’s Hospital, University of Calgary, Calgary, Canada

^4^ Division of Nephrology, University of Michigan, Michigan, US

^5^ Division of Nephrology, Department of Pediatrics, All India Institute of Medical Sciences, New Delhi, India.

^6^ Department of Paediatrics, Yong Loo Lin School of Medicine, National University of Singapore, Singapore

^7^ Laboratory of Hereditary Kidney Diseases, Imagine Institute, INSERM U1163, Paris Descartes University, Paris, France

^8^Department of Pediatric Nephrology, Reference center for idiopathic nephrotic syndrome in children and adults,
 Necker Hospital, APHP, 75015 Paris, France

^9^ Department of Pediatric Nephrology, University of Bristol, Bristol Royal Hospital for Children, Bristol, UK

**^10^** Hospital Samaritano and HRim/UNIFESP, Federal University of São Paulo, São Paulo, Brazil

**^11^** Department of Nephrology, University Hospital Erlangen, Erlangen, Germany

**^12^** Institute of Pathology, University Hospital of Cologne, Cologne, Germany

**^13^** Department of Nephrology; Luis Calvo Mackenna Children’s Hospital, University of Chile

^14^ Department of Nephrology; Children's Hospital of Fudan University, Shanghai, China

^15^ Department of Pediatrics, Prince Court Medical Centre, Kuala Lumpur, Malaysia

^16^ The Research Institute at Nationwide Children's Hospital, The Ohio State University, Columbus, US

^17^ Department of Paediatrics, University of Port Harcourt Teaching Hospital,  Port Harcourt, Rivers State, Nigeria.

^18^ Department of Child Health and Welfare (Pediatrics), Graduate School of Medicine, University of the Ryukyus, Okinawa,  Japan

^19^ Cochrane Kidney and Transplant, Centre for Kidney Research, The Children’s Hospital at Westmead and the Sydney School of Public Health, University of Sydney, Sydney,
 Australia

^20^ Department of Paediatric Kidney, Liver and Metabolic Diseases, Hannover Medical School Children’s Hospital, Hannover

^21^ Center for Rare Diseases, Hannover Medical School Children’s Hospital, Hannover, Germany.

**Supplemental Material:**

**Supplemental Tables:**

**Table S-1:** Area or expertise and responsibilities of core group members

| Name | Area of expertise | Responsibilities |
| --- | --- | --- |
| Anochie, Ifeoma | Pediatric nephrology | Drafting of recommendations and evidence text and grading of recommendations and reviewing the manuscript before submission |
| Becker, Jan Ulrich | Nephropathology | Drafting of recommendations and evidence text and grading of recommendations and reviewing the manuscript before submission |
| Boyer, Olivia | Pediatric nephrology  Renal genetics  Guideline development | Literature search and creation of evidence tables (genetics), drafting of recommendations and evidence text and grading of recommendations and reviewing the manuscript before submission |
| Cano, Francisco | Pediatric nephrology | Drafting of recommendations and evidence text and grading of recommendations and reviewing the manuscript before submission |
| Feltran, Luciana | Pediatric Nephrology | Drafting of recommendations and evidence text and grading of recommendations and reviewing the manuscript before submission |
| Gipson, Debbie | Pediatric nephrology  Guideline development | Drafting of recommendations and evidence text and grading of recommendations and reviewing the manuscript before submission |
| Haffner, Dieter | Pediatric nephrology  Guideline development | Coordination of work groups and the process of generating the manuscript. Drafting of first manuscript and incorporation of suggestions from the core group members, external experts and voting group members into the manuscript. Drafting of recommendations and evidence text and grading of recommendations and reviewing the manuscript before submission |
| Hodson, Elisabeth | Pediatric nephrology Guideline development  Epidemiology | Drafting of recommendations and evidence text and grading of recommendations and reviewing the manuscript before submission |
| Ng, Kar Hui | Pediatric nephrology | Drafting of recommendations and evidence text and grading of recommendations and reviewing the manuscript before submission |
| Müller-Deile, Janina | Adult nephrology ,Transition | Drafting of recommendations and evidence text and grading of recommendations and reviewing the manuscript before submission |
| Nakanishi, Koichi | Pediatric nephrology  Guideline development | Drafting of recommendations and evidence text and grading of recommendations and reviewing the manuscript before submission |
| Lim, Yam Ngo | Pediatric nephrology | Drafting of recommendations and evidence text and grading of recommendations and reviewing the manuscript before submission |
| Saleem, Moin | Pediatric nephrology  Renal genetics | Literature search and creation of evidence tables (genetics). Drafting of recommendations and evidence text and grading of recommendations and reviewing the manuscript before submission |
| Samuel, Susan | Pediatric nephrology, Epidemiology | Drafting of recommendations and evidence text and grading of recommendations and reviewing the manuscript before submission |
| Schaefer, Franz | Pediatric nephrology  Guideline development | Drafting of recommendations and evidence text and grading of recommendations and reviewing the manuscript before submission |
| Sinha, Aditi | Pediatric nephrology | Drafting of recommendations and evidence text and grading of recommendations and reviewing the manuscript before submission |
| Smoyer, William | Pediatric nephrology | Drafting of recommendations and evidence text and grading of recommendations and reviewing the manuscript before submission |
| Trautmann, Agnes | Pediatric nephrology  Epidemiology | Drafting of first manuscript and incorporation of suggestions from the core group members, external experts and voting group members into the manuscript. Literature search and creation of evidence tables, drafting of recommendations and evidence text and grading of recommendations and reviewing the manuscript before submission |
| Vivarelli, Marina | Pediatric nephrology  Guideline development | Drafting of recommendations and evidence text and grading of recommendations and reviewing the manuscript before submission |
| Xu, Hong | Pediatric nephrology | Drafting of recommendations and evidence text and grading of recommendations and reviewing the manuscript before submission |

**Table S2 Randomized Controlled Trials in SRNS – Conservative Treatment**

| **No** | **1^st^ author,**  **year,**  **country of origin**  **[Ref.]** | **Title of Publication** | **Study design** | **Keywords** | **Patients** | **Intervention and comparator** | **Outcomes** |
| --- | --- | --- | --- | --- | --- | --- | --- |
| **Immunosuppressive Therapies** | | | | | | | |
| **Cyclosporine versus Placebo/ Steroids** | | | | | | | |
| 1 | [Lieberman, 1996](https://www.ncbi.nlm.nih.gov/pubmed/8808110) [1]  USA | A randomized double‐blind placebo‐controlled trial of cyclosporine in steroid‐resistant idiopathic focal segmental glomerulosclerosis in children. Journal of the American Society of Nephrology 1996;7(1):56‐63. [MEDLINE: 8808110] | Parallel RCT multicentre | CsA Treatment  Remission status at 6 month  Time to response  Adverse events | N=24/31 analysed  **CsA Group:**  N=12/16 analysed  **Placebo:**  N=12/15 analysed  Age: 6 months-21 years  Mean age: 11.2 ± 4.2 years (CsA group); 11.4 ± 3.9 yrs (placebo)  Sex (M/F): 21/9 | **CsA Group:**  6 mg/kg/d for 6 months, adjusted to target level 300 to 500 ng/ml  **Placebo Group:**  Placebo for 6 months  **Co-interventions:**  Calcium channel blockers for hypertension | Complete remission at 6 months:  CsA: 4/12 (33.3%)  Placebo: 0/12 (0%)  Partial remission at 6 months:  CsA: 8/12 (66.7%)  Placebo: 2/12 (16.7%)  Proteinuria reduction from start to 6 months:  CsA: from 151.7±162.4mg/kg*d to 36.9±42.3mg/kg*d (p<0.05)  Declined by 70.7±19.2% (p<0.05)  Placebo: 166.9±137.1 to 195.4±173.7)  Increased by 11.4±29.0%  Time to response (at least 50% reduction of proteinuria): 4.4 ±1.8wks (CsA Group)  Adverse events  GFR decline:  CsA: From 103.4±36.7 to 82.9±19.1% (p=0.05)  Placebo: 86±31.3 to 75.1±30.6 (p=0.06)  No significant difference in the fractional GFR decline between both groups:  CsA: -15.7±18.4  Placebo: -11.8±19.0% Gingival hyperplasia (2/12 CsA),  worsening of hypertension (2/24, 1 in each group), intercurrent infections (2/24, 1 in each group) |
| 2 | [Ponticelli, 1993a](https://www.ncbi.nlm.nih.gov/pubmed/8315953)  [2] Italy | A randomized trial of cyclosporine in steroid‐resistant idiopathic nephrotic syndrome. Kidney International 1993;43(6):1377‐84. [MEDLINE: 8315953] | Parallel RCT multicentre | CsA Treatment  Remission status at 12 months  Time to response  Adverse events | N= 17/20 children analysed  All initial resistance  **CsA Group**:  N=10/10 analysed  **No Treatment Group**:  N=7/10 analysed  Age: > 2 yrs and < 16 yrs; mea n age ~ 6-8 yrs  Sex (M/F): not reported for children  Histology: MCD (8), FSGS (9) | **CsA Group:**  CsA 6 mg/kg/d for 6 months adjusted to 250 to 600 ng/ml; taper by 25% every 2 months in patients with response  **No treatment group:**  No treatment. „Rescue“ treatment with corticosteroids allowed for progressive kidney failure/ severe nephrotic syndrome  **Co-interventions:**  Not permitted: Nephrotoxic antibiotics, ACEi, NSAIDs, anti-epileptic drugs | Complete remission during 12 months:  CsA: 4/10 (40%), 2/10 (20% with relpases  Partial remission during 12 months:  CsA. 2/10 (20%)  In most cases, the remission occurred within the first month.  Cumulative probabililty of obtaining remission within one year (adults+children):  CsA: 0.65; Control: 0.16 (p>0.05)  Adverse events:  No differences in blood pressure  CsA (main symptoms):  Infections (5 in 3 patients). Gingival hyperplasia (7/10), hypertrichosis (3/10), transient abdominal discomfort (4/10).  Control (main symptoms):  Infections (6 in 3 children)  All symptoms disappeared after the 1^st^ year of observation.  In no case, CsA needed to be stopped or reduced. |
| 3 | [Garin, 1988](https://www.ncbi.nlm.nih.gov/pubmed/3046332)  [3] USA | Garin EH. Cyclosporine therapy for steroid-resistant nephrotic syndrome. A controlled  study. American Journal of Diseases of Children 1988;142(9):985-8. [CRSREF: 3333559; MEDLINE: 3046332] | Cross-over RCTs  single tertiary centre | CsA Treatment  Remission status at 8 weeks | N=8  **CsA Group**:  N=4  **Control Group:**  N=4  Age: 3-18 yrs, median age 12 yrs  Sex: M/F: 6/2  Histology: MCD (4), FSGS (4) | **CsA Group:**  5 mg/kg/d for 8 weeks adjusted to level ≤ 200 ng/ml  **Control/ No treatment group:**  No treatment  **Co-interventions:**  Not reported;  no patient on prednisone during study | Complete remission at 8 weeks:  CsA: 0/4; Control 0/4  Partial remission at 8 weeks:  CsA: 0/4; Control 0/4  Proteinuria at 0 and 8 weeks (UPr/Cr ratio in mg/ng crea):  CsA: 12.5±2.1 (wk0); 11.7±3.1 (wk8); P=0.70  Control: 11.9±2.4 (wk0); 17.3±3.5; P=0.002  No significant changes at 8 weeks when both groups compared (p=0.0286)  Serum albumin (g/l) in both groups:  CsA: 20±2 (wk0); 24±3 (wk8), p=0.09  Control: 20±3 (wk0); 18±3 (wk8); p=0.27  No significant changes at 8 weeks when both groups compared (p=0.082)  Renal function:  CsA was not associated with a change in creatinine clearance (P=0.48).  Controls: Creatinine clearance group decreased with time (p=0.023), oserved first after 6 weeks. |
| **Cyclosporin A versus other immunosuppressive therapies** | | | | | | | |
| 4 | [Plank, 2008](https://www.ncbi.nlm.nih.gov/pubmed/18481113)  [4] Austria and Germany (APN) | Cyclosporin A is superior to cyclophosphamide in children with steroid-resistant  nephrotic syndrome  Pediatr Nephrol. 2008 Sep;23(9):1483-93. doi:  10.1007/s00467-008-0794-1. Epub 2008 May 15. PubMed PMID: 18481113; PubMed  Central PMCID: PMC2730636. | Controlled multicentre randomized open label trial  Parallel RCT | CsA Treatment  CPH Treatment  Remission status at 3 month  Adverse events | n=32  **CSA group:**  n=15  **CPA group:**  n=17  Age: mean age 6.99±5.48 yrs (CsA); 6.84±3.9 yrs (CPA)  Sex: 19/13  Histology: MCD (10), FSGS (21), MesPGN (1) | **CSA group:**  oral CSA 150 mg/m^2^/d in 2 divided doses.  Trough level 80-120 ng/ml for 24 weeks  **CPA group:**  IV pulse therapy of 500 mg/m^2^ in a 4-h infusion. Infusion was repeated after 4, 8,12,16, 24, and 36 weeks (7 doses)  **Co-intervention:**  Tapering dose of alternate day prednisone therapy  Patients on ACE inhibitors (CsA 12/15; CPA 8/17) | Complete or partial remission at 12 weeks: 9/15 (60%) in CSA and 3/17 (17.6%) in CPA (P=0.027)  Complete remission at 12 weeks and maintained at 24 weeks:  2/15 (13.3%) in CSA and 1/17 (5.9%) in CPA (p=0.58)  Partial remission at 12 and 24 weeks:  7/15 (46.7%) in CSA and 2/17 (11.8%) in CPA (P=0.04)  Adverse events:  Number of adverse events per month and patient: CsA 1.22±1.08 (0.29-4.0); CPA 1.61±1.04 (0.33-3.0)  Serious adverse events: CsA 8/15, CPA15/17  Selected adverse events:  Most frequent adverse events in both arms: infections, arterial hypertension, Cushing syndrome.  Patients with CsA developed hypertrichosis and gingiva hyperplasia |
| 5 | FSGS-CT Study, 2011  [5-10]  USA | [Gipson DS](https://www.ncbi.nlm.nih.gov/pubmed/21734640). Clinical trial of focal segmental  glomerulosclerosis in children and young adults. Kidney International 2011;80(8):868-78. [CRSREF: 3333550; MEDLINE:  21734640]  [D'Agati VD](https://www.ncbi.nlm.nih.gov/pubmed/23220425). Association of histologic variants in FSGS clinical trial with presenting features and outcomes. Clinical Journal of The American Society of Nephrology: CJASN 2013;  8(3):399-406. [CRSREF: 3333545; MEDLINE: 23220425]  Ferris M, Patient recruitment into a multicenter randomized  clinical trial for kidney disease: report of the focal segmental glomerulosclerosis clinical trial (FSGS CT). Clinical &  Translational Science 2013;6(1):13-20. [CRSREF: 3333546; MEDLINE: 23399084]  [Gipson DS](https://www.ncbi.nlm.nih.gov/pubmed/21178977). Clinical trials treating focal segmental  glomerulosclerosis should measure patient quality of life. Kidney International 2011;79(6):678-85. [CRSREF: 3333551;  MEDLINE: 21178977]  [Hogg RJ](https://www.ncbi.nlm.nih.gov/pubmed/23143503). Renal function and proteinuria after  successful immunosuppressive therapies in patients with FSGS. Clinical Journal of the American Society of Nephrology:  CJASN 2013;8(2):211-8. [CRSREF: 3333553; MEDLINE: 23143503]  [Kopp JB](https://www.ncbi.nlm.nih.gov/pubmed/25573908). Clinical features and histology of apolipoprotein  L1-associated nephropathy in the FSGS Clinical Trial. Journal of the American Journal of Nephrology 2015;26(6):1443-8.  [CRSREF: 3333554; MEDLINE: 25573908] | Parallel RCT  multicentre study: 66 sites | CsA Treatment  DEXA Pulses  MMF Treatment  Remission status at 12 month  Adverse events | N=138  **DEXA/MMF:**  N=66  **CsA Group:**  N=72  Age:  age < 18 yrs: N=93 (67%)  age >18-40yrs: N=45 (33%)  Sex (M/F): 73/65  Histology: All FSGS | **DEXA/MMF group:**  Oral pulse DEXA: 0.9 mg/kg/d (max. 40 mg) daily on 2 consecutive days at the start of every second week in week 10-26, then every 4 weeks from week 30-50, for a total of 46 doses (over 12 months)  + Oral MMF 25-36 mg(kg/d (max. 2g/d)  **CsA group:**  Oral CsA 5-6 mg/kg/d (max. 2g/d) divided into 2 doses for 12 month  **Co-interventions:**  Prednisone (or prednisolone) 0.3 mg/kg/dose (max. 15 mg) every other day for the first 6 months of treatment period.  Lisinopril (0.36+/- 0.12 (range 0.04-0.56) mg/kg/d) for 18 months  Losartan (1.10 +/- 0.50 (range 0.55-2.69) mg/kg/d) for patients intolerant of ACEi  Additional antihypertensive therapies were not restricted by study protocol | Evaluation at week 26, 52, 78  **Primary outcomes:**  Complete remission at 52 weeks  DEXA/MMF: 6/66 (9%)  CsA: 14/72 (19%)  (p=0.11)  Partial remission at 52 weeks  DEXA/MMF: 22/66 (33%)  CsA: 33/66 (46%)  No remission at 52 weeks  DEXA/MMF: 38/66 (58%)  CsA: 25/72 (35%)  Treatment failure (kidney failure or death) during 78 week study period:  DEXA/MMF: 7/66 (11%)  CsA: 8/72 (11%)  **Secondary outcomes:**  Persistence of partial remission for 26 weeks following cessation of treatment (at 78 weeks):  DEXA/MMF:  sustained partial remission 17/22 (77%), relapses 4/22 (18%), unknown 1/22 (5%)  CsA: sustained partial remission 16/33 (48%), relapses 11/33 (33%), , unknown 6/33 (18%)  Comparing DEXA/MMF vs. CsA for maintaining at least partial remission at week 76: OR 1.21 (95% CI 0.56-2.66)  **Adverse events**  Number was similar in both arms.  DEXA was discontinued in 5/66 patients due to hyperglycemia (2, 1 required insulin therapy), fever and not re-started (2), pre-defined stop point (1).  MMF was discontinued in 1/66 patient due to gastrointestinal toxicity (week 20)  CsA was discontinued in 4/72 patients due to gout (1), hirsutism (1), severe dyslipidemia (1), patients choice (1). |
| 6 | Bhaumik 2002  India | Comparison of pulse methyl prednisolone vs cyclosporin based therapy in steroid  resistant focal segmental glomerulosclerosis **[abstract].** Indian Journal of Nephrology 2002;12(4):190. [CENTRAL:  CN-00460392; CRSREF: 3333594]  Abstract only | Parallel group RCT | CsA Treatment  MP Pulses | N=25  Not differentiated between children/ adults  **CsA Group:**  N=13  **MP Group:**  N=12  Age: 3-49 yrs | **CsA Group:**  CsA 1-4 mg/kg/day months  + oral prednisolone (10-40 mg/day) for at least 6  **Methylprednisolone (MP) Group:**  Methylprednisolone (250-750 mg IV) daily for 7 days and then weekly for at least 12 weeks  **Co-interventions:**  Blood pressure control  Dietary protein 0.8-1.0 g/kg/day  ACE inhibitors  Lipid lowering agents | Complete remission  CsA: 5/13 (38.4%)  MP Pulse: 2/12 (16.7%)  Decline in proteinuria + Stable creatinine  CsA 6/13 (46%)  IV MP 4/12 (33%)  Progression to ESKD (overall)  CsA: 2/13 (15%)  IV MP: 6/12 (50%)  Progressing to ESKD in 3 years:  CsA: 1/13 (8%)  MP: 4/12 (33%)  Rate of decline in GFR was significantly lower in CsA Group:  CsA: 0.4±0.16 ml/min/month  MP: 0.9±0.29 ml/minute/month  Hospitalisation for therapy related complications:  CsA: 0/13  MP: 3/12 (25%) |
| **Calcineurin-Inhibition: CsA vs. Tacrolimus** | | | | | | | |
| 7 | [Choudhry, 2009](https://www.ncbi.nlm.nih.gov/pubmed/19268410)  [11] India | Efficacy and safety of tacrolimus versus cyclosporine in  children with steroid-resistant nephrotic syndrome: a randomized controlled trial. American Journal of Kidney Diseases 2009;  53(5):760-9. [CRSREF: 3333540; MEDLINE: 19268410] | Parallel RCT  Single centre | CsA Treatment  Tac Treatment  Remission status at 6 and 12 month.  Relapses  Adverse events | N=41  Early/late resistance: 23/18  **TAC Group**  N=21 (M/F 14/7)  **CsA Group**  n=20 (M/F 11/9)  Age: mean age 75 (95% CI 53-97) months (TAC); 63 (95% CI 43-82) months (CsA)  Sex (M/F): 25/16  Histology: MCD (17), FSGS (17), MesPGN (7) | **Tacrolimus (TAC) Group:**  TAC 0.1-0.2 mg/kg/d in 2 divided doses for 12 months  Trough levels 5-8 ng/ml  **CsA Group:**  5-6 mg/kg/d in 2 divided doses for 12 months  Trough levels 100-150 ng/ml  **Co-interventions:**  Alternate day prednisone (1 mg/kg/d for 6 months, 0.5 mg/kg/day for 6 months)  Enalapril (0.3 mg/kg/d)  Atorvastatin  Calcium and vitamin D supplements | Response at 6 months:  Complete remission:  TAC: 9 (42.8%); CSA: 10 (50%)  Partial remission:  TAC: 9 (42.8%); CSA 6 (30%)  Complete or partial remission:  TAC 18 (85.7%); CsA 16 (80%)  Treatment failure:  TAC: 3 (14.3%); CSA 4 (20%), please see also below at 12 months  Response at 12 months:  Complete remission:  TAC 10 (47.6%); CSA 11 (55%)  Partial remission:  TAC 8 (38.1%), CSA 4 (20%)  Complete or partial remission:  TAC: 18 (85.7%), CSA 15 (75%)  Treatment failure:  TAC 3 (14.3%) incl. 1 with persistent nephrotoxicity at 8 weeks and 1 died due to pneumonia/sepsis at 8 months, CSA 5 (25%) incl. 2 with persistent nephrotoxicity at 12 weeks/8 months and 1 with secondary CSA-resistance at 7 months  The likelihood of remission (complete or partial) was similar in TAC/CsA group:  6 months: RR, 1.07; 95% CI, 0.81 to 1.41  12 months: RR, 1.14; 95% CI, 0.84 tp 1.55  Frequency of relapses:  Relapses:  TAC 2/18, 3 relapses  CsA 8/16, 13 relapses  Risk of relapses was higher in CsA Group:  CsA vs. Tac: RR 4,5 (95% CI, 1.1 to 10.2; p=0.01)  Mean trough levels at time of relapse:  TAC: 5.2 (range 2.2-8.2) ng/ml  CsA: 107.4 (range, 67.7 to 166 ng/ml)  Similar to those without relapses  Adverse events:  RENAL:  Nephrotoxocity (persistent/ reversible):  TAC: 2(4.7%)/8 (33.3%)  CSA 2 (10%)/ 10 (50%)  Worsening of hypertension:  TAC: 2 (9.5%)  CSA: 2 (10%)  -> No new-onset hypertension  NEUROLOGICAL:  Headache: TAC 1 (4.7%); CSA: 0  Paresthesia: TAC 1 (4.7%); CSA: 0  COSMETIC:  Hypertrichosis:  TAC: 0; CSA: 19 (95%)* (p>0.001)  Gingival hyperplasia:  TAC: 1 (4.7%); CsA 12 (60%)* (p>0.001)  Acne, skin infection:  TAC 2 (9.5%); CsA 5 (25%)  Diarrhea:  TAC 6 (28.6%); CsA 1 (5%) (p=0.04)  Severe infection (sepsis, pneumonia):  TAC 1 (4.7%); CsA 1 (5%) |
| 8 | Valverde 2012  Mexico | Efficacy of prednisone-tacrolimus  vs. prednisone-cyclosporine in steroid-resistant nephrotic syndrome [abstract]. Pediatric Nephrology  2010;25(9):1804. [EMBASE: 70438149]  Abstract only | Parallel RCT  Single-center | CsA treatment  Tac Treatment | N=17  CSA+Pred.: N=10  TAC+Pred.: N=7  Age: not provided  Sex: not provided  Histology: not provided | CSA+Pred.:  for 12 months; doses not provided  TAC+Pred.:  for 12 months: doses not provided | Complete remission:  CsA: 4/10 (40%)  Tac: 1/7 (14.3%)  Partial remission  CsA: 6/10 (60%)  Tac: 6/7 (85.7%)  Time to achieve remission:  CsA: 24±16 weeks  Tac:16±7 weeks  (no significant difference)  Hypertension:  CsA: 8/10 (80%),  Tac: 1/7 (14.3%)*, p<0.05 |
| **Tacrolimus versus other immunosuppressive therapies** | | | | | | | |
| 9 | [Gulati, 2012](https://www.ncbi.nlm.nih.gov/pubmed/22763815)  [12] India | Treatment with tacrolimus and prednisolone is  preferable to intravenous cyclophosphamide as the initial therapy for children with steroid-resistant nephrotic syndrome.  Kidney International 2012;82(10):1130-5. [CRSREF: 3333562; MEDLINE: 22763815] | Parallel RCT Multicentre study; 5 units | Tac Treatment  CPH treatment  Response status at 6 month and 12 month  Adverse events | N=131 (analysed 124)  Initial/ late resistance: 81/50  **TAC Group:** N=66 (63 analysed)  **CPH Group:** N=65 (61 analysed)  Age: 2-16 yrs  Sex (M/F): 86/45  Histology: MCD (78), FSGS (43), MesPGN (10) | **TAC Group:**  0.1 to 0.15 mg/kg/d for 12 months, adjusted to a level of 5-7 ng/ml or lower levels if patient in remission  **CPH Group:**  IV CPH 500 mg/m2 once a month for 6 months  **Co-interventions:**  Prednisolone: 1.5 mg/kg on alternate days for 2 weeks then tapered by 0.35 mg/kg every 2 weeks to 0.5 mg/kg  Enalapril  Calcium supplements | **Primary outcomes (6 month):**  Complete or partial remission at 6 months  TAC: 82.5% (52/63), CPH 45.9% (28/61) (P<0.001)  Complete remission at 6 months:  TAC: 52.4% (33/63), CPH: 14.8% (9/61) (p<0.001).  Partial remission at 6 months:  TAC: 30.1% (19/63), CPH: 31.1% (19/61) (p<0.001).  Non-response at 6 months/treatment failure at 6 months:  TAC 17.4% (11/63), CPH 54,1 % (33/61) (p<0.001).  Probability of complete or partial remission:  Tac vs. CPH ( log-rank p<0.001; HR 2.64; 95% CI 1.67-4.19 (p<0.001))  Mean time to remission:  TAC 3.5±1.7 months: CPH 4.5±1.7 months  Likelihood of remission:  MCD vs. FSGS/MesPGN: HR 1.74; 95% CI 1.08-2.8)  **Secondary outcomes (12 months) – analysed in n=80 patients**  Sustained remission at 12 months  TAC: 73.1% (38/52), CPH: 42,9% (12/28) (p=0.002)).  Steroid-sensitive course:  TAC 2/52; CPH 2/28  Non-nephrotic proteinuria with serum albumin > 2.5 g/dl: TAC 21.2% (11/52); CPH 39.3% (11/28) (p=0.08)  Resistant nephrotic syndrome:  TAC: 0/52; CPH 3/28  Adverse effects  eGFR:  Decline in eGFR <50ml/min*1.73m^2^:  TAC 1/63, CPH 2/62  Decline of eGFR: TAC 9; CPH -1.5 ml/min*1.73m^2^ (differences were not significant from baseline (p=0.24) or between groups (p=0.64), |
| 10 | [Sinha, 2017](https://www.ncbi.nlm.nih.gov/pubmed/28318625)  [13] India | Mycophenolate mofetil is inferior to tacrolimus in  sustaining remission in children with idiopathic steroid-resistant nephrotic syndrome. Kidney International 2017;  92(1):248-57. [MEDLINE: 28318625] | Parallel RCT multicentre | Tac Treatment  MMF treatment  Maintenance, not induction therapy  Remission status at 12 months  Relapses | N=60  All achieved complete/partial remission with 6 months treatment with TAC  Early/late resistance: 28/32  **TAC Group**: N=31  **MMF Group:** N=29  Age: 1-18 yrs  Sex: 44/16  Histology: MCD (34), FSGS (26) | All initial treatment with TAC for 6 months, achieved complete or partial remission after therapy with TAC  **TAC Group:**  TAC 0.15 mg/kg/d aiming for trough levels 4 to 8 ng/ml  **MMF Group:**  MMF 0.75 to 1 g/m^2^/d  + TAC tapered and discontinued within two weeks of randomisation  **Co-interventions:**  Prednisolone an alternate days (tapered)  Enalapril | **Primary outcome:**  Favourable Outcome at 12 months  TAC: 28/31 (90%)  MMF: 13/29 (45%)  p=0.0002  Sustained complete remission  TAC: 16/29; MMF: 11/29 CR with infrequent SS Relapses:  TAC: 1/31; MMF: 1/29  Sustained partial remission:  TAC: 1; MMF: 1  PR with infrequent SS Relapses: TAC: 4; MMF:0  **Secondary outcomes:**  Treatment Failure at 12 months:  TAC: 3/31 (10%); MMF: 16/29 (55%)  p=0.0002  Recurrence of steroid resistance:  TAC: 0; MMF: 6 (20.6%)  Frequent steroid sens. relapses  TAC: 3 (9.7%); MMF: 10 (34.5%)  The proportion of patients with treatment failure were significantly higher for patients receiving MMF than TAC at 6 months (0% vs. 31%) and 1 year (9.7% vs. 52%) (log rank P=0.0001).  No. of relapses by end of study:  TAC: mean 0.46 ± 0.76; median 0 (0-2)  MMF: mean 0.58 ±0.96; median 1 (0-3)  Steroid-sensitive/-resistant relapses:  TAC: 0.8 ±1.0 (0 (0-2))/ 0  MMF: 1.1 ±1.3 (1 (0-2))/ 0.2 ±0.4 (0 (0-2))  Occurrence of relapse:  TAC: 45.2 (29.2, 62.2)%  MMF: 58.6 (40.7, 74.5)%  Incidence of relapses (per person-yr)  TAC: 0.8 (0.5, 1.2);  MMF: 1.8 (1.3, 2.5)  p=0.001  Cumulative prednisolone received:  TAC: 0.3 ± 0.2 mg/kg/d  MMF: 0.5 ± 0.4 mg/kg/d  p=0.024  eGFR (ml/min*1.73 m^2^), median (IQR):  TAC: 98.7 (82.3-121.8)  MMF: 100.1 (87.4-142.2) |
| **Cyclophosphamide – oral and IV** | | | | | | | |
| 11 | [ISKDC 1974](https://www.ncbi.nlm.nih.gov/pubmed/4137139),  [14] Europe, USA, Mexico, Hong Kong, Japan | Prospective, controlled trial of cyclophosphamide therapy in children with the nephrotic syndrome. Report of the International Study of Kidney Disease in Children. Lancet 1974;2(7878):423‐7. [MEDLINE: 4137139] | parallel RCT multicentre | Oral CPH treatment  Remission status at 3 month  Time to response | N=31  **CPA-Predn. Group:**  N=18  **Prednisone Group:**  N=13  Age: not reported  Sex: not reported  Histology: MCD (14), FSGS (10), MesPGN (2), diffuse proliferative GN (1); unknown (2) | **CPH-Prednisone Group:**  Oral CPH 5 mg/kg/d till WCC < 5000 then 1-3 mg/kg/d  + intermittent prednisone for 90 days  **Prednisone group:**  Intermittent prednisone for 90 days  **Co-interventions:**  not reported | Complete remission at 90 days:  CPH-Pred.: 56% (10/18), Pred.: 40% (6/15)  Time to response:  CPH-Pred: 38.4 (range 6-80) days  Pred.: 95.5 (61-129) days  P<0.05 |
| 12 | [Tarsish, ISKDC 1996](https://www.ncbi.nlm.nih.gov/pubmed/8897562), [15]  Europe, USA, Canada | Cyclophosphamide does not benefit patients with focal segmental glomerulosclerosis. A report of the International Study of Kidney Disease in Children. Pediatric Nephrology 1996;10(5):590‐3. [MEDLINE: 8897562] | Parallel RCT multicentre | Oral CPH Treatment  Remission status at 12 month  Death  Adverse events | N=113 (53/60) (analysed/randomised)  CPA-prednisone Group: N=32/35  Prednisone Group:  N=21/25  Age: range not reported, mean age: 8.6 ±0.85 yrs (CPH-Pred.), 7.4 ± 0.75 years (Predn.)  Sex: not reported  Histology: All FSGS | **CPH-Prednisone Group:**  Oral CPH 2.5 mg/kg/d for 90 days  + alternate day predn. 40 mg/m2 for 12 months  **Prednisone Group:**  Alternate day prednisone for 12 months  **Co-interventions**: not reported | Complete remission:  CPH-Pred.: 25% (8/32), Pred.: 28.6% (6/21)  Partial remission  CPH-Pred.: 25% (8/32), Pred.: 28.6% (6/21)  Treatment failure/ No response:  CPH-Pred. 57% (20/35); Pred. 42.9% (9/21)  Death: 9,4% (5/53)  CPH-Predn. 3/32 (9.4%); Pred. 2/21 (9,5%)  Causes of death: Sepsis (2), Cardiorespiratory arrest (1), unknown (2)  Adverse events/ Side effects:  Hypertensive seizures (2, 1 per each group), hemorrhagic cystitis (1 in CPH group) |
| 13 | [Elhence, 1994](https://www.ncbi.nlm.nih.gov/pubmed/8142205)  [16] India | Intravenous pulse cyclophosphamide - a new regime for steroidresistant  minimal change nephrotic syndrome. Pediatric Nephrology 1994;8(1):1-3. [CRSREF: 3333542; MEDLINE:  8142205] | Parallel RCT  single tertiary centre | Oral CPH and IV CPH treatment  Remission status at 6 months  Time to relapse  Adverse events | N=13  Initial/ Late resistance: 5/8  **IV CPH**  N=7  **Oral CPH:**  N=4/6 analysed  Age: 3-16 yrs (IV group); 9-14.5 yrs (Oral group)  Sex (M/F): 11/2  Histology: All MCD | **IV CPH Group:**  IV CPH: 500 mg/m2/month for 6 months (cumulative dose: 90 mg/kg) + Prednisone 60 mg/m2/d for 4 weeks; 40 mg/m2 a.d. for 4 weeks and taper  **Oral CPH Group:**  Oral CPH: 2.5 mg/kg/d for 8 weeks (cumulative dose 150 mg/kg)  + Prednisone 60 mg/m2/d 4 weeks; 40 mg/m2 a.d. for 4 weeks and taper  **Co-interventions:**  Not reported | **Primary Outcomes:**  Remission at 6 months  IV CPH 7/7 (100%; Oral CPH 1/4 (25%)  Mean proteinuria-free days:  IV CPH: 274.3±44.6 days  Oral CPH: 165±165 days  **Secondary Outcomes:**  Adverse events/ Side effects:  Vomiting: IV CPH: 4/7; Oral CPH: 0/4  Infection: IV CPH 0/7; Oral CPH: 1/4  Alopecia: IV CPH 0/7; Oral CPH 2/4 |
| 14 | [Mantan, 2008](https://www.ncbi.nlm.nih.gov/pubmed/18566839)  [17] India | Efficacy of intravenous pulse cyclophosphamide treatment versus combination of intravenous dexamethasone and oral cyclophosphamide treatment in steroid‐resistant nephrotic syndrome. Pediatric Nephrology 2008;23(9):1495‐502. [MEDLINE: 18566839] | Parallel RCT Single tertiary centre | IV CPH treatment  Oral CPH Treatment  IV DEXA Pulses  Remission status at 6 and 18 months.  Adverse events | N=49/52 evaluated  Early/late resistance: 18/31  **IV CPH Group:**  N=26/27 evaluated  **Oral CPH+IV DEXA Group:**  N=23/25 evaluated  Age: 1-18 years  Sex (M/F): 35/17)  Histology: MCD (24), FSGS (14), MesPGN (11) | **IV CPH Group:**  IV CPH 500 mg/m^2^ monthly (max. 1g) for 6 doses; dose increased to 750 mg/m^2^ monthly of no response at 3 months; dose delayed if WCC < 4000  + Maintenance therapy was then started with prednisone: 0.5 mg/kg alternate days to 18 months  **Oral CPH+IV DEXA Group:**  Oral CPH 2 mg/kg/d from 3rd to 14th weeks and IV DEXA 5 mg/kg alternate days for 6 doses then every 2 weeks (4 pulses) and then monthly (4 pulses).  + Maintenance therapy was then started with prednisone: 0.5 mg/kg alternate days to 18 months  **Co-interventions:**  Alternate day prednisone (1.5 mg/kg for 1 month; 1.25 mg/kg for 1 month and 1 mg/kg for 4 months)  Enalapril 0.3 mg/kg/d | **Short-term outcome (6 months)**  Complete remission at 6 months:  IV CPH: 14/26 (53.8%)  Oral CPH+IV DEXA: 11/23 (47.8%)  Partial remission at 6 months:  IV CPH: 2/26 (76.9%)  Oral CPH+IV DEXA: 2/23 (8.7%)  No Response or failure to complete treatment:  IV CPH: 10/26 (38.5%)  Oral CPH+IV DEXA: 10/23 (43.5%)  **Long-term outcome (18 months):**  Favorable (maintenance of complete remission or steroid-sensitive relapses):  IV CPH: 14/26 (53.8%)  Oral CPH+IV DEXA: 9/23 (39.1%)  Unfavorable:  IV CPH: 12/26 (46.2%)  Oral CPH+IV DEXA: 14/23 (60.9%)  (p=0.2)  Proteinuria, serum albumin, serum cholesterol, GFR evaluated at 6months.  **Adverse events:**  Cushingoid features:  IV CPH: 15/26 (57.7%)  Oral CPH+IV DEXA: 17/23 (73.9%)  Infections (pneumonia, peritonitis, cellulitis, meningitis; most infections (85.7%) occurred in the first 3 months of treatment)  IV CPH: 6/26 (23%)  Oral CPH+IV DEXA: 8/23 (34.8%)  IV CPH: associated with adverse events:  Vomiting episodes (44.9%), reversible alopecia (26.9%), hemorrhagic cystitis (7.7%), leukopenia.  Oral CPH+IV DEXA associated with adverse events:  Hypertension (43.5%), hypokalemia (y3.5 mmol/L: 30.4%),.  1 patient with steroid encephalopathy |
| 15 | [Shah KM, 2017](https://www.ncbi.nlm.nih.gov/pubmed/?term=Shah+KM.+Indian+J+Nephrol.+2017+Nov-Dec%3A+27(6)%3A+430-434)  [18] India | Shah KM. A Randomized Controlled Trial of Intravenous versus  Oral Cyclophosphamide in Steroid-resistant Nephrotic Syndrome in Children. Indian  J Nephrol. 2017 Nov-Dec;27(6):430-434. | Parallel RCT | Oral versus IV Cyclophosphamide  Remission status | N=50  **Oral CPH:**  N=25  **IV CPH**:  N=25  Age: 1-15 yrs (mean: 5.12±2.9 yrs)  Sex (M/F):31/19  Histology: MCD 29/50 (58%), FSGS 13/50 (26%), MesprolGN 8/50 (16%) | **Oral CPH Group:**  Oral CPH 2 mg/kg/d for 12 weeks  **IV CPH Group:**  IV CPH 500 mg/m^2^ every 4 weeks for 6 months  **Co-Interventions:**  Alternate-day steroids in tapering doses | Complete remission:  Oral CPH: 11/25 (44%)  IV CPH: 13/25 (52%)  Partial remission_  Oral CPH: 2/25 (8%)  IV CPH: 2/25 (8%)  No response:  Oral CPH: 12/25 (48%)  IV CPH: 5/15 (60%)  Time to remission:  Oral CPH: 47.5±26.1 days  IV CPH: 86.1±29.1 days  P=0.002  Mean duration of remission was similar:  Oral CPH: 8.13±8.85 months  IV CPH: 9.15±8.28 months; (p=0.963)  Similar number of patients with sustained remission for up to 1 yr after cessation of treatment:  Oral CPH: 4/25; IV CPH 3/25.  Side effects:  Major side effects (peritonitis, Cellulitis):  Oral CPH: 2/25; IV CPH 5/25  Minor side effects (alopecia, emesis)  Oral CPH: 3/25; IV CPH 4/25  None of these patients required discontinuation of treatment.  No death. No hemorrhagic cystitis. |
| 15a | Ohri A.  India  (Please see Shah KM, 2017, same data) | Randomized controlled trial of oral versus intravenous  cyclophosphamide in idiopathic steroid resistant nephrotic syndrome [abstract]. Pediatric Nephrology 2010;  25(9):1879. [EMBASE: 70438526]  Abstract only | Parallel RCT | Oral versus IV Cyclophosphamide  Remission status | N=35  **Oral CPH:**  N=20  **IV CPH**:  N=15  Age: 1-12 yrs (mean: 5.12±2.9 yrs)  Sex (M/F): 24/11  Histology: MCD 21/35 (60%), FSGS 8/35 (23%), MesprolGN 6/35 (17%) | **Oral CPH Group:**  Oral CPH 2.5 mg/kg/d for 12 weeks  **IV CPH Group:**  IV CPH 750 mg/m^2^ every 4 weeks for 6 months  **Co-Interventions**: not reported | Complete remission:  Oral CPH: 8/20 (40%)  IV CPH:8/15 (53.3%)  Partial remission_  Oral CPH: 5/20 (25%)  IV CPH: 2/15 (13.5%)  No response:  Oral CPH: 5/20 (25%)  IV CPH: 5/15 (33%)  Time to remission:  Oral CPH: 44.6±30 days  IV CPH: 87.5±25 days  P=0.008  Side effects:  Severe infections  Oral CPH: 1/20; IV CPH: 5/15  Transient neutropenia:  Oral CPH: 1/20; IV CPH: 1/15  Alopecia:  Oral CPH: 5/20; IV CPH: 5/15 |
| **Other immunosuppressive therapies** | | | | | | | |
| 16 | [Abramowicz M](https://www.ncbi.nlm.nih.gov/pubmed/4191931), [19] ISKDC 1970  Europe, USA, Japan, Mexico | Controlled trial of azathioprine in children with nephrotic syndrome. A report for the international study of kidney disease in children. Lancet 1970;1(7654):959‐61. [MEDLINE: 4191931] | Parallel RCT multicentre | Azathiaprine  Remission status at 3 month | N=31  **AZA group:** N=16  **Placebo**: N=15  Age: not reported  Sex: not reported  Histology: MCD (5), FSGS (10), MesPGN (15), unknown (3) | **Treatment Azathioprine (AZA) Group:**  60 mg/m2/d  + intermittent prednisone for 90 days  **Placebo Group:**  Placebo  + intermittent prednisone for 90 days  **Co-interventions:**  not reported | Complete remission at 90 days:  AZA: 12.5% (2/16); Placebo 13.3% (2/15)  Partial remission at 90 days  AZA: 12.5% (2/16); Placebo 13.3% (2/15)  No response at 90 days:  AZA: 75% (12/16); Placebo 73% (11/15) |
| 17 | Kleinknecht 1980  [20], France | Irreversible renal failure after indomethacin in steroid‐resistant nephrosis. New England Journal of Medicine 1980;302(12):691. [MEDLINE: 6986554] | Parallel RCT multicentre | Chlorambucil  Indomethacon  Remission status  Progression to ESRD | N=30  Chlorambucil Group:  N=15  Indomethacin Group:  N=15  Age: not reported  Sex: not reported  Histology: MCD (9), FSGS (14), FSGS with mesangial proliferation (6) | **Chlorambucil Group:**  0.2 mg/kg/d for 6 months  **Indomethacin Group:**  3 mg/kg/d for 6 months  **Co-interventions:** not reported | Remission of nephrotic syndrome  Chlorambucil: 4/15 (27%)  Indomethacin: 4/15 (27%)  Persistent nephrotic syndrome:  Chlorambucil: 11/15 (73%)  Indomethacin: 9/15 (60%), 2/15 (13%) GFR decrease and ESKD 3 and 9 months later  Patients with persistent nephrotic syndrome:  10 patients initially treated with Chlorambucil received Indomethacin: 3/10 achieved remission, 3/10 persistent NS, 4/10 renal failure.  8 patients initially treated with Indomethacin received Chlorambucil: 5/8 achieved remission, 3/8 had persistent NS.  Summary of results (Cochrane review 2016):  No significant difference between Chlorambucil and indomethacin in the number who achieved remission and who progressed to ESKD. |
| 18 | [Wu B, 2015](https://www.ncbi.nlm.nih.gov/pubmed/25312783)  [21] China | Triple immunosuppressive therapy in steroid‐resistant nephrotic syndrome children with tacrolimus resistance or tacrolimus sensitivity but frequently relapsing. Nephrology 2015;20(1):18‐24. [MEDLINE: 25312783] | Parallel RCT Single tertiary centre | MMF  Oral CPH  Leflunomide  Remission status at 6 and 12 months | N=18/22 analysed  TAC sensitive but frequently relapsing (10)  Tac resistant (12)  **MMF group:**  N=5/7 analysed  **CPH group:**  N=6/8 analysed  **LEF group:**  N=7/7 analysed  Age: 1-17 years  Sex (M/F):  Histology: MCD (10), FSGS (5), MesPGN (1), IgM nephropathy (2) | **Triple immunosuppressive therapy:**  **MMF Group:**  MMF 20-30 mg/kg/d, divided into 2 doses daily for 12 months  **CPH Group:**  CPH 8-12 mg/kg/d for 2 days and then repeated at 2-4 week intervals for 3 to 6 months to maximum dose of less than 150 mg/kg  **LEF (leflunomide) group:**  0.5 to 0.6 mg/kg (maximum dose 30 mg) for 2 days and then 0.2 mg/kg/d (maximum dose 15 mg) for 12 months  **Co-interventions:**  TAC and prednisone (duplex immunosuppressive therapy) | **Duplex versus triple therapy:**  **Short-term response within 6 months**  Complete remission:  Duplex: 8/18 (44.4%)  Triple 14/18 (77.8%)  Partial/ No remission:  Duplex: 10/18 (55.6%)  Triple: 4/18 (22.2%)  **Long-term response after 12 months** (in patients with complete remission at 6 months)  Complete remission (no relapse, without frequent relapses):  Duplex: 0/8  Triple: 11/14 (78.6%)  **3 different groups:**  **Short-term response within 6 months:**  Complete remission:  MMF: 3/5 (60%)  CPH: 4/6 (66.7%)  LEF: 7/7 (100%)  Partial/ No remission:  MMF: 2/5 (40%)  CPH: 2/6 (33.3%)  LEF: 0/7  **Long-term response after 12 months** (in patients with complete remission at 6 months)  Complete remission (no relapse, without frequent relapses):  MMF: 3/3  CPH: 3/4  LEF: 5/7 |
| **Antibody therapies** | | | | | | | |
| 19 | [Magnasco, 2012](https://www.ncbi.nlm.nih.gov/pubmed/22581994)  [22] Italy | Rituximab in children with resistant idiopathic nephrotic syndrome. Journal of the American Society of Nephrology 2012;23(6):1117‐24. [MEDLINE: 22581994] | Parallel RCT Multicentre (4 units) | Rituximab  Multi-Drug-Resistant/ Refractory SRNS  Remission status at 3 months  Adverse events  CD20count monitoring | N=31 (all resistant to the combination of CNI+Pred for at least 6 months before randomization)  Early/late resistance: 16/15  RTX Group:  N=16 (early/late restistance: 9/7)  Control Gorup: N=15  (early/late restistance: 7/8)  Age: 1-16 yrs; mean age: 8.5 ± 4.4 years (RTX); 7.3 ± 3.7 yrs (Control)  Sex (M/F): 19/12  Histology: MCD (7), FSGS (19), no biopsy (4), inadequate material (1) | **Rituximab (RTX) Group:**  2 doses IV RTX 375 mg/m^2^; 1st dose at randomisation and 2nd dose 2 weeks later  **Control Group:**  No additional intervention other than standard therapy  **Co-interventions:**  Prednisolone, tapered off by 0.3 mg/kg/week if proteinuria < 1g/d/m^2^  CNI (at pre-enrolment doses): TAC (16), CsA (15) for RTX group, after 2 weeks from prednisone withdrawal, CNI was decreased by 50% and ceased after 2 weeks additional weeks  ARB or ACEi in 25 participants | Proteinuria at baseline and 3 months ( performed at a central lab, g/day per m^2^):  All:  RTX: 2.7 (1.7, 4,2) to 1.8 (0.9, 3.4)  Control: 2.4 (1.7-3.5) to 1.4 (0.9-2.8)  Early-resistance:  RTX: 2.9 (1.2, 6.6) to 2.7 (1.6, 7.8)  Control: 6 (1.5, 8.8) to 3.9 (1.2, 7.1)  Late resistance:  RTX: 1.3 (0.8, 6.3) to 0.8 (0.1, 1.7)  Control: 2.4 (0.8, 4.8) to 0.8 (0.1, 4.6)  Complete remission at 3 months:  RTX: 3/16 (18.8%) (all late resistance)  Control Group: 3/15 (20%) (all late resistance)  Primary efficacy measure was the percentage change in daily proteinuria at 3 months:  No significant proteinuria percentage reduction at study end (3 months) between RTX and Control group:  All: -12 (-73 to 110)% (p.077)  Early resistance: -3 (-67 to 179)% (p=0.05)  Late resistance: -48 (-79 to 93) (p=0.4)  CD20 counts were reduced to <1% at the first month in all rituximab-treated patients.  After 3 months, CD20 count was still undetectable in 15/16 patients (94%),  Kidney function, plasma proteins, cell blood counts, and cholesterol obtained monthly  Adverse events:  Acute:  Bronchospasma+hypotension (1/16)  Abdominal pain (4/16)  Skin rash (3/16)  Mild dyspnea (2/16)  Delayed (after 18 months follow-up):  No important side effects reported |
| 20 | FONT 1 study 2009  [23-26]  USA | [Joy MS](https://www.ncbi.nlm.nih.gov/pubmed/19073787), Phase I trial of rosiglitazone in FSGS: I.  Report of the FONT Study Group. Clinical Journal of the American Society of Nephrology: CJASN 2009;  4(1):39-47. [MEDLINE: 19073787]  [Joy MS](https://www.ncbi.nlm.nih.gov/pubmed/19932542), Phase 1 trial of adalimumab in Focal Segmental Glomerulosclerosis (FSGS): II. Report of the FONT (Novel Therapies for Resistant FSGS) study group.  American Journal of Kidney Diseases 2010;55(1):50-60. [MEDLINE: 19932542]  [Ternant D](https://www.ncbi.nlm.nih.gov/pubmed/26521258), A possible influence of age on absorption and  elimination of adalimumab in focal segmental glomerulosclerosis (FSGS). European Journal of Clinical  Pharmacology 2016;72(2):253-5. [MEDLINE: 26521258]  [Peyser A](https://www.ncbi.nlm.nih.gov/pubmed/?term=peyser+a+and+adalimumab), Follow-up of phase I trial of adalimumab and  rosiglitazone in FSGS: III. Report of the FONT study group. BMC Nephrol. 2010 Jan  29;11:2. | Parallel RCT (Phase 1)  Multicentre | Adalimumab  Rosiglitazone  Multi-Drug-Resistant/ Refractory SRNS  Proteinuria reduction at 4 month  Renal function at 4 month  Adverse events | N=19/21 analysed  Adults and children  **Adalimumab**  N=9/10 completed 16 week study  Age: 16.8±9.0 yrs (range 6-36 yrs)  **Rosiglitazone**  N=10/11 completed 16 week study.  Age: 15.4±6.2 yrs /range 4-28 yrs)  Sex (M/F): 10/11  Histology: all FSGS (biopsy proven) | **Adalimumab group:**  Adalimumab 24 mg/m2 s.c. (max. 40 mg/dose) on alternate weeks for 16 weeks for a maximum of 40 mg  **Rosiglitazone group:**  Rosiglitazone 3 mg/m^2^/ twice daily orally for 16 weeks (max.daily dose of 8 mg)  **Co-interventions:**  ACEi or ARB with unchanged dosage, diuretics, low dose prednisolone, lipid lowering agents  Patients were off all immunosuppressive medication (except low dose prednisolone) for at least 4 wk before enrolment. | Patients were evaluated at baseline and week 1,2,4,8,12 and 16  **Adalimumab group:**  4/9 patients achieved reduction in proteinuria of 50%.  No significant changes for urinary prot/cre ratio, serum creatinine, eGFR, serum albumin, liver function tests, ANA titer, hematocrit.  1/9 patient progressed to CKDV during observation period.  1 mild adverse effect probably related to adalimumab (injection site reaction).  **Rosiglitazone group:**  2/10 had 40% reduction in proteinuria  No significant changes in urinary prot/crea ratio, serum creatinine, eGFR, serum albumin and blood glucose.  4/10 patients progressed to CKDV during observation period.  3 mild adverse effects (hives, penile swelling, dizziness) possibly related to rosiglitazone  Comparison of eGFR between both groups;  No significant difference in eGFR (P=0.74)  In a similar percentage: stable eGFR during observation period in both groups (rosiglitazone group 5/9 (56%), adalimumab group 5/7 (71%)). |
| 21 | FONT II study 2011 [27, 28]  USA | [Trachtman H.](https://www.ncbi.nlm.nih.gov/pubmed/21310077) Novel therapies for resistant focal  segmental glomerulosclerosis (FONT) phase II clinical trial: study design. BMC Nephrology 2011;12(1):8. [MEDLINE:  21310077]  [Trachtman H](https://www.ncbi.nlm.nih.gov/pubmed/26198842). Efficacy of galactose and  adalimumab in patients with resistant focal segmental glomerulosclerosis: report of the FONT clinical trial group.  BMC Nephrology 2015;16:111. [MEDLINE: 26198842] | parallel RCT (Phase 1)  multicentre | Adalimumab  Galactose Group  Multi-Drug-Resistant/ Refractory SRNS  RAAS  Lipid lowering therapy  Reduction of proteinuria  Adverse events | N=21  Refractory FSGS  Adults and children  Age: 1-51 yrs  Histology: all FSGS (genetic forms included).  **Adalimumab group:**  N= 6/7 completed the 26 week study  **Galactose group:** N=7/7 completed the 26 week study  **Control group: 6/7** 6 completed the 26 week study  Age/Gender  All participants: 14.7 yrs (IQR 13.0, 20.8 years); 9M/12F  Adults and children aged 1-51 years | Patients off all immunosuppressive agents (except low dose prednisolone) for at least 4 weeks.  **Adalimumab group**  Adalimumab 24 mg/m2 (max. 40 mg/dose) s.c. on alternate weeks  for 26 weeks  **Galactose group**  Galactose 0.2 g/kg per dose orally twice a day, dissolved in 15–30 ml of water and ingested  15–30 min before breakfast and dinner for 26 weeks. The max. single dose was 15 g.  **Control group**  Conservative therapy as set out below only for 26 weeks.  **Co-interventions** in all participants:  Lisinopril (max. dose 10 mg for participants < 40 kg; 20 mg for participant ≥ 40 kg)  Losartan (max. dose 25 mg for participants < 40 kg; 50 mg for ≥  40 kg)  Atorvastatin (max. dose 10 mg for participants < 40 kg; 20 mg for ≥ 40 kg) | **Primary outcome:**  ≥50% reduction in proteinuria at 6 month:  Adalimumab: 0/6  Galactose: 3/7  Control: 2/6  Preservation of eGFR at 6 month:  Adalimumab: 4/6  Galactose: 4/7  Control: 5/6  Preservation of eGFR and ≥50% reduction in proteinuria at 6month:  Adalimumab: 0/6  Galactose: 2/7  Control: 2/6  **Adverse events:**  Most of the subjects had at least one adverse event (mainly: edema, infection, fatigue, pain, musculoskeletal, gastrointestinal etc.).  No discontinuation of drug necessary |
| **RAAS-Inhibition** | | | | | | | |
| 22 | [Bagga, 2004](https://www.ncbi.nlm.nih.gov/pubmed/14648339)  [29] India | Enalapril dosage in steroid-resistant nephrotic syndrome. Pediatric  Nephrology 2004;19(1):45-50. [CRSREF: 3333534; MEDLINE: 14648339] | Cross-over RCT  single centre | Enalapril  RAAS  Proteinuria reduction at 8 weeks  Biochemical parameters at 8 weeks  Blood pressure at 8 weeks  Adverse events | n=25/29 analysed  initial SRNS n=15  late SRNS n=10  **High dose**  n=14/15 analysed  **Low dose**  n=11/14 analysed  Age: 1-16 yrs  Sex (M/F): 18/7  Histology: MCD (4), FSGS (9), MCGN (7), MesPGN (7) | **High dose enalapril-group:**  0.6 mg/kg/d for 8 weeks in 2 doses  washout for 2 weeks, then low-dose for 8 weeks  **Low dose enalapril-group:**  0.2 mg/kg/d for 8 weeks in 2 doses,  washout for 2 weeks, then high-dose for 8 weeks  **Co-intervention:**  Alternate day prednisone, furosemide  No treatment with NSAIDs, calcium channel blockers and beta-adrenergic blockers | Urine albumin/Cr ratios after 8 weeks (baseline/8 weeks)  High-dose: 5.2 (2.1-10.5)/ 2.5 (0.8-3.3)* p<0.001  Low-dose: 3.9 (1.9-11.6)/ 2.3 (0.8-5.2)  Urine albumin/Cr reduction after 8 weeks (Median (95% CI):  High-dose: 62.9 (40.6-71.6)%  Low-dose: 34.8 (-7.9-76.6)%  P<0.05  17/25 (68%) patients had a significant reduction in proteinuria.  The median Ua/Uc percentage reduction following 16 weeks of treatment was 48.1 (20.9-78.7)% and 46.2 (33.4-79.1)% in patients with and without hypertension.  Combined data from both groups showed a median Ua/Uc reduction percentage:  High-dose: 52 (15.4-70.4)%*  Low-dose: 33 (-10.3 to 72.4)%*  P<0.05  Blood biochemistry at 8 weeks:  Albumin (g/dl)  High-dose: 3.5 (2.0-4.6)*  Low-dose: 4.4 (3.9-5.5)*, p<0.005  Cholesterol (mg/dl):  High-dose: 264 (241-303)  Low-dose: 208 (168-337)  Creatinin (mg/dl)  High-dose: 0.6 (0.4-0.8)  Low-dose: 0.5 (0.4-0.9)  Potassium (mmol/l):  High-dose: 5.0 (4.3-6.6)  Low-dose: 4.5 (4.0-6.0)  Blood pressure (mmHg) (baseline/8 weeks)  High-dose: 110/70 -> 106/65.5  Low-dose: 120/80 -> 114.3/74.4  The dose of enalapril did not influence the percentage reduction in syst./diayst. BP.  Adverse events:  Dry cough in 3/35 patients (8.6%), subsided after stopping treatment |
| 23 | [Yi, 2006](https://www.ncbi.nlm.nih.gov/pubmed/16773409)  [30] China | Effect of fosinopril in children with steroid‐resistant idiopathic nephrotic syndrome. Pediatric Nephrology 2006;21(7):967‐72. [MEDLINE: 16773409] | Parallel RCT Single tertiary centre | Fosinopril  RAAS  Proteinuria reduction at 4. 8, 12 weeks  (inclucing tubular proteinuria)  Adverse events | N=45/57 evaluated  **Fosinopril-predn**. **group:** N= 25/30 evaluated  **Prednisone group**:  N=20/27 evaluated  Age: mean age 8.7±3.5 yrs (Fosinopril-Pred.), 8.7±3.5 yrs (Predn.)  Sex (M/F): 32/15  Histology: MCD (3), FSGS (10), MNS (3), MCGN (4), MesPGN (11), no biopsy/ no information (14) | **Fosinopril-prednisone group:**  Fosinopril for 12 weeks (5mg/d for <5yrs of age; 5-7.5 mg/d for 5-10 yrs; 10 mg/d for > 10 years)  + Prednisone for 12 weeks (2mg/kg/d then reducing by 5 mg/d every 4 weeks to 1 mg/kg/d)  **Prednisone group:**  Prednisone for 12 weeks (2 mg/kg/d then reducing by 5 mg/d every 4 weeks to 1mg/kg/d)  **Co-interventions**: none | Proteinuria (g/d) at 4,8,12 weeks:  4 wks: Fos+Pred.: 1.25±0.64, Pred.: 2.52±0.56  (p<0.05)  8 wks: Fos+Pred.: 1.16±0.45, Pred.: 2.42±0.24  (p<0.05)  12 wks: Fos+Pred.: 1.10±0.41, Pred.: 2.05±0.46 (p<0.05)  Proteinuria reduction:  Significant differences between the start and completion of therapy in both groups  Fos+Pred: p<0.01; Pred. p<0.05.  Urinary retinol binding protein (RBP) and beta-2 microglobulin:  Significantly lower concentrations in Fos+Pred. group compared to pred. group at 12 wks (p<0.01)  Adverse events:  No significant differences between groups in Hb, potassium level, serum albumin, serum cholesterol, Creatinine clearance, blood pressure at 4, 8, 12 weeks |
| 24 | DUET Study 2017 [31, 32]  USA, Belgium, Czech Republic, Italy) | [Trachtman H](https://www.ncbi.nlm.nih.gov/pubmed/30361325), DUET: A phase 2 study evalu  ating the efficacy and safety of sparsentan in patients with FSGS. Journal of the American Society of Nephrology  2018;29(11):2745-54. [MEDLINE: 30361325]  [Komers R](https://www.ncbi.nlm.nih.gov/pubmed/29142983). Efficacy and safety of sparsentan compared with  irbesartan in patients with primary focal Segmental glomerulosclerosis: randomized, controlled trial design. Kidney  International Reports 2017;2(4):654-64. | Parallel RCT (Phase 2)  Multi-centre (44 study centres in USA (also children), Europe (Age 18-75 yrs) | Sparsentan  Irbesartan  Multi-Drug-Resistant/ Refractory SRNS  Proteinuria reduction at 8 weeks  Remission status at 8 weeks  Blood pressure at 8 weeks  Biochemical parameters at 8 weeks  Adverse events  Qualityof Life | **All:**  N=96/109 analysed  Age (range): 8-75 yrs  Age  Sex (M/F): 60/49  Histology: all FSGS  **N=23**  **Age ≤ 18 yrs:**  **Sparsentan Group:**  N=13 (children)  N=73 (all)  **Irbesartan Group:** N=10 (children)  N=36 (all) | **Sparsentan Group** (dual endothelin and angiotensin inhibitor):  200 mg, 400mg or 800 mg daily for 8 weeks; data from these groups pooled; participants < 50 kg received half dose in each group.  **Irbesartan group:**  150 mg daily for first week then 300 mg daily for next 7 weeks; participants<50 kg received half dose in each group.  **Co-interventions:**  Immunosuppressive treatments except RTX and CPH | **Efficacy - not differentiated between children and adult group:**  Reduction in urinary protein/creatinine ratio (UPC) from baseline to 8 weeks (% with 95% CI):  Sparsentan: -44.8 % (-52.7 to 35.7%) – pooled dose groups analyses (highest dose – 400 and 800 mg -: -47.4%; p=0.001)  Irbesartan: -18.5 % (-34.6 to 1.7%)  (p=0.006)  % with UPC ≤ 1.5 g/g with >40% reduction at 8 weeks (FPRE: FSGS partial remission end point = PARTIAL Remission):  Sparsentan: 28.1%  Irbesartan: 9%  (p=0.04)  Complete remission:  Sparsentan: 3/73 (4.1%)  Irbesartan: 1/36 (2.8%)  Changes to baseline albumin, 24h urinary protein, GFR (similar stable in both groups), BP (BP was reduced in sparsentan group but not in irbesartan group) , creatinine, lipid profiles after 8 weeks  **Safety:**  The overall incidences of treatment-emergent adverse events, drug-related or serious treatment-emergent adverse events were similar between sparsentan and irbesartan group:  More common in sparsentan group:  Hypotension, dizziness, edema, gastrointestinal (vomiting, diarrhea, nausea)  More common in irbesartn group:  Fatigue, nasal congestion, upper respiratory infections, muscle spasms, hyperkalemia.  Quality pf Life (SF36 in adults; PEDsQL: Pediatric Quality of Life Inventory, vs.4.0 in < 18 yrs):  So far, not reported |
| **Supportive therapies** | | | | | | | |
| 25 | [Hari P, 2018](https://www.ncbi.nlm.nih.gov/pubmed/30091061)  [33] India | Effect of atorvastatin on dyslipidemia and carotid intima-media-thickness in children with refractory nephrotic syndrome: a randomized controlled  trial. Pediatr Nephrol. 2018 Dec;33(12):2299-2309. | Parallel RCT |  | N=26/30 analysed  Atorvastatin  N=13/15 analysed  Placebo  N=13/15 analysed  Age 5-18 years  Sex: 70% male  Histology: MCD (9), FSGS (9), MPGN (11), membranous (1). | **Atorvastatin Group:**  Atorvastatin as fixed dose 10 mg/day for 12 month  Mean dose of atorvastatin 0.39±0.12 mg/kg/d (range 0.26 to 0.64 mg/kg)  **Placebo Group:**  Placebo for 12 months  **Co-interventions:**  None were receiving calcineurin-inhibitors (stopped at leats 6 month before, n=27).  Alternate-day prednisolone 0.2-0.3 mg/kg (n=3)  All patients received enalapril | **Summary: 12-month follow-up:**  No significant difference in lipid levels in atorvastatin group compared to placebo (LDL-C, Total cholesterol, Tricglycerides, , VLDL-C, high-density lipoprotein cholesterol, Apolipoprotein A, Apolipoprotein B)  Median percentage change in between baseline and 12 months:  LDL-C  Atorvastatin:15.8% (-0,9, 53.2)  Placebo: 9.5% (-12.6, 39.8)  P=0.41  Apolipoprotein B:  Atorvastatin: 19.9 % (-13.1. -33.9)  Placebo: 1.1 % (-14,4, 41.4)  P=0.008  No significant median percentatge change in total cholesterol, triglyceride, VLDL-C. HDL-C, apoA amd hs-CRP (p>0.5).  **LDL-C [mg/dl]**  Baseline:  Atorvastatin: 209.5±46.4  Placebo: 208.2±47.6  At 3 month:  Atorvastatin: 168.9±70.6  Placebo: 187.4±66.8  At 6 month:  Atorvastatin: 157.4±68.9  Placebo: 172.7±65,7  At 12 month:  Atorvastatin: 163.4±103.0  Placebo: 194.2±120.3  No significant correlation g´between change in lipid levels and per-kg dose (p>0.1).  No significant difference in cIMT in both groups at 6 months (Atorvastatin: 0.43±0.08 vs- Placebo: 0.45±0.06; p=0.7) and 12 months (p=0.9), also not in brachial artery flow-mediated dilation (%).  **Adverse events:**  Infections most common:  Upper respiratory infections (27 episodes)  Acute gastroenteritis (6 episodes)  Pyoderm (2 episodes)  Other (backache, lower leg pain, calf cramps, knee pain, headache, abdominal pain, poor appetite, vomiting)  Median Creatinkinase at 12 months:  Atorvastatin: 96 (58-160.5) IU/l  Placebo: 108.3 (56.3-153.5) IU/l  None had CK more than 3-times the upper limit of normal or symptoms suggestive of rhabdomyolysis.  Liver enzymes in normal range |
| 26 | Chongviriyaphan, 1999 [34]  Thailand | Effects of tuna fish oil on  hyperlipidemia and proteinuria in childhood nephrotic syndrome. Journal of the Medical Association of Thailand 1999;82  Suppl(1):122-8. [CRSREF: 3333537; MEDLINE: 10730531] | Cross-over RCT  Single centre | Tuna fish oil  Proteinuria reduction at 8 weeks  Lipid status at 8 weeks | N=5/6 completed study  Age: 7-17 years  Sex: All male  Histology: FSGS (3), MesPGN (1), No info (1) | Patients with no response to corticosteroids and CPH  **Treatment group:**  Tuna fish oil 8 capsules/d for 8 weeks  **Control group:**  Placebo (olive oil) 8 capsules /d for 8 weeks  **Co-intervention**: prednisolone, dipyrridamole, aspirin, coumadin, calcitriol | Urine protein excretion at 8 weeks:  No significant differences  Serum Creatinine and CrCl at 8 weeks:  No significant differences  Lipids (triglycerides, Cholesterol, HDL, LDL): No significant differences  Abstract summary of results:  The results showed no statistically significant difference in serum creatinine, triglyceride, cholesterol, urine protein and creatinine clearance between fish oil supplemented group and placebo group. |
| **Ongoing studies** | | | | | | | |
| 27 | Ghiggeri GM | Ofatumumab in children with steroid- and calcineurin-inhibitor-resistant nephrotic syndrome: a double-blind randomized, controlled, superiority trial.  [www.clinicaltrials.gov/ct2/show/NCT02394106](http://www.clinicaltrials.gov/ct2/show/NCT02394106) (date registered 20 March 2015) | Paralell RCT |  | N=50 (estimated enrolment)  Age 2-18 yrs  Start of study: 03/2015  Estimated study completion date: 03/2018 | **Run-in period (3 month):**  Careful review of compliance  Withdraw any immunosuppressive therapies:  prednisone will be tapered off by 0.3 mg/kg per week until complete withdrawal;  •calcineurin inhibitors and mofetile mycophenolate will be decreased by 50% and withdrawn after 2 additional weeks  In order to minimize the risk of complications of uncontrolled INS a treatment with ACE-inhibitor at 6 mg/m2 will be maintained or started in all patients.  Randomization will be stratified by eGFR at randomization: ≥90 and <90 ml/min/1.73 m2.  **Intervention Group:**  Ofatumumab 1500 mg/1.73m2 administered once, diluted in 1000 ml of normal saline  **Comparator Group:**  Normal saline, 1000 ml, administered once | **Primary Outcome:**  Complete or partial disease remission at 6 months  **Secondary Outcome:**  Complete or partial disease remission at 12 months  Adverse events at 1, 3, 6, 9 and 12 months after drug/placebo infusion  Measurement of frequency and severity of adverse events due to drug infusion  Abnormal laboratory values at 1, 3, 6, 9 and 12 months after drug/placebo infusion  Record of abnormal values in biochemical tests and hematology assessments. |
| 28 | Li Y.  China | Availability study of ACTH to treat children SRNS/SDNS.  [www.clinicalTrials.gov/show/NCT02972346](http://www.clinicaltrials.gov/show/NCT02972346) (date registered 23 November 2016) | Parallel RCT  Multi-center |  | N=42  Age: 3-12 yrs (SRNS/ SDNS) | **ACTH Treatment Group:**  Routine treatment plus ACTH 0.4 Unit/kg/day (Maximum 25Unit) for three consecutive days every 4 weeks  **Control Group:**  Routine treatment | To investigate the efficacy and safety of ACTH to treat NS  **Primary outcome measures:**  24h-proteinuria excretion (time frame: 1.5 yrs)  **Secondary outcome measures**:  Time to relapses (time frame: 1.5 yrs) |
| 29 | Trachtman 2018, [35]  USA | Trachtman H  Randomized clinical trial design to  assess abatacept in resistant nephrotic syndrome. [ClinicalTrials.gov: NCT02592798; Other: EudraCT2015-005450-36] | RCT, placebo controlled, quadruple blind, parallel Arms with switchover (Phase II) |  | N=90 (estimated enrolment)  Age  Treatment-resistant nephrotic syndrome.  Patients stratified for age (<18 yrs and >/=18) and APOL1 risk status  Start of study: 03/2016  Estimated completion date: 06/2020 | **Part 1:**  **16 weeks parallel arms:**  **Group 1: IV Abatacept** (dose adapted to weight from 500-1000 mg. Children < 18yrs and < 75 kg: 10 mg/kg/dose  **Group 2: Placebo** (normal saline)  On days 1, 14, 28, then every 28 days  **Part 2:**  **16 weeks cross-over:**  Group 1: Placebo (normal saline)  Group 2: IV Abatacept  **Part 3:**  169 day abatacept extension with all receiving abatacept  **Co-interventions:**  Standard immunosuppression (CNI, MMF, prednisone) – unchanged in 1 month  ACEi and ARB | **Primary Outcome:**  Difference in % of participants who achieve a renal response by 113 days (end of 1^st^ 16 week parallel group study)  **Secondary Outcome:**  Change in proteinuria, GFR, remission, quality of life (PROMIS)  Adverse events |
| 30 | Basu B, 2015  India | Efficacy and safety of rituximab to that of calcineurin inhibitors in children with steroid  resistant nephrotic syndrome  NCT02382575 | Open label RCT |  | N=120 (estimated enrolment)  Age: 3-16 yrs  Start of study 03/2015  Estimated study completion date: 03/2017 | **Rituximab Group:**  Rituximab infusions weekly for 2 to 4 doses over up to 4 weeks  **Comparison group (Tacrolimus):**  Oral tacrolimus given until the child has achieved 6 months of relapse free survival | 12-month relapse-free survival  Adverse effects |

**Table S3: Observational Studies in SRNS – Conservative Treatment**

| **65** | **Title of Publication** | **Study design** | **Key words** | **N** | **Population Characteristics** | **Treatment** | **Outcomes** |
| --- | --- | --- | --- | --- | --- | --- | --- |
| **SRNS Registries – Treatment and Outcome Studies** | | | | | | | |
| Trautmann A, 2015 [36] | Spectrum of Steroid-Resistant and Congenital Nephrotic syndrome in children: The PodoNet Registry Cohort | Retro-/prospective registry study  Multicenter (67 in 21 countries) | Registry  SRNS Spectrum  ESRD  Intensified immunosuppression  Steroid pulses  CPH  CNI  MMF  Rituximab  Extrarenal manifestations | 1655 | **Primary SRNS and congentical NS (NS)**  Age at onset: <3month (CNS): 98 (6.3%)  3-12month: 106 (6.9%)  1-5 yr: 786 (50.9%)  6-11yr : 350 (22.7%)  ≥ 12 yr: 204 (13.2%)  Histopathology:  Biopsies performed n=1368 at a median of 2 (1-8) months after 1^st^ disease manifestation  FSGS 56%, MCN 21%, MesPGN 12%, DMS 3%  Genetic Screening:  Screening performed in 1174/1655 participants: Genetic diagnosis established in 23.6% of children (*NPHS2* 49.8%, *WT1* 17.3%, *NPHS1* 14.8%, *SMARCAL1* 4.3%....)  Genetic disease depends on age:  <3month (CNS): 66.3%  3-12month: 35.6%  1-5 yr: 21.8%  6-11yr : 15.6%  ≥ 12 yr: 16.1%  Extrarenal manifestations: in 17.3% of patients, most common CNS abnormalities (microcephaly, brain anomaly and/or mental retardation) with 5.3%. | All received intensified immunosuppressive medication:  Available information on pharmacologic therapies N=1283  **Treatments** at any time during observation period:  Steroids: 42.6%  IV CPH Pulses: 3.5%  Oral CPH: 21.2%  CsA: 66.3%  Tac: 12.2%  MMF: 24.9%  Rituximab 6.6%  **Co-medication:**  Oral steroids 63.3-93%  RAS antagonists: 76% (ACEi 72.3%, ARB 33.7%) | **Response to applied therapies at any time: (complete/ partial remission)**  Oral steroids only: 10.9%/ 9.3%  Steroid pulses: 9.0%/ 15.1%  CPH (oral or IV): 16.8%/ 8.4%  CNI: 44%/ 21.6%  MMF: 38.5%/ 12.5%  CNI+MMF: 47.9%/ 21.8%  Rituximab 43.9%/ 15.2%  **Renal Function:**  **ESRD at last observation:**  CKD stage 1: 411 (60.2%) (of these complete remission 33%, partial remission 22.5%)  CKD stage 2: 178 (26.1%)  CKD stage 3: 55 (8.1%)  CKD stage 4: 22 (3.2%)  CKD stage 5: 17 (2.5%)  RRT: Dialysis: 11.7%, Tx 14.2%  Deceased: 2.3% Loss of follow-up: 10.6%  **ESRD status depending on age at disease onset:**  <3month (CNS): 60.9%  3-12month: 37.8%%  1-5 yr: 21.5%  6-11yr : 27.2%  ≥ 12 yr: 28.6% |
| Trautmann A, 2017 [37] | Long-term outcome of steroid-resistant nephrotic syndrome in children | Retro-/prospective registry study  Multicenter (67 in 21 countries) | Treatment response within 1 year  Treatment of genetic SRNS  Renal survival  ESRD progression risk factors | 1354 | **Primary SRNS** ≥ 3 months:  Age at onset:  3-12month: 113 (8.3%)  1-5 yr: 726 (53.6%)  6-11yr : 325 (24.0%)  ≥ 12 yr: 190 (14.0%)  Median duration of follow-up:  3.6 (1.5-6.8) yeras  Histopathology:  MCN 249 (21.6%), MesPGN 144 (12.5%), FSGS 666 (57.6%), DMS 26 (2.3%), Other 72 (6.1%) Unknown/no biopsy 199  **Patients for evaluation long-term outcome**:  Sporadic, non-genetic disease: n=713  Genetic disease: n=212  Familial disease (without established genetic diagnosis): n=139  Sporadic disease (no genetic screening perforemd): n=290  **Patients for evaluation of IIS efficacy within the 1st year after disease onset**: n=612  Sporadic, non-genetic disease: n=387  Genetic disease: n=74  Familial disease (no genetic cause identified): n=36  Sporadic disease (no genetic screening performed): n=115 | **Treatment with IIS within the 1^st^ year**:  1 drug: 390/612  2 drugs: 173/612  ≥ 3drugs: 59/612  Treatment episodes:  ORAL  CNI n=433  CPH n=98  MMF n=24  CNI+MMF  IV Pulses  Steroid Pulse n=236  IV+ORAL:  Ster.P.+CNI n=49  Ster.P.+other N=17  CPH P+other 8  Rituximab n=7 | **Short-term outcome:**  **Response to each IIS treatment:**  **Complete/ Partial/ No remission:**  ORAL:  CNI: 29.8%/ 18.9%/ 51.3%  CPH: 9.2%/ 8.2%/ 82.7%  MMF: 8.3%/ 8.3%/ 83.3%  CNI+MMF: 11.8%/ 29.4%/ 58.8%  IV Pulses:  Steroid P: 6.8%/ 10.6%/ 82.6%  IV P + ORAL:  Steroid Pulse+CNI: 8.2%/10.2%/81.6%  Steroid Pulse+Other: 5.9%/ 5.9%/88.2%  CPH Pulse+other: 12.5%/12.5%/75%  Rituximab+other: 28.6%/ 0/ 71.4%  **Best Response to IIS regimen within the 1^st^ year:**  **All patients (n=612):**  Complete remission: 150/612 (24.5%)  Partial remission: 101/612 (16.5%)  No response: 361/612 (59%)  **Sporadic, non-genetic SRNS (n=387):**  Complete remission: 92/387 (23.8%)  Partial remission: 67/387 (17.3%)  No response: 228/387(58.9%)  **Genetic disease (n=74):**  Complete remission: 2/74 (2.7%), transiently  Partial remission: 8/74 (10.8%)  No remission: 64/74 (86.5%)  **Long-Term Outcome (Renal survival):**  **ALL:**  5yr: 74% (95% CI 71% to 77%)  10yr: 58% (95% CI 53 to 61%)  15 yr: 48% (95% CI 43 bto 53%)  **Complete vs. Partial Responder vs. non-responder:**  10yr: 94% vs. 72% vs. 43% (p<0.001)  15 yr: 94% vs. xx vs. 37% (p<0.001)  **IIS-responsive sporadic SRNS vs. Non-responder vs. Genetic SRNS:**  10 yr: 96% vs. 53% vs. 27% (p<0.001)  15 yr: 96% vs. 48% vs. 17% (p<0.001)  **Genetic disease: *NPHS2-* vs. *WT1-*associated nephropathy:**  10 yr: 28% vs. 23%  **Histopathology MCN vs. FSGS vs. DMS:** 5yr: 92% vs. 69% vs. 80%  10yr: 79% vs. 52%  15yr: 79% vs. 27%  **Risk factors for progression to ESRD:**  Lower ESRD risk: age at onset 1-5 yrs, complete- and partial-IIS-responsiveness  Increased ESRD risk: advanced CKD at disease onset, Histopathology: DMS and FSGS, genetic diagnosis |
| Mekahli D, 2009 [38] | Long-term outcome of idiopathic steroid-resistant nephrotic syndrome: a multicenter study | Retrospective study  8 centres in Belgium, France Switzerland | Treatment response  CsA  CsA+MMF  CPH  Renal survival  Growth  Complications (thrombosis, infections…) | 78 | **Primary Steroid Resistance:**  N=45 (58%)  **Secondary Steroid Resistance:**  N= 33 (42%)  Median age at onset: 4.4 (1.1-15.0) yrs  Median age at last follow-up: 13.8 (2.8-32.3 yrs)  Median follow-up: 7.7 (1.0-19.7) yrs  Gender: M/F: 45/33 (ratio 1.4)  Initial Histology: MCD 35 (45%), FSGS 33 (42%), DMS 10 (13%)  Genetic screening: not reported | **18 different treatment strategies**, the main agents used were:  CsA alone (N=29, 37%): 150 mg/m2 daily in tweo divided doses to achieve a 12 h trough blood level 100-150 μg/l  CsA alone, followed by CsA+MMF (N=18, 23%): MMF 1200 mg/m2 daily in two divided doses  CPH 2-2.5 mg/kg daily for 10-12 weeks (max. cumulative dose of 180 mg/kg per course) followed by CsA (N=4, 5%)  Other combinations (N=22, 28%)  Treatment of symptoms (N=5, 6%) | **Remission status/Renal function at last follow-up:**  Complete remission: 35/78 (45%)  Partial remission: 10/78 (13%)  No remission: 12/78 (15%, incl. 2 patients died, 1 pat. requiring bilateral nephrectomies)  ESRD/Transplanted: 20/78 (26%), of those 10 with recurrence (7 with primary steroid resistance, 3 with secondary steroid resistance), 3 with 2^nd^ Tx, 1 with 3^rd^ Tx, 3 on dialysis.  Normal renal function: 48/78 (62%)  **Growth:**  Median initial height SDS at diagnosis: 0.4 (-2.8 ±3.9) (n=73).  Median height SDS at last follow-up: -0.2 (-5.1± 3.4) (p<0.001).  Treatment with GH in 10/73 patients: No statistical difference in growth (p=0.054).  **Long-term outcome:**  Renal survival rate at 5 yrs: 75%, at 10 yrs: 58%, at 15 yrs: 53%.  Patient survival rate at 15 yrs: 97%  Only independent risk factor for ESRD: age at omnset > 10 yrs (p<0.001)  **Complications:**  Thrombosis 6/78 patients (8%) (4 cerebral veins, 1 iliac veine, 1 inferior vena cava)  Severe bacterial or viral infections requiring hospitalization: 25/78 (31%) (most frequent: Streptococcus pneumonia).  Death: 2/78 (2%) (1 fulminate pneumococcial sepsis during relapse of NS, 1 therapy-refractory relapse with renal and hemodynamical failure) |
| Büscher A, 2016 [39]  Germany | Rapid Response to Cyclosporin A and Favorable Renal Outcome in Nongenetic Versus Genetic Steroid-Resistant Nephrotic syndrome | Retrospective study  Multicenter (8) | Treatment reponse  CsA and renal survival  in non-genetic, genetic SRNS,  CNS  ESRD | 231 | **Primary SRNS:** N=169 (73%)  **Non-genetic**: 69/169 (58%)  Median age at onset 61 (32-103) months  Histology:  FSGS (68/98, 69%), MCN (23/98, 23%), DMS (1/98), Other (6/98)  **Genetic**: 71/169 (42%)  Median age at onset 42 (15-96 months)  Histology:  FSGS (43/65, 66%), MCN (11/65, 17%), DMS (9/65, 14%), Other (2/65)  **CNS:** N=62 (27%) (97% genetic)  Histology: DMS 15/62 (40%), Finish type 8/62 (21%). FSGS 8/62 (21%), MCN 5/62 (13%)  Median observation time of 113 nonths (IQR, 50-178)  Gender M/F: 106/125  Genetic screening: Performed in all patients for NPHS2 and WT1; following an age-ralted algorithm for NPHS1, NPHS2, LAMB2, TRPC6, PLCE1.  In 68/231 gene panel diagnostic was performed | **Non-genetic, primary SRNS:**  CsA: 81/98  RAAS: 80/98  **Genetic SRNS:**  CsA: 32/68 (47%)  RAAS: 51/64 (80%)  **CNS:**  Low exposure rate to CsA 9/56 (16%)  RAAS: 40/52 (77%) | **Non-genetic, primary SRNS**  Short-term outcome (response to CsA):  Complete remission: 49/81 (60%)  Partial remission: 15/81 (31%)  No response: 17/81 (21%)  Median time to complete response: 2.5 (1-5) months  82% of patients responded within 6 months of CsA treatment.  Median time to partial response: 10.5 (3-37) months  Long-term outcome (Renal survival):  Normal renal function: 69/96 (72%); CKD 3/96;  ESKD 24/96 (25%), 2% of patients with complete remission, 33% with PR, 53% with no remission.  Median time to ESKD: 36 (16-101) months  Recurrence after Tx: 8/16 (50%)  **Genetic SRNS:**  Short-term outcome (response to CsA):  Complete remission: 1/32 (3%) within 2.5 months (ACTN4-mutation)  Partial remission: 5/32 (16%) (3 in NPHS2, 2 in WT1)  No response: 26/32 (81%)  Long-term outcome (renal survival):  Median time to ESRD 44 (5-76) months  Development of ESRD: 66%  Normal renal function at end of observation: 26%  Recurrence after RTx: 2/29 (7%)  **CNS:**  Short-term (response to CsA):  Complete response 1/9 (comp. het. *NPHS1* mut.) within 2 months  Partial remission: 0/9  No remission: 8/9  Long-term:  Median time to ESRD 24 (7-40) months  Development of ESRD: 83%  Normal renal function at end of observation: 10%  Recurrence after RTx: 0%  **Non-genetic vs. genetic SRNS:**  Patients with non-genetic SRNS developed less frequently ESRD (27% vs. 74%, p<0.001) and CKD (3% vs. 8%, p=0.16).  Normal renal function preserved in 70% vs. 19% (p<0.001).  Median renal survival: 205 months vs- 48 months (p>0.001)  98% of patients with non-genetic disease and complete response to CsA maintained a normal renal function. |
| Büscher A, 2010 [40]  Germany | Immunosuppression and Renal Outcome in Congenital and Pediatric Steroid-Resistant Nephrotic Syndrome | Retrospective Study  (2 centres) | Treatment response and renal survival in non-genetic, genetic SRNS and CNS | 91 | **Primary SRNS:** N=65  **Non-genetic**: 41/65 (63%)  Mean age at onset 77.6±53.2 months  Histology (40/41):  FSGS (28), MCD (10), DMS (1), MesProGN (1)  **Genetic**: 24/65 (37%)  Mean age at onset 43.5±42.8 months  **CNS:** N=26 (100% genetic)  Mean follow-up: 103.0±68.2 months | **Non-genetic, primary SRNS:**  CsA: 31/41  RAAS: 34/41  **Genetic SRNS:**  CsA: 7/24  RAAS: 21/24  **CNS:**  CsA: 5/26  RAAS: 21/26 | **Non-genetic, primary SRNS**  Short-term (response to CsA):  Complete remission: 17/31 (55%)  Partial remission: 4/31 (31%)  No response: 10/31 (32%)  Long-term:  Mean time to ESRD 50.1±47.0 months  Development of ESRD: 29%  Normal renal function at end of observation: 63%  Recurrence after RTx: 9%  **Genetic SRNS:**  Short-term (response to CsA):  Complete remission: 0/24  Partial remission: 2 (WT1)/24(8%)  No response: 22/24(92%)  Long-term:  Mean time to ESRD 44.7±44.0 months  Development of ESRD: 58%  Normal renal function at end of observation: 38%  Recurrence after RTx: 0%  **CNS:**  Short-term (response to CsA):  No response to CSA: 5/5  Long-term:  Mean time to ESRD 37.4±27.6 months  Development of ESRD: 84%  Normal renal function at end of observation: 8%  Recurrence after RTx: 6%  **Non-genetic SRNS vs. Genetic (SRNS+CNS):**  Response to CsA significantly better (68% vs. 17%, p=0.05)  Less development of ESRD (29% vs. 71%, p<0.01)  Significantly slower progression to ESRD (p=0.02) |
| Ruf RG, 2004 [41] | Patients with mutations in NPHS2 (podocin) do not respond to standard steroid treatment of nephrotic syndrome | Retrospective analysis | Treatment response to CsA in *NPHS2*-associated glomerulopathy | 29 | **Genetic SRNS: *NPHS2*-associated glomerulopathy** (homozygous and comp.-heterozygous mutations)  With available data on immunosuppressive treatment  1 part of publication | Treatment with CsA or CPH (standard treatment) in combination with oral steroids | **Remission status:**  Complete rem.: 0/29 (0%)  Partial rem.: 5/29 (17%)  No rem.: 25/29 (83%) |
| Gipson DS, 2006 [42]  USA | Differential risk of remission and ESRD in childhood FSGS | Retrospective study | Treatment response and outcome  ESRD | 60 | Primary FSGS, SRNS  Age at diagnosis: range 1-21 years  Median follow-up: 33 months  Gender: M/F: 28/32  Histology: all primary FSGS  Genetic screening: not reported | **Treatments:**  Steroids: 35/60 (58%)  CNI 13/60 (23%)  CPH 7%  MMF 8%  ACEi/ARB 47% | **Remission status:**  Complete remission: 12/60, 20%  Partial remission: 20/60, 33%  No remission: 28/60, 47%  **Progression to ESRD:**  Complete rem. 1/12 (8%)  Partial rem. 2/20 (10%)  No rem. 18/28 (64%)  Renal survival was much improved in patients with CR/PR compared with no remission.  Complete remission was asscociated with 90% decreased risk of ESRD (HR0.10, 95% CI 0.001-0.79, p=0.03)  Death 2/70 (3%) after reaching ESRD |
| Abeyagunawardena AS, 2006 [43]  UK | Predictors of long-term outcome of children with idiopathic focal segmental glomerulosclerosis | Retrospective study | Treatment response  CPH  CsA  CPH+Vincristine  Relapses  Renal survival  Adverse events | 66 | Primary SRNS n=55  Secondary SRNS n=11  CPH: n=51  CsA: n= 15  CPH + vincristine: n=6  Mean Age at onset 6.4 yrs (range 0.4-14.1)  Gender M/F: 38/28  Histopathology: all FSGS | **Initial Treatment:**  **CPH 3 mg/kg/d for 8 weeks**  **or**  **CsA** 3-6 mg/kg/d in 2 divided doses, adjusted to 12hr trough level 50-150 ng/ml  **Co-medication:**  Oral prednisolone alternate-day-regimen  ACEi  In **resistant patients** additionally: Chlorambucil 0.2mg/kg/d for 12 weeks or Vincristine 1.5 mg/m2 weekly for 8 weeks | **Remission status:**  **CPH**  Complete rem.: 43% (22/51)  Mean time to rem.: 46 (8-78) days  **CsA:**  Complete rem.: 40% (6/16)  **CPH+Vincristine:**  Compl. Rem. 3/6 (50%)  **After all applied IS regimen:**  Complete and stable remission: 35/66 (53%)  Partial remission: 22/55 (33%)  No rem.: 9/66 (14%)  **Relapses in complete responders:**  22/35 relapses (63%)  13/35 sustained remission (37%)  **Renal survival at 10 yr:**  Responder: 90%  Non-responder: 48%  **Adverse events:**  Death 2/66 (complications due to heavy IS)  Peritonitis, septicemia 8/66  Renal vein thrombosis 1/66  Persistent hypertension 11/66  Convulsions 3/66 |
| Inaba A, 2016 [44]  Japan | Long-term outcome of idiopathic steroid-resistant nephrotic syndrome in children | Retrospective analysis  1 center Tokyo | Treatment reponses  CPH  CsA  ESRD  Adverse events/ complications | 69 | **Primary steroid resistance:**  N=54 (78%)  **Secondary steroid resistance:**  N=15 (22%)  Median age at diagnosis: 3.2 (1.1-15.3) yrs  Median age at last follow-up: 13.9 (6.9-28.1) yrs  Median follow-up: 10.1 (4.5-19.3) yrs  Gender M/F: 45/24  Histology: MCD 39 (57%), FSGS 22(32%), DMS (8 (11%)  Genetic screening: not reported | **Children with FSGS:**  **1^st^ SRNS treatment**:  **CPH** (N=11): 2.5 mg/kg/day (max. 100 mg/day) orally for 12 weeks (total cumulative dose 210 mg/kg)  or  **CsA** (N=10): achieving blood trough level 120-150 ng/ml for the initial 3 months, followed by 80-100 ng/ml for months 4-12, and 60-80 ng/ml for month 13-24, combined with prednisolone in tapering doses for 1 year)  **Children with MCD/DMS:**  **1^st^ SRNS treatment**  CPH (N=13) CsA (N=29)  ±  Methylprednisolone Pulses | **Short-term outcome:**  Initial Complete Remission rate at 4 month of treatment:  FSGS subgroup:  CPH: 0%  CsA: 40% (p=0.001)  MCD/DMS subgroup:  CPH: 38.5 %  CsA: 90.9% (p=0.001)  Only MCD subgroup:  CPH: 45.4%  CsA: 75%.  **Long-term outcome:**  Renal survival at 10 yrs:  FSGS subgroup:  CPH: 54.6%  CsA: 100% (p=0.013)  MCD/DMS subgroup:  CPH: 90.9%  CsA: 96.6%  Only MCD subgroup:  CPH: 100%  CsA: 100%  Permanent remission rates at 10 yrs: FSGS subgroup:  CPH: 27.3%  CsA: 10%  MCD/DMS subgroup:  CPH: 24.1%  CsA: 23.1%  **Risk factors for progression to ESKD:**  Initial immunosuppressive treatment CPH: HR 20.2, 95% CI 1.6-260.6; p=0.021  Initial histopathologic pattern: FSGS: HR 10.7, 95% CI 1.3-89.7; p=0.029)  Age at onset of > 11 yrs: HR 36.3; 95% CI 2.2-604.6; p=0.012)  **Complications:**  No death in follow-up period  Nephrotic-Syndrome or treatment-related:  Short stature (5, 7.2%), Excessive body weight (9, 13%), Obesity (4, 5.8%), Hypertension (22, 31.9%), ESKD (9, 13%),  CsA-related significant nephrotoxicity: 6/55 with repeat biopsies (10.9%) |
| Otukesh H, 2009 [45]  Iran | Management and Outcome of Steroid-Resistant Nephrotic Syndrome in children | Retrospective study  Single center | Treatment response  CPH  CsA  ESRD  Renal survival | 73 | **Primary SRNS** n=59 (80.8%)  **Secondary SRNS** n=14 (19.2%)  Mean follow-up duration: 6.0±4.2 yrs  Gender M/F: 38/35  Histopathology: 70/73  MCD 19 (27%), FSGS 26 (37%), diffuse MPGN 21(30%), global sclerosis 4 /6%).  Genetic screening: not reported | **Applied drugs:**  CPH: 53/73  CsA: 17/73  MMF 1/73 | **Response to initially applied IS:**  **CPH/ CsA/ MMF**  Complete rem.: 8/53; 7/17; 1/1  Partial rem.: 3/53; 1/17  No rem.: 33/53; 5/17  Relapses: 8/53; 4/17  Type of resistance to steroids (primary vs. secondary) was associated with IIS responsiveness: secondary SRNS significantly more frequently responsive (p=0.02).  Resistance to any IIS was also associated with resistamce to other IIS:  84% CsA resistant Patients were resistant to CPH  **Progression to ESRD:**  19/73 (26%)  **Renal survival:**  Mean 11.62 years (95% CI 10.01 to 13.23)  Mean time to ESRD 4.9±3.7 yrs.  Primary steroid resistance:  1yr: 94%, 5yr: 70%, 10yr: 56%, 15 yr: 34%  Secondary steroid resistance:  1yr: 100%, 5yr: 100%, 10yr: 83%, 15 yr: 83%  (p=0.03) |
| Straatmann C, 2013 [46]  USA | Treatment outcome of late steroid-resistant nephrotic syndrome: a study by the Midwest Pediatric Nephrology Consortium | Retrospective study  Multicenter (8) | Secondary SRNS  Late steroid resistance  Treatment responbse:  CPH  CsA  MMF  RTX  ESRD  Adverse events | 29 | **Secondary SRNS (Late steroid resistance)**  Mean age at onset: 4.3±3.4 yrs.  Median time from onset to development of late steroid resistance: 19 (2-170) months  Minimum follow-up: 2 years  Mean follow-up: 85±47 months  Gender M/F: 19/10  Histopathology (1^st^ biopsy): MCD 19/29, FSGS 7/29, mesangial hypercellularity 2/29, focal global gs 1/29 | Applied immunosuppressive drugs:  1 drug n=10 (34%)  ≥ 2 drugs n=18 (62%)  Most frequent sequential combination CNI+MMF  CPH n=9  CsA n=15  Tac n=13  MMF n=18  Rituximab n=4  None n=1  Co-medication:  oral steroid 25/27  ≥50% of total treatment time: 18/29  ACEi | **Remission status at end of individual drug:**  Complete or partial:  ALL: 69%  CPH: 67%  CsA: 87%,  Tac: 85%  MMF: 67%  RTX: 75%  **Status at latest follow-up:**  Complete: 14/29 (48%)  Partial: 6/29 (21%)  No: 6/29 (21%)  ESRD 3/29 (10%) of those 2 Tx, 1 FSGS recurrence  Mean age at ESRD 13.7±4.8 yrs.  Median time from late steroid resistance to ESRD: 7.3 (4.3-8.9 yrs)  **Adverse events:**  CPH: viral encephalitis, peritonitis  CNI: rhabdomyolysis, bacteremia, PRES, status epilepticus, nephrotoxicity (2), AKI (2), peritonitis.  MMF: sepsis, peritonitis |
| **Cyclosporine (CsA)** | | | | | | | |
| Ehrich JH, 2007 [47]  Germany | Steroid-resistant idiopathic childhood nephrosis: overdiagnosed and undertreated | Retrospective non-randomized trial | Treatment response  IV-MP+CsA  CsA  Relapses  Maintenance  CsA  CsA monitoring  Renal function  Growth  Side effects | 86 | **FSGS Group:**  n= 52 (A: 25/52; B: 27/52)  primary steroid-resistant: 46/52  secondary steroid-resistant: 6/52  **MCN Group:**  n= 14 (A: 3/14; B: 11/14)  **Genetic/ syndromal SRNS:**  n=20 (B: 9/20)  No IS treatment: 11/20  NPHS2: 1/11; WT1 2/11, SMARCAL-1:8/11)  Mean age at onset: 7±4 years  Mean age at final examination: 13±5 years  Mean follow-up: 5±3.6years  Genetic Screening: 47/78 children were analysed in NPHS2 (n=32), WT1 (n=6) or SMARCAL (n=7) | **Treatment:**  **A:**  **IV-MP Pulses:** 300-1000 mg/m^2^/day for 3-8 days  **+ oral prednisone** 40 mg/m^2^ on alternate days, after 6 month: tapered and withdrawn  **+ CsA** initial dose 150 mg/m^2^/day; initial trough levels 120-140 ng/ml. Long-term maintenance: 80-100 ng/ml.  **B:**  **oral prednisone** 40 mg/m^2^ on alternate days, after 6 month: tapered and withdrawn  **+ CsA** initial dose 150 mg/m^2^/day  All patients with partial remission were treated with another course of IVMP pulses and increasing CsA and Pred doses.  **“Rescue therapy” in FSGS Group:**  Plasmapheres 5/52 -> 2/5 in remission  MMF 3/52 -> 0/3 rem.  TAC 2/52 -> 0/2 rem. 1/1 | **FSGS Group:**  ALL – Remission status: Complete remission: 40/52 (77%), 3 after “rescue therapy”  Partial remission: after 6 months 6/52, after “rescue therapy” entered CR  No remission: 12/52 (33%)  Median time to remission: 4 (0.2-41) months  Patients who were treated with IV-MP Pulses additionally had a significantly better outcome: 84% vs. 64% cumulative proportion of sustained complete remission; p=0.02)  Relapse:  16/40 (40%) patients with CR developed 1^st^ relapse after a median interval of 1 year.  2/16 had frequent relapses  Maintenance:  Mean CsA trough levels 90±58 ng/ml  14/40 responders received lower CsA dosaage + MMV (median dosage 1000 mg/m^2^/d)  Renal function:  3/40 (8%) responsive patients developed CKD2  9/12 (75%) unresponsive patients developed CKD3-5; 6/12 entered CKD5, 5/6 requiring Tx,  p<0.001.  Growth at last follow-up:  0/40 responders had growth failure  7//18 non-responsive patients developed body height SDS < -2SDS  Side effects:  Striae, hypertrichosis, gingival hyperplasia: 8/40 responders  Cataract 1/40  No death  **MCN Group:**  ALL – Remission status:  Complete remission: 14/14  Median time to remission: 4 (1-24) months  Relapses:  4/14 had 1 or more relapses  Renal function: normal  **Genetic/syndromal SRNS:**  Remission status on 9/20 treated patients:  0/9 remission  Renal function:  19/20 progressed to CKD, 2/19 CKD2, 17/20 CKD5, Tx, 1 post-Tx-recurrence  Growth:  Growth failure in 50%  No death |
| Ghiggeri GM, 2004 [48]  Italy | Cyclosporine in patients with steroid-resistant nephrotic syndrome: An open-label, nonrandomized, retrospective study | Retrospective study  Multi-center (4 Italian centers) | Treatment response  CsA  Relapses  ESRD | 157 | Children and adults  **Non-genetic disease**: n=139  **CsA-treated group**: 55/139 (40%)  Median follow-up: 41 (23-92) months  **Non-CsA-treated group**: 84/139 (60%), of those CPH 45/139  Median follow-up: 48 (28-106) months  Median age at disease onset: 12 (4-32) yrs  Gender M/F: 84/55  Histology: FSGS 123/139 (88%), Mes.-IgM 16/139 (12%)  Genetic disease: 18/157 (11%), screening performed in all patients for NPHS2, NPHS1, α-Actinin 4 | Long-term CsA treatment > 2 yrs in non-genetic disease  **CsA-treated group:**  CsA 5 mg/kg/d starting doses, adjusted to maintain CsA serum trough levels between 50-100 ng/ml)  **Non-CsA-treated group:**  No treatment or CPH treatment (n=45): 2 mg/kg/d for 60 days  **Co-medication:**  Oral steroids, IV MP Pulses. | **CsA-treated group:**  **Response to CsA after 2 months:**  Complete remission: 20/55 (36%)  Partial remission:  No remission/ CsA intolerance: 35/55 (64%)  Relapses:  13/20 (60%) of CsA-responsive patients presented with ≥ 1 relapse after a follow-up of < 3 yrs  Median ages at disease onset:  CsA-responsive group: 5 (3-11) yrs  CsA-resistant/intolerant group: 8 (4-14) yrs  No-CsA-treated group: 19 (8-44) yrs  P<0.001)  **CPH-treated group:**  Non-responsiveness to CPH  **Progression to ESRD:**  CsA-responsive group:2/20 (10%)  CsA-non-responsive group: 21/35 (60%)  Non-treated group: 52/84 (62%)  (p=0.002) |
| Hamasaki Y, 2009 [49]  Japan | Cyclosporine and steroid therapy in children with steroid-resistant nephrotic syndrome | Prospective study  multicentre | Treatment response CsA  CsA monitoring  Renal function  Adverse events | 35 | **Primary steroid resistance:** n=26/35  **Secondary steroid resistance:** n=9/35  Age: 1-18 yrs  Median age at start of CsA treatment: 2.7 (1.4-15.0) years.  Age < 3 yrs: 20/35 (57%)  Mean duration from onset to CsA treatment: 3.4 (1.0-25.0) months  Median follo-up: 12 months  Gender M/F: 21/14  Histology: FSGS 7/35, MCD 23/35, diffuse mesangial proliferation (DMP) 5/35 | **CsA for 12 months**  Adjusted to maintain trough level of 120-150 ng/ml during initial 3 months, followed by 80-100 ng/ml during months 4-12.  **Co-medication:**  Oral prednisolone: 1mg/kg/day for 1^st^ 4 weeks, followed by 1mg/kg every other day months 2-12.  Patients with FSGS additionally: IV MP Pulses 30 mg/kg per day (max. 1g) for 3 days at weeks 1,2,5,9 and 13.  Antihypertensive drugs (calcium-channel blockers, ACEi, ARB), diuretics (furosemide), anticoagulants, statins | **Primary end point:**  **Remission rate at 12 months:**  Complete remission: 27/35 (77%)  MCD/DMP: 22/28 (79%)  FSGS: 5/7 (71%)  Partial remission: 2/35 (6%)  No remission/off-treatment: 6/35% (17%)  Mean CsA dose and trough level month 1-3:  6.0 (3.1-10.4) mg/kg/day; 110.2 (71.0-159.7) ng/ml  Mean CsA dose and trough level month 4-12:  5.1 (3.0-8.1) mg/kg/day; 88.6 (61.0-136.5) ng/ml  Relapses:  MCD/DMP group: 7/22 with complete remission  FSGS group: 2/5 with CR  **Secondary end point:**  **Remission rate at 4months:**  Complete remission: 28/35  Partial remission: 4/35  No remission: 3/35  **Change in renal function**:  No elevated serum creatinine  Similar eGFR at CsA start (119.9 ml/min*1.73m^2)^ and at month 12 (117.7 ml/min*1.73 m^2^)  Height:  Mean SD scores at CsA start and at 12 months: -0.38 and -0.08.  Mean difference in SDS for height was 0.30 (95% CI CI 0.10-0.50).  **Adverse events:**  **Major:**  Severe bacterial infections (peritonitis. Sepsis with multi-organ failure) 2/35  PRES 1/35  Mild CsA nephrotoxicity (26 biopsies were performed): 1/26  **Minor:**  Hypertension 10/35 (29%)  Hypertrichosis 18/35 (51%)  Hyperlipidemia 5/35 (14%), Obesity 3 (9%), Gastric pain 3/35 (9%).  2/35 (6%): Gingival hypertrophy, Alopecia, Sinus bradycardia  1/35 (3%): Glaucoma, Avne, Hyperuricemia |
| Tahar G, 2010 [50]  Tunesia | Cyclosporine A and steroid therapy in childhood steroid-resistant nephrotic syndrome | Retrospective study | Treatment response  CsA  Relapses  ESRD  Adverse events | 30 | **Primary steroid resistance** 19/30 (63%)  **Secondary steroid resistance** 11/30 (27%)  Mean age at start of treatment:  8 (1.4-14) years  Gender M/F: 19/11  Histology: FSGS 15/30 (50%), MCD 9/30 (30%), DMP 6/30 (20%)  Genetic screening: not reported | **CsA** initial dose of 150-200 mg/m^2^/day in 2 divided doses, adjusted to trough levels 100-150 ng/ml.  **Co-medication:**  Prednisone 30 mg/m^2^per day for 1 month, than on alternate days month 2-6  ± ACEi | **Remission status at 4 month:**  Complete remission: 15/30 (50%)  Partial remission: 9/30 (30%)  No remission: 6/30 (20%)  Time to complete/partial remission within:  1^st^ month: 6/24 (25%)  2^nd^ month: 8/24 (33%)  3^rd^ month: 8/24 (33%)  4^th^ month: 2/24 (8%)  Relapses:  5/15 with CR within 6 months after start of treatment  2/15 shortly after stopping CsA  8/15 maintained CR after 24 months of treatment and after discontinuation of treatment  CsA-resistant patients:  Trial of CPH and MMF -> no response  **Progression to ESRD:**  5/6 CsA-resistant children  4/15 CsA-responsive children  All: 9/30 (30%)  **Adverse events:**  Hypertrichosis (60%), gingival hypertrophie 27%, tremors 11.5%. |
| Liu Y, 2018  [51] China | Cyclophosphamide versus cyclosporine A therapy in steroid-resistant nephrotic syndrome: a retrospective study with a mean 5-year follow-up | Retrospective study  multicentre | Treatment response  CPH  CsA  Relapses  Adverse events | 127 | **CPH:** n=62  Mean age at onset: 9.6±5.2 years  Gender M/F: 28/34  Histology: MCD 38/62, FSGS 16/62, MesPGN 8/62  **CsA:** n=65  Mean age at onset 9.5±5.6 years  Gender M/F; 30/35  Histology: MCD 40/65, FSGS 15/65, MesPGN 10/65  Mean follow-up of 5 years  Genetic screening: not reported | **CPH treatment group:**  2-2.5 mg/kg/day orally for 3-6 months  Mean duration of CPH treatment: 4.4 ± 1.6 months  **CsA treatment group:**  1-5 mg/kg/day for 2 years adjusted to blood trough levels 100-200 ng/ml, reduced at 6-9 months after onset. Maintaining dose 1-3 mg/kg/day with trough levels 40-70 ng/ml month 12-24.  **Co-medication:**  Prednisone 1mg/kg/day, gradually reduced according to proteinuria status | **Relapse rate per year:**  Before treatment/ at the end of treatment:  CPH: 4.2±1.3/ 1.7±0.4  CsA: 4.2±3.7/ 1.2±0.6  (p=0.03)  5 year total relapse rate:  CPH: 11.2%  CsA: 6.2%  Relapse-free period CPH vs. CsA:  32.5±8.5 months vs. 38.3±7.5 months (p<0.001)  Mean time to first on-study relapse CsA vs. CPH: 10 month vs. 6 months (HR 0.63, 95% CI 0.12-3.71; p=0.0001)  CsA group showed a significantly delayed on-stidy relapse compared with CPH group (HR 0.82, 95% CI 0.47-1.93; p<0.0001)  **Remission at final follow-up:**  **CPH vs. CsA**  Complete rem.: 24/62 (39%) vs. 35/65 (54%) Partial remission: 8/62 (13%) vs. 11/65 (17%)  No remission: 30/62 (48%) vs. 19/65 (29%)  Total effective rate CPH vs. CsA: 51.6% (32/62) vs. 70.8% (46/65) (p=0.027)  CPH-resistant patients: 17/30 were switched to CsA-treatment after 6 months, 9 of them achieved compl. Remission, 8 failed.  **Adverse effects:**  **CPH:** Nausea 3/62, fungal infections 4/62, alopecia 2/62, reversible hair loss 2/62, leukopenia 5/62  **CsA:** nausea 4/65, fungal infections 3/65, reversible hair loss 3/65, tremors 3/65, leukopenia 3/65, hirsutism 4/65, alopecia 3/65 |
| Ingulli E, 1995 [52]  USA | Aggressive, long-term cyclosporine therapy for steroid-resistant focal segmental glomerulosclerosis | Retrospective study | Treatment response  CsA  ESRD  Side effects | 21 | **SRNS**  **CPH-resistant SRNS/FSGS**  Mean age 8.4±4.5 yrs  Mean duration of follow-up: 8.5±4.7 yrs.  Gender M/F: 13/8  Histology: all SRNS | **CsA** 6-7 mg/kg/day, adjusted to trough levels 100-200 ng/ml  Co-medication: low-dose prednisone | Median duration of CsA therapy: 27.5±22 (3 to 97) months  Median CsA dose 7 (4 to 20) mg/kg/day  At last follow-up:  Manintenance CsA 7/21  Discontinued 14/21 due to sustained remissioon (5/14), rising creatinine (5/14), non-compliance 2/14, CsA-resistancs 1/14, pregnancy 1/14  **At the end of CsA therapy:**  Mean proteinuria fell from 6.2±0.2 to 2.0±0.1g/24h (p<0.001)  Mean albumin increased from 1.95±0.04 to 3.41±0.04 g/dl (p>0.001)  Mean cholesterol level decreased from 472±12.7 to 257±5.3 mg/dl (p<0.005)  Mean creatinine increased from 0.78±0.02 to 1.16±0.03 mg/dl (p<0.005)  **Progression to ESRD**: 5/21 (24%)  **Side effects:**  Gingival hyperplasia n=6  Hypertrichosis n=8  Coarse facies n=4  Hypertension n=21  No need to discontinue treatment |
| El-Husseini A, 2005 [53]  Egypt | Long-term effects of cyclosporine in children with idiopathic nephrotic syndrome: a single-centre experience | Retrospective study | CsA  Response | 117  43 SRNS  74 SDNS | **SRNS (n=43) or SDNS (n=74)**  **SRNS patients:**  Mean age at CsA start: 12.1±5.9 yrs  Histopathology:  MCGN 11 (25.6%), FSGS 32 (74.4%) | **CsA**  Chidren > 6 yrs: 4-5 mg/kg/d in two divided doses; < 6yrs: 5-6 mg/kg/day in three divided doses, aiming at trough levels 100-150 ng/ml in the first 2 months, thereafter 50-100 ng/ml.  **Co-medication**: oral prednisolone 0.5 mg/kg/d | Mean CsA treatment duration: 33±12 months Mean CsA maintenance dose: 1.5±1 mg/kg/d  **Remission status SRNS patients:** Complete remission: 30/43 (69.8%)  Partial remission: 6/43 (13.9%)  No remission: 7/43 (16.3%)  Mean time to remission: 6.9±3.4 weeks  **Side effects:** Gym hyperplasia 13/43 (30.2%), Hypertrichosis (31/43 (72.1%), Hypertension (8/43 (18.6%), Renal dysfunction 5/43 (11.6%)  Indications for CsA discontinuation: Intentional stoppage 14/43 (32.6%), Resistance to CsA 6/43 (13.9%), Renal dysfunction 3/43 (7%), hypertension 3/42 (7%) |
| Klaassen I, 2015 [54] | Response to cyclosporine in steroid-resistant nephrotic syndrome: discontinuation is possible | Retrospective study  Single centre | CsA  Response  Treatment duration | 36 | **SRNS**  Median age at presentation 3.2 (0.06-15.0) years  Median follow-up 15.5 (1.8-27.7) years.  Histopathology: FSGS 23 (64%), MCD 13 (36%)  Genetic screening:  Performed in 33/36 (92%) in *NPHS2, NPHS1, WT1* | **CsA** 150 mg/m^2^/day in two divided doses, aiming at trough levels of 100-150 ng/ml in the first 12 months, thereafter 80-100 ng/ml  **Co-medication:**  Oral prednisone 30 mg/m^2^ a.d., discontinued over a time course of 2-6 months  ACEi | **Remission status:**  Complete remission: 19/36 (53%)  Partial remission: 10/36 (28%)  No Response: 7/36 (19%)  Median time to CR: 2 (range 0.5-7.8 years) months  **Change CsA to Tac:**  4/19 responders due to cosmetic side effects (hypertrichosis, gingival hypertrophy)  **Discontinuation of CsA possible:**  15/19 (79%) CsA-responders after a median of 3.1 (0.5-14) years  11/15: No further relapse with median follow-up of 9.7 years (0.7-21.6 yrs)  4/15 with relapses, responsive  **ESRD:**  0/19 responders  5/10 partial responders after median of 3.4 years (including 2 patients with *NPHS2* mutation)  4/6 non-responders after median of 1.5 years (including 3 genetic SRNS: 2 *NPHS2*, 1 *ACTN4*)  Total: 9/36 (25%) after a median of 2.4 years (0.5-18.1 years). |
| **Tacrolimus** | | | | | | | |
| Butani L, 2009 [55]  USA | Experience with tacrolimus in children with steroid-resistant nephrotic syndrome | Retrospective study  1 center | Tacrolimus  Treatment response  Relapses  Tac Monitoring  Side effects | 16 | **Primary steroid resistance:**  N=12  **Secondary steroid resistance:**  N=4  Median age at diagnosis: 5.2 (2-13.3 yrs)  Gender M/F: 10/6  Histology: MCD 7/16, FSGS 8/16, Membranous GN 1/16  Genetic screening in NPHS1 and NPHS2: performed in 12/16 patients, no genetic findings in those 12 patients in 2 genes | **Tacrolimus treatment**  Median start dose 0.1 (0.05-0.2) mg/kg/day  Target trough level 5-10 ng/ml  **Co-Medication:**  daily (4/16) /alternate (12/16) prednisolone  RAAS: 10/16 (ARB N=1, ACEI n=8, both N=1)  **Pre-Medication**: 3/16 (MP Pulses, CPH, chlorambucil, CsA) | **Remission status:**  Complete remission 15/16 (94%)  No remission: 1/16 (6%), already advanced CKD at Tac treatment start, was multidrug-resistant  **Median time to remission:**  Partial: 29 (13-180) days  Complete: 120 (16-730) days  3 with rapid reponse, 3 with response after > 200 days.  The remission rate was not affected by underlying histology.  **Relapses:**  9/15 weaned off steroids  (longest steroid-free interval: 28.5 (0.1-54) months).  6/15 remained on steroids due to steroid-responsive relapses  7/15 (47%): relapses during tapering steroids  **Tac Monitoring:**  Dose at last follow-up: 0.15 (0.06-0.38) mg/kg/day  Trough levels at last follow-up: 5 (1.8-12.3) ng/ml.  No significant differences among ethnicities.  **Side effects:**  Acute rise in creatinine during dehydration n: 1/16  Infectious complications: 2/16 during relapses (salmonella enteritids, streptococcal pneumoniae).  EBV-negative Hodgkin Lymphoma (after 6 months Tac)  Diabetes 0/16 |
| Wang A, 2012 [56]  China | Treatment of tacrolimus or cyclosporine A in children with idiopathic nephrotic syndrome | Prospective study, uncontrolled  Single center Zhejiang | Treatment response  CsA  Tac  Relapses  Adverse events | 74  SRNS n=34 | **CsA Group** (Oct 2007-July 2009):  N=24  SRNS: 8/24 (MCD 4/8, FSGS 2/8, MesPGN 2/8)  FRNS/SDNS: 16/24  Median age at onset: 7.6±4.5 yrs  Median age at treatment start: 7.7±5.0 yrs  Gender M/F: 18/6  **TAC Group** (Nov 2008-June 2011):  N=50  SRNS: 26/50 (MCD 13/23 performed biopsies, FSGS 5/23, MesPGN 0/23, IgMN 5/23)  FRNS/SDNS: 24/50  Median age at onset: 8.3±4.8 yrs  Median age at treatment start: 8.6±5.8 yrs  Gender M/F: 33/17  Genetic screening: only performed in NPHS2 in patients with biopsy-proven FSGS. | **CsA treatment group:**  CsA 3-4 mg/kg/d divided in 2 doses; adjusted to target trough level 100-150 ng/ml.  Overall final dose: 2.72±  0.59 mg/kg/day  **TAC treatment group:**  0.05-0.15 mg/kg/day, divided into 2 doses, adjusted to target trough levels 5-12 ng/ml.  Overall final dose: 0.087-0.28 mg/kg/day  Duration of treatment: at least 24 months  **Co-medication:**  Prednisone 1 mg/kg/day (max. 60 mg) for 4 weeks, alternate days until week 8, tapering off.  **Pre-medication:**  MMF 17/74  CPH 8/74 | **Short-term response within 6 month**  Induction of remission in SRNS:  **CsA:**  Complete: 3/8  Partial: 1/8  No: 4/4  **TAC:**  Complete: 22/26  Partial: 4/24  No: 0/26  TAC appeared to be better in inducing remission (p=0.001)  Maintaining of remission/no relapses (SRNS):  CsA: 1^st^ year: 1/3; 2^nd^ year: 1/3  TAC: 1^st^ year: 14/22, 2^nd^ year: 11/22  No significant differences between both groups  **Adverse effects:**  Nephrotoxicity: CsA: 4/24, Tac 0/50 (p=0.002)  Hirsutism: CsA: 8/24. Tac 0/50 (p<0.001)  No significant differences in ALT/AST elevation, gastrointestinal symptoms, transient hypertension, glucose intolerance and diabetes, early-stage cataract, psychiatric symptoms, severe infections, nutritional anemia. |
| Roberti I, 2010 [57]  USA | Long-term outcome of children with steroid-resistant nephrotic syndrome treated with tacrolimus | Retrospective study  Single center Livingston | Treatment response  Tacrolimus  Adverse events | 19 | Median age at onset: 10 (1.6-18) yrs  Gender M/F: 8/11  Histology:  FSGS 10/19, C1qN 4/19, membr.GN 2/19, MCD 1/19, MPGN 1/19, IgAN 1/19  Mean follow-up 55 (17-111) months  Genetic screening: Performed for NPHS2 an WT1 -> all negative | **Tacrolimus 0.1 mg/kg twice daily,** adjusted to blood trough level 5-8 ng/ml.  After complete remission: Tac was continued for at least 1 year and then slowly tapered by 20% each month over a 6-9 month period (Tac levels < 5 ng/ml).  **Co-medication:**  Prednisone a.d.  All ARBs (losartan 25-100 mg/day) and/or ACEi (enalapril 2.5-40 mg/dose/day).  **Pre-medication:**  8/19 patients: IV CPH (500-750 mg/m2 monthly, 3 doses), MMF (1200 mg/m2/day in 2 divided doses, 3 months), CsA 5 mg/kg twice daily, 3 months) or rituximab (375 mg/m2 weekly, 4 doses) | **Remission status:**  Complete remission: 11/19 (58%)  Partial remission: 6/19 (32%)  No remission 2/19 (9%)  Median time to response: 8 weeks (range 3 weeks to 3 months)  **Remission status at last follow-up:**  Loss of follow-up: 1/19  Sustained complete and partial remission 10/18 (55%)  No remission: 8/18 (45%) – 2 initial non-responders, 2 secondary tac resistance, 4 prpgression to ESKD.  Tacrolimus resistance after 16-18 months of treatment:  Primary resistance: 2/19  Secondary resistance: 4/19  **Adverse events:**  Significant side effects 3/19 (acute kidney injury with rising creatinine and hyperkalemia 1, hyperglycemia 2 in patients with positive family history with diabetes). |
| Jahan A, 2015 [58]  India | Clinical efficacy and pharmacokinetics of tacrolimus in children with steroid-resistant nephrotic syndrome | Prospective study  Single center Vellore, India | Treatment response  Tacrolimus  Relapses  Tac Monitoring  Pharmakokinetic  Renal function | 25 | Primary steroid resistance: n=18  Secondary steroid resistance: n=7  Age: 2-15 yrs  Median age at time if tacrolimus study: 7 (6-9) yrs  Gender ratio M/F: 1.3:1  Histology: FSGS 9/25 (36%), mild mesangial hypercellularity 15/25 (60%), 1/25 (4%) diffuse mesangial hypercellularity with focal tubular necrosis  Genetic screening: not generally performed due to financial constraints (only in 2 WT1 and NPHS2 -> negative) | **Tacrolimus** 0.10-0.19 mg/kg/day in two divided doses before meal for at least 3 months.  Median duration of Tac treatment: 14 (6-18) months  **Co-medication:**  All ACEi (enalapril 0.1-0.4 mg/kg/day) or ARB (losartan: 0.5-2mg/kg/day).  Prednisolone in tapering doses  **Pre-medication:**  11/25, of these 10/25 IV CPH Pulses, 1/25 MMF  **Pharmakokinetics:**  Age 2-9 yrs: 0h (C_0_), post-dose 0.5, 1, 1.5, 2, 4, 8, and 12h.  Age 10-15 yrs: 0h (C_0_), post-dose 0.5, 1, 1.5, 2, 2.5, 3, 4, 8, and 12h.  Tacrolimus concentration measured by liquid chromatography mass spectrometry. AUC_0-12h_ was normalized to body surface area (BSA) | **Remission status** on last follow-up at 14 months:  Complete remission: 16/25 (64%)  Partial remission: 4/25 (16%)  No remission: 5/25 (20%)  Median time to CR: 2.6 months  Median time to PR: 3.8 months  **Relapses:**  9/16 (56%) with complete remission until last follow-up  Minimum time to relapse after CR: 4 months  **Tac Pharmacokinetics:**  Mean C_0_ 2.78±2.03 ng/ml.  C_0_ remission group vs. relapse group: 2.95 ng/ml vs. 1.2 ng/ml, p=0.005  AUC_0-12h_ remission vs. relapse group: 79.75 vs. 35.15 μg*h/l; p=0.025)  Significant correlation between C_0_ and tacrolimus dose (p=0.01)  **Renal function:**  5/25 showed a rise of creatinine from 0.5 to 2.3 mg/dl (44.2-203.32 μmol/l) and fall of eGFR ranged from 30-65% from baseline. All were on Tac dose 0.1 mg/kg/day, 4/5 were in relapse.  Renal function did not improve after dose reduction by 25% in 72h, Tac was stopped and ACEi/ARB too. |
| Gulati S, 2008 [59]  India | Tacrolimus: a new therapy for steroid-resistant nephrotic syndrome | Prospective study | Tacrolimus  Remission status  Monitoring  Trough levels  Side effects  eGFR | 22 | Primary SRNS n=19  Secondary SRNS n=3  Mean age at onset: 7.33±5.9 yrs  Mean duration of follow-up: 290±126 days  Gender M/F: 20/2  Histopathology: MCD 9/22, FSGS 11/22, 2/22 diffuse mesangial hypercellularity | Tac 0.1 mg/kg/day in 2 divides doses, adjusted to trough level target 5.0-10.0 g/l for at least 1 year.  Co-medication:  Oral prednisone 60 mg/m2/d for 4 weeks, 40 mg/m2 a.d. for 4 weeks, tapered over next 4 weeks.  Pre-medication: 14/22:  IV CPH 5/14  Oral CPH 9/14  CsA 4/14 | **Remission status: n=19**  Complete remission: 16/19 (84%)  Partial remssion: 2/19 (10.5%)  No remission: 1/19  Mean time to achieve remission 63.2±44 days  Mean dose of Tac 0.18±0.07 mg/kg  Mean trough levels 9.54±5.13 ng/ml  Renal function (mean eGFR) prior Tac start and at end of study: 97.921.2 ml/min/1.73m^2^ vs. 96.4±18.4 ml/min*1.73 m^2.^  **Side effects:**  Withdrawn of Tac in 3/22 due to side effects (HUS 1/3, glucose intolerance 1/3, persistent diarrhea 1/3)  Watery diarrhea 7/22  Acute renal dysfunction 3/22  Hyperglycemia 2/22  Increasing hypertensive drug dose: 2/22 |
| Loeffler K, 2004 [60]  Canada | Tacrolimus therapy in pediatric patients with treatment-resistant nephrotic syndrome | Retrospective study | Treatment response  Tacrolimus  Monitoring  Adverse events | 16 | Major indications for Tac treatment: resistance/intolerance to prior IS treatments  Mean age at onset; 5.6 (1.6-13.3) years  Mean age at Tac start: 11.4 (3.5-18.1) years  Average time from diagnosis to Tac start: 5.3 (0.3-13.3 years, median 6 yrs)  Mean follow-up 6.5 (2.5-18) months  Gender m/F: 12/4  Histology: FSGS n=13, MCD n=1, IgA n=2  Genetic screening: not reported | **Tacrolimus** initial dose 0.1 mg/kg/d divided into 2 doses, adjusted to target levels 5.0-10.0 ng/ml  **Co-medication**: oral prednisone ad with tapering regimen, ACEi  **Pre-medication:** CsA n=15 (10/15 CsA responsive)  Chlorambucil n=5  MMF n=5  Levamisole n=3  IV MP n=3  CPH n=2 | **Remission status:**  Complete remission: 13/16 (81%)  Partial response: 2/16 (13%)  No response: 1/16  Average time to remission: 2 (0.5-5.5) months, median: 6 month  Relapse: 4/13 responsive patients  Mean Tac dose: 0.18mg/kg/day  Mean Tac trough level: 8.4 (4.4-12.8 ng/ml)  **Adverse events:**  Anemia 1/16  Seizure 1/16  Worsening or new-onset hypertension 5/16  Sepsis 1/16 (staph aureus, infected portacath) |
| **IV Steroid Pulses** | | | | | | | |
| Shenoy M, 2010 [61]  UK | Intravenous methylprednisolone in idiopathic childhood nephrotic syndrome | Retrospective study  Single center | IV methylprednisolone pulses  Treatment response | 16 | SRNS after 4 weeks standard prednisolone therapy  Median age at onset: 3.8 (1.6-11.9) years  Gender M/F: 12/4  Follow-up: 15-year study period  Histology: biopsy performed in primary and secondary non-responders  Genetic screening: negative in NPHS2 | **IV Methylprednisolone pulses** (15 mg/kg daily) for  3 days (n=6), 5 days (n=10) | **Confirmed initial SRNS after IV MP Pulses:**  6/16 (37.5%)  2/6 achieved remission with oral CPH for 8 weeks  2/6 non-responsive to oral CPH and CsA  2/6 responisve to combination of CsA and prednisone on alternate days.  6/6 remained a normal renal function  **Initial Responders:**  10/16 (62.5%), mean follow-up: 6.7 (1.6-14) yrs  3/10 developed secondary steroid resistance after 3, 31, 66 months and developed CKD stage 3-5  7/10 frequently relapsing nephrotic syndrome, requiring treatment with CPH, levamisole, tacrolimus, CsA, MMF |
| Zhang H, 2016 [62]  China | Children with steroid-resistant nephrotic syndrome: Long-term outcomes of sequential therapy | Retrospective  Single center Sichuan | IV Methylprednisolone Pulses  Adverse events | 63 | **Primary steroid resistance:**  N=28  Mean age at diagnosis: 10.8±7.2 yrs  Mean follow-up: 3.2±1.7 yrs  **Secondary steroid resistance:**  N=35  Mean age at diagnosis: 8.2±7.1 yrs  Mean follow-up: 2.9±1.8 yrs  Gender M/F: 43/20  Histology: not reported in all  Genetic screening: not reported | IV Methylprednisolone 15-30 mg/kg/day (max. dose 1000 mg) for 3 consecutive days.  +  Oral prednisone 60 mg/m^2^ divided into 3 doses (max. 60 mg) for 2-4 weeks,  Then tapering dose of oral prednisone  In case of relapse:  2 further courses of IVMP were administered | **Remission status:**  Complete remission: 19/63 (30%)  Partial remission: 16/63 (25%)  No remission: 28/63 (44%)  Mean time to achieve initial remission (CR/PR):  24.3±13.1 days.  **Non-responsive patients (28/63):**  Histology: FSGS 18, MCD 10  Used IS agents: CPH 10/28, CsA 1/28, Tac 7/28, MMF 6/28  Remission status after other IS agents:  CR 7/28 (25%), PR 14/28 (50%), No rem. 7/28 (25%)  **Adverse events:**  Transient hypertension 3/63 (4.8%)  Gastrintestinal symptoms, Nausea, Vomiting 5/63 (8%)  Excsessive weight gain and cushingoid appreanaces: 25/63 (40%)  Poor growth 26/63 (41%)  Hyperglycemia 3/63 (5%) |
| **Cyclophosphamide (CPH)** | | | | | | | |
| Rennert WP 1997 [63]  South Africa | Pulse cyclophosphamide for steroid-resistant focal-segmental glomerulosclerosis | Retrospective study  Single center | Cyclophosphamide Pulses  Treatment response  Adverse events | 10 | Primary SRNS: n=5  Secondary SRNS n=5  Mean age at onset: 6.2 ± 3.8 yrs  Mean time between diagnosis and start IV CPH treatment: 33±27 months (4-87)  Mean follow-up after CPH treatment: 26±9 months  Gender M/F: 6/4  Histology: all FSGS | IV CPH 500 mg/m^2^ monthly over 6 months  **Co-medication**: oral prednisone 60 mg/m^2^/d for 2 months, a.d. for 4 months, followed by 30 mg/m^2^  a.d. for 6 months, tapering and finally discontinued | **Remission status:**  Complete rem.: 7/10 within first 3 months  Partial rem.: 1/10  No response: 2/10  CPH-responders were treated earlier during disease course than non-responders: 25 (4-56) months vs. 69 (50-87) months  Normal eGFR during study  **Adverse effects:** None (no alopecia, cystitis, treatment-related infections, extensive nausea or vomiting) |
| **Mycophenolate Mofetil (MMF)** | | | | | | | |
| De Mello VR, 2010 [64]  Brazil | Mycophenolate mofetil in children with steroid/cyclophosphamide-resistant nephrotic syndrome | Retro-/prospective study | MMF  CsA+MMF  Treatment response  Relapses  CKD  Side effects | 52 | **Steroid- and CPH-resistant** nephrotic syndrome  **CsA+MMF Group:** n=34 (65.4%)  **MMF only Group:** n=18 (34.6%)  Age range: 2-17 yrs,  mean age: 12±4.7 yrs  Gender M/F: 30/22  Histology: MCD (19/52), FSGS (30/52), other (3/52)  Genetic screening: not performed  Time from diagnosis to MMF treatment start: Range 8-141 months | **CsA+MMF Group:**  Patient were treated according to CsA protocol. CsA was discontinued due to primary CsA resistance (8/34), secondary CsA resistance (6/34), nephrotoxicity (12/34), frequent and prolonged relapses (6/34), very high levels of cholesterol (2/34)  After CsA discontinuation, MMF was administered 500-600 mg/m^2^ twice daily  **MMF only Group:**  500-600 mg/m^2^ twice daily  **Pre-medication:**  Prednisolone, IV MP Pulses, CPH 2.5 mg/kg/day for 60-90 days  **Co-medication:**  ACE inhibitors, ARB | **Remission status**  CsA+MMF Group:  Complete remission: 7/34 (20.6%)  Partial remission: 13/34 (38.6%)  No response: 14/34 (41.2%)  MMF only Group:  Complete remission: 5/18 (27.8%)  Partial remission: 6/18 (33.3%)  No response: 7/18 (38.9%)  No statistical difference in the response between both groups  Relapses:  All with complete remission: 12/52 (23.1%): achieved remission within 6 months. On average 3 relapses in 7/12 children after discontinuation of steroid therapy  All with partial remission: 19/52 (36.5%): 6/19 developed relapses  Progression to CKD: 8/52 (4 of each group)  **Side effects:**  None 39/52 (75%)  Gastrontestinal complaints 11/52 (21%): nausea+abdominal pain 6, diarrhea+pain 2, pancreatitis 3 (1 patient died)  Severe infections (penumonia and peritonitis): 1/52 (1.9%)  Mild leukopenia and mild thrombocytopenia 1/52 (1.9%) |
| Li Z, 2010 [65]  China | Mycophenolate mofetil therapy for children with steroid-resistant nephrotic syndrome | Prospective study  Single center Changsha, China | MMF  Treatment response  Relapses  Adverse events | 24 | Primary steroid-resistant nephrotic syndrome: 8/24 (33.3%)  Secondary steroid resistance: 16/24 (66,7%)  Age: < 2 yrs  Age at onset: median age 1 year 7 month (range 8-24 months)  Gender M/F: 18/6  Histology: FSGS 5, IgMN 4, MesPGN 15  Genetic screening: not reported | Before MMF:  Prednisone 2 mg/kg/d for 8 weeks  **MMF** 25-30 mg/kg/day divided into 2 doses for 6-12 months  Prednisone dose was reduced stepwise by 5 mg/d every 4 weeks.  When remission was achieved at the end of month 6: MMF was reduced by 50% for 3 month and then stopped | Remission status at 2 months of MMF:  Complete remission: 9/24 (37.5%)  Partial remission: 7/24 (31.2%)  No response: 8/24 (33.3%)  Remission status at 4 months of MMF:  Complete remission: 13/24 (56.3%)  Partial remission: 6/24 (25%)  No response: 5/24 (20.8%)  **Remission status at 6 months of MMF:**  Complete remission: 15/24 (62.5%)  Partial remission: 6/24 (25%)  No response: 3/24 (12.5%)  Relapses:  7/15 (47%) within the first year after MMF discontinuation  **Side effects:**  Mild nausea and vomiting: 3/24  Mild diarrhea: 2/24  Mild leukopenia for 4 weeks: 3/24 |
| Montané B, 2003 [66]  USA | Novel therapy of focal glomerulosclerosis with mycophenolate and angiotensin blockade | Prospective study  Single center | MMF  MMF+RAAS  Proteinuria change  Clinical response | 9 | **Primary steroid resistance and failure to conventional treatment regimens**  Age at onset: range 2-15 yrs  Mean age: 9.2±5.4 yrs  Duration of treatment without responses before study treatment protocol: average of 5.6±6 yrs (oral steroids (9), IVMP (9), CPH or chlorambucil (9), CsA (7), Tac (2))  Gender M/F: 5/4  Histology: all FSGS  Genetic screening: not reported | Before MMF:  IV MP 15 mg/kg weekly for 4-8 weeks, oral prednisone was tapered  After 4 weeks of MP Pulses:  MMF with 250-500 mg/m^2^/day (max. 2g/day) for 24 months  **Co-medication:**  All with ACEi (enalapril 0.05-0.1 mg/kg/day or captopril 0.25-0.5 mg/kg/day) or ARB (candesartan 0.15-0.3 mhg/kg/day or losartan 0.5-2.0 mg/kg/day). | **Change in proteinuria (Up/c)**  Post MP Pulses: average decrease of -43% (95% CI -18% to -68%), 2/9 proteinuria reduction  6 months of MMF: decrease from baseline -72% (95% CI -58% to -86%), significantly improved from post-MP pulses: -29% decline, p<0.01)  Complete remission: 3/9 (33.3%)  Partial remission: 6/9 (66.7%)  12 months of MMF: proteinuria level was maintained  24 months of MMF: proteinuria level was maintained  Clinical response:  All resolved edema within 2 months  No. of hospitalizytion decreased from an average of 4 to 1 per year  Laborchemically response:  Increase of serum proteins to near normal values, serum albumin remained averaged less than 3g/dl at 24 months.  Lipid metabolism: significant improvement but nit normalization |
| Gargah T, 2011 [67]  Tunesia | Mycophenolate mofetil in treatment of childhood steroid-resistant nephrotic syndrome | Prospective study  Single center Tunis | MMF  Treatment response  Monotoring  Side effects | 6 | Primary steroid resistance: n=4  Secondary steroid resistance: n=2  Median age at start of MMF: 11 yrs (range 9-13).  Gender M/F: 4:2  Histology: FSGS 4/6, MCD 1/6, MesPGN 1/6  Genetic screening: not reported | MMF 600 mg/m^2^ twice daily, adjusted to maintain MPA levels at 2.5-5 μg/ml  + oral prednisolone 1 mg/kg/day  **Pre-medication:**  None: 2/6  CsA 3/6 (no response)  CPH 1/6 (no response) | **Remission status at 12 weeks:**  Complete: 1/6  Partial: 1/6  No: 4/6 (still after 24 weeks)  **Side effects:**  No episodes of bacterial or viral infections  No leukopenia  Minimal gastrointestinal complaints |
| Barletta GM, 2003 [68]  USA | Use of mycophenolate mofetil in steroid-dependent and –resistant nephrotic syndrome | Retrospective study | MMF  CsA+MMF  Maintain remission  Relapses | 5 | **Primary SRNS**  **CPH- and CsA-resistant SRNS**  MMF introduced as alternative treatment option to spare steroids and CsA | MMF initial dose 800 mg/m^2^/day, titrated up to 1200 mg/m^2^/day as WBC allowed.  MMF was given 8-12 weeks before attempting to wean steroids or CsA over the subseuqent onths  Co-medication:  H2 blockers  Pre-medication: steroids, CPH, CsA | Reduction of No. relapses pre-MMF vs. MMF therapy per 12 months:  2.85 (±0.4) vs. 1.07 (±0.3) (p<0.01).  Weaning off CsA and steroids 2/5  Reduction of CsA-dose: 1/5  Repeatedly Relapses on MMF+CsA 2/5 |
| Gellermann J, 2012 [69] | Sequential maintenance therapy with cyclosporine A and mycophenolate mofetil for sustained remission of childhood steroid-resistant nephrotic syndrome | Retrospective study | CsA  CsA + MMF  Maintaining remission  MMF side effects  MPA-AUC  Renal function | 23 | **Non-genetic, primary SRNS** (genetic and syndromal forms excluded) with complete remission to CsA+Pred.  Mean age at onset: 8.2 yrs (0.5-11)  Mean time to initial remission: 3.7 months  Mean time between MMF start and obtaining initial remission: 1.7 (0.3-5.8) years  Mean follow-up: 7.0 (1.7-16.5) yrs | **Induction**:  IV MP 500-750 mg/m2 for 3 days  CsA 5 mg/kg/d, according to trough levels 80-120 ng/ml.  Oral pred. tapered > 6 months  **Maintenance:**  MMF 600 mg/m^2^ twice daily (MPA trough levels 2-4.5 µg/ml)  Followed by reduction of CsA by 25-30% and further stepwise reduction to 50% of initial dose.  After 12 months combined MMF/low dose CsA: start with MMF monotherapy  **Co-treatment:**  Antihypertensive Treatment  Anti-proteinuric treatment | **Induction therapy:**  At start of study all patients were in SRNS remission (22 complete, 1 partial with immunoabsorption)  **Maintenance treatment:**  Sustained remission:  At the end of follow-up period, all patients were in remission either without therapy or with CsA monotherapy or with a combination of CsA+MMf or MMF alone  Relapses:  5/23 patients (21%) developed a total of 10 relapses  Renal function:  CsA nephrotoxicity 8/23 patients with increasing serum creatinine levels.  After conversion from CsA to MMF, eGFR increased significantly from 107 to 140 ml/min*1.73m2 after 12 months (n=16; p=0.001)  MPA-AUC Pharmacokinetic:  Mean dosage 1026 (510-1435) mg/day in 2 divided doses.  Mean MPA-AUC 70 (39-113) μg*h/ml.  Signigficant linear correlation of MMF dosage with AUC (r=0.609; p=0.016)  **Adverse effects (for MMF):**  Anemia 2/23  Abdominal pain and nausea, spontaneously resolving |
| **Combined immunosuppressive treatment strategies – 2nd line** | | | | | | | |
| Kim J, 2014  [70] USA | Second-Line Immunosuppressive Treatment of childhood nephrotic syndrome: A single-center experience | Retrospective study  Single center New York | Second line treatment  MMF  Tac  Rituximab  Combined treatment  Treatment response | 67 | SRNS  Mean age at diagnosis: 8.8±5.6 yrs  Mean duration of illness: 6.8±5.1 yrs  Gender M/F: 42/25  Histology: FSGS 54/67 (81%)  Genetic screening not reported | Second-line immunosuppressive therapies:  (if no response after 4-6 months or intolerance -> switch ton ext medication in the following order:   1. **MMF** 600 mg/m^2^ twice daily   N=10 (15%)   1. **Tacrolimu**s 0.1 mg/kg divided in 2 doses (target level 3-5 ng/ml) N=23 (34%) 2. **Rituximab** 375 mg/m^2^ per dose until B-cell depletion (1-2 doses) N=4 (6%) 3. **Simultanous usage of MMF/tacrolimus/prednisone**   **Co-medication:**  ACEi/ARB 44/67 (65%) | Response rates:  **MMF:**  Complete rem.: 4/10 (40%)  Partial rem.: 2/10 (20%)  No rem.: 4/10 (40%)  Mean duration of use: 18.1±13.5 months  **TAC:**  Complete rem.: 12/22 (54%)  Partial rem.: 5/22 (22%)  No rem.: 5/22 (22%)  Mean duration of use: 20.5± 19.3months  **CsA (**N=13, 19%)  Complete rem.: 4/13 (31%)  Partial rem.: 3/13 (23%)  No rem.: 6/13 (46%)  Mean duration of use: 29.0± 42.0months  **CPH (**N=4, 6%)  Complete rem.: 0/3  Partial rem.: 1/3 (33%)  No rem.: 2/3 (67%)  Mean duration of use: 3.0 ± 9.5 months  **Rituximab:**  Complete rem.: 1/4 (25%)  Partial rem.: 0  No rem.: 3/4 (75%)  Mild allergic reactions: 2/4 (50%)  Simultanous usage: n=3  Complete rem: 2/3 (67%) |
| **Sirolimus** | | | | | | | |
| Liern M, 2012 [71]  Argentina | Use of sirolimus in patients with primary steroid resistant nephrotic syndrome | Prospective, interventional, non-randomized study | Sirolimus  Treatment response  Drug monitoring  Adverse events | 13 | **Primary steroid resistance and resistant to CNI** (CsA, tacrolimus) **and CPH.**  Mean age: 10 (8-18 yrs)  Follow-up: 26 months  Histology: all primary FSGS  Genetic screening: not performed | **Sirolimus** 3.6 mg/m^2^ once a day (1-5 mg/m^2^/day, maximum dose 5 mg/day; blood concentrations 7-10 ng/ml) for 12 months in responsive patients  **Co-medication:**  Enalapril 0.1-0.3 mg/kg/day and Losartan 0.8-1.5 mhg/kg/day since start of study | **Remission status during study:**  Complete remission: 5/13 (38.5%)  Partial remission: 4/13 (30.8%), of those: 2/4 had relapses  No remission: 4/13  **Sirolimus dosages and blood levels:**  Patients with complete response: 3.1 mg/m2/d; 7.7 ng/ml  Patients with partial response: 3.5 mg/m2/day; 8.5 ng/ml)  Patients with no response: 5.8 mg/m2/day; 9 ng/ml)  **Adverse events:**  Anemia 3/13, acute diarrhea 2/13, oral ulcers: 3/13 |
| **Vincristine** | | | | | | | |
| Almeida MP, 1993 [72]  Portugal | Vincristine in steroid-resistant nephrotic syndrome | Retrospective study | Vincristine  Response  Renal function  Side effects | 7 | Primary SRNS: n=5  Secondary SRNS n=2  Resistance to:  CPH-resistance 5/7, levamisole 1, CsA 1  Age: 2-15 years (varied 8 months to 11 years)  Mean time from onset to VCR start: 39 months  Mean follow-up after VCR: 50 months  Histology: all FSGS | Vincristine IV 1.5 mg/m^2^ weekly for 8 weeks  +  oral prednisone 60 mg/m2/day for 4 weeks, then gradually tapered in an alternate-day regime | **Remission status:**  Complete remission: 2/7, stable over 2 years, then relapse (SS relapse), mean time to response: 4 weeks  No remission: 5/7  Renal function at last follow-up:  1/7 ESRD, renal Tx  **Side effects:**  Alopecia 1/7, Peritionitis 1/7, dental abscess 1/7, otitis media+pneumomnia 3/7. |
| Goonasekera CDA, 1997 [73]  UK | Vincristine and focal segmental sclerosis: do we need a multicentre trial | Retrospective study | Vincristine  Response | 8  Report of 2 cases | Primary SRNS  CPH-resistant SRNS | Vincristine IV 1.5 mg/m^2^ weekly for 8 weeks | **Remission status:**  Complete rem.: 2/8,  1 with relapses, both normal renal function |
| Krishnan RG, 2006 [74]  UK | Is there a role for vincristine in nephrotic syndrome | Retrospective data analysis | Vincristine  Response  Side effects | 9 | SRNS  Multidrug-resistant  Median age 5.3 (1.2-14.4) years  Mean follow-up after VCR: 2 years  Histology: FSGS 8/9 | Vincristine IV 1.5 mg/m^2^ weekly for 8 weeks | **Remission status:**  Complete remission: 2/9  **Side effects**: jaw pain (requiring carbamazepine), constipation, seizure, foot-drop for 6 months |
| Thalgahagoda S, 2017 [75]  Sri Lanka | Pulsed Vincristine therapy in steroid-resistant nephrotic syndrome | Retrospective study  Single center | Vincristine  Response  Relapses  Side effects | 54 | SRNS  CPH-resistant SRNS (6 weeks as evaluation period)  Median age 6.1 (3.5-11.6 years)  Gender M/F: 39/15  Histopathology: FSGS 32 (59%), MCN and mesangial proliferation 22 (41%) | Vincristine IV 1.5 mg/m^2^ weekly for 8 weeks  Co-medication: oral prednisone with a tapering regimen | **Remission status**:  Complete remission 21/54 (39%)  Partail remission: 7/54 (13%)  No response: 26/54 (48%)  **Sustained remission** at 6, 12, 24 and 60 months:  15/54 (28%), 11 (20%), 9 (17%), 7 (13%)  Relapses in responders:  6/21 (29%) within 6 month  **Side effects:**  Vomiting 7/54, Weight loss 4/54, Diarrhea 6/54,  Abdominal distension and cramps 21/54, mouth ulcers 3/54, headache 4/54, hair loss 38/54, constipation 13/54, loss of appetite 1/54, change in sense of taste 17/54, numbness and tingling in the hands and feet 8/54, reversible bilateral ptosis 3/54.  No discontinuation of treatmen |
| **Mizoribine** | | | | | | | |
| Saito T, 2017 [76]  Japan | Mizoribine therapy combined with steroids and mizoribine blood concentration monitoring for idiopathic membranous nephropathy with steroid-resistant nephrotic syndrome | Prospective, randomized trial  multicentre | Mizoribine  Response  Adolscents/adults  pharmakokinetics | 51 | Adolescent and mainly adult SRNS patients  Age 16-75 years  Group 1 (once a day): n=26  Median age: 60 (35-70) yrs  Gender M/F: 15/4  Group 2 (3-times-a-day): n=25  Median age: 60 (43-74 yrs)  Gender M/F: 14/4  Histology: Membranous Nephropathy as inclusion criterium | **Group 1 (once a day):**  Mizoribine (MZR) orally once a day after breakfast at 150 mg/day  + Prednisone 40 mg/day, tapered gradually to <10 mg/day by 48 weeks  **Group 2 (3-times-a-day):**  MZR 50 mg 3-times a day after meals  + Prednisone 40 mg/day, tapered gradually to <10 mg/day by 48 weeks  **Co-medication:** antihypertensive f´drugs, antidyslipidemic, antiplatelet and anticoagulants drugs | **Responses**  **Group 1 (once a day):**  At 1yr: complete remission without relapse: 7/19 (36.8%), 7/19 (36.8%) with partial remission, withdrawn 5/19  At 2yr: complete remission: 10/19 (52.6%), partial remission: 2/19, no response 1/19, withdrawn 6/19  **Group 2 (3-times-a-day):**  At 1yr: complete remission without relapse: 9/18 (50%), partial remission: 7/18 (39%), no response 2/18  At 2yr: complete remission 7/18, partial remission 8/18, no response 2/18, withdrawn 1/18  Mizoribine pharmacokinetics (see publication text for details) |
| **Antibody Treatments** | | | | | | | |
| **Anti-CD20-Antibodies** | | | | | | | |
| **Rituximab** | | | | | | | |
| Bagga A, 2007 [77]  India | Rituximab in patients with steroid-resistant nephrotic syndrome  NEJM | Retrospective case study | Rituximab  Response  Relapses  Multidrug-resistance | 5 | **Primary steroid resistance**: n=3  **Secondary steroid resistance**: n=2  **Multi-drug-resistant**  Age range 2.8-16 years  Histology: FSGS 3/5, MCN 2/5  Genetic screening: not reported | **Rituximab** 375 mg/m^2^ BSA once weekly for 4 weeks  + Cotrimoxazol prophylaxis for 6 months  **Co-medication:**  CNI, alternate-day prednisolone, enalapril 0.2-0.3 mg/kg/day  **Pre-medications:**  multiple medications for 1.8-14.2 years with periods of partial/complete remission (IV MP, CNI for at least 6 months, alcylating agents, MMF) | **Remission status:**  Complete remission: 4/5 at a median interval of 4 (2-8) weeks after last rituximab dose)  Partial remission: 1/5.  **Status during follow-up:**  Maintaining complete remission: 3/5 (despite tapering steroids and CNIs), duration min. 14, max. 58 weeks)  Relapse: 1/5 after 6 month. Treated with prednisone -> partial remission  Partial remission: 2/5 |
| Gulati A, 2010 [78]  India, USA | Efficacy and safety of treatment with rituximab for difficult steroid-resistant and –dependent nephrotic syndrome: multicentric report  CJASN | Retrospective study  multicentre | Rituximab  Response  eGFR  Multidrug-resistance  Side effects | 33 | Primary SRNS n=24  Secondary SRNS n=9  **Resistant to standard treatmen**t (IV steroids, Oral/IV CPH, CNI, levamisolem MMF, vincristine)  Mean age at onset: 6.3±4.8 yrs  Mean age at RTX treatment: 12.7±2.9 yrs  Mean duration of NS before RTX: 6.4±4.7 years (range 1-15 yrs)  Mean duration of CNI therapy before RTX: 22.7±17.1 (4 to 56) months  CNI toxicity: 11/33  Gender m/F: 17/16  Histology: MCD 17/33, FSGS, 16/33  Genetic screening: 17/33 were screened for *NPHS1* and *NPHS2* -> negative | **Rituximab** 375 mg/m^2^ weekly for 4 doses  **Co-medication:**  **Oral prednisone** 1.5 mg/kg a.d. for 2 weeks, 1 mg/kg for 4 weeks, 0.75 mg/kg for 4 weeks, then 0.5 mg/kg/d.  **Enalapril** 0.5 mg/kg/day in all patients.  **CsA/Tac (n=4);** if possible dosis reduction by 50% at 3 months  **MMF (n=2)** | **Remission status at 6 month after rituximab:**  Complete rem.: 9/33 (27.2%)  Partial rem.: 7/33 (21.2%)  No rem.: 17/33 (51.5%)  Median time to response: 32 (6 to 60) days after the last dose of RTX  **Remission status at last follow-up** (after 21.5±11.5 months):  Sustained complete rem.: 7/33  Partial remission: 8/33  No remission: 18/33, of those 9/18 eGFR < 60 ml/min*1.73 m2 at 12 months, incl. 5/9 with CKD stage 5.  **Side effects:**  Infusion reactions: 3/33  No serious infections |
| Nakayama M, 2008 [79] | Rituximab for refractory focal segmental glomerulosclerosis PNEP | Case reports | Rituximab  Response  Relapses  Multidrug-resistance | 2 | **Patient 1:**  Secondary SRNS  Age at onset: 8 yrs  Age at RTX: 10 yrs  Non-response to CsA (PRES, AKI), IV MP + plasmapheresis  Severe steroid side effects, mizoribine  Histology: 1st DMP, 2nd FSGS  Genetic screening: not reported  **Patient 2:**  Secondary SRNS  Age at onset: 11 yrs  Age at RTX: 12 yrs  Non-response to IV MP, CsA, steroids; IV MP+plasmapheresis  Severe steroid side effects, | Rituximab 375 mg/m^2^ single dose  Co-medication:  Low-dose predn. On alternate days, CsA (Pat.2) | **Patient 1:**  At 1 month: Partial remission  Within 8 months: Complete remission  Last follow-up at 16 months: maintaining remission, normal renal function  **Patient 2:**  At 2 month: Partial remission  Within 5 months: Complete remission  8 months: relapse, 2nd course of RTX  Normal renal function |
| Sinha A, 2015 [80]  India | Efficacy and safety of rituximab in children with difficult-to-treat nephrotic syndrome | Retrospective study | Rituximab  Response  Multidrug-resistance  ESRD progression  ESRD risk factors  Adverse events | 58 | **Primary steroid resistance**: n=32 (55%)  **Secondary steroid resistance**: n=26 (45%)  Age 1-18 years  Age at onset: 44.9±42.5 months  Age at rituximab: 107.8±59.1 months  Indications for rituximab:  Primary CNI-non-response: 34/58 (59%)  Delayed CNI-non-response: 24/58 (41%)  Gender M/F: 35/23  Histology: FSGS 41 (70%). MCD 17 (29%)  Genetic screening: 14/58 patients  Comp- het. NPHS2 1/14  Het. WT1 1/14 | **Rituximab** 375 mg/m^2^ per dose once weekly for 2-4 doses  4 doses: 39/58  3 doses: 10/58  2 doses: 9/58  **Co-medication:**  **Pre-medication:**  CPH oral/IV: 20 (35%)/16 (28%)  MMF 20 (35%) CsA/Tac/both: 16 (28%)/ 26 (45%)/ 13 (22%)  Use of ≥ 2 steroid sparing agents: 35 /60%) | **Remission status:**  Complete rem. 7/58 (12%)  Partial rem. 10/58 (17%)  No remission: 41/58 (71%)  Mean time to CR: 2.0±1.4 (IQR 1-2) months after rituximab therapy.  Median duration of CR: 9 (7-14) months  Genetic patients (4): Non-response in all  Remission: FSGS 8/41; MCN 9/17 (p=0.011)  **Progression to ESRD:**  1/7 complete responders  2/10 partial responders  20/41 (49%) non-responders progressed to CKD4-5  (p=0.016)  Proportion of ESKD non-response vs. responders (CR/PR)  At 1 yr: 18.5% vs. 0%/0%  At 2yrs: 32.8% vs. 0%/10%  At 5yrs: 79% vs. 20%/20%  **Predictors:**  FSGS predictor for non-response to RTX (OR 11.1; 95% CI 1.3, 99.8; p=0.028)  Non-response to RTX indenpendently predicted progression to CKD 4-5 (HR 9.97; 95% CI 1.2, 99.6; p=0.035)  **Adverse effects:**  Infusion-related reactions incl. Urticaria during 3 infusions:  Fever, chills, throat pain and hypertension in 1/58 each  Transient synovitis and delayed drug eruptions at 2-7 days 3/58  Other: transient leukopenia, hematuria, tachypnea, peritonitis, varicella and malaria in 1/58 each. |
| Kamei K, 2014 [81]  Japan | Rituximab treatment combined with methylprednisolone pulse therapy and immunosuppressants for childhood steroid-resistant nephrotic syndrome | Retrospective study | Rituximab  Response  Renal function  Multidrug-resistance  Adverse events | 10 | **CNI-resistant SRNS**  **Multi-drug-resistant SRNS**  (CNI, CPH, mizoribine, MMF, IV MP, plasma exchange 4/10. Lipid apheresis 1/10).  Impaired renal function 3/10 at RTX start  Age: 2-14 yrs  Histology: FSGS 7/10, MCN 2/10, DMP 1/10  Genetic screening: 3/10 patients in NPHS2 and WT1 -> negative | **Rituximab** 375 mg/m^2^ 1-4 doses (max. 500 mg)  (1 dose: 6/10; 2 doses: 2/10; 4 doses: 2/10)  +  Followed by  **IV MP Pulses** 30 mg/kg/day for 3 days) once every 2-4 weeks until remission  **Co-medication**: CNI, alkylating agent, prednisone, MMF | **Remission status:**  Complete remission: 7/10, 3/7 with relapses  Partial remission: 1/10  No remission: 2/10  Renal function:  7/7 responders preserved normal renal function  2/2 non-responsive patients progressed to ESRD  1 partial responder: mild renal insufficiency  **Adverse events:**  Infusions reactions 11/27 performed infusions (41%) (cough n=4, respiratory disturbances n=3, hypoxemia n=3, abdominal pain n=2, rash n=2, wheezing n=1, sore throat n=1, nausea n=1, hypertension n=1)  2 patients with late-onset adverse events: Agranulocytosis 56 days after 5th RTX infusion + upper respiratory infection n=1, severe pneumonis (H1N1) requiring ventilation n=1. |
| Ito S, 2013 [82]  Japan | Survey of rituximab treatment for childhood-onset refractory nephrotic syndrome | Retrospective study (survey)  multicentre | Rituximab  Response  Renal function  Multidrug-resistance  Adverse events | 19 | Primary SRNS n=8  Secondary SRNS n=11  Multi-drug resistant SRNS  Adverse affects of steroids and CsA  Histology: FSGS 11/19, MCN 8/19  Genetic screening: 3/19 in WT1 and NPHS2 -> negative  WT1: 1/3 | Rituximab 375 mg/m^2^ as 1-2 doses (single dose: 85%)  Co-medication: oral predn., CsA, MMF, mizoribine | **Remission status:**  Complete remission: 6/19 (32%)  Partial response: 6/19 (32%)  No reponse: 7/19 (38%), 1/7 with WT1-mutation  Median time to response (CR/PR) after RTX:  6 (1-12) months  **Adverse events:**  Infusion reactions: Sore throat n=15, Wheezing/cough n=7, dyspnea 7, fever 4, skin rash 3, nausea/vomiting 2, bradycardia 2, hypertension 2, hypotension 1, tachycardia 1, nasal stiffness 1, leg pain 1.  Late adverse events:  Sepsis n=1, Granulocytopenia n=2, mild liver failure n=1, fever n=1 |
| Fujinaga S, 2018 [83] Japan | Long-term outcomes after early treatment with rituximab for Japanese children with cyclosporine- and steroid-resistant nephrotic syndrome | Retrospective study | Rituximab  Response  Relapses  Multidrug-resistance  Adverse events | 6 | Primary steroid resistance, CsA-resistant SRNS after > 8 weeks despite the use of CsA (4-7 mg/kg/d in 2 divided doses, max. 150 mg/day) combined with ≥ 2 courses of IVMP (20 mg/kg/day for 3 days, max. 600 mg/day)  Median age at diagnosis: 3.8 yrs  Median age at RTX treatment: 4.2 yrs  Median follow-up: 5.1 yrs  Gender M/F: 3/3  Histology: not reported  Genetic screening: not reported | Rituximab 375 mg/m^2^ (max. 500 mg),  followed by re-treatment with IVMP (n=4) and/or prednisolone 2 mg/kg/day (n=4).  Rituximab was repeated (followed by IVMP/Prednisolone) when marked decrease in proteinuria was achieved.  Then tapering steroids and discontinuing within 12 months.  After that, CsA as maintenance treatment was discontinued, MMF (maintaining blood levels of 2-5 μg/ml) was initiated in case of relapse.  In case of relapses despite maintenance treatment with CsA or MMF, RTX was re-administered. | **Remission status:**  Complete remission 6/6 after a median of 158 days, discontinuation of steroids at a median of 7 months without relapses  **Relapses:**  5/6 after median of 486 days, in total 17 relapses in 5 patients during follow-up (median 5.1 yrs).  **Adverse events:**  Severe neutropenia (<500/mm^3^) 2/6  Hypogammaglobulinemia (<500 mg/dl) requiring IVIG infusions 4/6, 1/6 required IVIG despite re-emergence of B-cells  No severe bacterial infections |
| Sun Li, 2014 [84]  China | Efficacy of rituximab therapy in children with refractory nephrotic syndrome: a prospective observationsal study in Shanghai | Prospective study  Single center Shanghai | Rituximab  Response  Renal function  Multidrug-resistance  Side effects  B-cell monitoring | 3 | Complete study: N=12  SDNS/FRNS: n=9  **SRNS n=3**  Age at onset: 1.6-8.9 yrs  Gender M/F 9/3  Histology: MCD 7/12, FSGS 3/12, prol. GN 1/12, no biopsy 1/12  SRNS: MCD 2/3, FSGS 1/3  Genetic screening: performed in all 3 SRNS patients including NPHS1, NPHS2, WT1, PLCE1, CD2AP, TRPC6, APOL1, INF2, MYO1E, MYH9 -> negative | Rituximab 375 mg/m^2^  (max. 500 mg) as single dose,  repeated in 2/3 SRNS patients  **Co-medication:**  Oral steroids, CsA, Tac, MMF | **Remission status of SRNS patients:**  Complete remission: 2/3  Partial remission: 0/3  No remission 1/3  B-Cell-depletion: in all after 2 weeks  B-cell recovery: Average after 4.4 months (1 patient after 2 doses, 5.5 months, 1 patient 11 month)  **Side effects:**  Acute (during administration): skin rashes 3/12, hypotension 1/12, fever 1/12  Persistent deficiency of IgG 1/12 with 3 infections during follow-up (upper respiratory infections 2, pneumomia 1) |
| Zachwieja J and Silska-Dittmar M, 2017 [85]  Poland | Multicenter analysis oft he efficacy and safety of a non-standard immunosuppressive therapy with rituximab in children with steroid-resistant nephrotic syndrome | Retrospective study  Multicenter (7 polish centers) | Rituximab  Response  Reduction of immunosuppressives  Multidrug-resistance  B-cell monitoring  Adverse events | 30 | **Primary steroid resistant NS**  Treatment with 2-3 immunosuppressants before, for a minimum of 2 years  Mean age: 14.3±0.6 yrs.  Histology: FSGS 14/30, MCD 4/30, DMS 7/30, MesGN 4/30, Membr.GN 1/30  Genetic screening: not reported | Rituximab (RTX) single dose 375 mg/m^2^  **Co-Medication at start of RTX::**  CsA 23/30 (77%) Prednisone 18/30 (60%) | **Remission status BEFORE/AFTER RTX (6 months):**  Complete remission: 13/30 (43%)/ 16/30 (53%)  Partial remission: 8/30 (26%)/ 12/30 (40%)  No remission: 9/30 (31%)/ 2/30 (7%)  **Reduction of immunosuppressants:**  At start of RTX/ 6 months after RTX:  CsA: 23/30 (77%)/ 15/30 (50%)  Predn.: 18/30 (60%)/ 8/30 (27%)  B-cell-depletion: complete 25/30, significantly lower 5%30  B-cell-recovery: > 6 months in 25/30  **Side effects:**  Pneumonia (M.pneumoniae, PcP) 2/30 |
| Kari JA, 2011 [86]  Saudi Arabia | Rituximab for refractory cases of childhood nephrotic syndrome | Retrospective study  Single center | Rituximab  Response  Side effects | 4 | **Primary steroid resistance**: n=2  **Secondary steroid resistance**: n=2  **Multi-drug resistant** (IVMP, CPH, CsA, Tac, MMF)  Median age 10 (8-11) years.  Gender M/F: 2/2  Histology: MCD1/4, FSGS 2/4, IgMN 1/4  Genetic Screening: NPHS2 negative in all | Rituximab (RTX) single dose 375 mg/m^2^  Co-medication:  ACE inhibitors 4/4 | **Remission status:**  Partial remission: 1/4, relapse after 4 months despite depleted B-cell count  No remission ¾  **Side effects after 6 months follow-up:**  Infection (peritonitis) 1/4 |
| Basu B, 2015 [87]  India | Mycophenolate Mofetil following rituximab in children with steroid-resistant nephrotic syndrome | Retrospective study | Rituximab  RTX+MMF  Maintenance treatment  Reponse  Relapses | 24 | **Multidrug-resistant SRNS**  (Patients resistant to standard treatment)  Histology:  MCD 13/24 (54%), FSGS 11/24 (46%). | Rituximab 375 mg/m^2^  weekly 2-4 doses depending on B-cell level.  After 3 month:  Second course of rituximab (if >5 B cells/mm^3^)  +  maintenance therapy:  MMF 1200 mg/m^2^/day  N=15  Co-medication:  Enalapril 0.5 mg/kg/day in all patients  Other oral immunosuppressive drugs were tapered down or discontinued gradually | **Remission status** **after 1st course of rituximab**:  Complete rem.: 5/24 (21%), of those 4/5 relapsed again at a median interval of 53 (46-72) days.  Partial remission: 11/24 (46%)  No response: 8/24 (33%)  **Remission status after 6 months (with MMF**): n=15  Complete rem.: 10/15 (67%)  Partial remission: 5/15 (33%)  **Remission status after 6 months (RTX+MMF-responder**):  n=10  Sustained complete rem.: 6/10,  Relapse: 4/10  **Remission status after 24 months (all):**  Complete rem.: 6/24 (25%)  Partial rem.: 10/24 (42%) |
| **Ofatumumab** | | | | | | | |
| Vivarelli M, 2017 [88]  Italy | Ofatumumab in tweo pediatric nephrotic syndrome patients allergic to rituximab | Case report  retrospectively | Ofatumumab  Case  Induction of remission  RTX intolerance | 1 | Patient 1 (14 year old boy) SDNS  Age at onset: 2 years  **Patient 2 (3 year old boy) SRNS**  Age at disease onset: 18 months, primary steroid resistance, complete remission with 3 MP Pulses following CsA 5 mg/kg/d. When steroids discontinued after 6 months -> relapse 2 months later -> remission achieved with prednisone, tacrolimus (0.07 mg/kg/d) was added 6 months later -> rituximab in order to discontinue prednisone -> immediate severe allergic reaction despite pre-medication  Follow-up: > 12 months | Ofatumumab infusion  750 mg/1.73m^2^ | **Patient 2 with SRNS:**  Induction of remission and maintaining remission during follow-up period (>12 months) despite discontinuation of steroids with tacrolimus.  B cells reappeared (>5%) 7 months after the infusion. |
| Basu B, 2014 [89]  India | Ofatumumab for Rituximab-Resistant Nephrotic Syndrome | Case reports retrospectively | Ofatumumab  Multidrug-resistance  RTX-resistant  Response  Side effects | 5 | **Primary steroid resistance: n=3**  **Secondary steroid resistance: n=2**  **Resistant to multiple immunosuppressive medication, including rituximab-resistant SRNS**  Histology: FSGS 3/5, MCD 2/5 | Ofatumumab  1st week: 300 mg/1.73m^2^ BSA per week  2nd-6th week: 2000 mg/1.73m^2^ BSA per week  (Total of 6 doses)  **Co-medication:**  Ramipril  **Pre-medication**: Steroids, IV CPH Pulses, Tac, CsA rituximab, MMF, galactose | **Remission status:**  Complete remission: 5/5  3/5: after 4^th^ dose, 2/5 after 6^th^ dose  **Status during follow-up:**  Complete remission 4/5 (min. 25 wk to 50 wk)  Partial remission: 1/5 (1st relapse after week 8, 2nd relapse at week 25).  **Side effects:**  1 transient mild infusion reaction |
| **Anti-TGFβ-Antibodies - Fresolimumab** | | | | | | | |
| Trachtman H, 2011 [90]  USA | A phase 1, single-dose study of fresolimumab, an anti-TGFβ-antibody, in treatment-resistant primary focal segmental glomerulosclerosis |  | FSGS  Phase-1 study  Adults  Treatment resistance | 16 | Adults, no data in children  Primary, treatment-resistant FSGS with a minimum of eGFR of 25 ml/min*1.73m^2^  Mean age of patients: 37±12 years  Mean FSGS duration 3.0±2.1 years  Follow-up after fresolimumab: 112 days | Fresolimumab 1 of 4 single-dose levels (up to 4 mg/kg)  4 at each dose level:  1 mg/kg; 2mg/kg; 3 mg/kg; 4 mg/kg  Co-medication: 15/16 (94%)  RAAS 14/16  12/16 lipid lowering agent; 11/16 diuretics, 4 aspirin | Fluctuating proteinuria during study: median change from baseline was -1.2mg/mg,  Slight decline in eGFR: median decline baseline to final 5.85 ml/min*1.73 m^2^.  Half-life fresolimumab: 14 days  Well tolerated:  Infusion reaction; 1/16  Pustular rash 2/16  ... |
| **Obinutuzumab** | | | | | | | |
| No studies available |  |  |  |  |  |  |  |
| **Abatacept** | | | | | | | |
| Yu CC, 2013 [91] | Abatacept in B7-1-positive proteinuric disease | Prospective study | Abatacept  More case reports in post-transplant fsgs recurrence | 5,  1 SRNS native kidneys | FSGS (4 with recurrent FSGS after transplantation and one with primary FSGS)  Treatment-resistant (as well CNI, RTX, plasmapheresis...)  B7-1-staining of podocytes positive in all of 5 patients  Age: 7, 14, 19, 27, 27 yrs | Abatacept 10 mg/kg, 1-2 doses FSGS recurrence  Patient with native FSGS: day1, day, 15, than monthly  Co-medications: steroids | Abatacept (cytotoxic T-lymphocyte-associated antigen 4-immunoglobulin fusion protein CTLA-4-Ig) is a costimulatory inhibitor that targets B7-1 (CD80). B7-1 is induced in podocytes in various animal models of proteinuria.  Podocyte B7-1 expression is not evident in normal kidney podocytes but is found in patiengts with certain glomerular diseases, e.g. found in biopsies with proteinuric kidney diseases, especially recurrent FSGS.  FSGS in native kidney: induction of clinical remission fort he 1st time in more than 1 year. |
| **IL-2** | | | | | | | |
| Bonnani A, 2015 [92]  Canada, Italy | A Pilot Study of IL2 in drug-resistant idiopathic nephrotic syndrome | Prospective pilot study | IL2  Tregs  Pilot study | 5 | Treatment-resistant NS (i.e.steroids, CNI, MMF, rituximab)  Age: 11-17 years  Gender: all boys  Histology: FSGS 3, MCD 2 | 6 monthly cycles of low-dose Il2 (1x10^6^U/m^2^  first month, 1.5x10^6^U/m^2^ following months)  Co-medication:  ACEi, ARB, low dose prednisone | Cytokine Il2 stimulates Tregs maturation from T progenitor and is currently consideres a potential drug in clinical conditions in which high Tregs may be beneficial tot he outcome.  Treg infusion reverts proteinuria and reduces renal lesions in most animal models of nephrotic syndrome. Il2 up-regulates Tregs.  Treg levels increased by approx. 10% with differnecs in rapidity of response from 30 days up to 150 days.  Proteinuria did not change during study period.  Adverse events:  1/5 with asthma attack |
| **Non-immunosuppressive Treatments - Galactose** | | | | | | | |
| Sgambat K, 2013 [93]  USA | Effect of galactose on glomerular permeability and proteinuria in steroid-resistant nephrotic syndrome | Preospective pilot clinical trial | Galactose  Focal sclerosis permeability factor /FSPF) | 7 | Patients with idiopathic SRNS and positive circulating focal sclerosis permeability factor (FSPF) activity (>0.5)  Native kidneys N=5  Post-Tx recurrence: n=2  Age range: 2-21 yrs, mean age 7.7 ±4.0 yrs  Time from diagnosis to study enrolment: 28.1±23.0 months  Gender M/F: 4/3  Histology: MCD 1/5, FSGS 4/5, Post-Tx FSGS 2/2  Genetic screening: performed 6/7 in NPHS1, NPHS2, WT1 -> negative | Oral Galactose (0.2 mg/kg/dose twice daily, max. dose 30g/day) for 16 weeks  Co-medication:  Unchanged Immunosuppressants (CsA, Tac, MMF), ACE inhibitors, ARB, plasmapheresis regimen in patients with post-Tx recurrence | **FSPF activity after 16 weeks:**  Significantly decreased from 0.69±0.11 (Range 0.58-0.91) to 0.35±0.21 (range 0.00-0.58) (p=0.009).  4/7 patients: FSPF activity became negative  2 patients with post-Tx FSGS had significantly greater improvement of FSPF activity (p=0.006).  **Proteinuria reduction after 16 weeks:**  Unchanged in the study group, all remained in “no remission” status.  No side effects, well tolerated, no hyperglycemia. |

**Table S4: Studies - Extracorporal Treatments in SRNS – Pre-Transplant**

| **1^st^ author,**  **year,**  **country of origin**  **[Ref.]** | **Title of Publication** | **Study design** | **Key words** | **N** | **Population Characteristics** | **Treatment** | **Outcomes** |
| --- | --- | --- | --- | --- | --- | --- | --- |
| **Plasmapheresis/ plasma exchange in FSGS – native kidneys** | | | | | | | |
| Imaizumi T, 2007 [94]  Japan | Efficacy of steroid pulse, plasmapheresis, and mizoribine in a patient with focal segmental glomerulosclerosis | Case report | Plasmapheresis  FSGS  SRNS | 1 | 4 year old boy with FSGS complicated by leukoencephalopathy, induced by CsA | PP, plasma volume of 50 ml/kg BW was exchanged at each session.  6 times over 2 weeks.  Co-med. IVMP, mizoribine | Following PP proteinuria gradually decreased over the next 3 months to less than 1.0g/day. |
| Oliveira L, 2007 [95] | A case report of plasmapheresis and cyclophosphamide for steroid resistant focal segmental glomerulosclerosis: recovery of renal function after five months on Dialysis | Case report | Plasmapheresis  FSGS  SRNS | 1 | Adult, 74 years old  SRNS  FSGS  Rapid progression to ESRD  HD for 5 months espite high dose prednisone therapy. | PE and low dose oral CPH | Prompt return of urine output and renal recovery, r  2 years later: remains off dialysis with stable renal function (eGFR 40 ml/min) |
| Paglialonga F, 2015 [96] | Indications, technique, and outcome of therapeutic apheresis in European pediatric nephrology units | Registry analysis | Plasmapheresis | 13 | **FSGS in native kidneys: n=1 (PE)**  Preemptive PP pre-Tx n=2 (PE)  Recurrent post-Tx FSGS: n=8 (7 PE, 1 IA, 1DFPP)  Recurrent post-Tx congenital NS n=1 (PE) | No. sessions median 17 (10-38) over a median period of 25 days (14-105)  **Various IS co-medication**:  RTX, MMF, Tac, CsA | 7/9 post-Tx recurrence patients underwent PE  Median urinary protein/creatinine ratio (uPr/uCr) decreased from 9.9 mg/mg (1.8-18) to 1.1 mg/mg (0.2-1.7; p<0.005)  Complete remission 1/7  Partial sponse 6/7  2/2 IA and DFPP. Proteinuria disappeared in the first year 1 of 2, decreased to 1.3 mg/mg in the 2nd case. |
| Skalova S, 2010 [97] | Plasmapheresis-induced clinical improvement in a patient with steroid resistant nephrotioc syndrome due to podocin (NPHS2) mutation | Case report | SRNS  NPHS2  Plasmapheresis | 1 | SRNS was diagnosed within 1st year of life (5th month of life).  Renal Bx at 4.5 yrs: FSGS  4-9 yrs: no progression of disease, proteinuria 1g/24h, no therapy.  At 9 yrs: enalapril 0.15-0.2 mg/kg/day  14 ysr: 2nd biopsy, initiation of CsA, MP, enalapril (0.3 mg/kg/day) and losartan 1mg/kg/day.  By the age of 15 yrs: Proteinuria 9g/24h. albumin 19.6 g/l despite IS treatment with combination of CsA, IVMP, enalapril and losartan.  Then diagnosis of comp.het. NPHS2 (p.R138Q and p.V290M) mutation. | PE 10 sessions  Plasma volume was exchanged at a rate of 1000 ml/hr.  4 sessions every day, 6 sessions every other day  Pre-treatment: steroids, levamisol, CPH, IV MP, CsA | After PE: clinical improvement (proteinuria 3g/24h, albumin 30g/l), MP discontinued, lasting for 3 years. CsA discontinued 2 month after PE.  6 yr after PE: ESRD, hemodialysis |
| Vecsei AKW, 2001 [98]  Austria | Plasmapheresis-induced remission in otherwise therapy-resistant FSGS | Case report | FSGS  SRNS  plasmapheresis | 1 | SRNS, multidrug-resistant  FSGS  8 year old boy | Plasma exchange (PE): daily sessions, FFP at a dose of 60 ml/kg to replace the patients plasma at afiltration rate of 1ml/kg*BW*min,  later tapered to one weekly sessions  **Pre-treatment:**  Oral pred, IV MP Pulses, CsA (conventional dose and high-dose), MMF | After 5 sessions PE:  Normal serum protein levels, proteinuria < 0.5g/m^2^/day, previously risen creatinine stabilized, blood pressure normalized.  PE sessions were tapered to once weekly, steroids and MMF was withdrawn.  12 months after PE start (and 23 month after disease onset):  Good clinical condition  Partial remission (proteinuria <0.5mg/m^2^/day, normal serum protein levels, crea 1.3 mg/dl)  Maintenance immunosuppression with CsA (trough level 150 ng/ml)  PE every 2 weeks using 5% human albumin as exchange fluid. |
| **Preemptive Plasmapheresis before Tx** | | | | | | | |
| Verghese PS, 2017 [99]  USA | The effect of pre-transplant plasmapheresis in the prevention of recurrent FSGS | Retrospective study | end-stage renal disease  focal segmental glomerulosclerosis  kidney transplant  pediatrics, pediatric kidney transplantation  recurrence | 56 | All FSGS  Tx before 2006: N=31  Age: 10.4±5.4 yrs  Tx after 2006: N=25  Age: 13.2±4.5 yrs  Genetic screening: NPHS2 | **Tx before 2006:**  No PE  **Tx after 2006:**  1 or 3 pre- and 5 post-Tx 1.0 plasma volume/exchange every other day  PE with FFP  All had lymphocyte depleting induction immunosuppression  **Cave**: different center immunosuppression protocols | Incidence (27% vs. 26%, p=1.0) and time to recurrence of FSGS in the kidney allograft (p=0.22) were not significantly different in both groups |
| Gohh RY, 2005 [100]  U.S. | Preemptive Plasmapheresis and Recurrence of FSGS in High-Risk Renal Transplant Recipients | Prospective study | Focal segmental glomerulosclerosis  kidney transplantation  plasmapheresis | 10 | Children (n=1, 9 yrs old) and adults (n=9)  Study period 1999-2003  Patients at high-risk for recurrence due to, all FSGS  -rapid progression to renal failure (n=4)   - -prior transplant recurrence (n=6) | **Preemptive/prophylactic PP:**  10/10 (100%)  8 plasmapheresis sessions over a time span of 2 weeks perioperatively.  LRD: from 1 week before Tx until 1 week post-Tx.  Cad.Tx: 1st session within 24h after Tx.  **Therapeutic PP:**  3/10 (30%) | The expected recurrence rate was 60% based on inclusion criteria, but was only 30%.  7/10 without recurrence (all with 1st Tx, 3 with prior recurrence).  Recurrence: 3/10 patients within 3 months, of those 1 immediately post-Tx despite preemptive PP (only child)  All 3 had graft losses due to recurrence in prior transplant  Remission status after additional PP course:  Complete rem.: 0/3  Partial rem.: 2/3 (67%)  No response: 1/3 (33%)  Outcome:  Graft losses: 2/3 after 225 and 962 days  Deteriorated graft function and nephrotic: 1/3 |
| Gonzalez E, 2011 [101] | Preemptive plasmapheresis and recurrence of focal segmental glomerulosclerosis in pediatric renal transplantation | Prospective study | Focal segmental glomerulosclerosis  Recurrence post-transplantation  Plasmapheresis | 34 | 15/34 LD renal Tx  19/34 had DD renal Tx  Mean age 13±5 yrs  All FSGS  Genetic screening: Performed in NPHS2 | 13/15 with LD: preemptive PP (1-1o sessions)  4/19 with DD: 1-12 sessions | 19/34 (56%) had FSGS recurrence  10/19 had immediate recurrence within 48hrs after transplant surgery, 9/19 within first month post-Tx.  There was no difference in recurrence rate between patients receiving CsA vs. tacrolimus  No difference in recurrence rate in patients with LD and DD renal Tx.  LD renal Tx: There was no difference in the recurrence rate of FSGS in LD patients who received less or more than 5 sessions.  DD renal Tx:  4/19 with recurrence received pre-emptive PP  Conclusion: preemptive PP does not decrease the rate of recurrence after Tx but might be beneficial in treating high-risk patients with documented recurrence. |
| **Immunadsorption** | | | | | | | |
| Kuhn C, 2006 [102] | Effect of Immunoadsorption on Refractory Idiopathic Focal and segmental glomerulosclerosis | Case report | FSGS  SRNS  immunoadsorption |  | SRNS  Resistant to CSA and CPH, azathioprine, MMF, Tacrolimus  Age 34 years old  FSGS | Immunadsorption 10 times fort he duration of 4 weeks (1.5-2fold plasma volume treated)  **Co-medication:**  ACE-I and ARB  **Pre-medication:**  Steroids, CPH pulses | Constant increase in serum albumin  Decrease in proteinuria to 4.5 g/day.  2 month after end of IA: relapse, IA re-started 2 treatments per week for 4 weeks: with concomitant IS again increase of serum albumin and decrease in proteinuria.  An outlet of IA resulted in massive proteinuria.  IA were continued once a week: patient has no clinical signs of NS, Alb 36g/l, proteinuroa 3-3.5g/day. |
| **LDL apheresis** | | | | | | | |
| Muso E, 2001 [103], Japan | Significantly Rapid Relief from Steroid-resistant nephrotic syndrome by LDL apheresis compared with steroid monotherapy | Retrospective study | SRNS  LDL-apheresis | 27 | SRNS  FSGS or MCN  Age 15 to 65 yrs  LDL-A+steroids: n=17  Only steroids: n=10 | LDL-A: twice a week for 3 weeks followed by weekly LDL-A for 6 weeks  NO ACE-I | LDL-A group:  Significant decrease of urinary protein to < 3.5g/day in 13/17 patients, 9 had < 1.0g/day  Increase of albumine (p<0.05): 2.7±0.7 to 3.1±0.7g/dl  No significant changes in steroid only group.  Average duratoion needed for decrease of urine protein to < 3.5g/day was significantly shorter in LDL-A-group (14.7±19.6 days vs. 47.8±6.9 days; p=0.002). |
| Muso E, 2015 [104], Japan | Immediate therapeutic efficacy of low-density lipoprotein apheresis for drug-resistant nephrotic syndrome: evidence from the short-term results from the POLARIS Study | Multicenter prospective observational study | Nephrotic syndrome  FSGS  Low-density lipoprotein apheresis | 44 patien47 episodes | Drug-resistant NS  (steroids and CsA for at least 4 weeks treatment  Age range 18-84 yrs  Mean age: 55.4±17.3 years  FSGS 52.3% | Average no. of LDL-A sessions 9.6 times  Co-medication:?? Not reported | Short-term clinical efficacy:   - rapid improvement of hyperlipidemia - significantly improved proteinuria: 10/47 (21.3%) patients Up from >3.5 g/day to <1.0 g/day, 15/47 (31.9%) Up from > 3.5/day to 1-3.5 g/day   significantly improved hypoproteinemia |
| Hattori M, 1993 [105] | Treatment with a combination of low-density lipoprotein apheresis and pravastatin of a patient with drug-resistant nephrotic syndrome due to focal segmental glomerulosclerosis | Case report | Nephrotic syndrome  FSGS  SRNS  Low-density lipoprotein apheresis | 1 | 15 year old boy  FSGS  Drug-resistant (IV MP, 12-week course of CPH, oral pred, CsA | LDL-A twice weekly for 3 weeks (6 sessions)  With 4th apheresis pravastatin 20 mg(day was started.  Co-medication: CsA | Renal function improved markedly in parallel with areduction in hypercholesterinemia.  Urinary protein excretion decreased slighthly |
| Kawasaki Y, 2007 [106]  Japan | Long-term efficacy of low-density lipoprotein apheresis for focal and segmental glomerulosclerosis | Case report | FSGS  SRNS  Low-density lipoprotein apheresis | 1 | 13 year old boy with FSGS  SRNS, CsA-resistant NS (failed after 8 weeks) and hyperlipidemia | LDL-A was performed 24 times on one year.  Co-medication: Pravastatin  Pre-medication:  IVMP pulses  CsA | Following LDL-A, LDL decreased, parallel urinary excretion of protein decreased.  2nd biopsy showed improvemeent (no glomeruli with FSGS were found).  No proteinuria fro 12 years since LDL-A |
| Oto J, 2009 [107] Japan | Low-density lipoprotein apheresis in a pediatric patient with refractory nephrotic syndrome due to focal segmental glomerulosclerosis | Case report | Nephrotic syndrome  FSGS  Low-density lipoprotein apheresis | 1 | 8 year old girl  SRNS  Non-IgA diffuse mesangial proliferative GN  No remission to IVMP, CsA (2 weeks), oral CPH (7 weeks).  2nd Bx: FSGS.  High LDL-levels | LDL-apheresis 5 times in 3 weeks (PE 2000 ml per session), followed by MP Pulse therapy for 3 days, re-start CsA, combined with oral | Urinary protein decreased to 0.5-1.0 g/day |
| Hattori M, 2003 [108], Japan | A combined low-density lipoprotein apheresis and prednisone therapy for steroid-resistant primary focal segmental glomerulosclerosis in children | Retrospective study | Steroid-resistant primary focal segmental glomerulosclerosis (SR-FSGS)  Low-density lipoprotein (LDL-A)  children | 11 | Mean age at disease onset: 10.9 ± 2.7 yrs (median 12.0 yrs, range 7.0 to 14.4 yrs)  Biopsy-proven FSGS 11/11  SRNS and CsA-resistant  Primary SRNS: 5/11  Secondary SR: 6/11  Mean follow-up after LDL-A:  4.7 ± 3.7 yrs (median 4.1 yrs, range 0.6 to 11.1 yrs) | **LDL-apheresis (LDL-A)**  (Liposorber LA-15)  plasma exchange volumen: 60 ml/kg  regimen: twice weekly treatment for 3 weeks (1st course), then weekly treatment for 6 weeks (2nd course).  Mean no. of sessions: 11.5 ± 0.7 per patient  Mean interval between onset of disease and LDL-A start: 2.0 ± 1.1 years (median 1.9 yrs, range 0.7 to 3.8 yrs)  **Prednisone:**  Start at LDL-A session 7  Dose: 1 mg/kg/d for 6 weeks, followed by tapering schedule during subsequent months  **Pre-treatment:**  Oral steroids 11/11  Methylptrednisolone 9/11  CsA 11/11  Cyclophosphamide 8/11  No ACEi  Statins 10/11 | Remission status:  Complete remissio: 5/11 (45%)  Partial remission: 2/11 (18%)  No remission: 4/11 (36%)  Relapses: 3/5, achieved CR after reinitiation of standard prednisone therapy  Graft function:  Normal: 5/5 with CR during follow-up (median 4.5 yrs)  Stable: 1/2 with PR  Decline: 1/2 with PR, 4/11 with missing remission, ESRD 7.8 yrs after LDL-A  Complications of LDL-A:  Catheter-related infection 1/7 |
| **Leukocytapheresis (LCAP)** | | | | | | | |
| Takakura M, 2017 [109] | Successful treatment of rituximab- and steroid-resistant nephrotic syndrome with leukocytapheresis | Case report | SRNS  FSGS  Rituximab  leukocytapheresis | 1 | SRNS  Rituximab-resistant  FSGS  4 year old boy | LCAP: 4 treatments (2 times a week fro 2 weeks; 750 ml of whole blood was processed at a blood flow rate of 20 ml/min.  Co-medication:  Steroid pulses 500 mg/day for 3 days  **Pre-Treatment:**  Oral steroids  IV MP  CsA (stopped due to posterior leukoencephalopathy syndrome)  Mizoribine  RTX | After LCAP:  Significant reduction in proteinuria and in total number of lymphocytes, T cells and HLA-DR^+^ activated T cells. B cells reappeared after LCAP.  Patient became sensitive to steroids and RTX. Proteinuria decreased further, complete remission achieved.  12 months aftter LCAP+RTX: No significant proteinuria with maintennace treatment mizoribine. |

**Table S5: Treatment Studies in Pediatric Post-Transplant SRNS Recurrence**

| **1^st^ author,**  **year,**  **country of origin**  **[Ref.]** | **Title of Publication** | **Study design** | **Key words** | **N** | **Population Characteristics** | **Treatment** | **Outcomes** |
| --- | --- | --- | --- | --- | --- | --- | --- |
| **Multimodal treatment of post-transplant SRNS recurrence** | | | | | | | |
| Francis A, 2018 [110]  Australia and New Zealand | Treatment of recurrent focal segmental glomerulosclerosis post-kidney transplantation in Australian and New Zealand children: A retrospective cohort study | Retrospective study  Multicentre (n=4) cohort study | Focal segmental glomerulosclerosis  Disease recurrence  Kidney transplant  Outcomes and treatment | 24 | Recurrent FSGS  between 1990-2015  10/24 with primary SRNS  14/24 with secondary steroid resistance  18/22 with FSGS in native kidneys  4/22 with MCGN  10/24 with genetic screening; 1/24 CNS  Age < 18 years Median age at transplant: 12 (9-14) years  LRD 11/24 (46%) DD 13/24 (54%)  Median time to 1st recurrence: 4 days (IQR 2-5 days)  Immunosuppressiv e regimen:  Tac/MMF/Pred.: 11/24 (46%)  CsA/MMF/Pred.: 8/24 (33%)  CsA/Aza/Pred.: 4/24 (17%)  Induction therapy:  Basiliximab 14/24 (58%)  Other (MP, ATG) 3/24 (13%)  None 7/24 (29%)  Pre-emptive plasma exchange: 3/24 (12%) | 14 separate recurrence treatment strategies, most patients had multiple therapies, on average 2-3 therapies  Most common:  **Plasma exchange 20/24 (83%)**  Volume changes: 1.5 Treatment periods ranging 2 weeks to > 2yrs  3/20 with pre-emptive plasma exchange  **High-dose CsA 15/24 (63%),** of those CsA IV 4/15 (given for 1 months), CsA po 11/15 (aiming for trough levels > 350 ng/ml)  5 changed from Tac to high-dose CsA  **Methylprednisolone 9/24 (38%)**  3 doses á 250 mg/m^2^  **CPH 7/24 (29%):**  Oral administration for 6-8 weeks at 2 mg/kg/day; MMF was ceased during CPH  **Rituximab 5/24 (21%)**  3-4 doses á 500 mg, weekly  **Abatacept** (5 doses á 500 mg) **and Galactose** (11.4 gm BD 6 months)  **Co-medication:**  Immunosuppressive regimen  ACEi 4/24 (17%) | Remission status:  Complete remission: 15/24 (63%, 95% CI 43-79%)  Partial remission: 2/24 (8%; 95% CI 2-26%)  No remission: 7/24 (29%; 95% CI 15-49%), of those 5/7 with graft loss due to recurrence, 1/7 due to acute vascular rejection  Responses to different treatment (overlapping effects possible):  **Plasma Exchange:**  CR: 13/20 (65%, 95% CI 43-81%) PR.: 2/20 (10%; 95% CI 3-30%) No resp.: 5/20 (25%; 95% CI 11-47%)  Median dose of plasma exchange: Compl. Rem.: 25 exchanges oer 3 months  Partial/no Rem.: 55 exchanges over 7 months  **High-Dose CsA:**  CR: 9/15 (60%; 95% CI 36-80%)  PR: 0/15 No resp.: 6/15 (40%; 95% CI 15-58%)  **Methylprednisolone:** CR: 6/9 (66%) PR: 0/9  No resp.: 3/9 (33%)  **CPH:**  CR: 5/7 (72%) PR: 1/7 (14%)  No resp.: 1/7 (14%)  **Rituximab:** CR: 2/5 (40%)  PR: 1/5 (20%)  No resp.: 2/5 (40%)  Outcome:  Median 5 year-graft survival after recurrence: 77% (95% CI 40-96%)  Graft loss during study: 9/24 (38%), of those 5/9 due to recurrence  2/15 children with complete remission: Further relapses in same transplant  2/5 children with graft loss due to recurrence experienced recurrence in 2nd graft |
| Morello W, 2019 [111]  Italy | Post-transplant recurrence of steroid resistant nephrotic syndrome in children: the Italian experience | Retrospective study  Multicentre study (5) | Steroid resistant nephrotic syndrome  Kidney transplant  Post-transplant recurrenve | 32 with recurrence | Median age at disease onset: 2.8 (0-17.2 yrs)  Time to ESRD: 3.3 (1.7-14.3 yrs)  Median age at transplant: 11.8 (2.6-20.8 yrs)  Median time to recurrence: 2 days post-Tx  Median follow-up: 58.5 (0.7-157.8) months | **Plasmapheresis (PP):**  32/32 (100%)  median no. of sessions: 20 (range 4-79)  **Co-treatment:**  Rituximab 22/32  High-dose steroids 9/32  Ofatumumab 3/32  IVIG 2/32  Abatacept 2/32  Cyclophosphamide 2/32  CsA 1/32  Thymoglobulin 1/32  Mesenchymal stromal cells 2/32 | Recurrence rate in all patients with FSGS: 32/101 (31.7%)  Recurrence rate in idiopathic SRNS/ unknown SRNS (genetic SRNS excluded): 53%  Remission status:  Overall:  Complete remission 15/32 (47%)  Partial remission 4/32 (13%)  No remission: 13/32 (41%)  Graft loss:  11/13 with no remission  1/4 with partial remission due to rejection  1/15 with CR: 2nd untreatable late relapse 10 yrs after transplant |
| Shishido S, 2013 [112]  Japan | Combination of pulse methylprednisolone infusions with cyclosporine-based immunosuppression is safe and effective to treat recurrent focal segmental glomerulosclerosis after pediatric kidney transplantation | Retrospective study  Single centter | Children  Focal segmental glomerulosclerosis  Kidney transplantation  Nephrotic syndrome  recurrence | 10 | Recurrent FSGS in children between 2000-2009  M:F-ratio 1:1  Genetic screening performed (NPHS1, NPHS2, WT1, ACTN4), no genetic forms  Mean age at disease onset: 4.8±5.1 yrs  Immunsuppressive regimen:  CsA+oral steroids+MMF  Induction therapy: Basiliximab  Mean age at Tx.: 10.1±6.2 yrs | Combined treatment MP pulses with high-dose CsA-based IS:  **Methylprednisolone (MP)**  Dosing: 20 mg/kg/day (max. 1g) on 3 consecutive days at week 1, week 3, week 5, then monthly until 6 months after Tx.  In case of CR/PR: every 3 months until 24 months after Tx  **Oral CsA:**  Started at 350 mg/m^2^/d, targeting AUC0-4 of 3000-3500 ng*h/mL (150-250 ng/mL at trough concentration level) for the first 3 month, then 2000-2500 ng*h/mL  In case of recurrence:  1st month: AUC0-4 level of 4500-5500 ng*h/mL 2./3. month: 4000 ng*h/mL  thereafter: 3000 ng*h/mL  **Concomitant treatment:**  Oral steroids (2 mg/kg/d, tapered biweekly to a dosage of 4-8 mg/kg/d by 3 months) MMF 600-1200 mg/m^2^/d  ACEi (lisinopril) | Remission status within 18 months:  Complete remission: 7/10 (70%)  Partial remission: 1/10 (10%)  No remission: 2/10 (20%), 1 graft loss 4 months after Tx.  Time to CR:  4/7 within 3 months, 3/7 within 6-18 months  Overall graft survival:  1 yr: 90%  5 and 10 yrs: 79%  Patient survival: 100%  Adverse events:  Hypertension 3/10  Mild cataract 1/10 |
| **Intravenous Cyclosporine A (CsA)** | | | | | | | |
| Salomon R, 2003 [113]  France | Intravenous cyclosporine therapy in recurrent nephrotic syndrome after renal transplantation in children | Retrospective study  Single center | Nephrotic syndrome  Recurrence  Renal transplantation  Cyclosporine A | 17 FSGS recurrence  35 Tx pts.  36 grafts | Study period 1991-2001; children  Mean age at onset of SRNS: 4.9±2.7 (range 1.3-10) years  Time to ESRD: 5.5±3.0 yrs.  Mean age at Tx: 12.3±3.5 yrs.  LRD: 1/17  DD: 16/17  Immunosuppr. regimen: Induction: ATG or basiliximab  Maintenance: CsA+Aza+Predn.  16/17: Early recurrence within 4 days post-Tx  1/17 recurrence after 18 days  5/17 with acute renal failure at recurrence  Mean follow-up 4.1±3.3 (range 0.25-10) yrs | **IV CsA:**  Continous intravenous infusion at an initial dose of 3mg/kg*d; dose adaptions to maintain CsA levels between 250-350 ng/mL.  Switch to oral CsA after 3-4 weeks.  **+ Extracorporeal treatments:**  4/17 (24%) Plasma exchange (PE) during first two months due to persistent renal failure.  **Concomitant treatment:**  ACEi (both with partial remission) | Remission status at 2 months posttransplantation:  Complete rem.: 14/17 (82%)  Partial rem.: 2/17 (12%)  No rem.: 2/17 (6%)  Time to CR: 20.8±8.4 (range 12-40) days  Persistent remission 11/17 (65%) with a follow-up of 3.7±3 (range 0.3-9) years  Graft losses: 4/17 (24%), of those 1 with No rem., 1 with initial PR, 2 with initial CR  Renal graft survival:  1 yr: 92%; 5 yrs: 70%  Renal pathology: in 8/11 patients with persistent remission at latest follow-up:  No signs of CsA toxicity in biopsy at 1st year posttransplantation |
| Raafat RH, 2004 [114]  U.S. | High-dose oral cyclosporin therapy for recurrent focal segmental glomerulosclerosis in children | Retrospective study  Single center | Focal segmental glomerulosclerosis  FSGS  Renal transplantation  Cyclosporin A (CsA)  proteinuria | 16 | Children  Study period 1991-2003  DD 10/16  LRD 5/16, unrelated 1/16  Immunosuppressive regimen:  Induction: thymoglobulin (10/16), IL-2-recptor antibody(2/16), 1 IV CsA  Maintenance: CsA+MMF/Aza+ Predn.  Recurrence within 4 days post-Tx in 15/16 patients (95%), at day 20 1/16 | **High-dose CsA:**  Gradual increase of CsA dose until remission was achieved or evidence of renal toxicity (increase of serum creatinine)  Dose range 6-25 mg/kg/d  CsA levels were not used to guide the therapy (variation of trough levels between 200 and 1000 ng/mL)  **Plasmapheresis (PP):**  7/16 (44%) with 7-10 sessions, reapted in 3/7 children | Recurrence rate: 16/23 (67%)  Remission status:  Complete remssion: 11/16 (69%)  Partial remission: 2/16 (13%)  No remission: 3/16 (19%) (all had PP parallely)  Time to complete remission:  Within 30 days: 6/11  Within 3 months: 4/16  At 19 months (before partial rem.): 1/16  Graft losses:  3/16 (19%), of those, 1 due to recurrenve, 1 due to recurrence + non-compliance, 1 due to recurrence+rejectiom+non-compliance  Adverse events:  CsA toxicity with hirsutism and gingival hypertrophy 16/16 (100%) |
| **Cyclophosphamide** | | | | | | | |
| Cochat P, 1993 [115]  France | Recurrent nephrotic syndrome after transplantation: early treatment with plasmapheresis and cyclophosphamide | Prospective uncontrolled trial | Nephrotic syndrome  Transplantation  Plasmapheresis  cyclophosphamide | 3 | Children  Patients age:  6.5, 13.3 and 15.8 yrs  DD  Immunosuppressive regimen: Predn.+CsA+Aza  Early recurrence post-Tx | Early plasmapheresis within 5-10 days after onset of recurrence  Methylprednisolone Pulses  Cyclophosphamide (instead of azathioprine) over a 2-month period | Remission status:  Rapid and sustained complete remission: 3/3 (100%)  Time to remission: 12-24 daysd of treatment  2nd late recurrence: 1/3, controlled within 7 weeks  At last follow-up (18 or 27 months post-Tx): all with normal renal function, complete remission, normal blood pressure  Complications of reinforced immunosuppression:  Septic ankle arthritis 1/3 children |
| Cheong HI, 2000 [116]  Korea | Early recurent nephrotic syndrome after renal transplantation in children with focal segmental glomerulosclerosis | Retrospective study  Single center | FSGS  Recurrent nephrotic syndrome  Cyclophosphamide  Renal transplantation  Plasmapheresis | 6 | Study period 1999-2000  Children with age < 18 yrs  Mean age at SRNS onset: 7.2±3.3 yrs  Mean age at Tx: 11.8±3.2 yrs  LRD 5/7  DD 2/7  Immunosuppressive regimen post-Tx: CsA based  Duration of follow-up after Tx.: 43±20 months | Recurrence treatment according to [115] with minor modifications:  **Combination of**  **Plasma exchanges (PE):**  10 sessions over 2 weeks, the 1 session per week for 2 months  (1.5 plasma volume replaced by 4% albumin; IgG substituted as required)  **Methylprednisolone:**  3 boluses á 250 mg/m^2^/day  **Replacement of Azathioprin by Cyclocphosphamid (CPH) orally:** 2 mg/kg/day for 3 months  **CsA** with trough levels 150-200 ng/mL | Remission status:  Complete rem.: 3/6 (50%), of those persistent remission 2/6 (33%), 1 with recurrence proteinuria  Partial rem.: 3/1 (50%), of those 2 with additional acute rejection and 1 with delayed treatment  Graft loss: 1/6 (17%) |
| Dall’Amico R, 1999 [117]  Italy | Prediction and treatment of recurrent focal segmental glomerulosclerosis after renal transplantation in children | Retrospective study | Focal segmental glomerulosclerosis  Renal transplantation  Children  Recurrence | 15 of 29 | Study Period 1987-1998 | Treatment with plasmapheresis and cyclophosphamide  11/15 patients | Recurrence rate 15/29 (52%) after 1st Tx, 3/3 after 2nd Tx  Remission rate after PP and CPH:  Complete remission: 7/11 (63%)  Partial remisson: 2/11 (18%)  Graft losses: 6/15 within < 24 months |
| **Rituximab** | | | | | | | |
| Sethna C, 2011 [118] | Treatment of recurrent focal segmental glomerulosclerosis in pediatric kidney transpant recipients: effect of rituximab | Retrospective study  Single center | Focal segmental glonerulosclerosis  Recurrent FSGS  Pediatric Kidney transplant  Rituximab | 4 | Adolescents  Age range: 13-18 yrs  DD 4/4  Immunosuppressive regimen:  Induction: thymoglobulin  Maintenance: Tac+MMF-Predn.  Mean follow-up 22.5 months | **Rituximab**  375 mg/m^2^/dose once weekly for 4 doses  Administration 171±180 days (range 10-395 days) post-transplant and 114±169 days (range 8-389 days) after the PP start  **Plasmapheresis (PP)**  Initiation 58±106 days post-transplant (range 2-217 days)  2/4 patients treated with PP and rituximab within 2 weeks post-transplant. | Remission status:  Complete remission: 3/4 (75%), of those 1 relapse within 4 month  Partial remission: 1/4 (25%), but not maintained  Outcome at last follow-up (22-24 months post-rituximab):  Without PP: 3/4  PP-dependent: 1/4  eGFR range: 88-127 ml/min*1.73m^2^ |
| Kumar J, 2013 [119]  U.S. | Rituximab in post-transplant pediatric recurrent focal segmental glomerulosclerosis | Retrospective study  Multicenter (4)  study | Focal segmental glomerulosclerosis  Recurrent disease  Transplant  Proteinuria  Plasmapheresis | 8 | Children´s age range 5-17 yrs  LRD 6/8  DD 2/8  Immunosuppr. Regimen:  Induction: Thymoglobulin and MP 6/8  Basiliximab and MP 2/8  Maintenance: Tac/CsA+MMF  +Predn.  Immediate post-transplant SRNS recurrence 7/8,  After 4yrs: 1/8 | **Rituximab**  375 mg/m^2^/dose once weekly for 1-4 doses  **Pre-RTX extracorporeal treatment:**  Pre-emptive Plasmapheresis (PP): 4/8 (50%)  Therapeutic Plasmapheresis: 4/8 (50%)  Duration: 3 days to 61 months post-transplant before administration of RTX  **Concomitant treatment:**  ACEi and/or ARB | Remission status:  Complete rem.: 2/8 (25%)  Partial rem.: 4/8 (50%)  No rem.: 2/8 (50%)  Time to CR: 1.5-8 months  Time to PR: 0.5-12 months  Adverse events:  Acute tubular necrosis+AKI 1/8 (immediate after RTX)  CNS malignancy 1/8 (1yr after RTX)  RTX-associated lung-injury+death 1/8 (5 weeks after RTX) |
| Garrouste C, 2016 [120]  France | Rituximab for Recurrence of Primary Focal Segmental Glomerulosclerosis after kidney transplantation: clinical outcomes | Retrospective study  Multicenter (13) study | Focal segmental glomerulosclerosis  Recurrence  Kidney transplantation | 19 | Children and adults.  Age 35 (15-66) yrs  Age 1-16 yrs: 5/19 (27%)  Age 18-60 yrs: 14/19 (73)  Immunosuppressive regimen:  Induction with ATG 10/19 (53%)  Maintenance  CsA (orally 8/19; IV 5/19) or  Tac (6/19)  + MMF + Steroids  FSGS recurrence at 12 (1.5-27) days posttransplantation,  2/10 with late recurrence (100 and 247 days) | **Rituximab**  375 mg/m^2^, median of 2 (1-4) infusions  **+**  **Conventional treatment**: Plasma exchanges (PP), high doses of CNI, steroids)  Different timing:  Group 1: n=6 (32%)  Administration of Rituximab immediately after recurrence diagnosis, parallel PE, IV CsA (4/6) or Tac (2/6)  Group 2: n= 10 (53%)  Administration of RTX after failure of conventional treatment (resistance to IV CsA, steroids and PE) at a median time of 56 (24-138) days after FSGS recurrence  Group 3: n=3 (16%)  Administration of RTX after PE withdrawal or premature recurrence after PE discontinuation.  2/3 PE-dependent (CR, but relapse with weaning of PE) | Remission status:  Complete rem.: 9/19 (47%)  Partial rem.: 3/19 (16%)  No rem.: 7/19 (37%)  Group 1:  CR: 3/6 (50%); PR 1/6 (17%), No rem.: 2/6 (33%); both with graft losses 24 months later  4/6 with serious side effects (see below)  Group 2:  CR: 4/10 (40%); PR 1/10 (10%), No rem.: 5/10 (50%)  Graft losses: 3/10 (10%), of those 2 due to recurrence, 1 due to chronic antibody-mediated rejectio 12 yrs post-Tx.  8/10 with serious side effects (see below)  Group 3:  Maintaining CR: 2/3, PR 1/3  2/3 with serious side effects (see below)  Renal survival:  Overall at 5yrs: 77.4% (95% CI, 41.9-92.7)  1yr: CR/PR 100% vs. No rem. 86%  5 yrs: CR/PR 100% vs. No rem. 34.3%  Adverse events:  Severe infections during 1st year after Tx: 14/19 patients (infections: bacterial 16, viral 4, parasitic 1) |
| Alhasan KA, 2019 [121]  Saudi Arabia | Successful Treatment of Recurrent Focal Segmental Glomerulosclerosis after transplantation in children | Retrospective study  Single center | Focal segmental glomerulosclerosis  Children  Kidney transplantation  Recurrence  Rituximab  Abatacept | 6 | Study Period 2014-2016  Median age at SRNS diagnosis: 2.75 (range, 2-4) years  All negative in genetic screening: *NPHS1, NPHS2, WT1, PLCE1, LAMB2, CD2AP*  Time to ESRD: 19 (range, 8-30) months  LRD 5/6  LD, unrelated 1/6  Immunosuppr. regimen:  Induction: basiliximab 4/6  ATG 2/6 Maintenance: Tac+MMF+Predn.  Early recurrence within 1-3 days post-Tx | **Rituximab** 5/6 (83%)  375 mg/m^2^, 4 infusions every 2 weeks  **Concomitant extracorporeal treatment:**  **Plasma exchange (PE):**  All (100%):  **Prophylactic PE:**  3 every-other-day sessions 1 week prior transplant  **Therapeutic PE:**  Daily sessions for 2 weeks, every other day for 4-6 weeks, thereafter twice weekly  Duration of PE: 2-5 months  **Concomitangt treatment:**  2/6 Abatacept  3/6 Switch from Tac to CsA | Remission status:  Complete rem.: 5/6 (83%)  No rem.: 1/6 (17%)  Adverse events:  Death 1/6 due to pneumonia and sepsis at 8 months posttransplantation (received Abatacept after PP and RTX without response to any of the treatments) |
| Alasfar S, 2018 [122]  U.S. | Rituximab and therapeutic plasma exchange in recurrent focal segmental glomerulosclerosis postkidney transplantation | Prospective observational study | FSGS  Recurrence  Kidney transplantation  Plasma exchange  rituximab | 66 | Adolescents, mainly adults  Mean age at FSGS diagnosis: 29.9 yrs (range: 18-59 yrs)  Mean time to ESRD: 4 yrs /range 0-9 yrs)  1st Tx: 42/66  2nd Tx 22/66  3rd Tx: 2/66  LRD 25/66  DD: 41/66  Maintenance immunosuppressionCNI+MMF+Predn.  Median duration of follow-up post-Tx: 29.5 months | **Preemptive treatment** in high-risk recurrence patients:  Preemptive PP (3-10 sessions) and/or rituximab (1-2 doses á 375 mg/m^2^)  37/66 (56%)  **Standardized treatment of recurrence:**  Plasmapheresis (at least 10 sessions, 8/66 more with range 16-35 sessions)  Low dose IVIG (200 mg/kg)  Rituximab 20/66 (30%)  **Co-treatment:**  ACEi or ARB 35/66 (53%) | Recurrence rate :  Overall: 39/66 (59%)  with preventive treatments: 23/37 (62%)  without preventive treatments: 14/27 (51%)  Remission status:  Responder: 35/66 (53%), not differented between compl./partial remission  Non-responder: 31/66 (47%)  Subsequent relapses:  14/35 (40%): at least 1 relapse  Graft losses:  Overall: 8/66  Due to initial recurrence: 4/8  Due to subsequent relapse of recurrence: 2/8  Due to rejection: 1/8  Due to cardivascular death: 1/8 |
| **Ofatumumab** | | | | | | | |
| Bernard J, 2018 [123]  France | Ofatumumab in post-transplantation recurrence of a pediatric steroid-resistant idiopathic nephrotic syndrome | Case report | Nephrotic syndrome  Post-transplantation  Children  Abatacept  Rituximab  Ofatumumab | 1 | SRNS, FSGS  SRNS diagnosis at 5 yrs of age  ESRD at 7 yrs of age  Living related Tx at 9 yrs of age  Early recurrence and in the end  graft loss due to SRNS recurrence at 12 yrs of age  2nd Tx (from deceased donor) at 15 yrs of age.  Induction with ATG, Immunosuppressive regimen CsA+MMF+Pred. | **Ofatumumab** (2.5 yrs after recurrence diagnosis):  300 mg/1.73m^2^, in total 6 times.  **Pre-treatments:**  Immunoadsorption  -> IA-dependent  **Pre-medications:**  Change from CsA to Tac  Rituximab (3 infusions á 375 mg/m^2^) Abatacept (6 x 10 mg/kg infusions)  Dual ACEi/ARB therapy (enalapril and losartan)  -> SRNS recurrence resistant to Rituximab, Abatacept  **Co-medication:** Maintenance immunosuppression ACEi/ARB anti-pneumocystis prophylaxis with cotrimoxazole, later atovaquone during at least 1 year. | **Outcome:**  After Ofatumumab cessation of IA was possible. Partial remission of SRNS recurrence in 2nd renal transplant with maintaining proteinuria between UPCR 0.3-0.6 g/mmol, albumin > 30g/L, stable renal function (serum creatinine 100-100 μmol/L) |
| Solomon S, 2018 [124]  U.S. | Ofatumumab in post-transplantation recurrence of focal segmental glomerulosclerosis in a child | Case report | FSGS  Ofatumumab  Renal transplant | 1 | 13 year old boy  Initial manifestation: 3 yrs  Multidrug-resistant SRNS (CNI, MMF, rituximab)  Negative genetic screening (*INF-1, ACTN4, TRPC6, WT1, NPHS1, NPHS2)*  DD  Immunosuppressive regimen: Induction: thymoglobulin, MP Pulses  Maintenance: Tac+MMF+Predn.  Recurrence within 48 hrs after Tx  Mean follow-up: 13 months post-Tx | **Ofatumumab:**  4 months post-transplant  Pre-medication to avoid aanaphylaxis  Test dose: 300 mg/m^2.^  Dose 2: 700 mg/m^2^  Dose 3: 1g/m^2^  Dose 4: 1.5 g/m^2^  Dose 5-7: 2 g/m^2^  **Pre-treatment:**  Plasmapheresis (6 months), rituximab (2 doses), Switch from Tac to CsA (target range 250-300 ng/mL)  -> Improvement of proteinuria but remaining nephrotic-range | Partial remission after ofatumumab (2 and 4 months) |
| Colucci M, 2019 [125]  Italy | Ofatumumab rescue treatment in post-transplant recurrence of focal segmental glomerulosclerosis | Case report | FSGS  Ofatumumab  Children  Steroid-resistant nephrotic syndrome  Post-transplantation | 2 | 2 boys at the age of 16 and 18 yrs  Initial age at diagnosis 4 yrs and 7yrs  DD/ LRD  Immunosuppressive regimen:  Induction: Basiliximab, MP Pulses  Maintenance: CNI+MMF+ Predn.  Early post-transplant recurrence 2 and 3 days | **Ofatumumab:**  Dose 1: 1500 mg/m^2^ 17 months post-transplant (Case 1), 3 months (Case 2)  Dose 2: 1500 mg/m^2^ 24 months post-transplant, 5 months (Case 2)  **Pre-/Cotreatment:**  Plasmapheresis  Switch from Tac to high-dose CsA  Rituximab (1-2 infusions) | Remission status:  Case 1: partial remission after PP/High-dose CsA/rituximab and complete remission after ofatumumab, stable for more than 12 months  Case 2: partial remission |
| **Abatacept** | | | | | | | |
| Delville M, 2015 [126]  France | B7-1 blockade does not improve post-transplant nephrotic syndrome caused by recurrent FSGS | Prospective case series | Nephrotic syndrome  FSGS  Post-transplant recurrence  B7-1 Blockade  Abatacept  Belatacept | 9 | Adults (n=7) and children (n=2, 5 yrs with 1st Tx and 12 yrs old with 2nd Tx, 1st failed due to FSGS recurrence)  Native kidneys:  SDNS 8/9  Sec. SRNS (1/9)  LRD 1/9  DD 8/9  Recurrence in both children: at day 1 and 7 post-Tx | **Abatacept: n=5** (both children)  10 mg/kg, 2-3 doses  **Belatacept: n=4**  5 to > 10 infusions  **Extracorporeal treatment:**  Plasma exchange (PE) 6/9 (67%)  Immunoadsorption (IA) 3/9 (33%) (both children)  **Concomitant/ Pre-treatment:**  High dose CsA/ IV CsA  Steroids  Rituxmab 4/9 (both children) | Remission status:  Complete rem.: 0/9  Partial rem.: 0/9  No response: 9/9 (100%)  Children:  12 yr old child: 2nd graft  Initial complete remission after IA and RTX, then relapse after IA discontinuation, No weaning of IA possible after 5 Abatacept infusions, persistent proteinuria (partial remission) IA-dependent.  5-yr old child: 1st graft  No response to IA, IVIG, RTX. No response after 3 Abatcept infusions.  Conclusion of authors:  B7-1 blockade did not induce FSGS remission after transplant in nine patients |
| Alkandari O, 2016 [127]  Kuwait | Recurrent focal segmental glomerulosclerosis and Abatacept: Case report | Case report | End-stage renal disease  Immunosuppression  Pediatric  Renal transplant | 1 | 11 year old girl with SRNS (FSGS)  Negativ genetic screening for *NPHS2*  Time to ESRD: 2 yrs  Age at Tx: 13 yrs  Immunosuppressive regimen: Induction: Basiliximab  Maintenance: CsA + MMF + Predn.  Recurrence 6 days after Tx | Prophylactic treatment pre-Tx:   - Plasma exchange (PE) 2x (3 and 2 days before Tx) - Rituximab 375 mg/m^2^ at the day of Tx   Recurrence resistant to standard therapy: RTX, Plasmapheresis, high-dose CsA, corticosteroids)  **PE + Abatacept**  10 mg/kg, 3 doses at week 0, 2, 4 weeks  **Concomitant treatment:**  ACEi or ARB | No response to Abatacept in addition to PE and drug standard treatment (RTX, high-dose CsA, croticosteroids) |
|  |  |  |  |  |  |  |  |
| **Anti-TNFα antibody** | | | | | | | |
| Leroy S, 2009 [128]  France | Successful Anti-TNFα treatment in a child with posttransplant recurrent focal segmental glomerulosclerosis | Case report | Anti-tumor necrosis factor-α treatment  Nephrotic syndrome  Posttransplant recurrence  Steroid resistance | 1 | 12 yr old boy with CsA-resistant idiopathic NS (FSGS)  ESRD at 15 yrs of age  DD at 16 yrs of age  Immunosuppressive regimen:  Induction: Basiliximab  Maintenance:  CsA+MMF+Predn.  Recurrence at day 7 post-Tx | **Anti-TNFα monoclonal antibody (Infliximab):**  3 mg/kg twice a month+high-dose steroids, start at day 45 post-Tx and uncontrolled recurrent FSGS  **Plasmapheresis (PP):**  15 sessions within 1 month  **Pre-/ Concomitant treatment:**  Switch from high dose CsA orally (10 mg/kg/day, target CsA trough level 200-300 ng/mL) to IV CsA 5 mg/kg/day, thereafter maintaining level 400-500 ng/mL  High-dose steroids (60 mg/1.73m^2^ per day)  Cyclophosphamide orally 100 mg/day  Pefloxacine orally 800 mg/d | Remission after infliximab  2 relapses ocurred when anti-TNFα treatment (infliximab) was discontinued  Control of relapses with combination of etanercept (anti-TNFα blocking agent: 25 mg twice weekly) and high-dose steroids (60 mg/1.73m^2^/d)  Persistent remission at age of 20 yrs on 25 mg weekly etanercept maintenance therapy plus CsA, MMF, low-dose steroids  Side effects of infliximab/ etanercept:  none reported |
| **Extracorporeal Treatments** | | | | | | | |
| **Plasmapheresis** | | | | | | | |
| Kashgary A, 2016 [129] | The role of plasma exchange in treating post-transplant focal segmental glomerulosclerosis: A systematic review and meta-analysis of case-reports and case-series | Systematic review and meta-analysis of 77 case-reports and case-series | Focal segmental glomerulosclerosis  Kidney transplantation  Plasma exchange  Plasmapheresis  Systematic review | 423  45 pts. From 34 case reports  378 from case-series | Adults and children  21 countries  Time period 1985-2012    Median time to FSGS recurrence: 4 days (IQR 1; 18), reportet for 135/423 pts.  Median age at recurrence: 17 yrs (IQR 12; 33)  Age < 18 yrs 119/235 (51%)  Gender:  Male 126/121 (59%)  Female 86/212 (41%)  Donor type:  Living donor: 62/175 (65%)  Deceased donor: 113/175 (35%) | **Plasmaexchange (PE)**  Median time from recurrence to treatment with PE: 1 day (IQR 0;3)  Delayed start with PE > 2 weeks after recurrence diagnosis: 28/230 (12%); > 1 month: 22/230 (10%)  Median no. of PE sessions: 12 (IQR 10; 20)  Median weeks of PE treatment: 12 (IQR 4; 28)  **Concomitant treatments:**  Rituximab 41/404 (10%)  High dose steroids 341/344 (99%) | **Remissions status:**  Overall response (CR+PR): 71% (95% CI: 66-75%)  Complete rem.: 198/423 (47%)  Partial rem.: 119/423 (28%)  No rem.: 106/423 (25%)  **Children:**  CR+PR: 70% (95% CI: 61-78%)  ESRD: 83/381 (22%)  Time to ESRD after Tx: 0.9 (0.3; 2.0) years  **Association with remission:**  Adults vs. Children (235 pts.): OR 1.09 (95% CI 0.55 to 2.16)  Males vs. Females (212 pts): OR 2.85 (95% CI 1.44 to 5.62)  Proteinuria at recurrence (≥ 7g/day vs. < 7g/day) (148 pts.): OR 0.43 (95% CI 0.19 tp 0.97)  Received rituximab (404 pts.): OR 0.60 (95% CI 0.21 tp 1.75)  Transplant typ (living vs. deceased) (423 pts.): OR 1.0 (95% CI 0.43 to 2.3)  Plasma exchange start within 2 wks of FSGS |
| Ohta T, 2001 [130]  Japan | Effect of pre- and postoperative plasmapheresis on post-transplant plasmapheresis on posttransplant recurrence of focal segmental glomerulosclerosis in children | Retrospective study  Case series | Nephrotic syndrome  Children  Posttransplant recurrence  Focal segmental glomerulosclerosis | 8/20 pts.  9/21 grafts | Children  Study period 1984-1997  Native kidneys: FSGS  Age < 15 yrs  18 LRD, 3 DD  **Group A:** Therapeutic PP post-transplant  N=6  **Group B:** Prophylactic PP pre-transplant  N=15  IS regimen: CsA or Tac + MP+ azathioprine or mizoribine | **Plasmapheresis (PP)**  Prophylactic PP:  2-3 sessions before Tx (-5, -3, -1 day)  Therapeutic PP: immediately after recurrence diagnosis  Technique: membrane plasmaseparator  Plasma exchange volume: 50-75 ml/kg, replaced by 5-8% albumin in Ringer`s solution  No. PP sessions: 6 to > 100  **Concomitant treatment:**  2/8 patients: oral CPH + ACEi  1/8: ACEi | Incidence of recurrence:  Patients: 8/20 (40%)  Grafts: 9/21 (43%)  Group A: 4/6 (67%) Group B: 5/15 (33%)  FSGS recurrence within 24 hrs post-transplantation  Remission status:  Complete/ Partial remission (not differentiated):  Grafts: 6/9 (67%), of those 1/4 without prophylactic PP (Group A), 5/5 with prophylactic PP (Group B)  Graft survival not different between group A and B (approx. 60-65%)  Graft loss 2/9 recurrences  Deteriorated Graft function: 5/9  Adverse events of PP:  none |
| Hickson LJ, 2009 [131]  U.S. | Kidney transplantation for primary focal segmental glomerulosclerosis: outcomes and response to therapy for recurrence | Retrospective study  Case series  Single center | Focal segmental glomerulosclerosis  Kidney transplantation  Recurrent disease  Plasmapheresis  Rituximab | 14 | Children and Adults  **Children: n=6** Recurrence rate: 86% (6/7)  Median age at FSGS diagnosis: 8±6yrs Time to ESRD: 3.9±2.3  Median age at Tx: 13±6yrs  **Adults: n=8**  Recurrence rate: 35% (8/23)  13/14 LRD, 1/14 LD (unrelated)  Immunosuppr. regimen: Induction: ATG  Maintenance: Tac or Sirolimus +MMF +Predn. | **Plasmapheresis (PP)**  **Preemptive PP:** 7/14 (50%) days -5, -3, -1 pre-Tx.  **Therapeutic PP:** 7/14 (50%)  Daily for 3-7 days, then 3 days a week for 4-12 weeks, thereafter at frequencies varying from twice weekly to monthly depending on the clinical response  Start of PP: 1-315 days after Tx  PP duration: 4-1248 days  **Concomitant treatment:**  Pediatric patients: 4/6  Rituximab 375 mg/m^2^/ dose every 2 weeks for a total of 1-2 doses. | Overall remission status after initial PP course:  Complete rem.: 6/14 (43%), 3/6 children (50%)  Partial rem.: 3/14 /21%), 0/6 children  No Rem.: 5/14 (36%), 3/6 children (50%)  Stable normal graft function: 7/14 (50%), 3/6 children (50%)  Stable deteriorated graft function: 1/14 (7%), 1/6 children (17%)  Graft loss: 6/14 (43%), 2/6 children (33%)  Death 1/14 (7%)  Remssion status for 4 pediatric patients treated with PP+Rituximab:  Complete rem.: 2/4 (50%)  Partial rem.: 0/4  No rem.: 2/4 (50%) |
| Gonzalez E, 2011 [101] | Preemptive plasmapheresis and recurrence of focal segmental glomerulosclerosis in pediatric renal transplantation | Prospective study | Focal segmental glomerulosclerosis  Recurrence post-transplantation  Plasmapheresis | 34 | 15/34 LD renal Tx  19/34 had DD renal Tx  Mean age 13±5 yrs  All FSGS  Genetic screening: Performed in NPHS2 | **Plasmapheresis (PP)**  13/15 with LD: preemptive PP (1-10 sessions)  4/19 with DD: 1-12 sessions | 19/34 (56%) had FSGS recurrence  10/19 had immediate recurrence within 48hrs after transplant surgery, 9/19 within first month post-Tx.  There was no difference in recurrence rate between patients receiving CsA vs. tacrolimus  No difference in recurrence rate in patients with LD and DD renal Tx.  LD renal Tx: There was no difference in the recurrence rate of FSGS in LD patients who received less or more than 5 sessions.  DD renal Tx:  4/19 with recurrence received pre-emptive PP  Conclusion: preemptive PP does not decrease the rate of recurrence after Tx but might be beneficial in treating high-risk patients with documented recurrence. |
| Araya CE, 2011 [132]  U.S. | The factors that may predict response to rituximab therapy in recurrent focal segmental glomerulosclerosis: A systematic review | Systematic review | Focal segmental glomerulosclerosis  Recurrent FSGS  Renal transplantation  Plasmapheresis  Rituximab | 39 | Children (19/39; 49%)  Adults (20/39; 51%)  Median age at SRNS diagnosis: 6 (1-40) yrs  Timer to ESRD 3 (0.16-19 yrs)  Median age at transplant: 18 (5-48) yrs  LRD 13/39  DD 26/39  Immunosuppressive regimen:  Maintenance:  CNI (Tac/CsA) +MMF+Predn.  Diagnosis of recurrence post-Tx:  Within 1st week 62%  Within 1st month 74% | **Therapeutic Plasmapheresis (PP)**  38/39 (97%)  No. of sessions: 21 (0-133)  **+**  **Rituximab (RTX):**  39/39 (100%)  No. of doses: 4 (1-6)  Time to RTX administration from Tx: 210 (range 4-3543 days), from diagnosis of recurrence: 149 (range 3-1086) days  **Pre-Treatments:**  9/39 preemptive PP pre-Tx  **Concomitant treatments:**  4/39 CPH | Remission status:  Complete rem.: 17/39 (44%)  Partial rem.: 8/39 (21%)  No rem.: 14/39 (36%)  Time to response from RTX: 2 (0.63-12) months  Adverse events:  3/19 children: PTLD  1/19 severe anaphylactic reaction to RTX  1/19 neutropenia |
| Garcia CD, 2006 [133]  Brazil | Plasmapheresis for recurent posttransplant focal segmental glomerulosclerosis | Retrospective study  Single center | FSGS  Renal transplantation  Recurrence  plasmapheresis | 14 with recurrence | Study period 1977-2005  Children < 19 yrs  Mean age at Tx.: 12±4.3 yrs  Immunosuppressive regimen post-Tx:  Induction: Daclizumab/basiliximab 13/14  Maintenance:  Until 1997: CsA+Aza+Predn.  Since 1998: CsA+MMF+Predn. | **Plasmapheresis (PP):**  9/14 (64%) performed since 2001  10 sessions (No. Of sessions limited due to financial reasons)  (3 sessions/week), started within < 48 hrs after diagnosis of recurrence  Plasma volume exchange: 50-75 ml/kg per session with 5-8% albumin.  **Concomitant treatment:**  High-dose CsA (C2 levels 1700-1800 ng/mL) | Remissionstatus:  After 10 PP sessions: 9/14  Complete remission: 5/9 (55%)  Partial rem.: 1/9 (11%)  No rem.: 3/9 (33%)  No further recurrence during 2.6±1.4 yrs follow-up  Without PP: 5/14  No reponse in 5/5 (100%)  Adverse events after PP:  Infections 3/9 (1xCMV, 2x VZV) |
| Fuentes GM, 2010 [134]  Spain | Long-term outcome of focal segmental glomerulosclerosis after pediatric renal transplantation | Retrospective case series  Single center | Focal segmental glomerulosclerosis  Renal transplantation  Recurrence  Plasmapheresis  Cyclosporin | 9 with recurrence | Study period 1985-2007  Children < 18 yrs  Mean age at SRNS diagnosis: 5.9±3.6 yrs  Time to ESRD: 4.5±4.9 yrs  Age at Tx.: 11.6±3.9 yrs  Immunsuppresive regimen: 1985-1999: CsA+Aza+Predn. Since 2000: Tac+MMF+Predn. | **Treatment strategies:**  **Plasmapheresis (PP)+ MP Pulses**  7/9 (78%)  No. of PP sessions: 6-12  **PP+ High-Dose CsA**  1/9 (11%)  CsA as continous IV infusion at initial dose of 3 mg/kg/day, thereafter dose adaption to CsA levels 250-350 ng/ml.  **Concomitant treatment:**  Replacement Aza by CPH 2/9 | Remission status:  Complete/partial rem.: 7/9 (78%)  Renal graft survival:  1yr: 66%  3 yrs: 20%  Graft losses: 4/9 (44%)  Patient survival at 10 yrs: 100% |
| Gohh RY, 2005 [100]  U.S. | Preemptive Plasmapheresis and Recurrence of FSGS in High-Risk Renal Transplant Recipients | Prospective study | Focal segmental glomerulosclerosis  kidney transplantation  plasmapheresis | 10 | Children (n=1, 9 yrs old) and adults (n=9)  Study period 1999-2003  Patients at high-risk for recurrence due to, all FSGS  -rapid progression to renal failure (n=4)  -prior transplant recurrence (n=6) | **Preemptive/prophylactic PP:**  10/10 (100%)  8 plasmapheresis sessions over a time span of 2 weeks perioperatively.  LRD: from 1 week before Tx until 1 week post-Tx.  Cad.Tx: 1st session within 24h after Tx.  **Therapeutic PP:**  3/10 (30%) | The expected recurrence rate was 60% based on inclusion criteria, but was only 30%.  7/10 without recurrence (all with 1st Tx, 3 with prior recurrence).  Recurrence: 3/10 patients within 3 months, of those 1 immediately post-Tx despite preemptive PP (only child)  All 3 had graft losses due to recurrence in prior transplant  Remission status after additional PP course:  Complete rem.: 0/3  Partial rem.: 2/3 (67%)  No response: 1/3 (33%)  Outcome:  Graft losses: 2/3 after 225 and 962 days  Deteriorated graft function and nephrotic: 1/3 |
| Straatmann C, 2014 [135]  U.S. | Success with plasmapheresis treatment for recurrent focal segmental glomerulosclerosis in pediatric renal transplant recipients | Retrospective study  Single center | Focal segmental glomerulosclerosis  Recurrent disease  Kidney transplantation  Plasmapheresis  Graft function | 7 | Study period 1995-2013  LRD: 4/7  DD 3/7  Immunosuppressive regimen:  Induction: Basiliximab  Maintennace: CNI-based  Time to recurrence diagnosis post Tx.: 1-9 days  Median observation period 4.5 (0.8-16.3) yrs | **Therapeutic Plasmapheresis (PP):**  Single volumen plasma exchanges performed with 5% albumin replacement  Rapid of PP Initiation after diagnosis of recurrence (at a median of 5 (range 2-15) days post-Tx and within 3.7±2.7 days after diagnosis of recurrence), initially daily sessions until proteinuria improved (for 9.0±4.0 days), the tapered to 2-3 times weekly (for 8.7±9.4 wks), follwed by once weekly (2.8±5.1 wks) and once every other week intervals  **Concomitant treatment:**  1/7 Switch from Tac to CsA  2/7 CPH  1/7 Rituximab at week 21 and 28 of PP  None ACEi/ARB treatment | Remission status  Complete remission within 13.6±10.6 wks: 7/7 (100%)  Total course of PP ranged from 4 to 32 weeks (median 9 wks)  Sustained remission in a median observation period of 4.5 (0.8-16.3) yrs  Graft function:  No graft losses  Stable renal graft function in 6/7 with creatinine < 1.5 mg/dL |
| Mansur JB, 2019 [136]  Brazil | Clinical features and outcomes of kidney transplant recipients with focal segmental glomerulosclerosis recurrence | Retrospective study  Single-center cohort study | Chronic renal failure  Kidney transplantation  Focal segmental glomerulosclerosis  Plasmapheresis  Proteinuria | 61 | Adults and children:  Age < 18 years: 20/61 (33%)  Study Period 2003-2014  Native kidney biopsy findings:  FSGS 34/61 undetermined chronic glomerulopathy 27/61  Median time to recurrence diagnosis: 19.5 days  1st biopsy-confirmed FSGS recurrence: 18%, reaching 52.4% in follow-up biopsies  Confirmed (biopsy proven) FSFS recurrence : 34/61 (55%)  Suspected: 27/61 (45%)  Induction therapy 40/61: Basiliximab (20/40) or ATG (20/40)  Immunosuppressive regimen: CNI (CsA or Tac) + MMF or AZA or mTori (sirolimus or everolimus) + Pred. | **Plasmapheresis (PP):**  Spectra Optia apheresis system  Volume exchange: 1x, replacememnt 5% albumin  Frequency: 3 times per week, with gradual frequency reduction according to response  Mean number of PP sessions: 22±19  **Concomitant treatments:**  Steroid pulses (250-100 mg MP, 3 Pulses): 43/61 (70%)  High dose oral steroids (0.5-1.0 mg/kg/day): 30/61 (49%)  Introduction of CsA or CPH : 5/61 (8%)  Rituximab (375 mg/m^2^ per dose): 10/61 (16%) | Remission status within 12 months after PP initiation:  Complete remission: 12/61 (20%)  Partial remission: 10/61 (16%)  No response (= treatment failure after at least 20 PP sessions): 16/61 (26%)  Graft loss: 7/61 (11%)  Total graft loss during study period: 30/61 (49%), of those 27 due to FSGS recurrence  Overall 6-year graft survival: 65%  Overall 6-year patient survival: 91%  **Adverse events (AE):**  Infections (at least one): 33/61 (54%), of those bacterial: 19/33, CMV 12/33; fungal 2/33).  Discontinuation of PP due to AE: 16/61 (26%)  Death 5/61 (8%), 3 due to incectious complications, 1/5 due to neoplasia,  Highest adverse events rates:  Combination of PP, RTX, high dose steroids: 37.5%  Combination of PP, high dose steroids: 25%  PP alone 20% |
| Fencl F, 2016 [137]  Czech republic | Recurrence of nephrotic proteinuria in children with focal segmental  glomerulosclerosis: early treatment with plasmapheresis and immunoadsorption  should be associated with better prognosis. | Retrospective case series | Nephrotic syndrome  FSGS  Recurrence  Plasmapheresis  Immunoadsorption | 5 | Children  Age at onset  Primary FSGS | Plasma exchange (PE)  Immunoadsorption (IA)  **Early treatment PE/IA**: within 3-7 days after diagnosis of recurrence  **vs.**  **Delayed treatment PE/IA**: 14 and 406 days after diagnosis of recurrenvce | Remission status:  Early PE/IA start:  Complete remission: 3/3 (100%) within 6 months  Sustained remission in long-term outcome  Stable graft function 2.8/ 9.7 and 3.8 yrs) after transplantation, all continued with PE/IA treatment  Delayed PE/IA start:  No sustained remission: 2/2, progressed to ESRD with graft loss 1 and 6.7 yrs after transplantation |
| **Immunoadsorption** | | | | | | | |
| Allard L, 2018 [138]  France | Treatment by immunoadsorption for recurrent focal segmental sclerosis after paediatric kidney transplantation: a multicentre French cohort study | Retrospective study  Multicenter (6) cohort study (France) | Focal segmental glomerulosclerosis  Immunoadsorption  Kidney transplantation  Nephrotic syndrome | 12 | Children (age 1-15 years)  Median age at SRNS diagnosis 4.8 yrs.  11/12 primary SRNS  Study period 01/2011 until 06/2014  Genetic forms and secondary forms of SRNS excluded  Median age of Tx: 10.5 (6.1-16) years  All deceased donor type  Median HLA MM score: 3 (0-4)  Induction treatment:  Basiliximab 9/12  ATG 3/12  Preventive treatments:  1/12 Rituximab 2/12 Plasma-pheresis  Median time to FSGS recurrence: 4.5 (0-21) days.  Median time of follow-up:  After Tx: 16 months  After 1st IA: 13 months | **Immunoadsorption (IA):**  IgA column (Immunosorba): 3/12  IgG column (Therasorb): 9/12  Median volume of plasma processed: 3 (1.7-6.2)  Mean % reduction of IgG: 68 (54-84) %  Early initiaiton of IA: 8/12 (without PE or in rescue of PE failure) Delayed initiation of IA: 4/12 (226-164 days)  PE before IA: 6/12 with CR 1/6 and PR 3/6  Median no. of IA sessions: 39 (10-111)  Time of IA therapy: 4 (1-16) months  **Concomitant treatments:**  Increased immunosuppressive therapies 12/12:  IV CSA: 8/12 Change of CNI: 3/12  Rituximab: 7/12  ACEis/ARB 12/12 | **Remission status:**  Complete rem.: 8/12 (67%)  Partial rem.: 2/12 (17%)  No rem.: 2/12 (17%)  3 months after IA:  Maintaining rem. without IA: 2/12  IA dependent: 8/12  No effect of IA: 2/12  No graft loss during study period  **Side effects:**  No severe reported  Anaphylactic reactions: 1/12 |
| **Lipidapheresis** | | | | | | | |
| Shah, 2019  [139]  U.S. and UK | LDL-apheresis-induced remission of focal segmental glomerulosclerosis recurrence in pediatric renal transplant recipients | Retrospective case series  4 pediatric centers | FSGS  Lipoprotein LDL-apheresis (LDL-A)  End-stage renal disease (ESRD)  Plasma exchange (PE)  Steroid-resistant nephrotic syndrome (SRNS) | 7 | Children  At at FSGS diagnosis: range 19 months-7 years  No genetic SRNS  DD 5/7  LRD 2/7  Immunosuppressive regimen:  Induction: thymoglobulin 4/7, Alemtuzumab 1/7, Basiliximab 2/7  Maintenance: CsA/Tac+MMF+Predn.  Immediate or early recurrence post-Tx: | **LDL-apheresis (LDL-A)**  (Liposorber® LA-15 in cmobination with pulse solumedrol)  Minimum of 9 weeks of LDL-A:  Exchange volume 60 mL/kg (3 < 9 weeks: 14-22 weeks)  First 3 weeks: 2-3 sessions per week  Time to LDL-A initiation post-Tx: range 1 week to 18 months  **Methylprednisolone pulses:**  10-10 mg/kg with a single max. Dose of 1g  Administered after 3 weeks auf LDL-A  **Pre-/Co-treatments:**  Plasmapheresis 7/7 (duration range: 1 week to 18 months)  Rituximab 6/7  Abatacept 1/7 | Remission status:  Complete remission: 4/7 (57%)  Partial remission; 3/7 (43%)  eGFR:  At LDL-A start: range 20.8-182.1 (mean 71.6) ml/min*1.73 m_2_  after LDL-A completion: range 81.8-169.3 (mean 119.4) ml/min*1.73 m^2^  All with eGFR improvement  No graft losses |
| **Leukocytapheresis** | | | | | | | |
| Shimizu M, 2010 [140],  Japan | Successful treatment of recurrent focal segmental glomerulosclerosis after renal transplantation by lymphozytapheresis and rituximab | Case report | FSGS  Recurrence  Renal transplantation  Rituximab  lymphocytapheresis | 1 | Age at 1st man.:  6 yrs  multidrug-resistant, treatment including LDL-apheressis  Age at ESRD: 13 yrs  Age at Tx: 15 yrs (LRD)  Immunsupressive regimen:  Induction: basiliximab, MP  Maintenance: Tac+MMF+Predn.  Late recurrence 16 months post-transplant | **Lymphocytapheresis** (LCAP, Cellsorba, Japan)  4 sessions (3000 ml of whole blooed were processed at a blood flow rate of 50 ml/min, duration 60 min)  **Methylprednisolone Pulses:**  500 mg/day over 3 days  **Rituximab:**  2 doses of 375 mg/m^2^ | Complete remission after treatment strategy  Outcome 24 months after treatment:  Normal graft function  No significant proteinuria |

**Table S6**

Semiquantitative expression of typical dipstick results (van der Watt, Ped Neph 7th ed. 2016):

| Dipstick results | Proteinuria |  |
| --- | --- | --- |
| Negative | 0 to <15 mg/dl |  |
| Trace | 15 to <30 mg/dl |  |
| 1+ | 30 to <100 mg/dl |  |
| 2+ | 100 to <300 mg/dl |  |
| 3+ | 300 to <1000 mg/dl |  |
| If 4+ | >/=1000 mg/dl |  |

**Table S7:** Extrarenal features associated with inherited forms of SRNS

| Genes | Inheritance | Syndrome | Extrarenal features |
| --- | --- | --- | --- |
| *WT1* | AD | Denys Drash  Frasier | Male pseudohermaphroditism (ambiguous genitalia, delayed puberty, primary amenorrhea)  Genitourinary abnormalities  Nephroblastoma, gonadablastoma  [141] |
| *LAMB2* | AR | Pierson syndrome | Ocular malformations (microcoria, cataracts, other lens or retinal abnormalities)  Neurologic symptoms (hypotonia, psychomotor retardation)  [142] |
| *WDR73*  *LAGE3*  *OSGEP*  *TP53RK*  *TPRKB*  *WDR4*  *NUP107*  *NUP133* | AR, except LAGE3 (X-linked) | Galloway-Mowat syndrome | Central nervous system involvement (microcephaly, structural brain anomalies, epilepsy)  Hiatus hernia  Facial dysmorphism  Skeletal abnormalities  [143] |
| *ITGA3* | AR | Interstitial lung disease, nephrotic syndrome, and epidermolysis bullosa | Interstitial lung disease  Epidermolysis bullosa  Renal hypodysplasia  [144] |
| *ITGB4* | AR | Epidermolysis bullosa, junctional, with pyloric atresia | Epidermolysis bullosa  Pyloric atresia  [145] |
| *PMM2*  *ALG1* | AR | Congenital disorder of glycosylation | Neurological involvement (seizures, neurological deterioration, microcephaly, cerebral or cerebellar atrophy)  Skeletal, cardiac, hepatic, gastrointestinal, endocrine, coagulation abnormalities  [146, 147] |
| *CD151* | AR |  | Pretibial epidermolysis bullosa  Neurosensory deafness  Nail and teeth dystrophy  [148] |
| *CRB2* | AR |  | Prenatal onset ventriculomegaly  Uretero-pelvic renal anomalies  Lung hypoplasia  Cardiac malformation  [149, 150] |
| *MTTL1* | Mitochondrial | MELAS | Neurological involvement (encephalomyopathy, ataxia, seizures, dystonia, mental retardation)  Sensorineural hearing loss  Retinopathy  Diabetes mellitus  Hypoparathyroidism  Lactic acidosis  [151] |
| *COQ2*  *COQ4*  *COQ6*  *COQ7*  *ADCK3*  *PDSS1*  *PDSS2* | AR | CoQ10 deficiency | Neurological involvement (encephalomyopathy, ataxia, seizures, dystonia, mental retardation)  Neurosensory deafness  [151] |
| *SGPL1* | AR | Nephrotic syndrome type 14 (NPHS14) | Adrenal insufficiency  Ichthyosis  Immunodeficiency  Neurologic and skeletal abnormalities  [152, 153] |
| *COL4A3* | AR | Alport's syndrome | Ocular abnormalities (anterior lenticonus, corneal and retinal lesions)  Neurosensory hearing loss  Leiomyomas*  [154] |
| *COL4A4* | AR |  |  |
| *COL4A5* | XD |  |  |
| *COL4A6** | XD |  |  |
| *INF2* | AD | Charcot–Marie–Tooth (with nephropathy) | Peripheral neuropathy (distal muscle atrophy and weakness)  Sensorineural hearing loss  [155] |
| *LMX1B* | AD | Nail patella syndrome | Absent or dystrophic nails (including triangular lunulae) and distal digital abnormalities  Limb and pelvic abnormalities (Absent or hypoplastic patella, elbow abnormalities, iliac flaring and iliac horns)  Sensorineural hearing loss  Eye abnormalities including glaucoma  [156, 157] |
| *SMARCAL1* | AR | Schimke immuno-osseous dysplasia | Short stature (prenatal growth deficiency)  Spondyloepiphyseal dysplasia (disproportionate shortening of the trunk, lumbar lordosis, characteristic radiographic bony anomalies)  Defective cellular immunity (Lymphopenia with decreased CD4+ number, and impaired T cell function)  Other hematologic abnormalities, including humoral defects and pancytopenia.  Lentigines especially on trunk  Corneal opacities and other ophthalmologic abnormalities  Arteriopathy with cerebral infarcts and/or ischemia  [158, 159] |
| *ZMPSTE24* | AR | Mandibuloacral dysplasia | Generalized lipoatrophy  Postnatal growth retardation  Craniofacial abnormalities (dysmorphic bird-like face)  Skeletal abnormalities (mandibular and clavicular hypoplasia, delayed cranial suture closure, acro-osteolysis, joint contractures)  Dental abnormalities  Restrictive dermatopathy, skin atrophy, alopecia  [160] |
| *SCARB2* | AR | Action mycoclonus- renal failure syndrome | Myoclonus epilepsy  Demyelinating polyneuropathy  Hearing loss  Dementia  Dilated cardiomyopathy  [161, 162] |
| *MYH9* | AD | Fechtner syndrome  Sebastian syndrome  Epstein syndrome | Congenital macrothrombocytopenia  Döhle-like inclusions (leukocyte inclusion bodies)  Sensorineural deafness  Cataracts  [163, 164] |

*In contiguous deletions involving *COL4A5* and *COL4*

**Table S8** Dosing Recommendations for the Initial Prescription of ACEi and ARBs in children with SRNS

| Drug | Age | | Initial Dose | Maximal Dose |
| --- | --- | --- | --- | --- |
| ACE inhibitors | | | | |
| *Contraindications: pregnancy, angioedema* | | | | |
| *Common adverse effects: cough, headache, dizziness, asthenia* | | | | |
| *Severe adverse effects: hyperkalemia, acute kidney injury, angioedema, fetal toxicity* | | | | |
| Benazepril | ≥6 y[^a^](https://pediatrics.aappublications.org/content/140/3/e20171904.long#fn-30) | | 0.2 mg/kg per d (up to 10 mg per d) | 0.6 mg/kg per d (up to 40 mg per d) |
|  |  |  |  |  |
| Captopril | Infants | | 0.05 mg/kg per dose | 6 mg/kg per d, daily to 4 times a day |
|  | Children | | 0.5 mg/kg per dose | 6 mg/kg per d, three times a day |
| Enalapril | ≥1 mo[^a^](https://pediatrics.aappublications.org/content/140/3/e20171904.long#fn-30) | | 0.08 mg/kg per d (up to 5 mg per d) | 0.6 mg/kg per d (up to 40 mg per d), daily to twice daily |
|  |  |  |  |  |
| Fosinopril | ≥6 y | | 0.1 mg/kg per d (up to 5 mg per d) | 40 mg per d |
|  | <50 kg | |  |  |
|  | ≥50 kg[^a^](https://pediatrics.aappublications.org/content/140/3/e20171904.long#fn-30) | | 5 mg per d | 40 mg per d |
| Lisinopril | ≥6 y[^a^](https://pediatrics.aappublications.org/content/140/3/e20171904.long#fn-30) | | 0.07 mg/kg per d (up to 5 mg per d) | 0.6 mg/kg per d (up to 40 mg per d) |
|  |  |  |  |  |
| Ramipril^b^ | — | | 1.6 mg/m^2^ per d | 6 mg/m^2^ per d |
| Quinapril | — | | 5 mg per d | 80 mg per d |
| ARBs^b^ | | | | |
| *Contraindications: pregnancy* | | | | |
| *Common adverse effects: headache, dizziness* | | | | |
| *Severe adverse effects: hyperkalemia, acute kidney injury, fetal toxicity* | | | | |
| Candesartan^b^ | | 1–5 y[^a^](https://pediatrics.aappublications.org/content/140/3/e20171904.long#fn-30) | 0.2 mg/kg per d (up to 4 mg per d) | 0.4 mg/kg per d (up to 16 mg per d) |
|  |  | ≥6 y[^a^](https://pediatrics.aappublications.org/content/140/3/e20171904.long#fn-30) |  | |
|  |  | <50 kg | 4 mg per d | 16 mg per d |
|  |  | ≥50 kg | 8 mg per d | 32 mg per d |
| Irbesartan^b^ | | 6–12 y | 75 mg per d | 150 mg per d |
|  |  | ≥13 | 150 mg per d | 300 mg per d |
| Losartan^b^ | | ≥6 y[^a^](https://pediatrics.aappublications.org/content/140/3/e20171904.long#fn-30) | 0.7 mg/kg (up to 50 mg) | 1.4 mg/kg (up to 100 mg) |
|  |  |  |  |  |
| Olmesartan^b^ | | ≥6 y[^a^](https://pediatrics.aappublications.org/content/140/3/e20171904.long#fn-30) | - | - |
|  |  | <35 kg | 10 mg | 20 mg |
|  |  | ≥35 kg | 20 mg | 40 mg |
| Valsartan^b^ | | ≥6 y[^a^](https://pediatrics.aappublications.org/content/140/3/e20171904.long#fn-30) | 1.3 mg/kg (up to 40 mg) | 2.7 mg/kg (up to 160 mg) |
|  |  |  |  |  |

Table adapted from Flynn JT et al., Pediatrics 2017 [165]

-, not applicable/available

^a^, FDA pediatric labeling

^b^, mainly metabolized by the liver, thus no dose reduction in patients with advanced CKD neccessary

**Table S9:** Primary outcome parameters in children with SRNS to be used for patient registries and studies

| Measures | Measurements |
| --- | --- |
| Complete remission of NS | Normal serum albumin (≥ 35 g/l) Proteinuria remission (UPr/Crea ratio < 20 mg/mmol (0.2 mg/mg) |
| Percentage (%) reduction of proteinuria | Reduction (40-50%) of UPr/Crea ratio since 1^st^ manifestation/ start of specific drug |
| Preservation of renal function | Absence of CKD: Normal creatinine, urea, cystatin C eGFR ≥ 90 ml/min*1,73 m^2^  Absence of secondary complications of CKD (anemia metabolic acidosis, renal osteodystrophy etc.) |
| Absence of disease-related complications | Normal blood pressure  Adequate hydration and avoidance of pre-renal acute kidney injury (AKI) Adequate growth and weight  Number of infections  No thromboembolic events  Normal lipid metabolism  Euthyroidism  Normal bone metabolism and bone mineral density |
| Absence of side effects of medications | Normal blood pressure Adequate growth and weight  Normal pubertal development  Normal blood glucose metabolism  Normal bone density  Normal ophthalmological evaluation  No hypertrichosis or gingival hyperplasia  No recurrent infections  No leukopenia/ pancytopenia  No gastrointestinal symptoms  No neurotoxicity (headaches, tremor) |
| Good Quality of Life | Absence of hospitalization Normal participation to life (school, activities, social life)  Activity level |

**Table S10**: Future Research Recommendations**:**

|  | **Non-genetic SRNS** | **Genetic SRNS** |
| --- | --- | --- |
| **SRNS treatment** | Define duration of initial treatment with oral steroids (4 wks vs. 6 wks) | - |
|  | Establish RCT for oral steroids +/- steroid pulses | - |
|  | RCT to establish the added value of CNI in addition to RAAS inhibition | - |
|  | Therapeutic drug monitoring: CsA and Tacrolimus trough levels, CsA C2 levels and AUC for MMF | - |
|  | Evaluate and define the duration of combined PDN+CNI treatment in SRNS | - |
|  | Evaluate the median response times to CNI | - |
|  | Evaluate management in CNI-responsive forms: when to discontinue CNI, if and when to add MMF or to switch to MMF | - |
|  | Evaluate Rituximab treatment regimens |  |
|  | Evaluate the use of other anti-CD20 antibodies | - |
|  | Evaluate the effectivity and safety of dual endothelin receptor and AR blockade compared to ARB alone in children with SRNS. | |
|  | Evaluate LDL-apheresis in MDRNS forms | |
|  | Define the risk/ benefit and estimates of efficacy for medical nephrectomy regimens | |
| **Pre-transplant** | Define personalized risk for recurrence at initial transplant | |
|  | Identify effective and safe transplant recurrence prevention and management options (including preventive plasmapheresis) | |
| **Post-transplant recurrence** | Determine risk of post-transplantat recurrence of FSGS for live related versus cadaveric donation to recipients with non-genetic SRNS | - |
|  | Evaluate current treatment strategies for post-transplant recurrence (plasmapheresis, cyclosporine A, tacrolimus, rituximab, cyclophosphamide) |  |
|  | Evaluate LDL-apheresis an post-transplant-recurrence |  |
| **Donor Evaluation** | - | Improve the evidence to support kidney donor candidacy in the setting of genetic SRNS |
|  | Develop better strategies and tools to screen donor candidates for genetic kidney diseases that consider the accuracy, efficiency and costs of testing, including assessment of targeted gene panels for known mutations implicated in kidney diseases. | |
|  | Determine how a genetic predisposition to various kidney diseases relates to outcomes after donation. Use genetic information along with other candidate characteristics to estimate the long-term risk of ESKD in the absence and presence of donation. | |
|  | Develop reliable imaging criteria to exclude ADPKD in donor candidates based on the results of the CT angiogram, which is frequently used to assess the renal vasculature as a routine part of the donor evaluation | |
|  | Define the role of *APOL1* genotyping in the evaluation of donor candidates of sub-Saharan African ancestry | |
|  | Define the role of APOL1 and R229Q genotyping in the evaluation of donor candidates of sub-Saharan African ancestry and with FSGS in recipient | |
|  | Acquire adequate data to assess rare outcomes such as ESRD in donors from national registries incorporating biospecimens and long-term outcomes information | |
|  | Evaluation of donors at risk of inherited kidney diseases to estimate their long-term risk of ESKD | |
| **Nephropathology** | Genotype-histology correlation | |
| **Transition** | Determine best transition practices | |
| **Health outcomes** | Evaluate patient-reported health outcomes involving treatment options | |

**Table S11: Management of salt intake in nephrotic children**

**Table S11-1**

| How to reduce your child's salt intake |
| --- |
| Eat home-prepared meals, using fresh ingredients, instead of canned, frozen, or packaged meals. |
| Don't use salt in cooking or at the table.  Cook with herbs and spices. |
| Prefer cereals with a low sodium content, i.e. < 300mg per 100g |
| Seasonings with the word "salt" in the name, such as garlic salt, are high in sodium.  When seasoning foods, use fresh or frozen herbs, garlic or garlic powder, use onion powder instead of onion salt, and try celery seed rather that celery salt. |
| Prefer mineral water with a low sodium content, i.e. equal or less than 20mg per liter. |

**Table S11-2:**

| Food with HIGH salt (sodium) content |  | Food with LOW salt (sodium) content |
| --- | --- | --- |
| Canned foods (vegetables, meats, pasta meals) |  | Plain breads, rice, and pasta (not dried pasta or rice mixes) |
| Processed foods (meats, such as sausage, bologna, pepperoni, salami, hot dogs |  | Vegetables and fruits (fresh or frozen) |
| Cheese |  | Meats (fresh cuts; not processed meats) |
| Dried pasta and rice mixes |  | Milk and yogurt (these tend to be moderate in sodium) |
| Soups (canned and dried) |  | Beverages, such as juices, tea, fruit drink or punch, and soda (sports drinks have sodium so these may need to be limited) |
| Snack foods (chips, popcorn, pretzels, cheese puffs, salted nuts) |  |  |
| Dips, sauces, and salad dressings  Ketchup, mustard, curry spices, soy, barbecue and cocktail sauce |  |  |
| Some cereals (e.g. corn flakes). Their sodium content should be less than 300mg per 100g. |  |  |
| Some mineral water. Their sodium content should be equal or less than 20mg per liter. |  |  |

Adapted from <https://www.stanfordchildrens.org/en/topic/default?id=nutrition-and-nephrotic-syndrome-90-P03099>.

Please notice that these recommendations do not substitute for advice from

a dietician based on the individual needs of the nephrotic child.

**Supplemental material: Text**

**Literature search**

The Pubmed database was searched for published clinical studies (randomized controlled trials (RCTs), prospective uncontrolled trials/ studies, observational studies, registry studies), systematic reviews and meta-analyses by using the main key words “steroid-resistant nephrotic syndrome” OR nephrotic syndrome AND children (until September 15, 2019), filtered for English published articles in humans.

The search identified a total of 927 articles with suitable articles. 223 articles are referenced in the clinical practice recommendation, 192 articles in the supplemental material. On demand, an extended additional literatur research was performed for the genetics of SRNS, post-transplant SRNS recurrence, complications of SRNS, CKD, histopathology.

**Diagnosis of SRNS**

*Rationale for definition of SRNS (initial treatment duration and dose of oral steroids) :*

There is no universal consensus on the diagnosis of SRNS due to variability in the minimum duration of corticosteroid exposure. The ISKDC reports that 95% of children with SSNS achieve remission within the first 4 weeks of daily corticosteroid therapy and an additional 3% after a further 4 weeks [166]. According to Kidney Disease Improving Global Outcomes (KDIGO) Clinical Practice Guideline for Glomerulonephritis, a minimum of 4 weeks treatment with corticosteroids is required to define steroid resistance [167]. This recommendation is based on the finding that 95% of children with SSNS will demonstrate resolution of proteinuria with 4 weeks of daily corticosteroid therapy and 100% after an additional 2-3 weeks of alternate-day therapy. However, prolonged courses of daily corticosteroids are associated with an increased incidence of side effects.

Two recent reports highlight the risk of underdosing with prednisone/prednisolone when the child is dosed by weight rather than body surface area, particularly in young children. The two methods differ in calculation amounting to increase in dose by 10-11 mg per dose [168, 169]. However, a recent RCT showed no differences in outcomes when comparing PDN dosing by weight or by BSA in children with steroid sensitive NS [170].

Moreover, a number of centers in developed countries employ i.v. MPDN pulses in patients not responding to oral PDN after 4 weeks.

Rationale on role of steroid pulses: There are controversial reports on the effectiveness of MPDN in the management of SRNS. Some studies report response to IV pulsed steroid therapy in patients non-responsive to conventional oral steroid therapy. Regimes of the pulsed steroid therapy differ, as do response rates (6-44%) ([171-176].

In low-resource countries, MPDN is expensive for the average population. Evidence-based results from a survey conducted among pediatric nephrologists in Nigeria showed that none used MPDN in the diagnosis of SRNS.

Most pediatric nephrologists consider a child steroid-resistant if no response is observed after 4 or 6 weeks of PDN at standard daily doses. The addition of i.v. methylprednisolone boluses is employed routinely in many centers after 4 weeks of no response.

*Definition of hypoalbuminemia*

The definition for nephrotic syndrome are in accordance with ISKDC [177] and included heavy proteinuria, ≥40 mg/m^2^/hour, and hypoalbuminemia, ≤ 3 g/dl.

*Renal histopathology diagnosis and reporting*

Minimal number of glomeruli defining a satisfactory biopsy:

- less than 5 glomeruli is considered inadequate, but even on such biopsies, a histopathological diagnosis of membranous nephropathy, IgA nephropathy and other entities with diffuse glomerular involvement can be made and inform treatment decisions.
- Since the histopathological diagnosis is unknown at the time of biopsy, pediatric nephrologists should aim for a biopsy sample containing at least 20 glomeruli is the minimum which is necessary to confidently exclude focal disease [178]
- Since FSGS shows a predilection for juxtamedullary glomeruli and diffuse mesangial sclerosis (DMS) for subcapsular glomeruli, detection of both diseases is facilitated by respective cortical sample.
- Work-up: Light microscopy on numbered level sections stained for hematoxylin-eosin, periodic-acid Schiff, methenamine silver or Jones and trichrome-elastic stains, and immunohistology for immunoglobulins and complement split products as well as transmission electron microscopy (TEM) is considered diagnostic standard on any native kidney biopsy [179]. Multiple level sections are key to confidently rule out FSGS involves only a minute fraction of the affected glomerulus [180]. If in doubt, the examination of pre-prepared blank sections spaced in regular intervals between the routinely stained, numbered sections or the examination of additional sections is very helpful to rule out or confirm FSGS with confidence. The additional value of ancillary immunohistological stainings such as synaptopodin, dystroglycan, Annexin A3, CD44 and CD80 has not been firmly established and is thus considered optional. TEM can rule out IgM, C1q glomerulopathy, glomerulonephritis; it is also vital to confirm the diffuse or subtotal foot process effacement typical of both MC and FSGS. Moreover, it can yield hints for the differentiation between primary, secondary and genetic FSGS and enables the diagnosis Alport syndrome.

Diagnosis and Reporting:

Any case of FSGS should be classified according to this Columbia classification as either collapsing, tip lesion, cellular, perihilar or not otherwise specified (NOS) [6]; based on sufficient reproducibility [181], this classification can indicate prognosis with the tip lesion variant having the best, the NOS variant having an intermediary and the collapsing variant having the worst prognosis [6, 182-184]. Histopathological findings should not be seen as absolute but should be weighed considering the clinical and genetical findings [185]. It has been reported that certain genetic forms of FSGS may show specific features on TEM, e.g. abnormal mitochondria in mitochondrial gene defects [186], dense cytoplasmic aggregates in ACTN4 mutations [187], INF2 with irregular foot processes [188] and Collagen type IV [189]; since neither the reproducibility of these findings on TEM, nor the specificity and sensitivity are known, full, unrestricted genetic testing should still be performed with suitable panels as listed above.

A diagnosis of MCD with foot-process effacement by TEM requires the exclusion of FSGS and other non-podocyte histopathological findings that could give alternative explanations for the foot process effacement. Glomeruli may or may not show mild mesangioproliferation. Immunohistology for immunoglobulins and complement is usually negative or, particularly IgM, mildly positive.

Diffuse mesangial sclerosis is defined on histological grounds as confluent mesangioproliferation in small glomerular tufts with concomitant matrix increase obliterating capillary lumina as FSGS and global glomerulosclerosis in a peculiar, cannonball-like pattern. On TEM, foot process effacement varies, no immune complexes are found. DMS can occur in syndromatic and isolated forms and is usually associated with genetic defects in WT1 [190], LAMB2 [191] or PLCE1 [192].

Patients with SRNS and, if available, their histological diagnosis or preferably with virtual slides and TEM grids should be included in national or international registries.

**References:**

1. Lieberman KV, Tejani A (1996) A randomized double-blind placebo-controlled trial of cyclosporine in steroid-resistant idiopathic focal segmental glomerulosclerosis in children. Journal of the American Society of Nephrology : JASN 7:56-63.

2. Ponticelli C (1993) Cyclosporine in idiopathic nephrotic syndrome. Immunopharmacology and immunotoxicology 15:479-489.

3. Garin EH, Orak JK, Hiott KL, Sutherland SE (1988) Cyclosporine therapy for steroid-resistant nephrotic syndrome. A controlled study. American journal of diseases of children (1960) 142:985-988.

4. Plank C, Kalb V, Hinkes B, Hildebrandt F, Gefeller O, Rascher W (2008) Cyclosporin A is superior to cyclophosphamide in children with steroid-resistant nephrotic syndrome-a randomized controlled multicentre trial by the Arbeitsgemeinschaft fur Padiatrische Nephrologie. Pediatric nephrology (Berlin, Germany) 23:1483-1493.

5. Gipson DS, Trachtman H, Kaskel FJ, Greene TH, Radeva MK, Gassman JJ, Moxey-Mims MM, Hogg RJ, Watkins SL, Fine RN, Hogan SL, Middleton JP, Vehaskari VM, Flynn PA, Powell LM, Vento SM, McMahan JL, Siegel N, D'Agati VD, Friedman AL (2011) Clinical trial of focal segmental glomerulosclerosis in children and young adults. Kidney international 80:868-878.

6. D'Agati VD, Alster JM, Jennette JC, Thomas DB, Pullman J, Savino DA, Cohen AH, Gipson DS, Gassman JJ, Radeva MK, Moxey-Mims MM, Friedman AL, Kaskel FJ, Trachtman H, Alpers CE, Fogo AB, Greene TH, Nast CC (2013) Association of histologic variants in FSGS clinical trial with presenting features and outcomes. Clinical journal of the American Society of Nephrology : CJASN 8:399-406.

7. Gipson DS, Trachtman H, Kaskel FJ, Radeva MK, Gassman J, Greene TH, Moxey-Mims MM, Hogg RJ, Watkins SL, Fine RN, Middleton JP, Vehaskari VM, Hogan SL, Vento S, Flynn PA, Powell LM, McMahan JL, Siegel N, Friedman AL (2011) Clinical trials treating focal segmental glomerulosclerosis should measure patient quality of life. Kidney international 79:678-685.

8. Hogg RJ, Portman RJ, Milliner D, Lemley KV, Eddy A, Ingelfinger J (2000) Evaluation and management of proteinuria and nephrotic syndrome in children: recommendations from a pediatric nephrology panel established at the National Kidney Foundation conference on proteinuria, albuminuria, risk, assessment, detection, and elimination (PARADE). Pediatrics 105:1242-1249.

9. Ferris M, Norwood V, Radeva M, Gassman JJ, Al-Uzri A, Askenazi D, Matoo T, Pinsk M, Sharma A, Smoyer W, Stults J, Vyas S, Weiss R, Gipson D, Kaskel F, Friedman A, Moxey-Mims M, Trachtman H (2013) Patient recruitment into a multicenter randomized clinical trial for kidney disease: report of the focal segmental glomerulosclerosis clinical trial (FSGS CT). Clinical and translational science 6:13-20.

10. Kopp JB, Winkler CA, Zhao X, Radeva MK, Gassman JJ, D'Agati VD, Nast CC, Wei C, Reiser J, Guay-Woodford LM, Pollak MR, Hildebrandt F, Moxey-Mims M, Gipson DS, Trachtman H, Friedman AL, Kaskel FJ (2015) Clinical Features and Histology of Apolipoprotein L1-Associated Nephropathy in the FSGS Clinical Trial. Journal of the American Society of Nephrology : JASN 26:1443-1448.

11. Choudhry S, Bagga A, Hari P, Sharma S, Kalaivani M, Dinda A (2009) Efficacy and safety of tacrolimus versus cyclosporine in children with steroid-resistant nephrotic syndrome: a randomized controlled trial. American journal of kidney diseases : the official journal of the National Kidney Foundation 53:760-769.

12. Gulati A, Sinha A, Gupta A, Kanitkar M, Sreenivas V, Sharma J, Mantan M, Agarwal I, Dinda AK, Hari P, Bagga A (2012) Treatment with tacrolimus and prednisolone is preferable to intravenous cyclophosphamide as the initial therapy for children with steroid-resistant nephrotic syndrome. Kidney international 82:1130-1135.

13. Sinha A, Gupta A, Kalaivani M, Hari P, Dinda AK, Bagga A (2017) Mycophenolate mofetil is inferior to tacrolimus in sustaining remission in children with idiopathic steroid-resistant nephrotic syndrome. Pediatric nephrology (Berlin, Germany) 92:248-257.

14. (1974) Prospective, controlled trial of cyclophosphamide therapy in children with nephrotic syndrome. Report of the International study of Kidney Disease in Children. Lancet (London, England) 2:423-427.

15. Tarshish P, Tobin JN, Bernstein J, Edelmann CM, Jr. (1996) Cyclophosphamide does not benefit patients with focal segmental glomerulosclerosis. A report of the International Study of Kidney Disease in Children. Pediatric nephrology (Berlin, Germany) 10:590-593.

16. Elhence R, Gulati S, Kher V, Gupta A, Sharma RK (1994) Intravenous pulse cyclophosphamide--a new regime for steroid-resistant minimal change nephrotic syndrome. Pediatric nephrology (Berlin, Germany) 8:1-3.

17. Mantan M, Sriram CS, Hari P, Dinda A, Bagga A (2008) Efficacy of intravenous pulse cyclophosphamide treatment versus combination of intravenous dexamethasone and oral cyclophosphamide treatment in steroid-resistant nephrotic syndrome. Pediatric nephrology (Berlin, Germany) 23:1495-1502.

18. Shah KM, Ohri AJ, Ali US (2017) A Randomized Controlled Trial of Intravenous versus Oral Cyclophosphamide in Steroid-resistant Nephrotic Syndrome in Children. Indian journal of nephrology 27:430-434.

19. Abramowicz M, Barnett HL, Edelmann CM, Jr., Greifer I, Kobayashi O, Arneil GC, Barron BA, Gordillo PG, Hallman N, Tiddens HA (1970) Controlled trial of azathioprine in children with nephrotic syndrome. A report for the international study of kidney disease in children. Lancet (London, England) 1:959-961.

20. Kleinknecht C, Broyer M, Gubler MC, Palcoux JB (1980) Irreversible renal failure after indomethacin in steroid-resistant nephrosis. The New England journal of medicine 302:691.

21. Wu B, Mao J, Shen H, Fu H, Wang J, Liu A, Gu W, Shu Q, Du L (2015) Triple immunosuppressive therapy in steroid-resistant nephrotic syndrome children with tacrolimus resistance or tacrolimus sensitivity but frequently relapsing. Nephrology (Carlton, Vic) 20:18-24.

22. Magnasco A, Ravani P, Edefonti A, Murer L, Ghio L, Belingheri M, Benetti E, Murtas C, Messina G, Massella L, Porcellini MG, Montagna M, Regazzi M, Scolari F, Ghiggeri GM (2012) Rituximab in children with resistant idiopathic nephrotic syndrome. Journal of the American Society of Nephrology : JASN 23:1117-1124.

23. Joy MS, Gipson DS, Powell L, MacHardy J, Jennette JC, Vento S, Pan C, Savin V, Eddy A, Fogo AB, Kopp JB, Cattran D, Trachtman H (2010) Phase 1 trial of adalimumab in Focal Segmental Glomerulosclerosis (FSGS): II. Report of the FONT (Novel Therapies for Resistant FSGS) study group. American journal of kidney diseases : the official journal of the National Kidney Foundation 55:50-60.

24. Joy MS, Gipson DS, Dike M, Powell L, Thompson A, Vento S, Eddy A, Fogo AB, Kopp JB, Cattran D, Trachtman H (2009) Phase I trial of rosiglitazone in FSGS: I. Report of the FONT Study Group. Clinical journal of the American Society of Nephrology : CJASN 4:39-47.

25. Ternant D, Paintaud G, Trachtman H, Gipson DS, Joy MS (2016) A possible influence of age on absorption and elimination of adalimumab in focal segmental glomerulosclerosis (FSGS). European journal of clinical pharmacology 72:253-255.

26. Peyser A, Machardy N, Tarapore F, Machardy J, Powell L, Gipson DS, Savin V, Pan C, Kump T, Vento S, Trachtman H (2010) Follow-up of phase I trial of adalimumab and rosiglitazone in FSGS: III. Report of the FONT study group. BMC nephrology 11:2.

27. Trachtman H, Vento S, Gipson D, Wickman L, Gassman J, Joy M, Savin V, Somers M, Pinsk M, Greene T (2011) Novel therapies for resistant focal segmental glomerulosclerosis (FONT) phase II clinical trial: study design. BMC nephrology 12:8.

28. Trachtman R, Sran SS, Trachtman H (2015) Recurrent focal segmental glomerulosclerosis after kidney transplantation. Pediatric nephrology (Berlin, Germany) 30:1793-1802.

29. Bagga A, Mudigoudar BD, Hari P, Vasudev V (2004) Enalapril dosage in steroid-resistant nephrotic syndrome. Pediatric nephrology (Berlin, Germany) 19:45-50.

30. Yi Z, Li Z, Wu XC, He QN, Dang XQ, He XJ (2006) Effect of fosinopril in children with steroid-resistant idiopathic nephrotic syndrome. Pediatric nephrology (Berlin, Germany) 21:967-972.

31. Trachtman H, Nelson P, Adler S, Campbell KN, Chaudhuri A, Derebail VK, Gambaro G, Gesualdo L, Gipson DS, Hogan J, Lieberman K, Marder B, Meyers KE, Mustafa E, Radhakrishnan J, Srivastava T, Stepanians M, Tesar V, Zhdanova O, Komers R (2018) DUET: A Phase 2 Study Evaluating the Efficacy and Safety of Sparsentan in Patients with FSGS. Journal of the American Society of Nephrology : JASN 29:2745-2754.

32. Komers R, Gipson DS, Nelson P, Adler S, Srivastava T, Derebail VK, Meyers KE, Pergola P, MacNally ME, Hunt JL, Shih A, Trachtman H (2017) Efficacy and Safety of Sparsentan Compared With Irbesartan in Patients With Primary Focal Segmental Glomerulosclerosis: Randomized, Controlled Trial Design (DUET). Kidney international reports 2:654-664.

33. Hari P, Khandelwal P, Smoyer WE (2019) Dyslipidemia and cardiovascular health in childhood nephrotic syndrome. Pediatric nephrology (Berlin, Germany).

34. Chongviriyaphan N, Tapaneya-Olarn C, Suthutvoravut U, Karnchanachumpol S, Chantraruksa V (1999) Effects of tuna fish oil on hyperlipidemia and proteinuria in childhood nephrotic syndrome. Journal of the Medical Association of Thailand = Chotmaihet thangphaet 82 Suppl 1:S122-128.

35. Trachtman H, Gipson DS, Somers M, Spino C, Adler S, Holzman L, Kopp JB, Sedor J, Overfield S, Elegbe A, Maldonado M, Greka A (2018) Randomized Clinical Trial Design to Assess Abatacept in Resistant Nephrotic Syndrome. Nature reviews Nephrology 3:115-121.

36. Trautmann A, Bodria M, Ozaltin F, Gheisari A, Melk A, Azocar M, Anarat A, Caliskan S, Emma F, Gellermann J, Oh J, Baskin E, Ksiazek J, Remuzzi G, Erdogan O, Akman S, Dusek J, Davitaia T, Ozkaya O, Papachristou F, Firszt-Adamczyk A, Urasinski T, Testa S, Krmar RT, Hyla-Klekot L, Pasini A, Ozcakar ZB, Sallay P, Cakar N, Galanti M, Terzic J, Aoun B, Caldas Afonso A, Szymanik-Grzelak H, Lipska BS, Schnaidt S, Schaefer F (2015) Spectrum of steroid-resistant and congenital nephrotic syndrome in children: the PodoNet registry cohort. Clinical journal of the American Society of Nephrology : CJASN 10:592-600.

37. Trautmann A, Schnaidt S, Lipska-Zietkiewicz BS, Bodria M, Ozaltin F, Emma F, Anarat A, Melk A, Azocar M, Oh J, Saeed B, Gheisari A, Caliskan S, Gellermann J, Higuita LMS, Jankauskiene A, Drozdz D, Mir S, Balat A, Szczepanska M, Paripovic D, Zurowska A, Bogdanovic R, Yilmaz A, Ranchin B, Baskin E, Erdogan O, Remuzzi G, Firszt-Adamczyk A, Kuzma-Mroczkowska E, Litwin M, Murer L, Tkaczyk M, Jardim H, Wasilewska A, Printza N, Fidan K, Simkova E, Borzecka H, Staude H, Hees K, Schaefer F (2017) Long-Term Outcome of Steroid-Resistant Nephrotic Syndrome in Children. Journal of the American Society of Nephrology : JASN 28:3055-3065.

38. Mekahli D, Liutkus A, Ranchin B, Yu A, Bessenay L, Girardin E, Van Damme-Lombaerts R, Palcoux JB, Cachat F, Lavocat MP, Bourdat-Michel G, Nobili F, Cochat P (2009) Long-term outcome of idiopathic steroid-resistant nephrotic syndrome: a multicenter study. Pediatric nephrology (Berlin, Germany) 24:1525-1532.

39. Buscher AK, Beck BB, Melk A, Hoefele J, Kranz B, Bamborschke D, Baig S, Lange-Sperandio B, Jungraithmayr T, Weber LT, Kemper MJ, Tonshoff B, Hoyer PF, Konrad M, Weber S (2016) Rapid Response to Cyclosporin A and Favorable Renal Outcome in Nongenetic Versus Genetic Steroid-Resistant Nephrotic Syndrome. Clinical journal of the American Society of Nephrology : CJASN 11:245-253.

40. Buscher AK, Kranz B, Buscher R, Hildebrandt F, Dworniczak B, Pennekamp P, Kuwertz-Broking E, Wingen AM, John U, Kemper M, Monnens L, Hoyer PF, Weber S, Konrad M (2010) Immunosuppression and renal outcome in congenital and pediatric steroid-resistant nephrotic syndrome. Clinical journal of the American Society of Nephrology : CJASN 5:2075-2084.

41. Ruf RG, Schultheiss M, Lichtenberger A, Karle SM, Zalewski I, Mucha B, Everding AS, Neuhaus T, Patzer L, Plank C, Haas JP, Ozaltin F, Imm A, Fuchshuber A, Bakkaloglu A, Hildebrandt F (2004) Prevalence of WT1 mutations in a large cohort of patients with steroid-resistant and steroid-sensitive nephrotic syndrome. Kidney international 66:564-570.

42. Gipson DS, Chin H, Presler TP, Jennette C, Ferris ME, Massengill S, Gibson K, Thomas DB (2006) Differential risk of remission and ESRD in childhood FSGS. Pediatric nephrology (Berlin, Germany) 21:344-349.

43. Abeyagunawardena AS, Sebire NJ, Risdon RA, Dillon MJ, Rees L, Van't Hoff W, Kumarasiri PV, Trompeter RS (2007) Predictors of long-term outcome of children with idiopathic focal segmental glomerulosclerosis. Pediatric nephrology (Berlin, Germany) 22:215-221.

44. Inaba A, Hamasaki Y, Ishikura K, Kaneko T (2016) Long-term outcome of idiopathic steroid-resistant nephrotic syndrome in children: response to comments. Pediatric nephrology (Berlin, Germany) 31:511-512.

45. Otukesh H, Otukesh S, Mojtahedzadeh M, Hoseini R, Fereshtehnejad SM, Riahi Fard A, Sadigh N, Heshmatzade Behzadi A, Javadi R, Hooman N, Mehrazma M (2009) Management and outcome of steroid-resistant nephrotic syndrome in children. Iranian journal of kidney diseases 3:210-217.

46. Straatmann C, Ayoob R, Gbadegesin R, Gibson K, Rheault MN, Srivastava T, Tran CL, Gipson DS, Greenbaum LA, Smoyer WE, Vehaskari VM (2013) Treatment outcome of late steroid-resistant nephrotic syndrome: a study by the Midwest Pediatric Nephrology Consortium. Pediatric nephrology (Berlin, Germany) 28:1235-1241.

47. Ehrich JH, Geerlings C, Zivicnjak M, Franke D, Geerlings H, Gellermann J (2007) Steroid-resistant idiopathic childhood nephrosis: overdiagnosed and undertreated. Nephrology, dialysis, transplantation : official publication of the European Dialysis and Transplant Association - European Renal Association 22:2183-2193.

48. Ghiggeri GM, Catarsi P, Scolari F, Caridi G, Bertelli R, Carrea A, Sanna-Cherchi S, Emma F, Allegri L, Cancarini G, Rizzoni GF, Perfumo F (2004) Cyclosporine in patients with steroid-resistant nephrotic syndrome: an open-label, nonrandomized, retrospective study. Clinical therapeutics 26:1411-1418.

49. Hamasaki Y, Yoshikawa N, Hattori S, Sasaki S, Iijima K, Nakanishi K, Matsuyama T, Ishikura K, Yata N, Kaneko T, Honda M (2009) Cyclosporine and steroid therapy in children with steroid-resistant nephrotic syndrome. Pediatric nephrology (Berlin, Germany) 24:2177-2185.

50. Tahar G, Rachid LM (2010) Cyclosporine A and steroid therapy in childhood steroid-resistant nephrotic syndrome. International journal of nephrology and renovascular disease 3:117-121.

51. Liu Y, Yang R, Yang C, Dong S, Zhu Y, Zhao M, Yuan F, Gui K (2018) Cyclophosphamide versus cyclosporine A therapy in steroid-resistant nephrotic syndrome: a retrospective study with a mean 5-year follow-up. The Journal of international medical research 46:4506-4517.

52. Ingulli E, Singh A, Baqi N, Ahmad H, Moazami S, Tejani A (1995) Aggressive, long-term cyclosporine therapy for steroid-resistant focal segmental glomerulosclerosis. Journal of the American Society of Nephrology : JASN 5:1820-1825.

53. El-Husseini A, El-Basuony F, Mahmoud I, Sheashaa H, Sabry A, Hassan R, Taha N, Hassan N, Sayed-Ahmad N, Sobh M (2005) Long-term effects of cyclosporine in children with idiopathic nephrotic syndrome: a single-centre experience. Nephrology, dialysis, transplantation : official publication of the European Dialysis and Transplant Association - European Renal Association 20:2433-2438.

54. Klaassen I, Ozgoren B, Sadowski CE, Moller K, van Husen M, Lehnhardt A, Timmermann K, Freudenberg F, Helmchen U, Oh J, Kemper MJ (2015) Response to cyclosporine in steroid-resistant nephrotic syndrome: discontinuation is possible. Pediatric nephrology (Berlin, Germany) 30:1477-1483.

55. Butani L, Ramsamooj R (2009) Experience with tacrolimus in children with steroid-resistant nephrotic syndrome. Pediatric nephrology (Berlin, Germany) 24:1517-1523.

56. Wang W, Xia Y, Mao J, Chen Y, Wang D, Shen H, Fu H, Du L, Liu A (2012) Treatment of tacrolimus or cyclosporine A in children with idiopathic nephrotic syndrome. Pediatric nephrology (Berlin, Germany) 27:2073-2079.

57. Roberti I, Vyas S (2010) Long-term outcome of children with steroid-resistant nephrotic syndrome treated with tacrolimus. Pediatric nephrology (Berlin, Germany) 25:1117-1124.

58. Jahan A, Prabha R, Chaturvedi S, Mathew B, Fleming D, Agarwal I (2015) Clinical efficacy and pharmacokinetics of tacrolimus in children with steroid-resistant nephrotic syndrome. Pediatric nephrology (Berlin, Germany) 30:1961-1967.

59. Gulati S, Prasad N, Sharma RK, Kumar A, Gupta A, Baburaj VP (2008) Tacrolimus: a new therapy for steroid-resistant nephrotic syndrome in children. Nephrology, dialysis, transplantation : official publication of the European Dialysis and Transplant Association - European Renal Association 23:910-913.

60. Loeffler K, Gowrishankar M, Yiu V (2004) Tacrolimus therapy in pediatric patients with treatment-resistant nephrotic syndrome. Pediatric nephrology (Berlin, Germany) 19:281-287.

61. Shenoy M, Plant ND, Lewis MA, Bradbury MG, Lennon R, Webb NJ (2010) Intravenous methylprednisolone in idiopathic childhood nephrotic syndrome. Pediatric nephrology (Berlin, Germany) 25:899-903.

62. Zhang H, Wang Z, Dong LQ, Guo YN (2016) Children with Steroid-resistant Nephrotic Syndrome: Long-term Outcomes of Sequential Steroid Therapy. Biomedical and environmental sciences : BES 29:650-655.

63. Rennert WP, Kala UK, Jacobs D, Goetsch S, Verhaart S (1999) Pulse cyclophosphamide for steroid-resistant focal segmental glomerulosclerosis. Pediatric nephrology (Berlin, Germany) 13:113-116.

64. de Mello VR, Rodrigues MT, Mastrocinque TH, Martins SP, de Andrade OV, Guidoni EB, Scheffer DK, Martini Filho D, Toporovski J, Benini V (2010) Mycophenolate mofetil in children with steroid/cyclophosphamide-resistant nephrotic syndrome. Pediatric nephrology (Berlin, Germany) 25:453-460.

65. Li Z, Duan C, He J, Wu T, Xun M, Zhang Y, Yin Y (2010) Mycophenolate mofetil therapy for children with steroid-resistant nephrotic syndrome. Pediatric nephrology (Berlin, Germany) 25:883-888.

66. Montane B, Abitbol C, Chandar J, Strauss J, Zilleruelo G (2003) Novel therapy of focal glomerulosclerosis with mycophenolate and angiotensin blockade. Pediatric nephrology (Berlin, Germany) 18:772-777.

67. Gargah TT, Lakhoua MR (2011) Mycophenolate mofetil in treatment of childhood steroid-resistant nephrotic syndrome. Journal of nephrology 24:203-207.

68. Barletta GM, Smoyer WE, Bunchman TE, Flynn JT, Kershaw DB (2003) Use of mycophenolate mofetil in steroid-dependent and -resistant nephrotic syndrome. Pediatric nephrology (Berlin, Germany) 18:833-837.

69. Gellermann J, Ehrich JH, Querfeld U (2012) Sequential maintenance therapy with cyclosporin A and mycophenolate mofetil for sustained remission of childhood steroid-resistant nephrotic syndrome. Nephrology, dialysis, transplantation : official publication of the European Dialysis and Transplant Association - European Renal Association 27:1970-1978.

70. Kim J, Patnaik N, Chorny N, Frank R, Infante L, Sethna C (2014) Second-line immunosuppressive treatment of childhood nephrotic syndrome: a single-center experience. Nephron extra 4:8-17.

71. Liern M, De Reyes V, Fayad A, Vallejo G (2012) Use of sirolimus in patients with primary steroid-resistant nephrotic syndrome. Nefrologia : publicacion oficial de la Sociedad Espanola Nefrologia 32:321-328.

72. Almeida MP, Almeida HA, Rosa FC (1994) Vincristine in steroid-resistant nephrotic syndrome. Pediatric nephrology (Berlin, Germany) 8:79-80.

73. Goonasekera CD, Koziell AB, Hulton SA, Dillon MJ (1998) Vincristine and focal segmental sclerosis: do we need a multicentre trial? Pediatric nephrology (Berlin, Germany) 12:284-289.

74. Krishnan RG, Coulthard MG, Moghal NE (2006) Is there a role for vincristine in nephrotic syndrome? Pediatric nephrology (Berlin, Germany) 21:597.

75. Thalgahagoda S, Abeyagunawardena S, Jayaweera H, Karunadasa UI, Abeyagunawardena AS (2017) Pulsed Vincristine Therapy in Steroid-Resistant Nephrotic Syndrome 2017:1757940.

76. Saito T, Iwano M, Matsumoto K, Mitarai T, Yokoyama H, Yorioka N, Nishi S, Yoshimura A, Sato H, Ogahara S, Sasatomi Y, Kataoka Y, Ueda S, Koyama A, Maruyama S, Nangaku M, Imai E, Matsuo S, Tomino Y (2017) Mizoribine therapy combined with steroids and mizoribine blood concentration monitoring for idiopathic membranous nephropathy with steroid-resistant nephrotic syndrome. Clinical and experimental nephrology 21:961-970.

77. Bagga A, Sinha A, Moudgil A (2007) Rituximab in patients with the steroid-resistant nephrotic syndrome. The New England journal of medicine 356:2751-2752.

78. Gulati A, Sinha A, Jordan SC, Hari P, Dinda AK, Sharma S, Srivastava RN, Moudgil A, Bagga A (2010) Efficacy and safety of treatment with rituximab for difficult steroid-resistant and -dependent nephrotic syndrome: multicentric report. Clinical journal of the American Society of Nephrology : CJASN 5:2207-2212.

79. Nakayama M, Kamei K, Nozu K, Matsuoka K, Nakagawa A, Sako M, Iijima K (2008) Rituximab for refractory focal segmental glomerulosclerosis. Pediatric nephrology (Berlin, Germany) 23:481-485.

80. Sinha A, Bhatia D, Gulati A, Rawat M, Dinda AK, Hari P, Bagga A (2015) Efficacy and safety of rituximab in children with difficult-to-treat nephrotic syndrome. Nephrology, dialysis, transplantation : official publication of the European Dialysis and Transplant Association - European Renal Association 30:96-106.

81. Kamei K, Okada M, Sato M, Fujimaru T, Ogura M, Nakayama M, Kaito H, Iijima K, Ito S (2014) Rituximab treatment combined with methylprednisolone pulse therapy and immunosuppressants for childhood steroid-resistant nephrotic syndrome. Pediatric nephrology (Berlin, Germany) 29:1181-1187.

82. Ito S, Kamei K, Ogura M, Udagawa T, Fujinaga S, Saito M, Sako M, Iijima K (2013) Survey of rituximab treatment for childhood-onset refractory nephrotic syndrome. Pediatric nephrology (Berlin, Germany) 28:257-264.

83. Fujinaga S, Sakuraya K (2018) Single infusion of low-dose ofatumumab in a child with complicated nephrotic syndrome with anti-rituximab antibodies. Pediatric nephrology (Berlin, Germany) 33:527-528.

84. Sun L, Xu H, Shen Q, Cao Q, Rao J, Liu HM, Fang XY, Zhou LJ (2014) Efficacy of rituximab therapy in children with refractory nephrotic syndrome: a prospective observational study in Shanghai. World journal of pediatrics : WJP 10:59-63.

85. Zachwieja J, Silska-Dittmar M (2018) Multicenter analysis of the efficacy and safety of a non-standard immunosuppressive therapy with rituximab in children with steroid-resistant nephrotic syndrome.

86. Kari JA, El-Morshedy SM, El-Desoky S, Alshaya HO, Rahim KA, Edrees BM (2011) Rituximab for refractory cases of childhood nephrotic syndrome. Pediatric nephrology (Berlin, Germany) 26:733-737.

87. Basu B, Mahapatra TK, Mondal N (2015) Mycophenolate Mofetil Following Rituximab in Children With Steroid-Resistant Nephrotic Syndrome. Pediatrics 136:e132-139.

88. Vivarelli M, Colucci M, Bonanni A, Verzani M, Serafinelli J, Emma F, Ghiggeri G (2017) Ofatumumab in two pediatric nephrotic syndrome patients allergic to rituximab. Pediatric nephrology (Berlin, Germany) 32:181-184.

89. Basu B (2014) Ofatumumab for rituximab-resistant nephrotic syndrome. The New England journal of medicine 370:1268-1270.

90. Trachtman H, Fervenza FC, Gipson DS, Heering P, Jayne DR, Peters H, Rota S, Remuzzi G, Rump LC, Sellin LK, Heaton JP, Streisand JB, Hard ML, Ledbetter SR, Vincenti F (2011) A phase 1, single-dose study of fresolimumab, an anti-TGF-beta antibody, in treatment-resistant primary focal segmental glomerulosclerosis. Kidney international 79:1236-1243.

91. Yu CC, Fornoni A, Weins A, Hakroush S, Maiguel D, Sageshima J, Chen L, Ciancio G, Faridi MH, Behr D, Campbell KN, Chang JM, Chen HC, Oh J, Faul C, Arnaout MA, Fiorina P, Gupta V, Greka A, Burke GW, 3rd, Mundel P (2013) Abatacept in B7-1-positive proteinuric kidney disease. The New England journal of medicine 369:2416-2423.

92. Bonanni A, Bertelli R, Rossi R, Bruschi M, Di Donato A, Ravani P, Ghiggeri GM (2015) A Pilot Study of IL2 in Drug-Resistant Idiopathic Nephrotic Syndrome. PloS one 10:e0138343.

93. Sgambat K, Banks M, Moudgil A (2013) Effect of galactose on glomerular permeability and proteinuria in steroid-resistant nephrotic syndrome. Pediatric nephrology (Berlin, Germany) 28:2131-2135.

94. Imaizumi T, Kawasaki Y, Matsuura H, Matsumoto A, Takano K, Suyama K, Hashimoto K, Suzuki H, Hosoya M (2007) Efficacy of steroid pulse, plasmapheresis, and mizoribine in a patient with focal segmental glomerulosclerosis. Pediatric nephrology (Berlin, Germany) 22:1215-1218.

95. Oliveira L, Wang D, McCormick BB (2007) A case report of plasmapheresis and cyclophosphamide for steroid-resistant focal segmental glomerulosclerosis: recovery of renal function after five months on dialysis. Therapeutic apheresis and dialysis : official peer-reviewed journal of the International Society for Apheresis, the Japanese Society for Apheresis, the Japanese Society for Dialysis Therapy 11:227-231.

96. Paglialonga F, Schmitt CP, Shroff R, Vondrak K, Aufricht C, Watson AR, Ariceta G, Fischbach M, Klaus G, Holtta T, Bakkaloglu SA, Zurowska A, Jankauskiene A, Vande Walle J, Schaefer B, Wright E, Connell R, Edefonti A (2015) Indications, technique, and outcome of therapeutic apheresis in European pediatric nephrology units. Pediatric nephrology (Berlin, Germany) 30:103-111.

97. Skalova S, Podhola M, Vondrak K, Chernin G (2010) Plasmapheresis-induced clinical improvement in a patient with steroid-resistant nephrotic syndrome due to podocin (NPHS2) gene mutation. Acta medica (Hradec Kralove) 53:157-159.

98. Vecsei AK, Muller T, Schratzberger EC, Kircher K, Regele H, Arbeiter K, Schroth B, Aufricht C (2001) Plasmapheresis-induced remission in otherwise therapy-resistant FSGS. Pediatric nephrology (Berlin, Germany) 16:898-900.

99. Verghese PS, Rheault MN, Jackson S, Matas AJ, Chinnakotla S, Chavers B (2018) The effect of peri-transplant plasmapheresis in the prevention of recurrent FSGS. Pediatric transplantation 22:e13154.

100. Gohh RY, Yango AF, Morrissey PE, Monaco AP, Gautam A, Sharma M, McCarthy ET, Savin VJ (2005) Preemptive plasmapheresis and recurrence of FSGS in high-risk renal transplant recipients. American journal of transplantation : official journal of the American Society of Transplantation and the American Society of Transplant Surgeons 5:2907-2912.

101. Gonzalez E, Ettenger R, Rianthavorn P, Tsai E, Malekzadeh M (2011) Preemptive plasmapheresis and recurrence of focal segmental glomerulosclerosis in pediatric renal transplantation. Pediatric transplantation 15:495-501.

102. Kuhn C, Kuhn A, Markau S, Kastner U, Osten B (2006) Effect of immunoadsorption on refractory idiopathic focal and segmental glomerulosclerosis. Journal of clinical apheresis 21:266-270.

103. Muso E, Mune M, Fujii Y, Imai E, Ueda N, Hatta K, Imada A, Takemura T, Miki S, Kuwahara T, Takamitsu Y, Tsubakihara Y (2001) Significantly rapid relief from steroid-resistant nephrotic syndrome by LDL apheresis compared with steroid monotherapy. Nephron 89:408-415.

104. Muso E, Mune M, Hirano T, Hattori M, Kimura K, Watanabe T, Yokoyama H, Sato H, Uchida S, Wada T, Shoji T, Yuzawa Y, Takemura T, Sugiyama S, Nishizawa Y, Ogahara S, Yorioka N, Sakai S, Ogura Y, Yukawa S, Iino Y, Imai E, Matsuo S, Saito T (2015) Immediate therapeutic efficacy of low-density lipoprotein apheresis for drug-resistant nephrotic syndrome: evidence from the short-term results from the POLARIS Study. Clinical and experimental nephrology 19:379-386.

105. Hattori M, Ito K, Kawaguchi H, Tanaka T, Kubota R, Khono M (1993) Treatment with a combination of low-density lipoprotein aphaeresis and pravastatin of a patient with drug-resistant nephrotic syndrome due to focal segmental glomerulosclerosis. Pediatric nephrology (Berlin, Germany) 7:196-198.

106. Kawasaki Y, Suzuki S, Matsumoto A, Takano K, Suyama K, Hashimoto K, Suzuki J, Suzuki H, Hosoya M (2007) Long-term efficacy of low-density lipoprotein apheresis for focal and segmental glomerulosclerosis. Pediatric nephrology (Berlin, Germany) 22:889-892.

107. Oto J, Suga K, Matsuura S, Kondo S, Ohnishi Y, Inui D, Imanaka H, Kagami S, Nishimura M (2009) Low-density lipoprotein apheresis in a pediatric patient with refractory nephrotic syndrome due to focal segmental glomerulosclerosis. Journal of anesthesia 23:284-287.

108. Hattori M, Chikamoto H, Akioka Y, Nakakura H, Ogino D, Matsunaga A, Fukazawa A, Miyakawa S, Khono M, Kawaguchi H, Ito K (2003) A combined low-density lipoprotein apheresis and prednisone therapy for steroid-resistant primary focal segmental glomerulosclerosis in children. American journal of kidney diseases : the official journal of the National Kidney Foundation 42:1121-1130.

109. Takakura M, Shimizu M, Mizuta M, Inoue N, Tasaki Y, Ohta K, Furuichi K, Wada T, Yachie A (2018) Successful treatment of rituximab- and steroid-resistant nephrotic syndrome with leukocytapheresis. Journal of clinical apheresis 33:409-411.

110. Francis A, Didsbury M, McCarthy H, Kara T (2018) Treatment of recurrent focal segmental glomerulosclerosis post-kidney transplantation in Australian and New Zealand children: A retrospective cohort study. Pediatric transplantation 22:e13185.

111. Morello W, Puvinathan S, Puccio G, Ghiggeri GM, Dello Strologo L, Peruzzi L, Murer L, Cioni M, Guzzo I, Cocchi E, Benetti E, Testa S, Ghio L, Caridi G, Cardillo M, Torelli R, Montini G (2019) Post-transplant recurrence of steroid resistant nephrotic syndrome in children: the Italian experience.

112. Shishido S, Satou H, Muramatsu M, Hamasaki Y, Ishikura K, Hataya H, Honda M, Asanuma H, Aikawa A (2013) Combination of pulse methylprednisolone infusions with cyclosporine-based immunosuppression is safe and effective to treat recurrent focal segmental glomerulosclerosis after pediatric kidney transplantation. Clinical transplantation 27:E143-150.

113. Salomon R, Gagnadoux MF, Niaudet P (2003) Intravenous cyclosporine therapy in recurrent nephrotic syndrome after renal transplantation in children. Transplantation 75:810-814.

114. Raafat RH, Kalia A, Travis LB, Diven SC (2004) High-dose oral cyclosporin therapy for recurrent focal segmental glomerulosclerosis in children. American journal of kidney diseases : the official journal of the National Kidney Foundation 44:50-56.

115. Cochat P, Kassir A, Colon S, Glastre C, Tourniaire B, Parchoux B, Martin X, David L (1993) Recurrent nephrotic syndrome after transplantation: early treatment with plasmaphaeresis and cyclophosphamide. Pediatric nephrology (Berlin, Germany) 7:50-54.

116. Cheong HI, Han HW, Park HW, Ha IS, Han KS, Lee HS, Kim SJ, Choi Y (2000) Early recurrent nephrotic syndrome after renal transplantation in children with focal segmental glomerulosclerosis. Nephrology, dialysis, transplantation : official publication of the European Dialysis and Transplant Association - European Renal Association 15:78-81.

117. Dall'Amico R, Ghiggeri G, Carraro M, Artero M, Ghio L, Zamorani E, Zennaro C, Basile G, Montini G, Rivabella L, Cardillo M, Scalamogna M, Ginevri F (1999) Prediction and treatment of recurrent focal segmental glomerulosclerosis after renal transplantation in children. American journal of kidney diseases : the official journal of the National Kidney Foundation 34:1048-1055.

118. Sethna C, Benchimol C, Hotchkiss H, Frank R, Infante L, Vento S, Trachtman H (2011) Treatment of recurrent focal segmental glomerulosclerosis in pediatric kidney transplant recipients: effect of rituximab. Journal of transplantation 2011:389542.

119. Kumar J, Shatat IF, Skversky AL, Woroniecki RP, Del Rio M, Perelstein EM, Johnson VL, Mahesh S (2013) Rituximab in post-transplant pediatric recurrent focal segmental glomerulosclerosis. Pediatric nephrology (Berlin, Germany) 28:333-338.

120. Garrouste C, Canaud G, Buchler M, Rivalan J, Colosio C, Martinez F, Aniort J, Dudreuilh C, Pereira B, Caillard S, Philipponnet C, Anglicheau D, Heng AE (2017) Rituximab for Recurrence of Primary Focal Segmental Glomerulosclerosis After Kidney Transplantation: Clinical Outcomes. Transplantation 101:649-656.

121. Alhasan KA, Alherbish A, Osman A, Kari JA, Almojalli H (2019) Successful Treatment of Recurrent Focal Segmental Glomerulosclerosis After Transplantation in Children: A Single-Center Experience. Transplantation proceedings 51:517-521.

122. Alasfar S, Matar D, Montgomery RA, Desai N, Lonze B, Vujjini V, Estrella MM, Manllo Dieck J, Khneizer G, Sever S, Reiser J, Alachkar N (2018) Rituximab and Therapeutic Plasma Exchange in Recurrent Focal Segmental Glomerulosclerosis Postkidney Transplantation. Transplantation 102:e115-e120.

123. Bernard J, Bruel A, Allain-Launay E, Dantal J, Roussey G (2018) Ofatumumab in post-transplantation recurrence of a pediatric steroid-resistant idiopathic nephrotic syndrome. Pediatric transplantation 22:e13175.

124. Solomon S, Zolotnitskaya A, Del Rio M (2019) Ofatumumab in post-transplantation recurrence of focal segmental glomerulosclerosis in a child. Pediatric transplantation 23:e13413.

125. Colucci M, Labbadia R, Vivarelli M, Camassei FD, Emma F, Dello Strologo L (2019) Ofatumumab rescue treatment in post-transplant recurrence of focal segmental glomerulosclerosis. Pediatric nephrology (Berlin, Germany).

126. Delville M, Baye E, Durrbach A, Audard V, Kofman T, Braun L, Olagne J, Nguyen C, Deschenes G, Moulin B, Delahousse M, Kesler-Roussey G, Beaudreuil S, Martinez F, Rabant M, Grimbert P, Gallazzini M, Terzi F, Legendre C, Canaud G (2016) B7-1 Blockade Does Not Improve Post-Transplant Nephrotic Syndrome Caused by Recurrent FSGS. Journal of the American Society of Nephrology : JASN 27:2520-2527.

127. Alkandari O, Nampoory N, Nair P, Atta A, Zakaria Z, Mossad A, Yagan J, Al-Otaibi T (2016) Recurrent Focal Segmental Glomerulosclerosis and Abatacept: Case Report. Experimental and clinical transplantation : official journal of the Middle East Society for Organ Transplantation 14:456-459.

128. Leroy S, Guigonis V, Bruckner D, Emal-Aglae V, Deschenes G, Bensman A, Ulinski T (2009) Successful anti-TNFalpha treatment in a child with posttransplant recurrent focal segmental glomerulosclerosis. American journal of transplantation : official journal of the American Society of Transplantation and the American Society of Transplant Surgeons 9:858-861.

129. Kashgary A, Sontrop JM, Li L, Al-Jaishi AA, Habibullah ZN, Alsolaimani R, Clark WF (2016) The role of plasma exchange in treating post-transplant focal segmental glomerulosclerosis: A systematic review and meta-analysis of 77 case-reports and case-series. BMC nephrology 17:104.

130. Ohta T, Kawaguchi H, Hattori M, Komatsu Y, Akioka Y, Nagata M, Shiraga H, Ito K, Takahashi K, Ishikawa N, Tanabe K, Yamaguchi Y, Ota K (2001) Effect of pre-and postoperative plasmapheresis on posttransplant recurrence of focal segmental glomerulosclerosis in children. Transplantation 71:628-633.

131. Hickson LJ, Gera M, Amer H, Iqbal CW, Moore TB, Milliner DS, Cosio FG, Larson TS, Stegall MD, Ishitani MB, Gloor JM, Griffin MD (2009) Kidney transplantation for primary focal segmental glomerulosclerosis: outcomes and response to therapy for recurrence. Transplantation 87:1232-1239.

132. Araya CE, Dharnidharka VR (2011) The factors that may predict response to rituximab therapy in recurrent focal segmental glomerulosclerosis: a systematic review. Journal of transplantation 2011:374213.

133. Garcia CD, Bittencourt VB, Tumelero A, Antonello JS, Malheiros D, Garcia VD (2006) Plasmapheresis for recurrent posttransplant focal segmental glomerulosclerosis. Transplantation proceedings 38:1904-1905.

134. Fuentes GM, Meseguer CG, Carrion AP, Hijosa MM, Garcia-Pose A, Melgar AA, Torres MN (2010) Long-term outcome of focal segmental glomerulosclerosis after pediatric renal transplantation. Pediatric nephrology (Berlin, Germany) 25:529-534.

135. Straatmann C, Kallash M, Killackey M, Iorember F, Aviles D, Bamgbola O, Carson T, Florman S, Vehaskari MV (2014) Success with plasmapheresis treatment for recurrent focal segmental glomerulosclerosis in pediatric renal transplant recipients. Pediatric transplantation 18:29-34.

136. Mansur JB, Sandes-Freitas TV, Kirsztajn GM, Cristelli MP, Mata GF, de Paula MI, Grenzi PC, Martins SBS, Felipe CR, Tedesco-Silva H, Pestana JOM (2019) Clinical features and outcomes of kidney transplant recipients with focal segmental glomerulosclerosis recurrence. Nephrology (Carlton, Vic) 24:1179-1188.

137. Fencl F, Vondrak K, Rosik T, Zieg J, Chadimova M, Hacek J, Dusek J, Seeman T (2016) Recurrence of nephrotic proteinuria in children with focal segmental glomerulosclerosis: early treatment with plasmapheresis and immunoadsorption should be associated with better prognosis. Minerva pediatrica 68:348-354.

138. Allard L, Kwon T, Krid S, Bacchetta J, Garnier A, Novo R, Deschenes G, Salomon R, Roussey G, Allain-Launay E (2018) Treatment by immunoadsorption for recurrent focal segmental glomerulosclerosis after paediatric kidney transplantation: a multicentre French cohort. Nephrology, dialysis, transplantation : official publication of the European Dialysis and Transplant Association - European Renal Association 33:954-963.

139. Shah L, Hooper DK, Okamura D, Wallace D, Moodalbail D, Gluck C, Koziell A, Zaritsky JJ (2019) LDL-apheresis-induced remission of focal segmental glomerulosclerosis recurrence in pediatric renal transplant recipients. Pediatric nephrology (Berlin, Germany).

140. Shimizu M, Kitagawa K, Nishio S, Yokoyama T, Furuichi K, Ohta K, Wada T, Yachie A (2010) Successful treatment of recurrent focal segmental glomerulosclerosis after renal transplantation by lymphocytapheresis and rituximab. Transplant international : official journal of the European Society for Organ Transplantation 23:e53-55.

141. Lipska BS, Ranchin B, Iatropoulos P, Gellermann J, Melk A, Ozaltin F, Caridi G, Seeman T, Tory K, Jankauskiene A, Zurowska A, Szczepanska M, Wasilewska A, Harambat J, Trautmann A, Peco-Antic A, Borzecka H, Moczulska A, Saeed B, Bogdanovic R, Kalyoncu M, Simkova E, Erdogan O, Vrljicak K, Teixeira A, Azocar M, Schaefer F (2014) Genotype-phenotype associations in WT1 glomerulopathy. Kidney international 85:1169-1178.

142. Matejas V, Hinkes B, Alkandari F, Al-Gazali L, Annexstad E, Aytac MB, Barrow M, Blahova K, Bockenhauer D, Cheong HI, Maruniak-Chudek I, Cochat P, Dotsch J, Gajjar P, Hennekam RC, Janssen F, Kagan M, Kariminejad A, Kemper MJ, Koenig J, Kogan J, Kroes HY, Kuwertz-Broking E, Lewanda AF, Medeira A, Muscheites J, Niaudet P, Pierson M, Saggar A, Seaver L, Suri M, Tsygin A, Wuhl E, Zurowska A, Uebe S, Hildebrandt F, Antignac C, Zenker M (2010) Mutations in the human laminin beta2 (LAMB2) gene and the associated phenotypic spectrum. Human mutation 31:992-1002.

143. Braun DA, Rao J, Mollet G, Schapiro D, Daugeron MC, Tan W, Gribouval O (2017) Mutations in KEOPS-complex genes cause nephrotic syndrome with primary microcephaly. Pediatric nephrology (Berlin, Germany) 49:1529-1538.

144. Has C, Sparta G, Kiritsi D, Weibel L, Moeller A, Vega-Warner V, Waters A, He Y, Anikster Y, Esser P, Straub BK, Hausser I, Bockenhauer D, Dekel B, Hildebrandt F, Bruckner-Tuderman L, Laube GF (2012) Integrin alpha3 mutations with kidney, lung, and skin disease. The New England journal of medicine 366:1508-1514.

145. Kambham N, Tanji N, Seigle RL, Markowitz GS, Pulkkinen L, Uitto J, D'Agati VD (2000) Congenital focal segmental glomerulosclerosis associated with beta4 integrin mutation and epidermolysis bullosa. American journal of kidney diseases : the official journal of the National Kidney Foundation 36:190-196.

146. Altassan R, Witters P, Saifudeen Z, Quelhas D, Jaeken J, Levtchenko E, Cassiman D, Morava E (2018) Renal involvement in PMM2-CDG, a mini-review. Molecular genetics and metabolism 123:292-296.

147. Ng BG, Shiryaev SA, Rymen D, Eklund EA, Raymond K, Kircher M, Abdenur JE, Alehan F, Midro AT, Bamshad MJ, Barone R, Berry GT, Brumbaugh JE, Buckingham KJ, Clarkson K, Cole FS, O'Connor S, Cooper GM, Van Coster R, Demmer LA, Diogo L, Fay AJ, Ficicioglu C, Fiumara A, Gahl WA, Ganetzky R, Goel H, Harshman LA, He M, Jaeken J, James PM, Katz D, Keldermans L, Kibaek M, Kornberg AJ, Lachlan K, Lam C, Yaplito-Lee J, Nickerson DA, Peters HL, Race V, Regal L, Rush JS, Rutledge SL, Shendure J, Souche E, Sparks SE, Trapane P, Sanchez-Valle A, Vilain E, Vollo A, Waechter CJ, Wang RY, Wolfe LA, Wong DA, Wood T, Yang AC, Matthijs G, Freeze HH (2016) ALG1-CDG: Clinical and Molecular Characterization of 39 Unreported Patients. Human mutation 37:653-660.

148. Karamatic Crew V, Burton N, Kagan A, Green CA, Levene C, Flinter F, Brady RL, Daniels G, Anstee DJ (2004) CD151, the first member of the tetraspanin (TM4) superfamily detected on erythrocytes, is essential for the correct assembly of human basement membranes in kidney and skin. Blood 104:2217-2223.

149. Jaron R, Rosenfeld N, Zahdeh F, Carmi S, Beni-Adani L, Doviner V, Picard E, Segel R, Zeligson S, Carmel L, Renbaum P, Levy-Lahad E (2016) Expanding the phenotype of CRB2 mutations - A new ciliopathy syndrome? Clinical genetics 90:540-544.

150. Ebarasi L, Ashraf S, Bierzynska A, Gee HY, McCarthy HJ, Lovric S, Sadowski CE, Pabst W, Vega-Warner V, Fang H, Koziell A, Simpson MA, Dursun I, Serdaroglu E, Levy S, Saleem MA, Hildebrandt F, Majumdar A (2015) Defects of CRB2 cause steroid-resistant nephrotic syndrome. American journal of human genetics 96:153-161.

151. Emma F, Salviati L (2017) Mitochondrial cytopathies and the kidney. Nephrologie & therapeutique 13 Suppl 1:S23-s28.

152. Lovric S, Goncalves S, Gee HY, Oskouian B, Srinivas H, Choi WI, Shril S, Ashraf S, Tan W, Rao J, Airik M, Schapiro D, Braun DA, Sadowski CE, Widmeier E, Jobst-Schwan T, Schmidt JM, Girik V, Capitani G, Suh JH, Lachaussee N, Arrondel C, Patat J, Gribouval O, Furlano M, Boyer O, Schmitt A, Vuiblet V, Hashmi S, Wilcken R, Bernier FP, Innes AM, Parboosingh JS, Lamont RE, Midgley JP, Wright N, Majewski J, Zenker M, Schaefer F, Kuss N, Greil J, Giese T, Schwarz K, Catheline V, Schanze D, Franke I, Sznajer Y, Truant AS, Adams B, Desir J, Biemann R, Pei Y, Ars E, Lloberas N, Madrid A, Dharnidharka VR, Connolly AM, Willing MC, Cooper MA, Lifton RP, Simons M, Riezman H, Antignac C, Saba JD, Hildebrandt F (2017) Mutations in sphingosine-1-phosphate lyase cause nephrosis with ichthyosis and adrenal insufficiency. Nature genetics 127:912-928.

153. Prasad R, Hadjidemetriou I, Maharaj A, Meimaridou E, Buonocore F, Saleem M, Hurcombe J, Bierzynska A, Barbagelata E, Bergada I, Cassinelli H, Das U, Krone R, Hacihamdioglu B, Sari E, Yesilkaya E, Storr HL, Clemente M, Fernandez-Cancio M, Camats N, Ram N, Achermann JC, Van Veldhoven PP, Guasti L, Braslavsky D, Guran T, Metherell LA (2017) Sphingosine-1-phosphate lyase mutations cause primary adrenal insufficiency and steroid-resistant nephrotic syndrome. F1000Research 127:942-953.

154. Kashtan CE, Ding J, Garosi G, Heidet L, Massella L, Nakanishi K, Nozu K, Renieri A, Rheault M, Wang F, Gross O (2018) Alport syndrome: a unified classification of genetic disorders of collagen IV alpha345: a position paper of the Alport Syndrome Classification Working Group. Kidney international 93:1045-1051.

155. Boyer O, Nevo F, Plaisier E, Funalot B, Gribouval O, Benoit G, Huynh Cong E, Arrondel C, Tete MJ, Montjean R, Richard L, Karras A, Pouteil-Noble C, Balafrej L, Bonnardeaux A, Canaud G, Charasse C, Dantal J, Deschenes G, Deteix P, Dubourg O, Petiot P, Pouthier D, Leguern E, Guiochon-Mantel A, Broutin I, Gubler MC, Saunier S, Ronco P, Vallat JM, Alonso MA, Antignac C, Mollet G (2011) INF2 mutations in Charcot-Marie-Tooth disease with glomerulopathy. The New England journal of medicine 365:2377-2388.

156. Sweeney E, Fryer A, Mountford R, Green A, McIntosh I (2003) Nail patella syndrome: a review of the phenotype aided by developmental biology. Journal of medical genetics 40:153-162.

157. Bongers EM, Huysmans FT, Levtchenko E, de Rooy JW, Blickman JG, Admiraal RJ, Huygen PL, Cruysberg JR, Toolens PA, Prins JB, Krabbe PF, Borm GF, Schoots J, van Bokhoven H, van Remortele AM, Hoefsloot LH, van Kampen A, Knoers NV (2005) Genotype-phenotype studies in nail-patella syndrome show that LMX1B mutation location is involved in the risk of developing nephropathy. European journal of human genetics : EJHG 13:935-946.

158. Saraiva JM, Dinis A, Resende C, Faria E, Gomes C, Correia AJ, Gil J, da Fonseca N (1999) Schimke immuno-osseous dysplasia: case report and review of 25 patients. Journal of medical genetics 36:786-789.

159. Boerkoel CF, O'Neill S, Andre JL, Benke PJ, Bogdanovic R, Bulla M, Burguet A, Cockfield S, Cordeiro I, Ehrich JH, Frund S, Geary DF, Ieshima A, Illies F, Joseph MW, Kaitila I, Lama G, Leheup B, Ludman MD, McLeod DR, Medeira A, Milford DV, Ormala T, Rener-Primec Z, Santava A, Santos HG, Schmidt B, Smith GC, Spranger J, Zupancic N, Weksberg R (2000) Manifestations and treatment of Schimke immuno-osseous dysplasia: 14 new cases and a review of the literature. European journal of pediatrics 159:1-7.

160. Simha V, Agarwal AK, Oral EA, Fryns JP, Garg A (2003) Genetic and phenotypic heterogeneity in patients with mandibuloacral dysplasia-associated lipodystrophy. The Journal of clinical endocrinology and metabolism 88:2821-2824.

161. Berkovic SF, Dibbens LM, Oshlack A, Silver JD, Katerelos M, Vears DF, Lullmann-Rauch R, Blanz J, Zhang KW, Stankovich J, Kalnins RM, Dowling JP, Andermann E, Andermann F, Faldini E, D'Hooge R, Vadlamudi L, Macdonell RA, Hodgson BL, Bayly MA, Savige J, Mulley JC, Smyth GK, Power DA, Saftig P, Bahlo M (2008) Array-based gene discovery with three unrelated subjects shows SCARB2/LIMP-2 deficiency causes myoclonus epilepsy and glomerulosclerosis. American journal of human genetics 82:673-684.

162. Dibbens L, Schwake M, Saftig P, Rubboli G (2016) SCARB2/LIMP2 deficiency in action myoclonus-renal failure syndrome. Epileptic disorders : international epilepsy journal with videotape 18:63-72.

163. Bostrom MA, Freedman BI (2010) The spectrum of MYH9-associated nephropathy. Clinical journal of the American Society of Nephrology : CJASN 5:1107-1113.

164. Oh T, Jung Seo H, Taek Lee K, Jo Kim H, Jun Kim H, Lee JH, Il Cheong H, Young Lee E (2015) MYH9 nephropathy. Kidney research and clinical practice 34:53-56.

165. Flynn JT, Kaelber DC, Baker-Smith CM, Blowey D, Carroll AE, Daniels SR, de Ferranti SD, Dionne JM, Falkner B, Flinn SK, Gidding SS, Goodwin C, Leu MG, Powers ME, Rea C, Samuels J, Simasek M, Thaker VV, Urbina EM (2017) Clinical Practice Guideline for Screening and Management of High Blood Pressure in Children and Adolescents. Pediatrics 140.

166. (1981) The primary nephrotic syndrome in children. Identification of patients with minimal change nephrotic syndrome from initial response to prednisone. A report of the International Study of Kidney Disease in Children. The Journal of pediatrics 98:561-564.

167. Lombel RM, Hodson EM, Gipson DS (2013) Treatment of steroid-resistant nephrotic syndrome in children: new guidelines from KDIGO. Pediatric nephrology (Berlin, Germany) 28:409-414.

168. Feber J, Al-Matrafi J, Farhadi E, Vaillancourt R, Wolfish N (2009) Prednisone dosing per body weight or body surface area in children with nephrotic syndrome: is it equivalent? Pediatric nephrology (Berlin, Germany) 24:1027-1031.

169. Emma F, Montini G, Gargiulo A (2019) Equations to estimate prednisone dose using body weight. Pediatric nephrology (Berlin, Germany) 34:685-688.

170. Raman V, Krishnamurthy S, Harichandrakumar KT (2016) Body weight-based prednisolone versus body surface area-based prednisolone regimen for induction of remission in children with nephrotic syndrome: a randomized, open-label, equivalence clinical trial. Pediatric nephrology (Berlin, Germany) 31:595-604.

171. Hari P, Bagga A, Mantan M (2004) Short term efficacy of intravenous dexamethasone and methylprednisolone therapy in steroid resistant nephrotic syndrome. Indian pediatrics 41:993-1000.

172. Mori K, Honda M, Ikeda M (2004) Efficacy of methylprednisolone pulse therapy in steroid-resistant nephrotic syndrome. Pediatric nephrology (Berlin, Germany) 19:1232-1236.

173. Yorgin PD, Krasher J, Al-Uzri AY (2001) Pulse methylprednisolone treatment of idiopathic steroid-resistant nephrotic syndrome. Pediatric nephrology (Berlin, Germany) 16:245-250.

174. Waldo FB, Benfield MR, Kohaut EC (1992) Methylprednisolone treatment of patients with steroid-resistant nephrotic syndrome. Pediatric nephrology (Berlin, Germany) 6:503-505.

175. Imbasciati E, Gusmano R, Edefonti A, Zucchelli P, Pozzi C, Grassi C, Della Volpe M, Perfumo F, Petrone P, Picca M, et al. (1985) Controlled trial of methylprednisolone pulses and low dose oral prednisone for the minimal change nephrotic syndrome. British medical journal (Clinical research ed) 291:1305-1308.

176. Murnaghan K, Vasmant D, Bensman A (1984) Pulse methylprednisolone therapy in severe idiopathic childhood nephrotic syndrome. Acta paediatrica Scandinavica 73:733-739.

177. (1982) Early identification of frequent relapsers among children with minimal change nephrotic syndrome. A report of the International Study of Kidney Disease in Children. The Journal of pediatrics 101:514-518.

178. Corwin HL, Schwartz MM, Lewis EJ (1988) The importance of sample size in the interpretation of the renal biopsy. American journal of nephrology 8:85-89.

179. Chang A, Gibson IW, Cohen AH, Weening JJ, Jennette JC, Fogo AB (2012) A position paper on standardizing the nonneoplastic kidney biopsy report. Clinical journal of the American Society of Nephrology : CJASN 7:1365-1368.

180. Fuiano G, Comi N, Magri P, Sepe V, Balletta MM, Esposito C, Uccello F, Dal Canton A, Conte G (1996) Serial morphometric analysis of sclerotic lesions in primary "focal" segmental glomerulosclerosis. Journal of the American Society of Nephrology : JASN 7:49-55.

181. Meehan SM, Chang A, Gibson IW, Kim L, Kambham N, Laszik Z (2013) A study of interobserver reproducibility of morphologic lesions of focal segmental glomerulosclerosis. Virchows Archiv : an international journal of pathology 462:229-237.

182. Silverstein DM, Craver R (2007) Presenting features and short-term outcome according to pathologic variant in childhood primary focal segmental glomerulosclerosis. Clinical journal of the American Society of Nephrology : CJASN 2:700-707.

183. Thomas DB, Franceschini N, Hogan SL, Ten Holder S, Jennette CE, Falk RJ, Jennette JC (2006) Clinical and pathologic characteristics of focal segmental glomerulosclerosis pathologic variants. Kidney international 69:920-926.

184. Paik KH, Lee BH, Cho HY, Kang HG, Ha IS, Cheong HI, Jin DK, Moon KC, Choi Y (2007) Primary focal segmental glomerular sclerosis in children: clinical course and prognosis. Pediatric nephrology (Berlin, Germany) 22:389-395.

185. Sethi S, Glassock RJ, Fervenza FC (2015) Focal segmental glomerulosclerosis: towards a better understanding for the practicing nephrologist. Nephrology, dialysis, transplantation : official publication of the European Dialysis and Transplant Association - European Renal Association 30:375-384.

186. Hotta O, Inoue CN, Miyabayashi S, Furuta T, Takeuchi A, Taguma Y (2001) Clinical and pathologic features of focal segmental glomerulosclerosis with mitochondrial tRNALeu(UUR) gene mutation. Kidney international 59:1236-1243.

187. Henderson JM, Alexander MP, Pollak MR (2009) Patients with ACTN4 mutations demonstrate distinctive features of glomerular injury. Journal of the American Society of Nephrology : JASN 20:961-968.

188. Brown EJ, Schlondorff JS, Becker DJ, Tsukaguchi H, Tonna SJ, Uscinski AL, Higgs HN, Henderson JM, Pollak MR (2010) Mutations in the formin gene INF2 cause focal segmental glomerulosclerosis. Nature genetics 42:72-76.

189. Gast C, Pengelly RJ, Lyon M, Bunyan DJ, Seaby EG, Graham N, Venkat-Raman G, Ennis S (2016) Collagen (COL4A) mutations are the most frequent mutations underlying adult focal segmental glomerulosclerosis. Nephrology, dialysis, transplantation : official publication of the European Dialysis and Transplant Association - European Renal Association 31:961-970.

190. Schumacher V, Scharer K, Wuhl E, Altrogge H, Bonzel KE, Guschmann M, Neuhaus TJ, Pollastro RM, Kuwertz-Broking E, Bulla M, Tondera AM, Mundel P, Helmchen U, Waldherr R, Weirich A, Royer-Pokora B (1998) Spectrum of early onset nephrotic syndrome associated with WT1 missense mutations. Kidney international 53:1594-1600.

191. Zenker M, Aigner T, Wendler O, Tralau T, Muntefering H, Fenski R, Pitz S, Schumacher V, Royer-Pokora B, Wuhl E, Cochat P, Bouvier R, Kraus C, Mark K, Madlon H, Dotsch J, Rascher W, Maruniak-Chudek I, Lennert T, Neumann LM, Reis A (2004) Human laminin beta2 deficiency causes congenital nephrosis with mesangial sclerosis and distinct eye abnormalities. Human molecular genetics 13:2625-2632.

192. Gbadegesin R, Hinkes BG, Hoskins BE, Vlangos CN, Heeringa SF, Liu J, Loirat C, Ozaltin F, Hashmi S, Ulmer F, Cleper R, Ettenger R, Antignac C, Wiggins RC, Zenker M, Hildebrandt F (2008) Mutations in PLCE1 are a major cause of isolated diffuse mesangial sclerosis (IDMS). Nephrology, dialysis, transplantation : official publication of the European Dialysis and Transplant Association - European Renal Association 23:1291-1297.
